# Supplementary material for: Perceptions, barriers, and facilitators of maternal health service utilization in southern Ethiopia: A qualitative exploration of community members’ and health care providers’ views
Source: PLoS One. 2024 Dec 19;19(12):e0312484. doi: 10.1371/journal.pone.0312484 (PMC11658624; doi:10.1371/journal.pone.0312484)
Supplement: S1 File — (DOCX) [file pone.0312484.s001.docx]

**Module 1: IDI Guide (English version)**

| **I** | **Section I: Identification** | |
| --- | --- | --- |
| 1 | Questionnaire ID | **____________________________** |
| 2 | Name of Woreda | **____________________________** |
| 3 | Name of Kebele | **____________________________________** |
| 4 | Name of moderator | **_______________________________** |
| 5 | Name of note taker | **_________________________________** |
| 6 | Date of discussion | **_______________________________** |
| 7 | Start time: | **______:________** |
| 8 | End time: | **____:______** |

| **II** | **Section II: Participant Demographic Intake Sheet** | | | | | |
| --- | --- | --- | --- | --- | --- | --- |
| 1 | Participant code |  |  |  |  |  |
| 2 | Age |  |  |  |  |  |
| 3 | Religion |  |  |  |  |  |
| 4 | Marital status |  |  |  |  |  |
| 5 | Are you employed? (Yes/No) |  |  |  |  |  |
| 6 | Educational level |  |  |  |  |  |
| **For recently delivered women** | | | | | | |
| 7 | Gravidity |  |  |  |  |  |
| 8 | Parity |  |  |  |  |  |
| 9 | Place of delivery in last pregnancy (facility/home) |  |  |  |  |  |

**Interview guide**

**Antepartum for all participants**

1. **The practice of ANC**
2. How early do women go for ANC? **Probe** why do they go at that time? Why earlier or later?
3. How often do they go to ANC? **Probe** why do they go at that time?
4. Do women think skilled attendance during pregnancy helps their pregnancy?
5. **Reasons for use of ANC**
6. Explain factors that would motivate women to utilize ANC service in their pregnancy?
7. **Barriers for attending ANC use**
8. If women do not go for ANC, what are their reasons?
9. What are barriers to accessing ANC? **Probe** for;
10. Financial barriers and opportunity costs
11. Distance and access
12. Socio-cultural
13. Quality of care
14. **Reasons for discontinuation across the continuum**
15. Why do women go to the facility for first ANC, but discontinue for subsequent ANC visits?

**Probe** for;

1. Financial barriers and opportunity costs
2. Distance and access
3. Socio-cultural
4. Quality of care
5. **Traditional practices during pregnancy**
6. Can you tell us about the traditional practices and beliefs during pregnancy?
7. Do you think these traditional beliefs, religious practices, and cultural norms affect mothers to use care during pregnancy? Explain how and why?
8. How do you see community volunteers/TBAs and health professionals and maternal health services provided to the community?
9. **For recently delivered mothers only**
10. How do you rate the quality of care you received during ANC follow-up? What kinds of services do you receive in ANC? Are you satisfied?
11. If the mother received ANC; **Ask:** Explain factors that motivate you to utilize ANC service in their pregnancy
12. If the mother did not go for ANC; **Ask**: what are the reasons not attending ANC services?
13. Explain the support you get from the community to and decision making on health services during pregnancy.
14. Explain us your experiences relating to the utilization of ANC care provided by skilled birth attendants. Prove for;
15. their interactions with skilled birth attendants during ANC,
16. their confidence in skilled birth attendants’ abilities, and
17. respect and compassion of attendants (respect for the traditional beliefs of the women, etc)
18. **For community religious leaders and community volunteers only**

**Community perceptions about health providers and maternal health programs**

1. How the communities see the maternal health programs and health professionals? Tell me the perception about maternal health care services. Perception about different care providers.
2. What efforts your community made to increase maternal health service in your community?
3. **In your opinion, what should be improved regarding ANC services?**

**Intrapartum for all participants**

1. **The practice of facility delivery**
2. Do women think skilled attendance during childbirth helps themselves and their babies?
3. **Reasons for use of facility delivery**
4. Explain factors that would motivate women to utilize delivery service in their pregnancy

**Probe** for reasons for using continuum of care

1. **Barriers for attending facility delivery**
2. If women deliver at home, what are their reasons? Explain the constraints that influenced women to utilize facility delivery services?

**Probe** for;

1. Financial barriers and opportunity costs
2. Distance and access
3. Socio-cultural
4. Quality of care and non-dignified care
5. **Reasons for discontinuation across the continuum**
6. Why do women go to the facility for ANC, yet mostly deliver at home?

**Probe** for;

1. Financial barriers and opportunity costs
2. Distance and access
3. Socio-cultural
4. Quality of care and non-dignified care
5. **Traditional practices during intrapartum period**
6. Can you tell us about the traditional practices and beliefs during childbirth?
7. Do you think these traditional beliefs, religious practices, and cultural norms affect mothers to use care during delivery? Explain how and why?
8. How do you see community volunteers/TBAs and health professionals and maternal health services provided to the community?
9. **For recently delivered mothers only**
10. How do you rate the quality of care you received from the facility during childbirth?
11. What kinds of services do you receive in childbirth? Are you satisfied?
12. If delivered in health facility; **Ask:** Explain factors that motivate you to deliver in health facility
13. For home delivered mothers; **Ask:** what does she think are the obstacles when accessing a health care facility? Her reasons for discontinuation?
14. Explain the support you get from the community to and decision making on health services during delivery
15. Explain us your experiences relating to the utilization of facility delivery care provided by skilled birth attendants. Prove for;
16. their interactions with skilled birth attendants during facility delivery,
17. their confidence in skilled birth attendants’ abilities, and
18. respect and compassion of attendants (respect for the traditional beliefs of the women, etc)
19. **For community religious leaders and community volunteers only**

**Community perceptions about health providers and maternal health programs**

1. How the communities see the maternal health programs and health professionals? Tell me the perception about maternal health care services. Perception about different care providers.
2. What efforts your community made to increase maternal health service in your community?
3. **In your opinion, what should be improved regarding facility delivery services? Continuity of care?**

**Postpartum for all participants**

1. **The practice of PNC**

1. How early do women go for PNC? **Probe** why do they go at that time? Why earlier or later?

1. How often do they go to PNC? **Probe** why do they go at that time?
2. Do women think skilled attendance during postpartum helps their babies and themselves?
3. **Reasons for use of PNC**
4. Explain factors that would motivate women to utilize PNC service in their childbirth

**Probe** for reasons for using continuum of care

1. **Barriers for attending PNC use**
2. If women don’t go for PNC, what are their reasons?
3. What are barriers to accessing PNC? **Probe** for;
4. Financial barriers and opportunity costs
5. Distance and access
6. Socio-cultural
7. Quality of care
8. **Reasons for discontinuation across the continuum**
9. Why do women go to the delivery at the facility, yet mostly don’t receive PNC?
10. Explain the obstacles influenced women to utilize skilled care during postpartum in your community?

**Probe** for;

1. Financial barriers and opportunity costs
2. Distance and access
3. Socio-cultural
4. Quality of care
5. **Traditional practices during pregnancy**
6. Can you tell us about the traditional practices and beliefs during postpartum period?
7. Do you think these traditional beliefs, religious practices, and cultural norms affect mothers to use care during postpartum period? Explain how and why?
8. How do you see community volunteers/TBAs and health professionals and maternal health services provided to the community?
9. **For recently delivered mothers only**
10. How do you rate the quality of care you received during PNC follow-up? What kinds of services do you receive in PNC? Are you satisfied?
11. If use PNC: **Ask:** Explain factors that motivate you to utilize PNC service?
12. If women do not go for PNC; **Ask:** what are the reasons for not getting PNC? And what are the reasons for discontinuation?
13. Explain the support you get from the community to and decision making on health services during postpartum period?
14. Explain us your experiences relating to the utilization of PNC care provided by skilled birth attendants. Prove for;
15. their interactions with skilled birth attendants during PNC,
16. their confidence in skilled birth attendants’ abilities, and
17. respect and compassion of attendants (respect for the traditional beliefs of the women, etc)
18. **For community religious leaders and community volunteers only**

**Community perceptions about health providers and maternal health programs**

1. How the communities see the maternal health programs and health professionals? Tell me the perception about maternal health care services. Perception about different care providers.
2. What efforts your community made to increase maternal health service in your community?
3. **In your opinion, what should be improved regarding PNC services? Continuum of care?**

**Thank you for your participation!!!**

**Module 2: FGD guide (English version)**

| **I** | **Section I: Identification** | |
| --- | --- | --- |
| 1 | Questionnaire ID | **____________________________** |
| 2 | Name of Woreda | **____________________________** |
| 3 | Name of Kebele | **____________________________________** |
| 4 | Name of moderator | **_______________________________** |
| 5 | Name of note taker | **_________________________________** |
| 6 | Date of discussion | **_______________________________** |
| 7 | Start time: | **______:________** |
| 8 | End time: | **____:______** |

**Discussion guide**

**Antepartum for all participants**

1. **The practice of ANC**

1. How early do women go for ANC? **Probe** why do they go at that time? Why earlier or later?

1. How often do they go to ANC? **Probe** why do they go at that time?
2. Do women think skilled attendance during pregnancy helps their pregnancy?
3. **Reasons for use of ANC**
4. Explain factors that would motivate women to utilize ANC service in their pregnancy?
5. **Barriers for attending ANC use**
6. If women do not go for ANC, what are their reasons?
7. What are barriers to accessing ANC? **Probe** for;
8. Financial barriers and opportunity costs
9. Distance and access
10. Socio-cultural
11. Quality of care
12. **Reasons for discontinuation across the continuum**
13. Why do women go to the facility for first ANC, but discontinue for subsequent ANC visits?

**Probe** for;

1. Financial barriers and opportunity costs
2. Distance and access
3. Socio-cultural
4. Quality of care
5. **Traditional practices during pregnancy**
6. Can you tell us about the traditional practices and beliefs during pregnancy?
7. Do you think these traditional beliefs, religious practices, and cultural norms affect mothers to use care during pregnancy? Explain how and why?
8. How do you see community volunteers/TBAs and health professionals and maternal health services provided to the community?
9. **For recently delivered mothers only**
10. How do you rate the quality of care you received during ANC follow-up? What kinds of services do you receive in ANC? Are you satisfied?
11. If the mother received ANC; **Ask:** Explain factors that motivate you to utilize ANC service in their pregnancy
12. If the mother did not go for ANC; **Ask**: what are the reasons not attending ANC services?
13. Explain the support you get from the community to and decision making on health services during pregnancy.
14. Explain us your experiences relating to the utilization of ANC care provided by skilled birth attendants. Prove for;
15. their interactions with skilled birth attendants during ANC,
16. their confidence in skilled birth attendants’ abilities, and
17. respect and compassion of attendants (respect for the traditional beliefs of the women, etc)
18. **For community religious leaders and community volunteers only**

**Community perceptions about health providers and maternal health programs**

1. How the communities see the maternal health programs and health professionals? Tell me the perception about maternal health care services. Perception about different care providers.
2. What efforts your community made to increase maternal health service in your community?
3. **In your opinion, what should be improved regarding ANC services?**

**Intrapartum for all participants**

1. **The practice of facility delivery**

1. Do women think skilled attendance during childbirth helps themselves and their babies?

1. **Reasons for use of facility delivery**
2. Explain factors that would motivate women to utilize delivery service in their pregnancy

**Probe** for reasons for using continuum of care

1. **Barriers for attending facility delivery**
2. If women deliver at home, what are their reasons? Explain the constraints that influenced women to utilize facility delivery services?

**Probe** for;

1. Financial barriers and opportunity costs
2. Distance and access
3. Socio-cultural
4. Quality of care and non-dignified care
5. **Reasons for discontinuation across the continuum**
6. Why do women go to the facility for ANC, yet mostly deliver at home?

**Probe** for;

1. Financial barriers and opportunity costs
2. Distance and access
3. Socio-cultural
4. Quality of care and non-dignified care
5. **Traditional practices during intrapartum period**
6. Can you tell us about the traditional practices and beliefs during childbirth?
7. Do you think these traditional beliefs, religious practices, and cultural norms affect mothers to use care during delivery? Explain how and why?
8. How do you see community volunteers/TBAs and health professionals and maternal health services provided to the community?
9. **For recently delivered mothers only**
10. How do you rate the quality of care you received from the facility during childbirth?
11. What kinds of services do you receive in childbirth? Are you satisfied?
12. If delivered in health facility; **Ask:** Explain factors that motivate you to deliver in health facility
13. For home delivered mothers; **Ask:** what does she think are the obstacles when accessing a health care facility? Her reasons for discontinuation?
14. Explain the support you get from the community to and decision making on health services during delivery
15. Explain us your experiences relating to the utilization of facility delivery care provided by skilled birth attendants. Prove for;
16. their interactions with skilled birth attendants during facility delivery,
17. their confidence in skilled birth attendants’ abilities, and
18. respect and compassion of attendants (respect for the traditional beliefs of the women, etc)
19. **For community religious leaders and community volunteers only**

**Community perceptions about health providers and maternal health programs**

1. How the communities see the maternal health programs and health professionals? Tell me the perception about maternal health care services. Perception about different care providers.
2. What efforts your community made to increase maternal health service in your community?
3. **In your opinion, what should be improved regarding facility delivery services? Continuity of care?**

**Postpartum for all participants**

**I. The practice of PNC**

1. How early do women go for PNC? **Probe** why do they go at that time? Why earlier or later?

1. How often do they go to PNC? **Probe** why do they go at that time?
2. Do women think skilled attendance during postpartum helps their babies and themselves?
3. **Reasons for use of PNC**
4. Explain factors that would motivate women to utilize PNC service in their childbirth

**Probe** for reasons for using continuum of care

1. **Barriers for attending PNC use**
2. If women don’t go for PNC, what are their reasons?
3. What are barriers to accessing PNC? **Probe** for;
4. Financial barriers and opportunity costs
5. Distance and access
6. Socio-cultural
7. Quality of care
8. **Reasons for discontinuation across the continuum**
9. Why do women go to the delivery at the facility, yet mostly don’t receive PNC?
10. Explain the obstacles influenced women to utilize skilled care during postpartum in your community?

**Probe** for;

1. Financial barriers and opportunity costs
2. Distance and access
3. Socio-cultural
4. Quality of care
5. **Traditional practices during pregnancy**
6. Can you tell us about the traditional practices and beliefs during postpartum period?
7. Do you think these traditional beliefs, religious practices, and cultural norms affect mothers to use care during postpartum period? Explain how and why?
8. How do you see community volunteers/TBAs and health professionals and maternal health services provided to the community?
9. **For recently delivered mothers only**
10. How do you rate the quality of care you received during PNC follow-up? What kinds of services do you receive in PNC? Are you satisfied?
11. If use PNC: **Ask:** Explain factors that motivate you to utilize PNC service?
12. If women do not go for PNC; **Ask:** what are the reasons for not getting PNC? And what are the reasons for discontinuation?
13. Explain the support you get from the community to and decision making on health services during postpartum period?
14. Explain us your experiences relating to the utilization of PNC care provided by skilled birth attendants. Prove for;
15. their interactions with skilled birth attendants during PNC,
16. their confidence in skilled birth attendants’ abilities, and
17. respect and compassion of attendants (respect for the traditional beliefs of the women, etc)
18. **For community religious leaders and community volunteers only**

**Community perceptions about health providers and maternal health programs**

1. How the communities see the maternal health programs and health professionals? Tell me the perception about maternal health care services. Perception about different care providers.
2. What efforts your community made to increase maternal health service in your community?
3. **In your opinion, what should be improved regarding PNC services? Continuum of care?**

**Thank you for your participation!!!**

**Module 3: KII guide (English version)**

| **I** | **Section I: Identification** | |
| --- | --- | --- |
| 1 | Questionnaire ID | **____________________________** |
| 2 | Name of Woreda | **____________________________** |
| 3 | Name of Kebele | **____________________________________** |
| 4 | Name of moderator | **_______________________________** |
| 5 | Name of note taker | **_________________________________** |
| 6 | Date of discussion | **_______________________________** |
| 7 | Start time: | **______:________** |
| 8 | End time: | **____:______** |

**KII guide**

**Antepartum for all participants**

1. **The practice of ANC**

1. How early do women go for ANC? **Probe** why do they go at that time? Why earlier or later?

1. How often do they go to ANC? **Probe** why do they go at that time?
2. Do women think skilled attendance during pregnancy helps their pregnancy?
3. **Reasons for use of ANC**
4. Explain factors that would motivate women to utilize ANC service in their pregnancy?
5. **Barriers for attending ANC use**
6. If women do not go for ANC, what are their reasons?
7. What are barriers to accessing ANC? **Probe** for;
8. Financial barriers and opportunity costs
9. Distance and access
10. Socio-cultural
11. Quality of care
12. **Reasons for discontinuation across the continuum**
13. Why do women go to the facility for first ANC, but discontinue for subsequent ANC visits?

**Probe** for;

1. Financial barriers and opportunity costs
2. Distance and access
3. Socio-cultural
4. Quality of care
5. **Traditional practices during pregnancy**
6. Can you tell us about the traditional practices and beliefs during pregnancy?
7. Do you think these traditional beliefs, religious practices, and cultural norms affect mothers to use care during pregnancy? Explain how and why?
8. How do you see community volunteers/TBAs and health professionals and maternal health services provided to the community?
9. **For recently delivered mothers only**
10. How do you rate the quality of care you received during ANC follow-up? What kinds of services do you receive in ANC? Are you satisfied?
11. If the mother received ANC; **Ask:** Explain factors that motivate you to utilize ANC service in their pregnancy
12. If the mother did not go for ANC; **Ask**: what are the reasons not attending ANC services?
13. Explain the support you get from the community to and decision making on health services during pregnancy.
14. Explain us your experiences relating to the utilization of ANC care provided by skilled birth attendants. Prove for;
15. their interactions with skilled birth attendants during ANC,
16. their confidence in skilled birth attendants’ abilities, and
17. respect and compassion of attendants (respect for the traditional beliefs of the women, etc)
18. **For community religious leaders and community volunteers only**

**Community perceptions about health providers and maternal health programs**

1. How the communities see the maternal health programs and health professionals? Tell me the perception about maternal health care services. Perception about different care providers.
2. What efforts your community made to increase maternal health service in your community?
3. **In your opinion, what should be improved regarding ANC services?**

**Intrapartum for all participants**

1. **The practice of facility delivery**

1. Do women think skilled attendance during childbirth helps themselves and their babies?

1. **Reasons for use of facility delivery**
2. Explain factors that would motivate women to utilize delivery service in their pregnancy

**Probe** for reasons for using continuum of care

1. **Barriers for attending facility delivery**
2. If women deliver at home, what are their reasons? Explain the constraints that influenced women to utilize facility delivery services?

**Probe** for;

1. Financial barriers and opportunity costs
2. Distance and access
3. Socio-cultural
4. Quality of care and non-dignified care
5. **Reasons for discontinuation across the continuum**
6. Why do women go to the facility for ANC, yet mostly deliver at home?

**Probe** for;

1. Financial barriers and opportunity costs
2. Distance and access
3. Socio-cultural
4. Quality of care and non-dignified care
5. **Traditional practices during intrapartum period**
6. Can you tell us about the traditional practices and beliefs during childbirth?
7. Do you think these traditional beliefs, religious practices, and cultural norms affect mothers to use care during delivery? Explain how and why?
8. How do you see community volunteers/TBAs and health professionals and maternal health services provided to the community?
9. **For recently delivered mothers only**
10. How do you rate the quality of care you received from the facility during childbirth?
11. What kinds of services do you receive in childbirth? Are you satisfied?
12. If delivered in health facility; **Ask:** Explain factors that motivate you to deliver in health facility
13. For home delivered mothers; **Ask:** what does she think are the obstacles when accessing a health care facility? Her reasons for discontinuation?
14. Explain the support you get from the community to and decision making on health services during delivery
15. Explain us your experiences relating to the utilization of facility delivery care provided by skilled birth attendants. Prove for;
16. their interactions with skilled birth attendants during facility delivery,
17. their confidence in skilled birth attendants’ abilities, and
18. respect and compassion of attendants (respect for the traditional beliefs of the women, etc)
19. **For community religious leaders and community volunteers only**

**Community perceptions about health providers and maternal health programs**

1. How the communities see the maternal health programs and health professionals? Tell me the perception about maternal health care services. Perception about different care providers.
2. What efforts your community made to increase maternal health service in your community?
3. **In your opinion, what should be improved regarding facility delivery services? Continuity of care?**

**Postpartum for all participants**

1. **The practice of PNC**

1. How early do women go for PNC? **Probe** why do they go at that time? Why earlier or later?

1. How often do they go to PNC? **Probe** why do they go at that time?
2. Do women think skilled attendance during postpartum helps their babies and themselves?
3. **Reasons for use of PNC**
4. Explain factors that would motivate women to utilize PNC service in their childbirth

**Probe** for reasons for using continuum of care

1. **Barriers for attending PNC use**
2. If women don’t go for PNC, what are their reasons?
3. What are barriers to accessing PNC? **Probe** for;
4. Financial barriers and opportunity costs
5. Distance and access
6. Socio-cultural
7. Quality of care
8. **Reasons for discontinuation across the continuum**
9. Why do women go to the delivery at the facility, yet mostly don’t receive PNC?
10. Explain the obstacles influenced women to utilize skilled care during postpartum in your community?

**Probe** for;

1. Financial barriers and opportunity costs
2. Distance and access
3. Socio-cultural
4. Quality of care
5. **Traditional practices during pregnancy**
6. Can you tell us about the traditional practices and beliefs during postpartum period?
7. Do you think these traditional beliefs, religious practices, and cultural norms affect mothers to use care during postpartum period? Explain how and why?
8. How do you see community volunteers/TBAs and health professionals and maternal health services provided to the community?
9. **For recently delivered mothers only**
10. How do you rate the quality of care you received during PNC follow-up? What kinds of services do you receive in PNC? Are you satisfied?
11. If use PNC: **Ask:** Explain factors that motivate you to utilize PNC service?
12. If women do not go for PNC; **Ask:** what are the reasons for not getting PNC? And what are the reasons for discontinuation?
13. Explain the support you get from the community to and decision making on health services during postpartum period?
14. Explain us your experiences relating to the utilization of PNC care provided by skilled birth attendants. Prove for;
15. their interactions with skilled birth attendants during PNC,
16. their confidence in skilled birth attendants’ abilities, and
17. respect and compassion of attendants (respect for the traditional beliefs of the women, etc)
18. **For community religious leaders and community volunteers only**

**Community perceptions about health providers and maternal health programs**

1. How the communities see the maternal health programs and health professionals? Tell me the perception about maternal health care services. Perception about different care providers.
2. What efforts your community made to increase maternal health service in your community?
3. **In your opinion, what should be improved regarding PNC services? Continuum of care?**

**Thank you for your participation!!!**
