# Supplementary material for: Perceptions, barriers, and facilitators of maternal health service utilization in southern Ethiopia: A qualitative exploration of community members’ and health care providers’ views
Source: PLoS One. 2024 Dec 19;19(12):e0312484. doi: 10.1371/journal.pone.0312484 (PMC11658624; doi:10.1371/journal.pone.0312484)
Supplement: S2 File — (DOCX) [file pone.0312484.s002.docx]

**1. Ama -Amate Qormishshi Borri-xa’mo Massago (Sidaamu Afii Gano)**

| **I** | **Gafa I: Ayimmate Xawishsha** | |
| --- | --- | --- |
| 1 | Borri-xa’mote Badooshshi Kiiro | **____________________________** |
| 2 | Woradu Su’ma | **____________________________** |
| 3 | Olluu su’ma | **____________________________________** |
| 4 | Mereerima Su’ma (moderate) | **_______________________________** |
| 5 | Qaagiishsha amadannohu/te su’ma | **_________________________________** |
| 6 | Hasaawa barra | **_______________________________** |
| 7 | Hanafote yanna: | **______:________** |
| 8 | Gumulshu yanna: | **____:______** |

| **II** | **Gafa II: Beeqqisiisaancho Mannoomitte Taje** | | | | | |
| --- | --- | --- | --- | --- | --- | --- |
| 1 | Beeqqo assinoonni koodde |  |  |  |  |  |
| 2 | Diro |  |  |  |  |  |
| 3 | Amma’nokki |  |  |  |  |  |
| 4 | Adhamate ikkito |  |  |  |  |  |
| 5 | Mootimmate woy hallanyaho loosata/to? (Ee/Dee’ni) |  |  |  |  |  |
| 6 | Rosu deerra |  |  |  |  |  |
| **MULE ILTINO AMARA CALLA** | | | | | | |
| 7 | Me’’e higge godowootta |  |  |  |  |  |
| 8 | Meu qaaqquulli noohe |  |  |  |  |  |
| 9 | Konne gedensiidikki qaaqqo hiikko ilitta? Mine/fayyimmate uurrinshara? |  |  |  |  |  |

**Qaali-xa’mote Massago**

**Godombbanni yannara wo’munku ha’runsaanora**

1. **Godombanni yanna uurrinshate noo owaante rosicho**
2. Godowinni no ama ilate balaxote ha’runso hiittoonni hadhe afidhanno fayyimmate owaante? Seekkite xa’mitinanni yannara mayira balaxxe woy gedensaanni hadhanno fayyimmate uurrishsha?
3. Ilate balaxote ha’runso me’’e hige hadhanno? Xullaallisse xa’mi: ilate balaxote yannara mayra ha’ranno amuwu?
4. Amuwu godowinni hee’re qajeelino ogeeyye godowammo qaaqqira ha’runso assi’ra kaa’litanno yite heddanno?
5. **Ilate balaxote ha’runso korkaati fayyimmate uurrinshara.**
6. Godowinni no amuwi ilate balaxote ha’runso horora baqqi assannorichi maatiro xawisi?
7. **Ilate balaxote horora tuncu yitanno mitiimma**
8. Amuwu ilate balaxote ha’runsora fayyimmate uurrinsha ha’ranokkiha ikkiro, korkaatinsa maati?
9. Owaante fayyimmate uurrinshawiinni afi’rannokki gede mitiinsannori hiikkonneeti maati?

**Xullaallisse xa’mi**;

1. Womaashshunna owaante afi’rate baantanni waagi mitiimaati?
2. Fayyimmate uurrinshsha xeertinyina afi’rate mitiima.
3. Dagoomitenna budu mitiimmaati?
4. Owaantete silanchimma hoogate mitiimma?
5. **Owaante afi’nanni hee’nenni darga ganikki mereeroho uurrisate akata/bude korkaata**
6. Amuwu ilate balaxote ha’runso umo ha’runse mereero uurrisannohu mayraati?

**Xullaallisse xa’mi**;

a) Womashshunna owaante afiraate bantanni waggi mitimaati?

1. Fayyimmate urrinshsha xeeritinyina afi’raate mitiima.

c) Dagoomitenna budu mitiimmaati?

d) Owaantete silanchimma hoogate mitiimma?

1. **Godowii hee’nanni yannara budu rosicho.**
2. Budu rosichi maati? Ammanooshshi qarqarikkira godombanni yannara assi’nanirichi maati?
3. Budu ammanooshshi, Ama’note rosichinna balchoomunni assinanniri godombanni yannara, amuwu keeranchimmara qarra abbanno yite hedatta qarqarikkira? Hiittoonni xawisi? Mayra xawisi?
4. Uminsa fajjonni dagoomu owaante uytanno amuwi/budunni ilanno kaa’litanno amuwinna fayyimmate ogeeyyenna amuwu fayyimmate owaante calla uytanno ogeeyye loossanna hiittoonni la’’atta qarqarikkira?
5. **Muli barri giddo calla ilino amuwira**
6. Ama ati godowinni heedhe afi’roota ha’runso owaantete silanchimma hiittoo deerrinni la’’atta? Godowinni hedhe afi’roota owaante may may dannite? Afiroota owantetenni kassi yoota?
7. Ama ilate balaxote ha’runso assidhinoha ikkiro fayyimmate uurrinshira, Xa’mi: ilate balaxote ha’runso owaante assi’rata gede kakkayisiheri maati godowinni heedhenna? Xawisi?
8. Godowinni heedhe balaxote owaante ha’runso assidhinokki korkaati maati?
9. Qarqarikkinni afi’rita amaale: godowinni nootta yannara fayyimmate owaante afi’rate murcokkira hiittenneeti irko afi’ritta? Xawisi?
10. Qajeelino fayyimmate ogeeyyenni godobbe nootta yannara afi’rita fayyimmate owaante gade?

BUUXI:

- 1. Qajeeltino fayyimmate ogeeyyenni godowitta yannara afi’nanni owaantera fayyimmate ogeeyye yiittata sumuu yitinote?
  2. Fayyimmate oggimmansa lifixxinote uytanno owaantera?
  3. Fayyimmate ogeeyye ayirrisannote, shooshaqqinote owaante uytanno woyite?

1. **Qarqaru budu soorreyyenna amma’note massagaanoranna uminsa fajjonni owaante uytanno mannira calla xa’minanni**

**Qarqaru dagoomi hedo fayyimmate ogeeyyenna amuwu fayyimmate owaante lainohunni**

1. Fayyimmate oggeyye qarqari’ne hiittoonni la’anno amuwu fayyimmate owaantera? Qarqari’ne manni hedo amuwu fayyimma owaante aana hittoote? Addi addi hedo fayyimmate ogeeyye daafira nooheta kulie?
2. Lifixxino amuwu fayyimma owaante kawiinni roorenkanni lede woyyaabbinota assate qarqaru teessaano maricho assa hasiissanno?
3. **Ate hedonni, ilate balaxote ha’runsora fayyimmate uurrinshara uynanni owaantera soorrama noose yaattari no?**

**Wo’munku ha’runsaanora illanni yannara**

1. **Illanni yannaranna uurrinshate noo owaante rosicho**
   - - 1. Ilate yannara, amuwaho ikko danqaho uynanni owaante fayyimmate uurrinshanni afi’nanni kaa’lo kaa’litanno yite hedatta?
2. **Ilate yannaranna ha’runso korkaati fayyimmate uurrinshara.**
   - - 1. Ilate yannara amuwa baqqi assannorichi owaante afi’ranno gede assannorichi maatiro xawisi? Xullaallisse xa’mi: korkaatu maati ganyine owaante afi’rate kori maati?
3. **Ilate yannara horora tuncu yitanno mitiimma**
   - - 1. Amuuwu mine illinoha ikkiro, mayira illi mine? korkaatinsa maati? Fayyimmate uurrinshara ilate owaante horo afi’rate gadachaansa korkaati maatiro xawisi?

**Xullaallisse xa’mi**;

1. Womaashshunna owaante afi’rate baantanni waagi mitiimaati?
2. Fayyimmate urrinshsha xeeritinyina afi’raate mitiimma.
3. Dagoomitenna budu mitiimaati?
4. Owaantete silanchimmanna teeda agara hoogate mitiimmaati?
5. **Owaante afi’nanni hee’nenni darga ganikki mereeroho uurrisate akata/bude korkaata**
   - - 1. Amuwu mine ilihu gedensaanni mayra fayyimmate uurrinsha roorenkanni ha’ranno?

**Xullaallisse xa’mi**;

- - - - 1. Womashshunna owaante afiraate bantanni waggi mitimaati?
        2. Fayyimmate uurrinsha xeertinyina afi’rate mitiimma
        3. Dagoomitenna budu mitiimmaati?
        4. Owaantete silanchimmanna teeda agara hoogate mitiimmaati?

1. **Illanni yannara budu rosicho.**
   - - 1. Budu rosichi maati? Ammanooshshi qarqarikkira illanni yannara assi’nanirichi maati?
       2. Budu ammanooshshi, Ama’note rosichinna balchoomunni assinanniri ilate yannara assinanniri amuwu keeranchimmara qarra abbanno yite hedatta qarqarikkira? Hiittoonni xawisi? Mayra xawisi?
       3. Uminsa fajjonni dagoomu owaante uytanno amuwi/budunni ilanno kaa’litanno amuwinna fayyimmate ogeeyyenna amuwu fayyimmate owaante calla uytanno ogeeyye loossanna hiittoonni la’’atta qarqarikkira?
2. **Muli barri giddo calla ilino amuwira**
   - - 1. Ilate yannara, uynohe owaante isilanchimma fayyimmate uurrinshawinni may dani owaante afi’rita? Hitooni xawisaata? Afiroota owaantenni kassi yoota?
       2. Fayyimmate uurrinshshara iloota ikkiro, fayyimmate uurrinshara ilate gede kakkaysiheri korkaatu maati?
       3. Mine iloottaha ikkiro, mitiimmate yite hedattahu fayyimmate uurrinshara maati? Mine ilate korkaati maati fayyimmate urrinshanni agure?
       4. Qarqarikkinni afi’rita amaale: ilate yannara no yannara fayyimmate owaante afi’rate murcokkira hiittenneeti irko afi’ritta? Xawisi?
       5. Qajeelino fayyimmate ogeeyyenni ilate horo afi’rate gadekki xawisittaero? Ilita yannara afi’rata fayyimmate owaante gade?

BUUXI:

- - - - 1. Qajeeltino fayyimmate ogeeyyenni godowitta yannara, ilate yannaranna ilate gedensaanni afi’nanni owaantera fayyimmate ogeeyye yiittata sumuu yitinote?
        2. Fayyimmate oggimmansa lifixxinote uytanno owaantera?
        3. Fayyimmate ogeeyye ayirrisannote, shooshaqqinote owaante uytanno woyite?

1. **Qarqaru budu soorreyyenna amma’note massagaanoranna uminsa fajjonni owaante uytanno mannira calla xa’minanni**

**Qarqaru dagoomi hedo fayyimmate ogeeyyenna amuwu fayyimmate owaante lainohunni**

- - - 1. Fayyimmate oggeyye qarqari’ne hiittoonni la’anno amuwu fayyimmate owaantera? Qarqari’ne manni hedo amuwu fayyimma owaante aana hittoote? Addi addi hedo fayyimmate ogeeyye daafira nooheta kulie?
      2. Lifixxino amuwu fayyimma owaante kawiinni roorenkanni lede woyyaabbinota assate qarqaru teessaano maricho assa hasiissanno?

1. **Ate hedonni, fayyimmate urrinshshara illate yannara sorromma noose yattari no? Ganyine owaante urritukki afi’raate?**

**Wo’munku ha’runsaanora ilate yanna gedensaanni**

1. **Ilate gedensiidi yanna uurrinshate noo owaante rosicho**

Ama ilate gedensaanni fayyimmate owaante afi’rate hiittoonni hadhanno? Tenne yannara/keeshshite mayra hadhannoro xunsite xa’mi?

Me’’e higgine hadhinanni ilate gedensaanni fayyimmate uurrinshshara? Xullaallise xa’mi: tenne yannara mayra ha’ranno amuwu fayyimmate uurrinshsha?

Amuwaahonna danqaaho uyinanni owaante illate gedensanni fayyimmate urrinshshara qajjilinno oggeyyenni kaa’litanno yite hedaata?

1. **Ilate gedensiidi ha’runso korkaati fayyimmate uurrinshara.**

Ilate gedensaanni fayyimmate owaantenni afi’nanni owaantera baqqi assanorichi maatiro xawisi? Xullaallisse xa’mi: ganyine horo afi’rate korkaati maatiro xawisi?

1. **Ilate gedensiidi horora tuncu yitanno mitiimma**

Amuuwu illate gedensanni owaante afi’raate fayyimmate urrinshsha haranokki korkaatinsa maati? Ilate gedensiidikki owaante fayyimmate uurrinshawinni afi’ra hoogate korkaati maati?

**Xullaallisse xa’mi**

1. Womaashshunna owaante afi’rate baantanni waagi mitiimaati?
2. Fayyimmate uurrinsha xeertinyinna afi’rate mitiima.
3. Dagoomitenna budu mitiimaati?
4. Owaantete silanchimma hoogate mitiimma?
5. **Owaante afi’nanni hee’nenni darga ganikki mereeroho uurrisate akata/bude korkaata**

Amuwu roorenkanni fayyimmate uurrinshara ilannohu mayraati? Roorenkanni ilate gedensaanni fayyimmate uurrinshawinni owaante afi’ranokkihu amuwu mayraati? Amuwu qajeeltino ogeeyyenni owaatooshshe afi’rannoha godowinni hee’re hattono ilate yannaranna ilate gedensaanni mitiinsannori maatiro qarqarikkira xawisi?

**Xullaallisse xa’mi**;

1. Womaashshunna owaante afi’rate baantanni waagi mitiimmaati?
2. Fayyimmate uurrinsha xeertinyinna afi’rate mitiimma.
3. Dagoomitenna budu mitiimmaati?
4. Owaantete silanchimma hoogate mitiimma?
5. **Ilate gedensaanni budu rosicho.**

Budu rosichi maati? Ammanooshshi qarqarikkira ilate gedensaanni assi’nanirichi maati?

Budu ammanooshshi, ama’note rosichinna balchoomunni assinanniri ilate gedensaanni assinanniri amuwu keeranchimmara qarra abbanno yite hedatta qarqarikkira? Hiittoonni xawisi? Mayra xawisi?

Uminsa fajjonni dagoomu owaante uytanno amuwi/budunni ilanno kaa’litanno amuwinna fayyimmate ogeeyyenna amuwu fayyimmate owaante calla uytanno ogeeyye loossanna hiittoonni la’’atta qarqarikkira?

1. **Muli barri giddo calla ilino amuwira**

Hiittoonni deerrinni xawisatta? Ilate gedensaanni afi’rootta owaante? May owaante afi’ritta? Afi’rootta owaantenni kassi yootta?

Ilate gedensaanni assi’nanni owaante afi’roottaha ikkiro, korkaatu maati?

Illate gedensanni afi’nanni owaante afidhinokkiha ikkiro,

a) Owaante afi’ra hoogate korkaati maati?

b) Ilate gedensaanni afi’nanni owaante mereereho darga gannikkinni aguranni korkaati maati?

13. Qarqarikkinni afi’rita amaale: ilate gedensaanni no yannara fayyimmate owaante afi’rate murcokkira hiittenneeti irko afi’ritta? Xawisi?

14. Qajeelino fayyimmate ogeeyyenni ilate horo afi’rate gadekki xawisittaero? Ilate gedesiidikki fayyimmate owaante gade?

BUUXI:

1. Qajeeltino fayyimmate ogeeyyenni godowitta yannara, ilate yannaranna ilate gedensaanni afi’nanni owaantera fayyimmate ogeeyye yiittata sumuu yitinote?
2. Fayyimmate oggimmansa lifixxinote uytanno owaantera?
3. Fayyimmate ogeeyye ayirrisannote, shooshaqqinote owaante uytanno woyite?
4. **Qarqaru budu soorreyyenna amma’note massagaanoranna uminsa fajjonni owaante uytanno mannira calla xa’minanni**

**Qarqaru dagoomi hedo fayyimmate ogeeyyenna amuwu fayyimmate owaante lainohunni**

- - - 1. Fayyimmate oggeyye qarqari’ne hiittoonni la’anno amuwu fayyimmate owaantera? Qarqari’ne manni hedo amuwu fayyimma owaante aana hittoote? Addi addi hedo fayyimmate ogeeyye daafira nooheta kulie?
      2. Lifixxino amuwu fayyimma owaante kawiinni roorenkanni lede woyyaabbinota assate qarqaru teessaano maricho assa hasiissanno?

1. **Ate hedonni, marichi sorooma hassissanossi illate gedensanni no yannara? Ganyine owaante urritukki afi’raate?**

**Ha’runsokkira galaxxeemmo!**

**2. Illachishshu Gaamo Hasaawa Massago (FGD) guide (Sidaamu Afii Gano)**

| **I** | **Gafa I: Ayimmate Xawishsha** | |
| --- | --- | --- |
| 1 | Borri-xa’mote Badooshshi Kiiro | **____________________________** |
| 2 | Woradu Su’ma | **____________________________** |
| 3 | Olluu su’ma | **____________________________________** |
| 4 | Mereerima Su’ma (moderate) | **_______________________________** |
| 5 | Qaagiishsha amadannohu/te su’ma | **_________________________________** |
| 6 | Hasaawa barra | **_______________________________** |
| 7 | Hanafote yanna: | **______:________** |
| 8 | Gumulshu yanna: | **____:______** |

| **II** | **Gafa II: Beeqqisiisaancho Mannoomitte Taje** | | | | | |
| --- | --- | --- | --- | --- | --- | --- |
| 1 | Beeqqo assinoonni koodde |  |  |  |  |  |
| 2 | Diro |  |  |  |  |  |
| 3 | Amma’nokki |  |  |  |  |  |
| 4 | Adhamate ikkito |  |  |  |  |  |
| 5 | Mootimmate woy hallanyaho loosata/to? (Ee/Dee’ni) |  |  |  |  |  |
| 6 | Rosu deerra |  |  |  |  |  |
| **MULE ILTINO AMARA CALLA** | | | | | | |
| 7 | Me’’e higge godowootta |  |  |  |  |  |
| 8 | Meu qaaqquulli noohe |  |  |  |  |  |
| 9 | Konne gedensiidikki qaaqqo hiikko ilitta? Mine/fayyimmate uurrinshara? |  |  |  |  |  |

**Qaali-xa’mote Massago**

**Godombbanni yannara wo’munku ha’runsaanora**

1. **Godombanni yanna uurrinshate noo owaante rosicho**

Godowinni no ama ilate balaxote ha’runso hiittoonni hadhe afidhanno fayyimmate owaante? Seekkite xa’mitinanni yannara mayira balaxxe woy gedensaanni hadhanno fayyimmate uurrishsha?

Ilate balaxote ha’runso me’’e hige hadhanno? Xullaallisse xa’mi: ilate balaxote yannara mayra ha’ranno amuwu?

Amuwu godowinni hee’re qajeelino ogeeyye godowammo qaaqqira ha’runso assi’ra kaa’litanno yite heddanno?

1. **Ilate balaxote ha’runso korkaati fayyimmate uurrinshara.**

Godowinni no amuwi ilate balaxote ha’runso horora baqqi assannorichi maatiro xawisi?

1. **Ilate balaxote horora tuncu yitanno mitiimma**

Amuwu ilate balaxote ha’runsora fayyimmate uurrinsha ha’ranokkiha ikkiro, korkaatinsa maati?

Owaante fayyimmate uurrinshawiinni afi’rannokki gede mitiinsannori hiikkonneeti maati?

**Xullaallisse xa’mi**;

Womaashshunna owaante afi’rate baantanni waagi mitiimaati?

Fayyimmate uurrinshsha xeertinyina afi’rate mitiima.

Dagoomitenna budu mitiimmaati?

Owaantete silanchimma hoogate mitiimma?

1. **Owaante afi’nanni hee’nenni darga ganikki mereeroho uurrisate akata/bude korkaata**

Amuwu ilate balaxote ha’runso umo ha’runse mereero uurrisannohu mayraati?

**Xullaallisse xa’mi**;

Womashshunna owaante afiraate bantanni waggi mitimaati?

Fayyimmate urrinshsha xeeritinyina afi’raate mitiima.

Dagoomitenna budu mitiimmaati?

Owaantete silanchimma hoogate mitiimma?

1. **Godowii hee’nanni yannara budu rosicho.**

Budu rosichi maati? Ammanooshshi qarqarikkira godombanni yannara assi’nanirichi maati?

Budu ammanooshshi, Ama’note rosichinna balchoomunni assinanniri godombanni yannara, amuwu keeranchimmara qarra abbanno yite hedatta qarqarikkira? Hiittoonni xawisi? Mayra xawisi?

Uminsa fajjonni dagoomu owaante uytanno amuwi/budunni ilanno kaa’litanno amuwinna fayyimmate ogeeyyenna amuwu fayyimmate owaante calla uytanno ogeeyye loossanna hiittoonni la’’atta qarqarikkira?

1. **Muli barri giddo calla ilino amuwira**

Ama ati godowinni heedhe afi’roota ha’runso owaantete silanchimma hiittoo deerrinni la’’atta? Godowinni hedhe afi’roota owaante may may dannite? Afiroota owantetenni kassi yoota?

Ama ilate balaxote ha’runso assidhinoha ikkiro fayyimmate uurrinshira, Xa’mi: ilate balaxote ha’runso owaante assi’rata gede kakkayisiheri maati godowinni heedhenna? Xawisi?

Godowinni heedhe balaxote owaante ha’runso assidhinokki korkaati maati?

Qarqarikkinni afi’rita amaale: godowinni nootta yannara fayyimmate owaante afi’rate murcokkira hiittenneeti irko afi’ritta? Xawisi?

Qajeelino fayyimmate ogeeyyenni godobbe nootta yannara afi’rita fayyimmate owaante gade?

BUUXI:

A. Qajeeltino fayyimmate ogeeyyenni godowitta yannara afi’nanni owaantera fayyimmate ogeeyye yiittata sumuu yitinote?

B. Fayyimmate oggimmansa lifixxinote uytanno owaantera?

C. Fayyimmate ogeeyye ayirrisannote, shooshaqqinote owaante uytanno woyite?

1. **Qarqaru budu soorreyyenna amma’note massagaanoranna uminsa fajjonni owaante uytanno mannira calla xa’minanni**

**Qarqaru dagoomi hedo fayyimmate ogeeyyenna amuwu fayyimmate owaante lainohunni**

Fayyimmate oggeyye qarqari’ne hiittoonni la’anno amuwu fayyimmate owaantera? Qarqari’ne manni hedo amuwu fayyimma owaante aana hittoote? Addi addi hedo fayyimmate ogeeyye daafira nooheta kulie?

- - - 1. Lifixxino amuwu fayyimma owaante kawiinni roorenkanni lede woyyaabbinota assate qarqaru teessaano maricho assa hasiissanno?

1. **Ate hedonni, ilate balaxote ha’runsora fayyimmate uurrinshara uynanni owaantera soorrama noose yaattari no?**

**Wo’munku ha’runsaanora illanni yannara**

1. **Illanni yannaranna uurrinshate noo owaante rosicho**

Ilate yannara, amuwaho ikko danqaho uynanni owaante fayyimmate uurrinshanni afi’nanni kaa’lo kaa’litanno yite hedatta?

1. **Ilate yannaranna ha’runso korkaati fayyimmate uurrinshara.**

Ilate yannara amuwa baqqi assannorichi owaante afi’ranno gede assannorichi maatiro xawisi? Xullaallisse xa’mi: korkaatu maati ganyine owaante afi’rate kori maati?

1. **Ilate yannara horora tuncu yitanno mitiimma**

Amuuwu mine illinoha ikkiro, mayira illi mine? korkaatinsa maati? Fayyimmate uurrinshara ilate owaante horo afi’rate gadachaansa korkaati maatiro xawisi?

**Xullaallisse xa’mi**;

Womaashshunna owaante afi’rate baantanni waagi mitiimaati?

Fayyimmate urrinshsha xeeritinyina afi’raate mitiimma.

Dagoomitenna budu mitiimaati?

Owaantete silanchimmanna teeda agara hoogate mitiimmaati?

1. **Owaante afi’nanni hee’nenni darga ganikki mereeroho uurrisate akata/bude korkaata**

Amuwu mine ilihu gedensaanni mayra fayyimmate uurrinsha roorenkanni ha’ranno?

**Xullaallisse xa’mi**;

a. Womashshunna owaante afiraate bantanni waggi mitimaati?

- - - - 1. Fayyimmate uurrinsha xeertinyina afi’rate mitiimma
        2. Dagoomitenna budu mitiimmaati?
        3. Owaantete silanchimmanna teeda agara hoogate mitiimmaati?

1. **Illanni yannara budu rosicho.**
2. Budu rosichi maati? Ammanooshshi qarqarikkira illanni yannara assi’nanirichi maati?
3. Budu ammanooshshi, Ama’note rosichinna balchoomunni assinanniri ilate yannara assinanniri amuwu keeranchimmara qarra abbanno yite hedatta qarqarikkira? Hiittoonni xawisi? Mayra xawisi?
4. Uminsa fajjonni dagoomu owaante uytanno amuwi/budunni ilanno kaa’litanno amuwinna fayyimmate ogeeyyenna amuwu fayyimmate owaante calla uytanno ogeeyye loossanna hiittoonni la’’atta qarqarikkira?
5. **Muli barri giddo calla ilino amuwira**
6. Ilate yannara, uynohe owaante isilanchimma fayyimmate uurrinshawinni may dani owaante afi’rita? Hitooni xawisaata? Afiroota owaantenni kassi yoota?
7. Fayyimmate uurrinshshara iloota ikkiro, fayyimmate uurrinshara ilate gede kakkaysiheri korkaatu maati?
8. Mine iloottaha ikkiro, mitiimmate yite hedattahu fayyimmate uurrinshara maati? Mine ilate korkaati maati fayyimmate urrinshanni agure?
9. Qarqarikkinni afi’rita amaale: ilate yannara no yannara fayyimmate owaante afi’rate murcokkira hiittenneeti irko afi’ritta? Xawisi?
10. Qajeelino fayyimmate ogeeyyenni ilate horo afi’rate gadekki xawisittaero? Ilita yannara afi’rata fayyimmate owaante gade?

BUUXI:

- - - - 1. Qajeeltino fayyimmate ogeeyyenni godowitta yannara, ilate yannaranna ilate gedensaanni afi’nanni owaantera fayyimmate ogeeyye yiittata sumuu yitinote?
        2. Fayyimmate oggimmansa lifixxinote uytanno owaantera?
        3. Fayyimmate ogeeyye ayirrisannote, shooshaqqinote owaante uytanno woyite?

1. **Qarqaru budu soorreyyenna amma’note massagaanoranna uminsa fajjonni owaante uytanno mannira calla xa’minanni**

**Qarqaru dagoomi hedo fayyimmate ogeeyyenna amuwu fayyimmate owaante lainohunni**

1. Fayyimmate oggeyye qarqari’ne hiittoonni la’anno amuwu fayyimmate owaantera? Qarqari’ne manni hedo amuwu fayyimma owaante aana hittoote? Addi addi hedo fayyimmate ogeeyye daafira nooheta kulie?
2. Lifixxino amuwu fayyimma owaante kawiinni roorenkanni lede woyyaabbinota assate qarqaru teessaano maricho assa hasiissanno?
3. **Ate hedonni, fayyimmate urrinshshara illate yannara sorromma noose yattari no? Ganyine owaante urritukki afi’raate?**

**Wo’munku ha’runsaanora ilate yanna gedensaanni**

1. **Ilate gedensiidi yanna uurrinshate noo owaante rosicho**

Ama ilate gedensaanni fayyimmate owaante afi’rate hiittoonni hadhanno? Tenne yannara/keeshshite mayra hadhannoro xunsite xa’mi?

Me’’e higgine hadhinanni ilate gedensaanni fayyimmate uurrinshshara? Xullaallise xa’mi: tenne yannara mayra ha’ranno amuwu fayyimmate uurrinshsha?

Amuwaahonna danqaaho uyinanni owaante illate gedensanni fayyimmate urrinshshara qajjilinno oggeyyenni kaa’litanno yite hedaata?

1. **Ilate gedensiidi ha’runso korkaati fayyimmate uurrinshara.**

Ilate gedensaanni fayyimmate owaantenni afi’nanni owaantera baqqi assanorichi maatiro xawisi? Xullaallisse xa’mi: ganyine horo afi’rate korkaati maatiro xawisi?

1. **Ilate gedensiidi horora tuncu yitanno mitiimma**

Amuuwu illate gedensanni owaante afi’raate fayyimmate urrinshsha haranokki korkaatinsa maati? Ilate gedensiidikki owaante fayyimmate uurrinshawinni afi’ra hoogate korkaati maati?

**Xullaallisse xa’mi**

Womaashshunna owaante afi’rate baantanni waagi mitiimaati?

Fayyimmate uurrinsha xeertinyinna afi’rate mitiima.

Dagoomitenna budu mitiimaati?

Owaantete silanchimma hoogate mitiimma?

1. **Owaante afi’nanni hee’nenni darga ganikki mereeroho uurrisate akata/bude korkaata**

Amuwu roorenkanni fayyimmate uurrinshara ilannohu mayraati? Roorenkanni ilate gedensaanni fayyimmate uurrinshawinni owaante afi’ranokkihu amuwu mayraati? Amuwu qajeeltino ogeeyyenni owaatooshshe afi’rannoha godowinni hee’re hattono ilate yannaranna ilate gedensaanni mitiinsannori maatiro qarqarikkira xawisi?

**Xullaallisse xa’mi**;

- 1. Womaashshunna owaante afi’rate baantanni waagi mitiimmaati?
  2. Fayyimmate uurrinsha xeertinyinna afi’rate mitiimma.
  3. Dagoomitenna budu mitiimmaati?
  4. Owaantete silanchimma hoogate mitiimma?

1. **Ilate gedensaanni budu rosicho.**

Budu rosichi maati? Ammanooshshi qarqarikkira ilate gedensaanni assi’nanirichi maati?

Budu ammanooshshi, ama’note rosichinna balchoomunni assinanniri ilate gedensaanni assinanniri amuwu keeranchimmara qarra abbanno yite hedatta qarqarikkira? Hiittoonni xawisi? Mayra xawisi?

Uminsa fajjonni dagoomu owaante uytanno amuwi/budunni ilanno kaa’litanno amuwinna fayyimmate ogeeyyenna amuwu fayyimmate owaante calla uytanno ogeeyye loossanna hiittoonni la’’atta qarqarikkira?

1. **Muli barri giddo calla ilino amuwira**

Hiittoonni deerrinni xawisatta? Ilate gedensaanni afi’rootta owaante? May owaante afi’ritta? Afi’rootta owaantenni kassi yootta?

Ilate gedensaanni assi’nanni owaante afi’roottaha ikkiro, korkaatu maati?

Illate gedensanni afi’nanni owaante afidhinokkiha ikkiro,

a) Owaante afi’ra hoogate korkaati maati?

b) Ilate gedensaanni afi’nanni owaante mereereho darga gannikkinni aguranni korkaati maati?

13. Qarqarikkinni afi’rita amaale: ilate gedensaanni no yannara fayyimmate owaante afi’rate murcokkira hiittenneeti irko afi’ritta? Xawisi?

14. Qajeelino fayyimmate ogeeyyenni ilate horo afi’rate gadekki xawisittaero? Ilate gedesiidikki fayyimmate owaante gade?

BUUXI:

a. Qajeeltino fayyimmate ogeeyyenni godowitta yannara, ilate yannaranna ilate gedensaanni afi’nanni owaantera fayyimmate ogeeyye yiittata sumuu yitinote?

b. Fayyimmate oggimmansa lifixxinote uytanno owaantera?

c. Fayyimmate ogeeyye ayirrisannote, shooshaqqinote owaante uytanno woyite?

1. **Qarqaru budu soorreyyenna amma’note massagaanoranna uminsa fajjonni owaante uytanno mannira calla xa’minanni**

**Qarqaru dagoomi hedo fayyimmate ogeeyyenna amuwu fayyimmate owaante lainohunni**

1. Fayyimmate oggeyye qarqari’ne hiittoonni la’anno amuwu fayyimmate owaantera? Qarqari’ne manni hedo amuwu fayyimma owaante aana hittoote? Addi addi hedo fayyimmate ogeeyye daafira nooheta kulie?
2. Lifixxino amuwu fayyimma owaante kawiinni roorenkanni lede woyyaabbinota assate qarqaru teessaano maricho assa hasiissanno?
3. **Ate hedonni, marichi sorooma hassissanossi illate gedensanni no yannara? Ganyine owaante urritukki afi’raate?**

**Ha’runsokkira galaxxeemmo!**

**3 Qara mashalaqqisannora shiqqino qaalli-xa’mo**

| **I** | **Gafa I: Ayimmate Xawishsha** | |
| --- | --- | --- |
| 1 | Borri-xa’mote Badooshshi Kiiro | **____________________________** |
| 2 | Woradu Su’ma | **____________________________** |
| 3 | Olluu su’ma | **____________________________________** |
| 4 | Mereerima Su’ma (moderate) | **_______________________________** |
| 5 | Qaagiishsha amadannohu/te su’ma | **_________________________________** |
| 6 | Hasaawa barra | **_______________________________** |
| 7 | Hanafote yanna: | **______:________** |
| 8 | Gumulshu yanna: | **____:______** |

| **II** | **Gafa II: Beeqqisiisaancho Mannoomitte Taje** | | | | | |
| --- | --- | --- | --- | --- | --- | --- |
| 1 | Beeqqo assinoonni koodde |  |  |  |  |  |
| 2 | Diro |  |  |  |  |  |
| 3 | Amma’nokki |  |  |  |  |  |
| 4 | Adhamate ikkito |  |  |  |  |  |
| 5 | Mootimmate woy hallanyaho loosata/to? (Ee/Dee’ni) |  |  |  |  |  |
| 6 | Rosu deerra |  |  |  |  |  |
| **MULE ILTINO AMARA CALLA** | | | | | | |
| 7 | Me’’e higge godowootta |  |  |  |  |  |
| 8 | Meu qaaqquulli noohe |  |  |  |  |  |
| 9 | Konne gedensiidikki qaaqqo hiikko ilitta? Mine/fayyimmate uurrinshara? |  |  |  |  |  |

**Qaali-xa’mote Massago**

**Godombbanni yannara wo’munku ha’runsaanora**

1. **Godombanni yanna uurrinshate noo owaante rosicho**
   - - 1. Godowinni no ama ilate balaxote ha’runso hiittoonni hadhe afidhanno fayyimmate owaante? Seekkite xa’mitinanni yannara mayira balaxxe woy gedensaanni hadhanno fayyimmate uurrishsha?
       2. Ilate balaxote ha’runso me’’e hige hadhanno? Xullaallisse xa’mi: ilate balaxote yannara mayra ha’ranno amuwu?
       3. Amuwu godowinni hee’re qajeelino ogeeyye godowammo qaaqqira ha’runso assi’ra kaa’litanno yite heddanno?
2. **Ilate balaxote ha’runso korkaati fayyimmate uurrinshara.**
   - - 1. Godowinni no amuwi ilate balaxote ha’runso horora baqqi assannorichi maatiro xawisi?
3. **Ilate balaxote horora tuncu yitanno mitiimma**
   - - 1. Amuwu ilate balaxote ha’runsora fayyimmate uurrinsha ha’ranokkiha ikkiro, korkaatinsa maati?
       2. Owaante fayyimmate uurrinshawiinni afi’rannokki gede mitiinsannori hiikkonneeti maati?

**Xullaallisse xa’mi**;

a. Womaashshunna owaante afi’rate baantanni waagi mitiimaati?

- 1. Fayyimmate uurrinshsha xeertinyina afi’rate mitiima.
  2. Dagoomitenna budu mitiimmaati?
  3. Owaantete silanchimma hoogate mitiimma?

1. **Owaante afi’nanni hee’nenni darga ganikki mereeroho uurrisate akata/bude korkaata**
2. Amuwu ilate balaxote ha’runso umo ha’runse mereero uurrisannohu mayraati?

**Xullaallisse xa’mi**;

a) Womashshunna owaante afiraate bantanni waggi mitimaati?

- - 1. Fayyimmate urrinshsha xeeritinyina afi’raate mitiima.

c) Dagoomitenna budu mitiimmaati?

d) Owaantete silanchimma hoogate mitiimma?

1. **Godowii hee’nanni yannara budu rosicho.**
2. Budu rosichi maati? Ammanooshshi qarqarikkira godombanni yannara assi’nanirichi maati?
3. Budu ammanooshshi, Ama’note rosichinna balchoomunni assinanniri godombanni yannara, amuwu keeranchimmara qarra abbanno yite hedatta qarqarikkira? Hiittoonni xawisi? Mayra xawisi?
4. Uminsa fajjonni dagoomu owaante uytanno amuwi/budunni ilanno kaa’litanno amuwinna fayyimmate ogeeyyenna amuwu fayyimmate owaante calla uytanno ogeeyye loossanna hiittoonni la’’atta qarqarikkira?
5. **Muli barri giddo calla ilino amuwira**
6. Ama ati godowinni heedhe afi’roota ha’runso owaantete silanchimma hiittoo deerrinni la’’atta? Godowinni hedhe afi’roota owaante may may dannite? Afiroota owantetenni kassi yoota?
7. Ama ilate balaxote ha’runso assidhinoha ikkiro fayyimmate uurrinshira, Xa’mi: ilate balaxote ha’runso owaante assi’rata gede kakkayisiheri maati godowinni heedhenna? Xawisi?
8. Godowinni heedhe balaxote owaante ha’runso assidhinokki korkaati maati?
9. Qarqarikkinni afi’rita amaale: godowinni nootta yannara fayyimmate owaante afi’rate murcokkira hiittenneeti irko afi’ritta? Xawisi?
10. Qajeelino fayyimmate ogeeyyenni godobbe nootta yannara afi’rita fayyimmate owaante gade?

BUUXI:

- 1. Qajeeltino fayyimmate ogeeyyenni godowitta yannara afi’nanni owaantera fayyimmate ogeeyye yiittata sumuu yitinote?
  2. Fayyimmate oggimmansa lifixxinote uytanno owaantera?
  3. Fayyimmate ogeeyye ayirrisannote, shooshaqqinote owaante uytanno woyite?

1. **Qarqaru budu soorreyyenna amma’note massagaanoranna uminsa fajjonni owaante uytanno mannira calla xa’minanni**

**Qarqaru dagoomi hedo fayyimmate ogeeyyenna amuwu fayyimmate owaante lainohunni**

1. Fayyimmate oggeyye qarqari’ne hiittoonni la’anno amuwu fayyimmate owaantera? Qarqari’ne manni hedo amuwu fayyimma owaante aana hittoote? Addi addi hedo fayyimmate ogeeyye daafira nooheta kulie?
2. Lifixxino amuwu fayyimma owaante kawiinni roorenkanni lede woyyaabbinota assate qarqaru teessaano maricho assa hasiissanno?
3. **Ate hedonni, ilate balaxote ha’runsora fayyimmate uurrinshara uynanni owaantera soorrama noose yaattari no?**

**Wo’munku ha’runsaanora illanni yannara**

1. **Illanni yannaranna uurrinshate noo owaante rosicho**
   - - 1. Ilate yannara, amuwaho ikko danqaho uynanni owaante fayyimmate uurrinshanni afi’nanni kaa’lo kaa’litanno yite hedatta?
2. **Ilate yannaranna ha’runso korkaati fayyimmate uurrinshara.**
   - - 1. Ilate yannara amuwa baqqi assannorichi owaante afi’ranno gede assannorichi maatiro xawisi? Xullaallisse xa’mi: korkaatu maati ganyine owaante afi’rate kori maati?
3. **Ilate yannara horora tuncu yitanno mitiimma**
   - - 1. Amuuwu mine illinoha ikkiro, mayira illi mine? korkaatinsa maati? Fayyimmate uurrinshara ilate owaante horo afi’rate gadachaansa korkaati maatiro xawisi?

**Xullaallisse xa’mi**;

a.Womaashshunna owaante afi’rate baantanni waagi mitiimaati?

- 1. Fayyimmate urrinshsha xeeritinyina afi’raate mitiimma.
  2. Dagoomitenna budu mitiimaati?
  3. Owaantete silanchimmanna teeda agara hoogate mitiimmaati?

1. **Owaante afi’nanni hee’nenni darga ganikki mereeroho uurrisate akata/bude korkaata**
2. Amuwu mine ilihu gedensaanni mayra fayyimmate uurrinsha roorenkanni ha’ranno?

**Xullaallisse xa’mi**;

- - - - 1. Womashshunna owaante afiraate bantanni waggi mitimaati?
        2. Fayyimmate uurrinsha xeertinyina afi’rate mitiimma
        3. Dagoomitenna budu mitiimmaati?
        4. Owaantete silanchimmanna teeda agara hoogate mitiimmaati?

1. **Illanni yannara budu rosicho.**
2. Budu rosichi maati? Ammanooshshi qarqarikkira illanni yannara assi’nanirichi maati?
3. Budu ammanooshshi, Ama’note rosichinna balchoomunni assinanniri ilate yannara assinanniri amuwu keeranchimmara qarra abbanno yite hedatta qarqarikkira? Hiittoonni xawisi? Mayra xawisi?
4. Uminsa fajjonni dagoomu owaante uytanno amuwi/budunni ilanno kaa’litanno amuwinna fayyimmate ogeeyyenna amuwu fayyimmate owaante calla uytanno ogeeyye loossanna hiittoonni la’’atta qarqarikkira?
5. **Muli barri giddo calla ilino amuwira**
6. Ilate yannara, uynohe owaante isilanchimma fayyimmate uurrinshawinni may dani owaante afi’rita? Hitooni xawisaata? Afiroota owaantenni kassi yoota?
7. Fayyimmate uurrinshshara iloota ikkiro, fayyimmate uurrinshara ilate gede kakkaysiheri korkaatu maati?
8. Mine iloottaha ikkiro, mitiimmate yite hedattahu fayyimmate uurrinshara maati? Mine ilate korkaati maati fayyimmate urrinshanni agure?
9. Qarqarikkinni afi’rita amaale: ilate yannara no yannara fayyimmate owaante afi’rate murcokkira hiittenneeti irko afi’ritta? Xawisi?
10. Qajeelino fayyimmate ogeeyyenni ilate horo afi’rate gadekki xawisittaero? Ilita yannara afi’rata fayyimmate owaante gade?

BUUXI:

- - - - 1. Qajeeltino fayyimmate ogeeyyenni godowitta yannara, ilate yannaranna ilate gedensaanni afi’nanni owaantera fayyimmate ogeeyye yiittata sumuu yitinote?
        2. Fayyimmate oggimmansa lifixxinote uytanno owaantera?
        3. Fayyimmate ogeeyye ayirrisannote, shooshaqqinote owaante uytanno woyite?

1. **Qarqaru budu soorreyyenna amma’note massagaanoranna uminsa fajjonni owaante uytanno mannira calla xa’minanni**

**Qarqaru dagoomi hedo fayyimmate ogeeyyenna amuwu fayyimmate owaante lainohunni**

1. Fayyimmate oggeyye qarqari’ne hiittoonni la’anno amuwu fayyimmate owaantera? Qarqari’ne manni hedo amuwu fayyimma owaante aana hittoote? Addi addi hedo fayyimmate ogeeyye daafira nooheta kulie?
2. Lifixxino amuwu fayyimma owaante kawiinni roorenkanni lede woyyaabbinota assate qarqaru teessaano maricho assa hasiissanno?
3. **Ate hedonni, fayyimmate urrinshshara illate yannara sorromma noose yattari no? Ganyine owaante urritukki afi’raate?**

**Wo’munku ha’runsaanora ilate yanna gedensaanni**

- - 1. **Ilate gedensiidi yanna uurrinshate noo owaante rosicho**

Ama ilate gedensaanni fayyimmate owaante afi’rate hiittoonni hadhanno? Tenne yannara/keeshshite mayra hadhannoro xunsite xa’mi?

Me’’e higgine hadhinanni ilate gedensaanni fayyimmate uurrinshshara? Xullaallise xa’mi: tenne yannara mayra ha’ranno amuwu fayyimmate uurrinshsha?

Amuwaahonna danqaaho uyinanni owaante illate gedensanni fayyimmate urrinshshara qajjilinno oggeyyenni kaa’litanno yite hedaata?

- - 1. **Ilate gedensiidi ha’runso korkaati fayyimmate uurrinshara.**

Ilate gedensaanni fayyimmate owaantenni afi’nanni owaantera baqqi assanorichi maatiro xawisi? Xullaallisse xa’mi: ganyine horo afi’rate korkaati maatiro xawisi?

- - 1. **Ilate gedensiidi horora tuncu yitanno mitiimma**

Amuuwu illate gedensanni owaante afi’raate fayyimmate urrinshsha haranokki korkaatinsa maati? Ilate gedensiidikki owaante fayyimmate uurrinshawinni afi’ra hoogate korkaati maati?

**Xullaallisse xa’mi**

a. Womaashshunna owaante afi’rate baantanni waagi mitiimaati?

b. Fayyimmate uurrinsha xeertinyinna afi’rate mitiima.

c. Dagoomitenna budu mitiimaati?

d. Owaantete silanchimma hoogate mitiimma?

- - 1. **Owaante afi’nanni hee’nenni darga ganikki mereeroho uurrisate akata/bude korkaata**

Amuwu roorenkanni fayyimmate uurrinshara ilannohu mayraati? Roorenkanni ilate gedensaanni fayyimmate uurrinshawinni owaante afi’ranokkihu amuwu mayraati? Amuwu qajeeltino ogeeyyenni owaatooshshe afi’rannoha godowinni hee’re hattono ilate yannaranna ilate gedensaanni mitiinsannori maatiro qarqarikkira xawisi?

**Xullaallisse xa’mi**;

a.Womaashshunna owaante afi’rate baantanni waagi mitiimmaati?

b. Fayyimmate uurrinsha xeertinyinna afi’rate mitiimma.

c. Dagoomitenna budu mitiimmaati?

d. Owaantete silanchimma hoogate mitiimma?

- - 1. **Ilate gedensaanni budu rosicho.**

Budu rosichi maati? Ammanooshshi qarqarikkira ilate gedensaanni assi’nanirichi maati?

Budu ammanooshshi, ama’note rosichinna balchoomunni assinanniri ilate gedensaanni assinanniri amuwu keeranchimmara qarra abbanno yite hedatta qarqarikkira? Hiittoonni xawisi? Mayra xawisi?

Uminsa fajjonni dagoomu owaante uytanno amuwi/budunni ilanno kaa’litanno amuwinna fayyimmate ogeeyyenna amuwu fayyimmate owaante calla uytanno ogeeyye loossanna hiittoonni la’’atta qarqarikkira?

- - 1. **Muli barri giddo calla ilino amuwira**

Hiittoonni deerrinni xawisatta? Ilate gedensaanni afi’rootta owaante? May owaante afi’ritta? Afi’rootta owaantenni kassi yootta?

Ilate gedensaanni assi’nanni owaante afi’roottaha ikkiro, korkaatu maati?

Illate gedensanni afi’nanni owaante afidhinokkiha ikkiro,

a) Owaante afi’ra hoogate korkaati maati?

b) Ilate gedensaanni afi’nanni owaante mereereho darga gannikkinni aguranni korkaati maati?

13. Qarqarikkinni afi’rita amaale: ilate gedensaanni no yannara fayyimmate owaante afi’rate murcokkira hiittenneeti irko afi’ritta? Xawisi?

14. Qajeelino fayyimmate ogeeyyenni ilate horo afi’rate gadekki xawisittaero? Ilate gedesiidikki fayyimmate owaante gade?

BUUXI:

a.Qajeeltino fayyimmate ogeeyyenni godowitta yannara, ilate yannaranna ilate gedensaanni afi’nanni owaantera fayyimmate ogeeyye yiittata sumuu yitinote?

b. Fayyimmate oggimmansa lifixxinote uytanno owaantera?

c. Fayyimmate ogeeyye ayirrisannote, shooshaqqinote owaante uytanno woyite?

- - 1. **Qarqaru budu soorreyyenna amma’note massagaanoranna uminsa fajjonni owaante uytanno mannira calla xa’minanni**

**Qarqaru dagoomi hedo fayyimmate ogeeyyenna amuwu fayyimmate owaante lainohunni**

1. Fayyimmate oggeyye qarqari’ne hiittoonni la’anno amuwu fayyimmate owaantera? Qarqari’ne manni hedo amuwu fayyimma owaante aana hittoote? Addi addi hedo fayyimmate ogeeyye daafira nooheta kulie?
2. Lifixxino amuwu fayyimma owaante kawiinni roorenkanni lede woyyaabbinota assate qarqaru teessaano maricho assa hasiissanno?
   - 1. **Ate hedonni, marichi sorooma hassissanossi illate gedensanni no yannara? Ganyine owaante urritukki afi’raate?**

**Ha’runsokkira galaxxeemmo!**
