# Supplementary material for: Perceptions, barriers, and facilitators of maternal health service utilization in southern Ethiopia: A qualitative exploration of community members’ and health care providers’ views
Source: PLoS One. 2024 Dec 19;19(12):e0312484. doi: 10.1371/journal.pone.0312484 (PMC11658624; doi:10.1371/journal.pone.0312484)
Supplement: S3 File — (DOCX) [file pone.0312484.s003.docx]

**FGD: 01**

**Antepartum for all participants**

1. **The practice of ANC**

**Moderator:** How early do women go for ANC?

**D3:** at the fourth month of pregnancy. First, she will give a urine sample and confirm her pregnancy. After confirming her pregnancy, she will start ANC visits in the sixth month until the date of delivery. Then, she will give birth at the health center.

**D1:** she will go to the health center and confirm her pregnancy when she has symptoms of pregnancy. She will start her follow-up in the fourth month. She will take an appointment for the next visit during the time of her confirmation test.

**D9:** after she confirmed her pregnancy by urine sample she will go back to the health facility based on her appointment time. She will take two vaccinations during her follow-up. She will check herself and the fetus's health one or two times besides the first visit. She will go to the HC when she has symptoms of labor in the ninth month. She can also go meanwhile when she feels any abnormal symptoms.

**D2:** she will start the follow-up after she gives a urine sample and confirm her pregnancy.

**D5:** after the confirmation of the pregnancy, she will provide with a drug to prevent anemia. She may have gastritis and the health care provider (HCP) will help her too.

**D7:** after they confirmed pregnancy they will check the position of the baby. They will advise her immediately to go to the health institution after her labor start.

**D3:** if our case is beyond their capacity they will refer us to another hospital. They will provide us with an ambulance and will refer us. They will refer before any problem that occurs to the mother and fetus. They will also advise us to bring our pregnant friends who are not yet started follow-up.

**Moderator:** Explain factors that would motivate women to utilize ANC services during their pregnancy.

**D7:** when a pregnant woman has under-nutrition they will advise her to take more food.

**D3:** we will visit the health center to identify and solve our problems. We cannot check our problems if we stay at home. They have better knowledge as compared to us this is why we visit the Health Center (HC). They will also refer to the next level if our problem is beyond their capacity.

**D1:** they are skilled professionals so we have to go to them, to check for our health status. Health extension workers (HEW) give us health education; they will always tell us to come and check however, few will not accept their advice and try to deliver at home and may die. After we take health education besides ourselves use, we also educate others especially those with older age mothers who refuse and failed to accept the education. We will provide health education during home-to-home visits.

**D8:** mothers will fear visiting HC after the pregnancy; we will educate and encourage them to start the follow-up. When mothers fear, we will encourage them to start the follow-up; even we will follow their condition regularly.

**D2:** previously mother will not go to the health institution for delivery due to this the mother and fetus will face problems. After we receive the health education, they will usually go to the HC for the service. When she delivers at home, blood will retain in her uterus and will have abdominal cramps, and also the newborn will also face a problem. However, if she delivers at the HC, the mother, and the newborn will not face any problems since the place is clean.

**D9:** due to the health problem with the conception she will visit the health institution. She will also visit the HC if she has unusual feelings. She will be checked, and the baby's position also checked for anemia and blood pressure. She will confirm the above problems when she visits the HC, if there is any problem she will take medication. They will refer her if the problem is above their capability.

**Moderator:** if women do not go for ANC, what are their reasons?

**D1:** yes, previously some mothers don't go for ANC follow-up, currently the HEWs in the kebele know the number of pregnant mothers and which mother is pregnant. So, they will provide health education to each mother to start ANC follow-up. Currently, all pregnant mothers have follow-ups until they deliver, no mother will deliver without follow-up.

**D9:** nothing left from the HEWs they will hold a pregnant mothers forum every 28 days. They will also inform and teach us to inform and bring our neighbors to the facility. However, some of the mothers fear attending the health facility however, we convincingly tell them to go to the health facility for delivery.

**D3:** they will not attend ANC follow-up due to lack of knowledge; for example, they think ANC follow-up lack privacy and exposes women's privacy. The mother will face many problems if she has not attended ANC follow-up.

**Moderator-**What are the barriers to accessing ANC?

**D5:** lack of transportation and money for transportation are the reasons for not having ANC follow-up. Previously mothers deliver at home and they will suffocate her from the air. Additionally, they will cut the umbilical cord improperly using available sharp materials. Also, the blood will retain in the mother's uterus resulting in an abdominal crump. Currently, after they are exposed to health education they are delivered at the health facility due to they are healthy and not dying, even they will be referred if there are any problems.

**Moderator:** Are there any socio-cultural-related barriers in your community?

**D5:** previously they haven’t knowledge but now they are getting an education. HCPs are encouraging mothers to start follow-up early. Previously when a woman gets pregnant they will hide her to prevent others not to see her. However, currently, mothers are motivated to attend ANC follow since the community is exposed to education.

**D2:** nowadays HDAs present in all villages and they will give education to the women to attend health facilities, all mothers have leaders who specifically follow them. For example, it has been 16 years since I get married. When I get pregnant with my first child a community member inform me to deliver at home and I gave birth at home due to there is no strong service at the time. However, my second and third child was delivered to the health facility. When I gave birth to my first child, they suffocated me with a lot of clothes and they smoked inside the house. The traditional birth attendant shakes me to deliver the placenta using traditional methods. However, I delivered my second and third children to the health facility without any problems. Nowadays, everybody motivates mothers to give birth at the health facility since there is a better service. In addition, currently, we have an agreement that if a mother delivers at home, the family members will be arrested, so they fear and will bring the pregnant mother for delivery at any time.

**Moderator:** Is there any quality of care-related barriers in your community?

**D9:** The mother will get service when she goes to the health facility and there is also home-to-home service. There are no mothers who come to health facility and goes back due to lack of service. The problem is on the side of the mothers because they don’t come but if they come they will get the service. They will not attend the health facility due to a lack of knowledge.

**Moderator:** Why do women go to the facility for first ANC, but discontinue for subsequent ANC visits?

**D3:** after attending the first visit they will discontinue because they forget their appointment day. Then they will come to the health facility after prolonged labor. The service is available every day but they forget their appointment, the service providers are always available. They will discontinue because of their problem. They will only go for the service if they have prolonged labor. But, currently, we have an agreement that the male partner will be punished with 500 birrs if his wife gives birth at home. If a mother delivers at home the father and the traditional attendant will be punished with 500 birr. So they will bring the mother to a health facility to deliver the placenta otherwise they will be arrested and punished. They will bring her using a motor or cart even after the baby was delivered at home to remove the placenta but they will be punished. Generally, they will discontinue due to the mother's lack of knowledge but not because of the service's unavailability.

**D1:** they will give an appointment card. If that mother can read she will remember the appointment unless she will give it to others who can read and tell her the appointment day. She is the one that must care for her; there is a saying in Sidama culture **“Luboona qamato anu dada’lano”**. I have to give more focus and attention to the side of the mothers rather than the service providers. It is my responsibility to remember and attend each visit. If the mother is undernourished and has anemia they will give her the medications. Also, they will educate us during our forum meeting. The HEWs will give us medication and food if the mother is undernourished. Not attending and discontinuation of the ANC follow-up is our problem not the HEWs.

**D8**: They will inform all pregnant mothers to start ANC follow-up during home-to-home visits by HDAs. The discontinuation is because of the fear and lack of knowledge.

**V. Traditional practices during pregnancy**

**Moderator:** Can you tell us about the traditional practices and beliefs during pregnancy?

**D5:** they will give **“Amessa”** to the newborn if the newborn has a rash. The traditional birth attendant will inform us how to feed the newborn. The children could be hurt if they give ‘Amessa’ at an early age.

**D1:** if the mothers gave birth at home and placental retained. To remove it they will hold her up for two times and they will hobble her. The attendant will hold the mother up on her back and she will wake the mother up and down several times. When the labor is prolonged, they will make the mother drink **the "Soicho”** plant. However, currently, there is no association between tradition, religion, and delivery, and there is no such kind of problem currently.

**Moderator:** Do you think these traditional beliefs, religious practices, and cultural norms affect mothers to use care during pregnancy? Explain how and why?

**D9:** traditionally, a pregnant mother will smoke in her house and she will be told not to come outside. Nowadays all pregnant mothers can go even to health facilities for service.

**D3:** now everybody knew that there is no relation between tradition and delivery and no one will accept the thought of the tradition.

**D2:** when I was pregnant, I was told that I have blood group incompatibility and they told me to give birth at the hospital. I discuss it with my friend and she told me that she also have a similar problem and advised me to ignore their advice and get prayer from a prophet but I did not accept her idea and delivered at the hospital.

**Moderator:** How do you see community volunteers/TBAs and health professionals and maternal health services provided to the community?

**D9:** the HDAs will be oriented by the health providers to identify pregnant mothers in their vicinity. Since they identify the pregnant mother, the mother with malnutrition, and children with illness and malnutrition during home-to-home visits so they are very important for the service. They also link the identified mothers and the children with the health center to get food and other medication. The HDAs are also helpful for mothers to get family planning services within 45 days after delivery.

**VI. For recently delivered mothers only**

**Moderator:** How do you rate the quality of care you received during ANC follow-up? What kinds of services do you receive in ANC? Are you satisfied?

**D1:** previously, I am sick having malaria symptoms, and went to Leku Hospital and they told me that I was pregnant. I started in the fourth month of my pregnancy. After taking two TAT vaccinations I went to Leku Hospital for an ultrasound to check the position of the baby. I got nutritional advice from the healthcare workers, to eat selected foods adequately. I completed my follow-up and gave birth to my baby at this health center. When I come to the health center at three o'clock with labor the service providers are available and delivered at six o'clock. They felt my pain, gave me the best service standing for long hours, and enabled me to deliver safely. Only two health professionals attended the delivery, they never let in other individuals but they let in other healthcare workers freely. In addition, the cleanness of the blanket they gave me was poor. The cleanliness of the health center is very poor and the mosquitoes in the waiting room are causing a problem even several mothers went back to their homes early due to this problem. After the delivery, when I got back after 45 days they gave me vaccination and family planning service but post-natal care was not provided.

**Moderator:** Explain to us your experiences relating to the utilization of ANC care provided by skilled birth attendants.

**D5:** they gave medication for abdominal pain, they give a cape for the baby after delivery, they gave me a drug for my blood and they will refer me if I have a problem beyond their capability.

**D7:** during my ANC follow-up, they will follow up and address if I have anemia and or blood pressure. If the health center did not avail the drug, they will make us buy from a private pharmacy which is a problem for us. Previously pregnant mother attending delivery was given soap, a bed net, and clothing for the newborn but nothing is given currently she just goes home.

**D6:** some times when we come for ANC service they are ill-tempered and they are not welcoming at all. We only go there for the service, not for living, so they have to improve this behavior and they must provide the service to the mother with sympathy.

**D4:** HEWs give us the service with high care during ANC follow up but the health workers at the health center do not give similar service during ANC and delivery. The service is not satisfactory at provided by the healthcare workers at the health center is not satisfactory. I brought my pregnant neighbor they investigated her with hearing aid material and only told us to go to the private clinics, the healthcare workers are not doing a good job. Healthcare workers give appointments for a week without investigating the mother and this is making mothers discontinue the follow-up and deliver home. Pregnant mothers are getting very poor service at the health center level.

**D9:** when a pregnant mother goes to HC it is difficult to get the service even to get a service card but it is smoother at HP with HEWs. The laboratories also have a major problem during urine tests; they usually say that there is no chemical to do the tests. They have no tests for urine and stool, they only test for our blood, and they do not have a test for STIs. They only do pregnancy tests and nothing other during follow-up. There is also a problem with medications; they usually prescribe drugs to private pharmacies and the drugs are not available usually.

**D1:** they usually prescribe drugs to buy from private pharmacies which are very expensive for the mothers; even most of the pregnant mothers come with no money for the service. Not alone other drugs Amoxicillin is not available at the HC. When the pregnant mother is poor, she could not buy the drug from private pharmacies and she will not take it. Some of the pregnant mothers even did not have money for transport and they may even do not have anything to eat at home. Government officials usually inform us "Mother must not die while giving birth", but the service provided at the HC is poor. Generally, the HC is not giving good service, particularly to pregnant mothers.

**D3:** during ANC follow-up, they only investigate our blood they did not investigate our urine and stool. When I was pregnant the last time, I was diagnosed with a urinary tract infection in the eighth month but they told me to buy the drug from private clinics that is very expensive at the time. They did not have the examination and the drug.

**D1:** previously bed nets were always given to all pregnant mothers but currently they are not giving us for one year. Because of this, many pregnant mothers are suffering from malaria. When pregnant mothers got malaria, it will be severe quickly and they are usually referred to Leku hospital for management. Pregnant mothers are usually at high risk for most of the diseases. We did not get bed net in the last five years so the government should see our problem and provide us soon.

**D2:** healthcare workers at the HC usually are not compassionate to the mothers who are from rural areas. When they become angry without reason towards us, we usually went to traditional birth attendants for her service. For example, one mother who encountered a healthcare worker with poor behavior immediately went to a traditional attendant and they massage her abdomen to correct the position of the baby she aborted the baby in the process.

**D9:** pregnant mothers from rural areas somewhat they may have poor personal hygiene and the health workers usually undermine them. These problems usually occur in the delivery room. When the healthcare workers demoralize the pregnant mother, she usually did not want to go there again for any service. They even did not provide us with health education to improve our hygiene.

**The practice of PNC**

**Moderator:** How early do women go for PNC?

**D3:** we usually go to the HF after 45 days for child immunization and use family planning services. Afterward, I will return based on the appointment given for my baby's immunization program.

**D9:** the mother might return any time before the 45^th^ day if the baby has a rash or high-grade fever.

**Moderator:** Do women think skilled attendance during postpartum help their babies and themselves?

**D8:** it helps us to learn how to breastfeed, and how to wash our baby.

**D7:** they will give us HE regarding complementary feeding after the sixth month.

**Moderator:** Explain factors that would motivate women to utilize PNC services in their childbirth

**D3:** The reason why we come for the PNC is not due to the encouragement of the health workers rather we return for the sake of our and the babies' health. Their service did not encourage service utilization at all. HEWs workers encourage us to deliver at the HF, and not to feed other foods before the child reaches six months. They also taught us how to properly breastfeed our children. Generally, only the HEWs encourage us to utilize health services.

**D8:** nowadays all pregnant mothers give birth at health facilities.

**Barriers to attending PNC use**

**Moderator:** If women don’t go for PNC, what are their reasons?

**D9:** due to a shortage of cash for transportation she might be unable to go for the service and they usually referred without the service this makes the mother not use the service.

**D3:** even when we reach using a motorbike and the ambulance drivers will not return us. The motorbikers usually ask for two or three hundred birr for transporting pregnant women. Moreover, some areas are inaccessible for motorbikes. The ambulances will not come to the HP but usually, they refuse to come unless the HEW calls them. There is a huge transportation problem for pregnant mothers.

**Traditional practices during pregnancy**

**Moderator:** Can you tell us about the traditional practices and beliefs during the postpartum period?

**D3:** the community thought when the baby is exposed to another person's eye early days he/she might be eaten by “Buda”.

**D5:** when a rash or other illness occurs on the mother or the baby they think it will heal by itself, they believe exposing the mother and the baby in the early days is bad for their health. However, this belief vanished gradually, and currently all mothers and newborns are going to the health facility for their illness or follow-up.

**D9:** previously the mother use traditional practices but now no mothers practiced traditional feedings like ‘Amessa’, and cow milk. They will not give other food until the newborn reaches six months.

**For recently delivered mothers only**

**Moderator:** How do you rate the quality of care you received during PNC follow-up? What kinds of services do you receive at PNC? Are you satisfied?

**D3:** HADs are giving us support regarding the service. They have the list of mothers who deliver currently and they remind us to attend the PNC. They also remind us of the day of immunization for our baby.

**D9:** HEWs and HDAs are working together smoothly. The HEWs gave training for HDAs to identify malnourished and the HDAs identify mothers and report to the HEWs for treatment. The HEWs train HDAs on anthropometric measurement, they will measure all mothers and children, and if they find a malnourished mother or child they will send them to the health center for treatment.

**D3:** generally, HDAs are the backbone of the service and our health. They always remind us to use all health services.

**Moderator:** In your opinion, what should be improved regarding PNC services?

**D1:** if our health is ok until the 45^th^ day after delivery we may not go to the HC. During immunization, we go to the HP at two o'clock but the HEWs bring the vaccine with the carrier around four, which is very boring. Family planning services are also given after a long waiting hour which is difficult for several mothers, this could expose the mother to unwanted pregnancy. There is a long waiting time for all services.

**D9:** the HEWs are living in Leku, Hawassa, or Yirba so they come to the HP at noon after we waited for hours which makes the service utilization difficult. Moreover, Immunization service is provided only once a week on Wednesdays, which is difficult for mothers.

**D2:** there is a shortage of family planning commodities for more than two weeks, when there is no Depo at the HP we are forced to use them at private clinics paying 100 birrs, if the mother did not have that birr she might be exposed to unwanted pregnancy.

**FGD: 02**

1. **The practice of ANC**

**Moderator:** How early do women go for ANC?

**D6:** she will start the follow-up and vaccination starting from the fourth month. Then she will return after eight months. Finally, if her time for delivery reaches she will be back. Moreover, if she has any health problems she will be examined. She will return within two months after delivery for immunization.

**D4:** we will say it is late when she starts the follow-up after six or seven months. When she started follow-up late, she will be at risk for many health problems. In my experience, my wife started the follow-up late in the seventh month, but the service was good. The baby born in the health facility was healthier and special. Delivering at the health facility is very important for the mother also.

**Moderator:** How often do they go to ANC?

**D1:** when she goes to the follow up they will examine her health, position, and presentation whether it is on her head or buttock, or leg. She will be examined and investigated for necessary health conditions. Currently, most of the pregnant mothers go for follow up out of ten mothers nine will go for follow-up. Health education is helping us a lot, it is provided by the HDAs. Using the health education by the HEWs mothers are delivering at the health facility. All mothers are practicing all the health extension packages.

**Reasons for discontinuation across the continuum**

**Moderator:** Why do women go to the facility for first ANC, but discontinue for subsequent ANC visits?

**D9:** currently all women will follow up regularly, pregnant mothers have coordination with the development army and the home-to-home visit will not allow the discontinuation of the follow-up. No pregnant mother will be left at home without ANC follow-up. We have a pregnant mothers' forum every 28 days and mothers who delivered at home will be punished with 500 birr. Every mother did not want the punishment so all women are giving birth at the health facility. The one-to-five team head is responsible for bringing the pregnant mother to the health facility. Currently, we can say all mothers are giving birth at HC. At the HC they will be examined their blood and urine and the service is very good so the pregnant mothers are going according to their appointment. The HPs are referring to the HC if the mother has a problem. Previously children did not go to the health center for the service, most of the children's hygiene is poor and they are **“Koshsha”**. Previously the HDAs are going home to home for remembering the service but now all pregnant mothers are going by themselves. Nowadays most mothers deliver at the HC. Previously due to our poor knowledge, we give birth at home.

**Barriers to attending ANC use**

**Moderator:** If women do not go for ANC, what are their reasons?

**D2:** in our country the reason for discontinuation might be due to that we are a pastoralist community and we shift areas. However, when we settle, all mothers start the follow-up. All permanent residents of the vicinity will give birth at the health facility.

**D4:** the HP might be far from their home, so they might have transportation problems. The HEWs use community health volunteers for regular follow-up. When the labor starts at night, there will be a transportation problem and this could make the mother give birth at home. The community health volunteers create awareness about the importance of delivering the HC during their home-to-home visits.

**D**5: previously due to lack of knowledge mothers delivered at home but now due to the hard work of the community health volunteers all mothers are giving birth at the HC. Nowadays we have waiting rooms and pregnant mothers are going early even five days for delivery. When the mother stays in the waiting room and gives birth peacefully, the waiting room itself is creating awareness in the community.

**D3:** due to a lack of awareness most of the community did not go to the HC for delivery, they usually say “Koni albani amau fayimate uurinshshara illete agentino”, and they give birth at home. Nowadays community thought and awareness changed, pregnant mothers are staying for five or six days in the waiting room, and pregnant mothers are lucky now. Staying in the waiting room is becoming a good experience for mothers in the village. When the pregnant mother faces a problem at any time, we will bring her to the HC using a motorbike or ambulance.

**Traditional practices during pregnancy**

**Moderator:** Can you tell us about the traditional practices and beliefs during pregnancy?

**D2:** the wife of the young's are going for follow up but the wife of the old ones are not going regularly. All my children are born at home. But my daughter-in-law deliver at the HC, the labor started at nine and we bring her to the HC using a motorbike, and delivered peacefully. My second daughter in law labor started at six in the night, then I took her to the HC using a motorbike and she give birth peacefully, the children born in the HC are “Busulle”. The newborn will get food when the delivery is at the HC, so all community members are advising each other to use the HC delivery service. Previously, the children of Sidama people did not go anywhere like Addis Ababa or Hawassa because the placenta was buried in their home. Nowadays all people knew the importance of delivering at the HC.

**Reasons for discontinuation across the continuum**

**Moderator:** Why do women go to the facility for first ANC, but discontinue for subsequent ANC visits?

**D7:** we are a pastoralist community we move from place to place so it could the reason for the discontinuation. Additionally, pregnant mothers are weak to travel long distances for the service. All young knew the family planning service now. The youths are learning about family planning and birth spacing at school or the church and they are in return they are teaching their parents. The main reason is the long distance of the HP; the HEWs also did not live around our area so it is difficult to get the service at night. Therefore, the government must build another health center around our vicinity. Since we achieved our regional government request, the regional/federal government has to build another health facility that can be reached within 15 minutes. The location of the facility is the main reason for discontinuation but the service provided by the HEW is very important. Previously the mother give immediately after giving birth but now they are using family planning and they are now able to space the birth. The mothers are giving birth with their self-control since they are now practicing birth spacing. We have to thank the government for providing a bed net for pregnant mothers first; the HEWs are currently collaborating with the government in distributing the bed net. Our kebele is very wide and it could take half to full hour travel to reach the health facility.

**Traditional practices during pregnancy**

**Moderator:** Can you tell us about the traditional practices and beliefs during pregnancy?

**D4:** traditionally pregnant mothers are responsible for cleaning the dung of the cattle by carrying it on their backs which is harmful to the mothers. All preventing pregnant mothers from taking random drugs, they have to be examined first. Currently, there is no traditional practice in our area.

**D3:** the traditional practice in our vicinity is when the pregnancy is unwanted they will drink traditional medicine to abort the pregnancy. They are also taking unspecified drugs for abortion, nowadays the practice is diminishing significantly. Now, the shortage of food is a major problem. Previously, FAFA is being provided for pregnant mothers but it is not adequate.

**D5:** previously pregnant mothers have to deliver at home without the knowledge of the community, which is very harmful to the mother and the baby. Now the mothers are delivering at the health facility but now due to climate change, we are facing food insecurity. The food support provided by the government is not adequate, it is better if the rains come again. The Plumpy nut given to the children is very important, the children are improving quickly. Previously Female genital mutilation is a harmful traditional practice here.

**D2:** widower and single mothers are giving birth at home because of the fear of social stigma they might face during seeking the delivery service.

**Moderator:** How do you see community volunteers/TBAs and health professionals and maternal health services provided to the community?

**D6:** HDAs taught us about removing stagnant water, using a bed net, and using a latrine. Additionally, they inform us to take pregnant mothers to the HC.

**D5:** the HEWs educate the HDAs to do home-to-home visits at all villages and teach pregnant mothers to use ANC. When a pregnant mother starts labor the HDAs will call for the HEWs to send an ambulance. Until the pregnant mother reaches the time of delivery, HDAs follow her individually.

**For recently delivered mothers only**

**Moderator:** How do you rate the quality of care you received during ANC follow-up?

**D9:** we are getting a very good service. The HC and HP are doing integrated work and they are giving education about delivering at the health facility. Previously the HDAs are informing pregnant mothers regularly to attend the follow-up but now the pregnant mother themselves are going. Since the service is provided free of cost they are encouraged to start the follow-up, they are happy with the free service. Previous HDAs are not available and we are not getting the service. Pregnant mothers are getting the service based on their appointment. My child whom I delivered at home is weak in education but my son's child is very intelligent because he is born in HC. The health post did not have electricity and we cannot afford generators. If electricity and water are available every pregnant mother delivers here.

**Moderator:** If the mother received ANC; Ask: Explain factors that motivate you to utilize ANC service in their pregnancy

**D7:** I delivered my last child at home because of my poor knowledge but after I become HAD I told everyone to deliver at the HC. Previously there was a large number of maternal mortality but now there is no maternal mortality. The service itself is attracting pregnant mothers.

**D4:** HDAs are continuously giving health education for initiating ANC follow-up and facility delivery. They teach that if you did not follow and get vaccinated you may face health problems. We have a pregnant mothers' forum two times a month. They are providing health education regarding personal hygiene and health service utilization. Moreover, they teach us to vaccinate our children by comparing the health of a child who is immunized and a non-immunized child. In addition to the HDAs, the HEWs, and healthcare workers from the woreda office are also providing education. They are providing good service; the main problem is the accessibility of the facility due to the long-distance travel and lack of electricity. Health service is more important than the education service.

**Moderator:** Explain to us your experiences relating to the utilization of ANC care provided by skilled birth attendants.

**D3:** previously going to a health institution is believed to be bad but now the service is very good and the community is also changing its perception towards the utilization.

**In your opinion, what should be improved regarding ANC services?**

**D6:** we only have one HC for four kebele the government must hire midwives nurse for the health post to improve the service for pregnant mothers.

**D8:** one of the residents our kebele who is a daily laborer his pregnant wife has labor and the motorbike refuses to take her for free so we collect money and pay for it. This means the transportation problem due to the long-distance travel to the institution is the major problem causing difficulty in using the delivery service.

**The practice of facility delivery**

**Moderator:** Do women think skilled attendance during childbirth helps themselves and their babies?

**D1:** when they give birth at the health institution there is no bleeding but when they give birth at home they will have heavy bleeding. It is believed that she has "Gobarichchi kision". Therefore, if she is delivered at HC there is no bleeding after delivery and both the baby and the mother will be healthy.

**Reasons for use of facility delivery**

**Moderator:** Explain factors that would motivate women to utilize delivery services in their pregnancy Probe for reasons for using a continuum of care

**D7:** the symptoms of labor symptoms are the main reason for seeking the service.

**D6:** the presentation of the baby, blood pressure measurement, information, and expected date of delivery. and symptoms of labor are the reasons that make the mother seek the service. In addition, the HDAs members also encourage the mothers to utilization of this service.

**Barriers to attending facility delivery**

**Moderator:** If women deliver at home, what are their reasons? Explain the constraints that influenced women to utilize facility delivery services.

**D5:** the delivery at home because of the cost of transportation, and the availability and cost of food at the health facility. Generally, it is related to financial problems.

**D2:** when the labor starts at midnight the cost the transportation will be folded three times up to a hundred birr for each person, and most of the pregnant mothers even do not have any money. They give birth at home due to the cost of the transport since our kebele is the farthest from the facility and the cost of food during a stay at the waiting room is also very expensive for most of us. In addition to the distance, the lack of electricity and water at the HP is also forcing mothers to deliver at home. Previously mothers who gave birth will not take a shower for at least ten days causing poor personal hygiene but now all mothers will take a bath after they gave birth which improves their hygiene. The mothers who gave birth at the health facility have good hygiene “su’nitino, biffino”.

**D4:** Mothers who have many children usually deliver because their labor is quick and we may not be able to reach the facility. Moreover, elder mothers will become shy and reluctant to give birth at the same place their daughter was delivered.

**Reasons for discontinuation across the continuum**

**Moderator:** Why do women go to the facility for ANC, yet mostly deliver at home?

**D4:** single and widower mothers will not go to the facility for delivery because they fear; when they are asked for the father's name, they will be shy.

**Traditional practices during the intrapartum period**

**Moderator:** Can you tell us about the traditional practices and beliefs during childbirth?

**D1:** they will cover the mother with a blanket during the labor but now the belief and the awareness of the community improved, the mothers are giving birth at HC. Previously the community believe that when the child was delivered at the health facility its umbilical cord will be buried there which is believed to be bad luck for the baby but now this belief is not acceptable.

**Moderator:** How do you see community volunteers/TBAs and health professionals and maternal health services provided to the community?

**D5:** HEWs and HDAs are working together to help us. They are conducting home-to-home visits to provide us with health education. The HDAs also call the ambulance when pregnant mothers have labor. One to five team leaders have also supported us regularly. Health professionals sometimes collect money for poor delivery for transportation.

**D3:** each HDA has twenty team members and they are working with HEWs, the leaders have a book for noting the expected delivery period of each member, they will also give us brochures with delivery process information. The brochure shows the growth progress of their baby each month.

**For recently delivered mothers only**

**Moderator:** How do you rate the quality of care you received from the facility during childbirth?

**D5:** the HC is giving a great service; they refer the mother to an ambulance if her problem is beyond their capacity and they give treatment food for mothers with malnutrition. They are distributing drugs for the prevention of eye disease and the chemical “Wuha agar” for treating drinking water.

**D4:** they taught the mothers to exclusively breastfed their baby until the sixth month and to give him additional meals after the sixth month.

**Moderator:** For home-delivered mothers; what does she think are the obstacles when accessing a health care facility?

**D7:** we delivered at home because of a lack of knowledge due to the unavailability of health education providers at the time. Previously the delivery service provided at the HC is not good enough now they are giving quality service. They give every laboring mother intravenous glucose which is very helpful for her health.

**Moderator:** Explain to us your experiences relating to the utilization of facility delivery care provided by skilled birth attendants.

**D1:** nowadays the belief of the community changed. The community is taking well every strategy the government working on. The HC is acting as a bridge between the government and the community. Every pregnant mother wants to deliver at the health facility because they are giving the mother wheat flour.

**Community Perceptions about health providers and maternal health programs**

**Moderator:** How do the communities see the maternal health programs and health professionals? Tell me your perception of maternal health care services and your perception of different care providers.

**D6:** there is a huge shortage of healthcare providers. When four mothers come for delivery at the same time, only one professional will be available at the time and it is difficult to manage. The government has to increase the number of professionals. When the HCs refer the mother to Yirba Hospital, the hospital itself refers the mother to Hawassa referral hospital, which is difficult for the mother. Why does the government add health professionals? The professional that is working at Hawassa must be available at Yirba Hospital. When we take the mother to Hawassa she might die on the road.

**D9:** there is a shortage of health workers at the health center, one time I took three mothers at the same time which is difficult to manage by one worker and I participated in the delivery. Even some times the healthcare worker will be unavailable in the delivery room and we are forced to assist deliver the baby several times. I confronted the professionals many times but they have huge attitude problems. Water is not available at the facility.

**D2:** when we take the laboring mother to the health center they come late. They always say the time is early for the delivery but most mothers deliver immediately. The healthcare workers at the health centers have attitude problems.

**The practice of PNC**

**Moderator:** How early do women go for PNC?

**D1:** she will go on the 45^th^ day. After the mother and the child were vaccinated they will be appointed again.

**Moderator:** How often do they go to PNC?

**D6:** if she went after the 45^th^ day she will be considered late but if she went on the 45^th^ day it is considered as the right time. If she presented later than 45 days, the immunization and other services may not be useful.

**D7:** they will start the post-natal follow-up on the 45^th^ day and they will be again appointed to come again after 28 days. Sometimes Depo injection will not be available and we are forced to pay 80 birr for the service at private clinics. When this happens the mothers believe family planning medication will not be available always and eventually will stop the post-natal follow-up. After delivery shortage of family planning drugs is the major problem.

**Traditional practices during pregnancy**

**Moderator:** Can you tell us about the traditional practices and beliefs during the postpartum period?

**D4-** previously the community uses “Amessa" for newborn babies but after the health education provided; we only give breast milk until the sixth month. Currently, this practice completely stopped. After the government provided health education repeatedly the community changed its awareness.

**Moderator:** How do you see community volunteers/TBAs and health professionals and maternal health services provided to the community?

**D7:** HDAs always visit the mother after delivery at home and if the mothers have any problem they will communicate with the HEWs and they will address the problem.

**For recently delivered mothers only**

**Moderator:** How do you rate the quality of care you received during PNC follow-up?

**D4-** at the health center level the service is unsatisfactory during post-natal care: necessary drugs are not available; the health workers are willing to serve.

**D9-** previously FAFA and plumpy nut were given to malnourished mothers and children but now the service completely stopped.

**Moderator:** in your opinion, what should be improved regarding PNC services?

**D6:** family planning drugs must be available, and clothes for the newborns, the government has to encourage the HDAs. The "koshoro" given to malnourished children is exposing their teeth to early dental caries. The biscuit is very sweet which is making their teeth health bad.

**D5:** previously soap was distributed by the government but it also stopped. This service has to resume soon since our living is becoming poor.

**D1:** flour for porridge has to give to the community. Bed net will give during discharge for all newly delivered mothers.

**FGD: 03**

**Antepartum for all participants**

1. **The practice of ANC**

**Moderator:** How early do women go for ANC?

**D4:** After 6 months, the pregnant mother will begin her follow-up in the health facility accordingly to her appointment.

**D5:** After 3 months, the pregnant mother will begin her follow-up in the health facility.

**D3:** if a pregnant mother comes health facility at 3 months, we say she got early her ANC but if a pregnant mother comes health facility after 6 months, we say she got lately her ANC.

**Moderator:** How often do they go to ANC?

**D2:** during her pregnancy time, she will visit the health facility at least 3 times.

1. **Reasons for the use of ANC**

**Moderator:** Do women think skilled attendance during pregnancy helps their pregnancy?

**D1:** pregnant mother will follow her and the baby's health in addition to the position of the baby, health education regarding how she sleeps, and how to feed nutritional advice in the facility.

**D8:** in the health facility she will get nutritional advice, and wear styles (not tight clothes) during her px time.

**D5:** she must get nutritional advice during her pregnancy time.

3. Explain factors that would motivate women to utilize ANC services in their pregnancy?

**D5:** when the pregnant mother get visited the health facility, she will have health education that makes motivate her.

**D8:** Health development armies are motivating factors for pregnant mothers.

**III . Barriers to attending ANC use**

**Moderator:** If women do not go for ANC, what are their reasons?

**D6:** all pregnant mothers will visit the health facility.

**D1:** all pregnant mothers should visit health facilities b/se integration of HDA and HEWS accordingly their "got" there is saying" no mother should not die when giving birth”. So, all mothers should attain health facilities.

**Moderator:** What are the barriers to accessing ANC?

**D2:** she will teach during pregnancy time, but pregnant mothers are busy with household work and will be absent usually.

**D5-**inaccessibility of transportation is a problem; since our village is inaccessible to ambulances we take pregnant mothers using carts. The travel to the facility is taken more than an hour.

**Socio-cultural**

**D4-** previously delivering at HF is believed to be bad culturally but it is now stopped. The community believes the babies' umbilical cords have to be buried inside the house, but this belief stopped after the health education. Additionally, society believed others should not hear the laboring mother's voice. Nowadays, the government is teaching us that a "mother should not die while giving birth" and HDAs and HEWs are working together to follow the pregnant mother in each village.

**D7-** the problem is due to the long-distance travel to the facility. Road infrastructure problem is also a major obstacle, several mothers delivered on the road while traveling to the facility. Plus at the health center, there is no electricity they are using solar lights. Absence of the health professionals is also a problem.

**Reasons for discontinuation across the continuum**

**Moderator:** Why do women go to the facility for first ANC, but discontinue for subsequent ANC visits?

**D7-**they discontinue the follow-up when they feel ok. But if she faces any symptoms the mother will go for follow-up.

**D6-** previously they waited for TBAs for delivering but currently this practice is believed to be shameful. When she gave birth at home the blood and other “Koshasha" will retain in her uterus, and the retained blood "Hanu" will expose her to abdominal pain.

**Traditional practices during pregnancy**

**Moderator:** Can you tell us about the traditional practices and beliefs during pregnancy?

**D7-** going to the traditional birth attendant “Ogette” is the main traditional practice.

**D2-** the traditional birth attendant “Ogette" will look for the position of the baby and she will massage the mother's abdomen. However, this practice is stopped.

**D7-** during home delivery, the TBA will cut the umbilical cord using a blade which is harmful to traditional practice.

**D4-** the pregnant woman will be given "soicho” to drink. Soicho is prepared from the herb, it will be shacked with water and the mothers will be forced to drink. This is a harmful traditional practice.

**Moderator:** How do you see community volunteers/TBAs and health professionals and maternal health services provided to the community?

**D1-** the HDAs knew the number of pregnant mothers in each village. They will register all pregnant mothers and report to the HEWs. They also knew the mothers who reached the viability period.

**D5-** the HDAs knew the number and each of pregnant woman and they give health education to all for follow-up.

**D7-** when we deliver alt home the HCWs will give us FAFA. The FAFA was given to us three times until we gave birth. They also provide a plumpy net to our children.

**Moderator:** How do you rate the quality of care you received during ANC follow-up? What kinds of services do you receive in ANC? Are you satisfied?

**D2-** the service quality is moderate. The HWs do not provide the FAFA to all pregnant mothers. The food is given only to the mothers whose MUAC are less than 21 cm, the mother with MUAC greater than 21 cm will complain at the HWs. When we told them the criteria they still complain about not receiving it.

**D8-** the food must be given to all pregnant mothers, rather than selecting using MUAC. The pregnant mother who took the FAFA will be happy but those who did not take them usually curse the HWs.

**D4-** the service quality is medium. Generally, the mothers are not happy with the distribution plan and criteria of the FAFA distribution.

**Moderator:** Explain to us your experiences relating to the utilization of ANC care provided by skilled birth attendants.

**D1-** it is good. The service is good; the professionals are compassionate and give the service respectfully.

**D2-** the ANC examination is done in a separate room privately.

**D4-** since the HEWs is born in our Kebele; all pregnant mother is the relative of the HEWs, they will never complain about their hygiene, they consult her regarding personal hygiene.

**For community religious leaders and community volunteers only Community perceptions about health providers and maternal health programs**

**Moderator:** How do the communities see the maternal health programs and health professionals?

**D4-** the community perceived the service as good. The advice provided by the HWs is very good, they will not complain if the mother has poor hygiene. Even if the mother's "emana" is dirty the midwives will teach her about personal hygiene. The newborn are also healthy and they advise her to feed their child correctly and neatly.

**D2-** previously we do not have a health facility in our vicinity and several mothers died while giving birth. But now the mothers are when they get pregnant since the service is available in our kebele. They are giving us a good service.

**D1-** there is a saying “meate godoburo, labbahuno ledo godowano yano". Currently, there is no problem, the workers at the HC are giving us advice. Households without latrines and those who gave birth at home are seen as peculiar. Our father and mother did not live a good life but now we are living a healthy life.

**Moderator:** In your opinion, what should be improved regarding ANC services?

**D6-** lack of electricity is the only problem. The HWs are delivering our mother while holding a torch in their mouth.

**D1-** since there is a shortage of Depo our mother is having an unwanted pregnancy. Why our government did stop the Depo injection service? The private clinics are asking for up to one hundred birrs per injection.

**D5-** lack of electricity in our health facilities is the major problem we are currently facing. When the healthcare workers' phones are switched off, it will be difficult to call them for the service. Our government has to make electricity available.

**D8-** when I took one laboring mother there is no electricity, so she refused to deliver at the health facility without light and I took her to another facility-using cart.

**D7-** the problem I noticed is the health workers did not send the delivered mother immediately, they say we have to watch her for hours. But the mothers complain anyway.

**Intrapartum for all participants**

**The practice of facility delivery**

**Moderator:** Do women think skilled attendance during childbirth helps themselves and their babies?

**D1-** when the laboring mother has a problem more than their capacity they refer the mother to the hospital.

**D2-** the service is good. They will measure her blood pressure, they will prevent excessive bleeding. They also serve the newborn in a good way; they will examine.

**D7-** when the baby is distressed they will send the mother to the hospital.

**Moderator:** Explain factors that would motivate women to utilize delivery services in their pregnancy

**D5-** the healthcare workers are saving the life of our wives and children. When the pregnant women deliver at home she might die.

**D1-** previously when the mother deliver at home, the baby will be hurt too. They discard the clostridium "gora”. Previously we did not give additional food to our infants but now we start giving at the sixth month. We are getting good nutritional advice.

**D8-** all mothers are aware currently, the pregnant mothers themselves are forced to the utilization of the delivery service. They say I have to go to the facility early.

**Barriers to attending facility delivery**

**Moderator:** If women deliver at home, what are their reasons? Explain the constraints that influenced women to utilize facility delivery services.

**D9-** the educational capability of children who are delivered at a health facility is much higher. So, the fathers are also changing their attitude. Luck of knowledge and fear are the main reasons for giving birth at home. When she delivers at home, they cover her with “sema”. They also say our parents deliver at home so we also have to give birth at home. It is a bad practice.

**D1-** road infrastructure and transportation problems are the major problem. Plus only one ambulance is available for our woreda.

**D2-** they forget their expected day of delivery and give birth at home sometimes. Sometimes when she is at home alone she could give birth at home since the facility is located far.

**Traditional practices during the intrapartum period**

**Moderator:** Can you tell us about the traditional practices and beliefs during childbirth?

D8- the mother believes a new air/wind is bad for the laboring mother. They also say that male workers deliver the baby which makes them fearful. Delivering at home will expose the mother to placental retention.

**D5-** traditionally the laboring mother has to hide from others, and also they cover her with four or five blankets distressing her. When she gives birth at home; she might die and the baby also could die.

**Moderator:** Do you think these traditional beliefs, religious practices, and cultural norms affect mothers to use care during delivery? Explain how and why?

**D1-** if it is an illness; the religious leaders will pray for her but if this is labor she has to go to the health facility.

**Moderator:** How do you see community volunteers/TBAs and health professionals and maternal health services provided to the community?

**D9-** the HDAs will encourage mothers to go to HC for delivery and they will follow and advise her. If the mother fears getting the service, they will advise and encourage her.

**D5-** HDAs and HEWs are working as hand and glove together. The HDAs encourage mothers while the healthcare workers provide the service.

**Moderator:** How do you rate the quality of care you received from the facility during childbirth?

**D8-** the HWs give private rooms for the mother while giving birth. Only one healthcare worker attends the labor and delivery.

**D5-** only the service provider and the laboring mother will enter the room. They will wash the mother after the delivery. The service is very good in general.

**Moderator:** How do the communities see the maternal health programs and health professionals? **D1-** the community is very satisfied with the service. Previously when the mother gave birth to a male baby the mother will take bathe after four days. If it is a female baby, she will take a bath after three days. Nowadays the mother and the newborn will take the bath without restriction.

**D2-** all community members are very happy with the service. Maternal mortality and bleeding during delivery decreased.

**D1-** the society is very happy with the availability and also with the service provided by the HC.

**Moderator:** **In your opinion, what should be improved regarding facility delivery services?**

**D6-** we do not have a problem

**D2-**the problem is the easy availability of the ambulance. When the ambulance becomes late the mother might give birth on the way. The drivers have attitude problem.

**D1-** we have a problem regarding the service providers. They are providing us with a good service.

**D9-** they are not giving cloth for the newborn baby. They are not giving bed nets; the newborn might be at risk for malaria. The provision of soap also stopped.

**The practice of PNC**

**Moderator:** How early do women go for PNC? Why do they go at that time? Why earlier or later?

**D1-** she will return after 45 days. Then she again will return after 3 months. And the last vaccination is in the 9^th^ month.

**Moderator:** Do women think skilled attendance during postpartum help their babies and themselves?

**D9-**they give her fafa for the mother and plumy nut for the baby if it’s necessary. They will follow the growth of the baby. The baby also gets vaccination for polio “lanshshawannoki gede". They advise the mother to initiate additional meals after the sixth month.

**D3-** besides the shortage of the drug all services are good.

**Traditional practices during pregnancy**

**Moderator:** Can you tell us about the traditional practices and beliefs during the postpartum period?

**D3-**previously all newborn babies were given "Amesa" but nowadays the practice is stopped after the provision of health education. Currently, only breast milk is given until the 6^th^ month.

**D4-** now no one is practicing these things.

**D6-** previously we will cut the tonsils of our newborns but the practice stopped. The one who cut will be arrested. Now we took to the HC when the baby has tonsillitis.

**D9-** previously we gave our babies “fiancho” drinking which is prepared from herbs.

**D1-**previously there is a high number of child mortality but now child mortality is history in our vicinity.

**Moderator:** How do you see community volunteers/TBAs and health professionals and maternal health services provided to the community?

**D4-** immunization service is given to children. HEWs and HDAs knew all mothers who gave birth and follow them collaboratively.

**Moderator:** In your opinion, what should be improved regarding PNC services? Continuum of care?

**D2-**the service is very good and we always thank our government for the service.

**D8-** previously some children died of malnutrition but now we can prevent this through the service given by the health facilities.

**D9-** the government must improve the availability of drugs in the facilities.

**FGD: 04**

**Antepartum for all participants**

**The practice of ANC**

**Moderator:** how early do women go for ANC? Why do they go at that time? Why earlier or later?

**D2-** Beginning in the fourth month, she will begin receiving follow-up care and shots. She will then make a return after eight months. Finally, if her delivery time arrives, she will return. Also, she will be evaluated to see if she has any health issues. She will come back for vaccination within two months of giving birth.

**D5-** When the mother begins the follow-up after six months; we could say it is too late. She will be at risk for a variety of health issues because she started her follow-up late. When the mother gives birth at HC; she will be happy and healthy and the baby who was born at the HC was healthier than the ones that were born at home. For the sake of the mother and the baby, delivering at the health facility is crucial.

**Moderator:** How often do they go to ANC?

**D3-** one pregnant mother could visit two or three times during her pregnancy period however if she got any unusual symptoms or the position of the baby is not good she could go to the health center anytime.

**D7-**she will start the follow-up in the fourth month and could go two times before the delivery. If she has bleeding or when the baby stops movement, she has to contact the healthcare workers immediately.

**Reasons for the use of ANC**

**Moderator:** Explain factors that would motivate women to utilize ANC services during their pregnancy.

**D6-** The expectant mother will have her and the baby's health monitored, as well as the baby's position, and receive health information about how to feed and sleep in the facility. The healthcare providers will also educate her on what to expect during childbirth and provide prenatal classes to help prepare her for labor, delivery, and postpartum care. In the case of any complications or risks, the expectant mother may be referred to a hospital of care to ensure the best possible outcome for both her and the baby. The HDAs will also work closely with the mother to develop a personalized birth plan that meets her individual needs and preferences.

**D2-** During her follow-up, her blood and urine will be examined. This is to assess any changes or potential issues that may arise in her health. It is also important for detecting and preventing any future health concerns. She will get drugs for anemia freely, and this makes her want to follow through with her ANC visit.

**Barriers to attending ANC use**

**Moderator:** If women do not go for ANC, what are their reasons?

**D5-** Due to a lack of information, they will not attend ANC follow-up appointments. They were not made aware of the significance of ANC follow-up visits or the advantages they may have for both their health and the health of their unborn child. In addition, they might not have a way to get to the appointments or someone to watch their other kids while they are gone. For instance, they believe that ANC follow-up exposes women's private lives and lacks privacy. If the mother misses the ANC follow-up, she will have a lot of issues.

**D1-**Before, some mothers skipped the ANC checkup; at the moment, the HEWs in the kebele are aware of how many pregnant women there are and which of them is carrying a child. As a result, each mother will receive health education before beginning ANC follow-up. All pregnant women currently receive follow-up care up until the time of delivery; no mother will give birth without it.

**D7-** They might have transportation issues because the HP may be far from their home. The HEWs regularly follow up with HDAs. There will be a transportation issue when labor begins at night, which may force the mother to deliver the baby at home. During their house-to-house visits, the HDAs and HEWs raise awareness of the value of delivering the HC.

**Reasons for discontinuation across the continuum**

**Moderator:** Why do women go to the facility for first ANC, but discontinue for subsequent ANC visits?

**D8-** The reasons for stopping ANC follow-up could be due to that most pregnant mothers have financial constraints and also few mothers have a lack of understanding about the importance of continued care during pregnancy.

**D2-** Mothers used to give birth at home due to a lack of knowledge, but thanks to the HDAs and HEWs tireless work, all mothers now give birth in hospitals. There are waiting rooms now, and expectant mothers are using them well in advance of the five-day delivery window. The waiting room itself raises awareness in the neighborhood when the mother remains there and gives birth without incident.

**D3-** generally most of the mothers stop their follow-up due to lack of transportation or financial problem. However, those mothers who discontinue the follow-up will return with the help of the HDAs.

**Traditional practices during pregnancy**

**Moderator:** Can you tell us about the traditional practices and beliefs during pregnancy?

**D4-** They would shuffle her by holding for two if the mother gave birth at home and there was a failed placental removal. They will make the mother drink "soicho" if the labor is prolonged. Currently, since all mothers gave birth to their babies at the health center, the tradition has vanished.

**D2-** I was advised to give birth in an HC while I was pregnant because of my blood group compatibility. I talked to my friend about it, and she admitted that she shared with me her experience of giving birth at the HC which is very good and I took her suggestion and gave birth in the health facility.

**D6-** previously calling the traditional birth attendant "Ogette" is common for home delivery which is the main traditional practice in our vicinity. Now all mothers will immediately go to the health facility when labor starts.

**Moderator:** How do you see community volunteers/TBAs and health professionals and maternal health services provided to the community?

**D5-** The HEWs and the health professional from the health center train the HDAs to make home visits to all villages' pregnant women and instruct them on proper prenatal care and nutrition. They also ensure that pregnant women receive regular check-ups and provide education for safe childbirth. The HDAs will ask the HEWs to send an ambulance when a pregnant woman goes into labor. HDAs follow each pregnant woman separately until it is time for delivery. This coordinated work has helped reduce maternal mortality in our kebele.

**D1-** The healthcare professionals will direct the HDAs to locate pregnant mothers in their village. They are crucial to the service because they identify pregnant women, undernourished mothers, and kids who are ill or undernourished during home visits. To get food and other medications, they also connect the identified mothers and kids with the health center. To obtain family planning services within 45 days of delivery, mothers can benefit from the HDAs.

**For recently delivered mothers only**

**Moderator:** How do you rate the quality of care you received during ANC follow-up? What kinds of services do you receive in ANC? Are you satisfied?

**D1-** At this medical facility, I previously finished my follow-up and gave birth to my child. The service providers are available and deliver at six o'clock when I arrive at the health center in labor at three o'clock. They were sympathetic to my plight, gave me excellent service while standing for a long time, and helped me deliver safely. Only two healthcare professionals attended the delivery; no other people were admitted, but other healthcare professionals were admitted without restriction. Additionally, the blanket they gave me was not very clean. The health center is extremely unclean, and the mosquitoes in the waiting area are a problem. Several mothers left early because of this issue.

**D6-** There is currently no issue, and the HC staff is advising us. Families with pregnant mothers who gave birth at home are regarded as odd. Although our parents did not lead good lives in terms of health services, we now lead healthy lives thanks to our government.

**Moderator:** If the mother received ANC; Ask: Explain factors that motivate you to utilize ANC service in their pregnancy

**D2-** Continuous health education is provided by HDAs for facility delivery, follow-up, and initiating programs. They preach that you risk developing health issues if you don't listen to them and get vaccinated. Twice a month, we have a forum for expectant mothers. They are educating people about using personal hygiene products and healthcare services. By contrasting the health of an immunized child with that of an unimmunized child, they also instruct us to vaccinate our kids. Along with the HDAs, the HEWs and healthcare professionals working in the Woreda office are also educating the community. Although they offer good service, the facility's accessibility due to the distance and lack of electricity is the main issue. The importance of the health service outweighs that of the educational service.

**D9-** Due to my lack of knowledge, I had to give birth to my first and second child at home. However, after developing a bleeding problem in the second delivery, I advised everyone to have their babies at the HC. Maternal mortality used to be very common, but it is now completely absent. Pregnant women are drawn to the service itself.

**D5-** the service delivered by the health professional and the follow-up done by the HDAs make most pregnant mothers start follow-up.

**Moderator:** Explain to us your experiences relating to the utilization of ANC care provided by skilled birth attendants.

**D5-**They will check to see if I have anemia and blood pressure during my ANC follow-up. However most of the time the health center does not have the medication, they will force us to purchase it from a private pharmacy, which causes us problems since the price of the drug in the private pharmacies are expensive. Moreover, a woman attending a delivery was given FAFA, soap, a bed net, and clothing for the newborn, but right now, she is just sent home without anything.

**D2-** HEWs provide us with high-quality care during ANC follow-up, but health center staff does not provide a comparable level of care during ANC and delivery. The healthcare provided by the staff at the health center is not satisfactory, nor is the service. I brought my pregnant neighbor with me, and they only checked her out with hearing aid supplies before telling us to go to the private clinics because the medical staff wasn't doing their jobs well. Healthcare professionals schedule appointments for a week without checking on the mother, which causes mothers to stop following up and delivering at home. At the level of the health center, pregnant mothers receive incredibly subpar care.

**D4-** When a pregnant woman visits HC, it can be difficult to obtain services, let alone a service card, but at HP with HEWs, things go more smoothly. The laboratory also has a significant issue with urine tests; typically, they claim that there is no chemical available to conduct the tests. They only test our blood; they don't have any tests for STIs. They also don't test our urine or stool. During the follow-up, they only perform pregnancy tests and nothing else. The availability of medications is another issue; they typically prescribe medicines to independent pharmacies.

**D1-** the HC is not providing good service to pregnant mothers, as they prescribe expensive drugs and Amoxicillin is not available, making it difficult for them to afford transport and food.

**D2-** Before this, all expectant moms received free bed nets; however, this has not been the case for the past three years. As a result, malaria affects a lot of expectant mothers. Most diseases have a high-risk factor for pregnant women. As we haven't received a bed net in the last five years, the government needs to acknowledge our issue and provide for us quickly.

**Moderator:** In your opinion, what should be improved regarding ANC services?

**D5:** sometimes if a woman had labor and the motorcyclist wouldn't transport the pregnant mother for the regular price, so we have to collect money and paid for it. This indicates that the main issue preventing the use of the delivery service is the transportation issue brought on by the need to travel a great distance to the institution.

**D7:** The availability of the ambulance is the issue. The mother might give birth in the ambulance if it is running late. The drivers have a problem with attitude. This situation calls for urgent action to improve the ambulance service, particularly in terms of response time. Additionally, driver training and proper selection procedures may address the issue of driver attitudes and behavior toward patients.

**The practice of facility delivery**

**Moderator:** Do women think skilled attendance during childbirth helps themselves and their babies?

**D2-** they provide good service. Nevertheless, there is room for improvement in terms of customer communication and response times. Additionally, some of the mothers have complained about their displeasure with certain facets of the service, like billing or equipment upkeep. The service has the potential to improve even further overall. Her blood pressure will be checked, and they'll stop any excessive bleeding. They will examine the infant as part of their good service.

**D7-** When they give birth in a medical facility, there is no bleeding; however, at home, there will be significant bleeding. An air according to speculation may hit her. Therefore, if she gave birth at HC, there would be no postpartum bleeding and both the mother and the child would be healthy.

**D1:** To ensure the safety and health of the mother and child, healthcare providers at the HC can offer additional support and interventions. When a laboring mother's problems exceed their ability to handle them, they refer her to the hospital.

**Barriers to attending facility delivery**

**Moderator:** If women deliver at home, what are their reasons? Explain the constraints that influenced women to utilize facility delivery services.

**D4-** Children born in a health facility are healthier. Currently not only the mothers the fathers are well adapted to health facility delivery. The main reasons for giving birth at home are poor knowledge of the household members. Few families also claim that because our parents give birth at home, we must as well.

**D1-** Delivering health services has a major transportation and road infrastructure problem. Many pregnant mothers who require medical care might not be able to access it promptly without an adequate transportation system and road infrastructure. This sometimes can lead to home delivery and improve overall health outcomes. In addition, there is only one ambulance available to our woreda.

**D2-** the mothers usually forget their expected day of delivery and give birth at home sometimes. This can result in increased risks for both the mother and the baby, as transportation for medical assistance may not be immediately available. The HDAs are working hard to remember all mothers in their village and keep track of their due dates to ensure safe delivery.

**Traditional practices during the intrapartum period**

**Moderator:** Can you tell us about the traditional practices and beliefs during childbirth?

**D3-** During labor, they would cover the mother with a blanket, but as community perception and understanding of childbirth improved; mothers began giving birth at hospitals. The community used to believe that the baby would suffer bad luck if the umbilical cord was buried there when they were delivered at the hospital, but that belief is no longer acceptable.

**D5:** Traditionally there is a community belief that a woman who is in labor must hide from people and will be covered with blankets during the labor.

**Moderator:** How do you rate the quality of care you received from the facility during childbirth?

**D1-** the HWs at the health center give the mother private rooms while she is giving birth. Labor and delivery are attended by just one medical professional and one relative from the mother's side.

**D9-** only the service provider and the laboring mother will enter the room. They will wash the mother after the delivery. The service is very good in general.

**D7-** we delivered at home because of a lack of knowledge due to the unavailability of health education providers at the time. Previously the delivery service provided at the HC is not good enough now they are giving quality service. They give every laboring mother intravenous glucose which is very helpful for her health. However, we are not getting bed net so the government should see our problem and provide us with it soon.

**D5-** the HC is providing excellent service; they are doing a great job. I hope they continue to have this good job always because of their admirable commitment to providing high-quality care especially the hard work of the HDAs and HEWs are excellent. When the mother's condition is too severe for them to handle, they refer her to Yirba Hospital.

**D4-** the mothers were instructed to exclusively breastfeed their children up to the sixth month and to give them additional meals after that.

**Moderator:** Explain to us your experiences relating to the utilization of facility delivery care provided by skilled birth attendants.

**D2-** The community's beliefs have changed in modern times. Every government strategy is well received by the community. The HC serves as a link between the state and the populace. Because the mother is given wheat flour there, every expectant mother wants to give birth there.

**D2-** Everyone in the community, especially pregnant mothers, is pleased with the care that is being given by the health facilities. Pregnant women have expressed particular gratitude for this, saying that they felt safe and supported throughout.

**The practice of PNC**

**Moderator:** How early do women go for PNC? Why do they go at that time? Why earlier or later?

**D8-** We typically visit the HF for child immunizations and family planning services after 45 days. Then, I will then come back to the appointment set for my baby's immunization program.

**D2-** If the baby remains healthy and symptom-free; the mother may have to continue staying away until the 45th day. However, if the baby's condition worsens before then, the mother may have to return earlier.

**D1:** She will leave after 45 days. They will be reappointed for one month following the vaccination of the mother and the child.

**Moderator:** Do women think skilled attendance during postpartum help their babies and themselves?

**D3-** the healthcare providers teach us how to wash our babies and breastfeed them. Also, they taught us to return if the baby has a fever, diarrhea, or malaria.

**D9-** they will give us HE regarding complementary feeding. This guidance can help prevent malnutrition and ensure proper growth and development. This information can be valuable when deciding which complementary foods to offer.

**Moderator:** Explain factors that would motivate women to utilize PNC services in their childbirth.

**D3-** The reason why we come for the PNC is not due to the encouragement of the health workers rather we return for the sake of our and the babies' health. Their service did not encourage service utilization at all. HEWs workers encourage us to deliver at the HF, and not to feed other foods before the child reaches six months. They also taught us how to properly breastfeed our children. Generally, only the HEWs encourage us to utilize health services.

**D8-** nowadays all pregnant mothers give birth at health facilities.

**D9-** If necessary, they give her FAFA for the mother and plumy nut for the child. They will monitor the infant's development. The infant also receives the vaccine for children's diseases. After the sixth month, they advise the mother to start new meals.

**Traditional practices during pregnancy**

**Moderator:** Can you tell us about the traditional practices and beliefs during the postpartum period?

**D4-** Previously, the community would give newborns "Amessa," but now that we have received health education, we only provide breast milk up until the sixth month. As of right now, this practice has completely ceased. The community's awareness changed after the government repeatedly provided health education.

**D5-** When the baby develops an illness, society believes it will go away on its own because it is unhealthy to expose the mother and the baby to the environment after delivery. This notion gradually faded, though, and today all new mothers and their babies visit a medical facility for treatment or follow-up.

**Moderator:** How do you see community volunteers/TBAs and health professionals and maternal health services provided to the community?

**D9:** HEWs and HDAs are collaborating effectively. The HEWs provided training for HDAs to recognize malnourished patients, and the HDAs recognized mothers and reported to the HEWs for care. All mothers and children will be measured by the HEWs who train HDAs in anthropometric measurement; if they are found to be malnourished, they will be taken to the health center for treatment.

**D7-** After delivery, HDAs always visit the mother at home, and if the mother has any issues, they will discuss them with the HEWs and have them resolved. Additionally, HDAs are providing the mother with education on infant feeding practices. This personalized support by HDAs is greatly improving the health outcomes of both the mother and her baby.

**For recently delivered mothers only**

**Moderator:** How do you rate the quality of care you received during PNC follow-up? What kinds of services do you receive at PNC? Are you satisfied?

**D4 -** although the health institutions' post-natal care is provided satisfactorily, there are times when essential medications are not available. To guarantee the best possible care for post-natal patients, the health center must enhance its drug supply. The healthcare professionals are ready to help.

**For recently delivered mothers only**

**Moderator:** In your opinion, what should be improved regarding PNC services? Continuum of care?

**D1-** We may postpone going to the hospital if our health is good up until the 45th day after delivery. HEWs come to the health post late. Family planning services are also provided after a long waiting period, which is difficult for many mothers and may put the mother at risk for unintended pregnancy. All services have lengthy wait times.

**D8-** Despite the supply shortage, the service is excellent, and we are grateful to our government for it.

**D5-** Family planning supplies have been scarce particularly Depo for over a month. The mother could be exposed to an unwanted pregnancy when there is no Depo at the HP we are required to use at private clinics.

**FGD: 05**

**Antepartum for all participants**

**The practice of ANC**

**Moderator:** How early do women go for ANC? Why do they go at that time? Why earlier or later?

**D2-** they will start the follow-up in the sixth month.

**D4-** the pregnant mothers have to start in the fourth month. The health professional will check the health of the baby and the health of the mother, they will check whether the baby is moving or not, and also she will take vaccination during the follow-up.

**D3-** the HEWs and health workers will advise the mother to plan for health facility delivery to prevent excessive bleeding during home delivery. By taking the health education provided by the health workers during the ANC follow up the community significantly decreased maternal mortality.

**Moderator:** How often do they go to ANC? Why do they go at that time?

**D5-** They will go for a follow-up in the sixth month. During the follow up the mother and the baby's health will be checked. After the mother reaches six months of pregnancy she must not do hard work, she has to have extended rest, and other household members have to support her.

**D6-** after she started her follow in the sixth month, they will check for the health of the mother and the position of the baby. The mother will return in the 9^th^ month for getting her vaccination. When the labor start we will call for an ambulance the ambulance will come with the health worker and they will take her to the health facility for delivery. During delivery, the health professionals will continuously follow the mother and baby till she gave birth peacefully. Previously all mothers give birth at home, and the babies usually will be ill, and they even might die. Forty-five days after delivery the mother and the newborn will return to the health facility for immunization “lanshawiho" and other illnesses. Thanks to our government, our children are healthy and they are peaceful.

**D6-** previously pregnant mother should have four visits but now she has to have eight visits. The mothers have to start the ANC follow-up in the 16^th^ week. She will be provided the drug "iron" for 28 days to prevent anemia, in addition, she will take vaccination. During the follow up besides the mother's health the position, presentation, and heartbeat "Wodana ganani nosi" of the baby will be checked. Generally, all-around examinations and checkups will be done regularly. As I said earlier, the follow-up will be conducted starting from the 16^th^ week.

**Moderator:** Do women think skilled attendance during pregnancy helps their pregnancy?

**D7-** for pregnant mothers HDAs are very supportive and they prove them with a very good health education. The HDAs always advise the mother to take the vaccine and the mother accepts the advice and they will be vaccinated based on their appointment. When the mother gets vaccination during her pregnancy, she will give birth to a healthy baby, which makes the father and mother happy. It seems easy when we talk about it but it is very important. HEWs and HDAs are doing a great job in terms of health education in our kebele that is helping us keep our health good.

Nowadays everybody in our kebele is taking seriously the health education provided by the HEWs. Especially pregnant mothers always accepting, and following the direction given by the HEWs, they are getting their vaccination accordingly. Those pregnant mothers who cannot read are asking their husbands to check for appointment dates on the card. The community is accepting the education provided by the HEWs and the government has to make sure that service provided is continuous and quality. Generally, all healthcare workers are giving a good service in our kebele.

**Reasons for the use of ANC**

**Moderator:** Explain factors that would motivate women to utilize ANC services during their pregnancy.

**D5-** we have HPs around which the mother goes. At the HP they will examine the mother's abdomen to check for the conditions. They will teach the mother to have adequate rest, and continue the follow-up. As said earlier they will check the position and condition of the baby since "qaqqu amatte gido xiwamiro ama ledo xiwantana”. They will also advise them to eat nutritious food “maxaxante sagale”. In addition, they are giving flour to make porridge for pregnant and lactating mothers.

**D8-** the previous health education offered by the HDAs will motivate the expectant mother to begin the follow-up. This is essential to make sure that any problems are found early and can be fixed right away, lowering the possibility of complications. Additionally, routine examinations offer a chance to track the baby's development and growth, ensuring their general health and well-being.

**Barriers to attending ANC use**

**Moderator:** If women do not go for ANC, what are their reasons?

**D5-** The main reason the pregnant mother did not begin her follow-up was a lack of awareness. She has no knowledge about the importance of regular check-ups during pregnancy and did not know about available healthcare services.

**D3-** lack of transportation and financial problems can both pose significant barriers to accessing antenatal care. These issues usually prevent mothers from attending appointments, receiving necessary tests, and getting the support they need throughout their pregnancy.

**D1-** access to prenatal care may be significantly hampered by the service provider's possible presence at the health post. This is especially concerning for expectant women traveling from remote areas where there may not be many prenatal care providers available.

**Reasons for discontinuation across the continuum**

**Moderator:** Why do women go to the facility for first ANC, but discontinue for subsequent ANC visits?

**D2-** most of our neighbor's mothers discontinue due to long waiting times at the health facility. Moreover, the financial problem usually hinders an obstacle to attending their appointment.

**D9-** lack of awareness or bad health care providers could make the mother discontinue the follow-up.

**D4-** the decision to stop was made out of ignorance or reluctance. To allay these worries, HDAs will offer thorough education on the advantages of prenatal care and the dangers of stopping it. Additionally, they will provide resources and support to help allay the mothers' fears and uncertainties.

**D7-** sometimes the services rendered are the reason that pregnant mothers are no longer followed up with; other times, they will berate us. To prevent complaints, healthcare providers must place a high priority on providing quality care and acting professionally.

**Traditional practices during pregnancy**

**Moderator:** Can you tell us about the traditional practices and beliefs during pregnancy?

**D3-** the pregnant woman's abdomen will be massaged by the traditional birth attendants. The practice of abdominal massage during pregnancy is based on traditional beliefs. Given the availability of safe and effective medical interventions, its potential for harm to both the mother and the unborn child makes it an outdated practice that needs to be avoided.

**D1-**as a result of the education they (TBAs) received regarding the importance of health facility delivery. They are "ogette" and currently advise expectant mothers to go to a health facility.

**D5-** currently the traditional practice stopped since the HEWs and HDAs are working on a follow-up with all pregnant mothers in our kebele.

**D7-** the traditional birth attendant took training and they are working with the HDAs.

**Moderator:** How do you see community volunteers/TBAs and health professionals and maternal health services provided to the community?

**D3-** HDAs will follow the pregnant mother at home they will encourage her to start the follow-up early. HEWs also provide education on proper prenatal care, monitor her health, and ensure any necessary medical interventions are taken.

**D9-** they are helping us a lot. The quality of the antenatal care the health worker is providing is very good. We are grateful for their efforts in providing us with excellent antenatal care. The health worker's care and attention have made us feel very comfortable during this time.

**D3-** the government has to encourage the HDAs by giving incentives for their effort.

**For recently delivered mothers only**

**Moderator:** How do you rate the quality of care you received during ANC follow-up? What kinds of services do you receive in ANC? Are you satisfied?

**D8-** the HEWs are doing a very good job. But they are living in Hawassa and they sometimes come late.

**D6-** In the health center some of the service providers have attitude problems towards the mother from rural areas.

**D1-** all professionals are giving us a good service.

**D7**- they are giving satisfactory service, especially HDAs and HEWs.

**Intrapartum for all participants**

**The practice of facility delivery**

**Moderator:** Do women think skilled attendance during childbirth helps themselves and their babies?

**D9-** when they give birth at the health institution, there is no bleeding, but when they give birth at home, they will have heavy bleeding. It is believed that she has "Gobarichchi kision". Therefore, if she is delivered at HC, there is no bleeding after delivery, and both the baby and the mother will be healthy.

**D2-** the mother’s blood pressure and urine will be checked. They will also address any worries she may have and make sure she is comfortable throughout the procedure. They will examine the infant as part of their good service.

**D5-** if the mother has anemia they will give her medication and will also educate her on birth symptoms, and the place to deliver. They will help her give birth when she reaches that point.

**D1-** currently all mothers knew the importance of health facility delivery and every mother will follow by her one to five team leaders.

**Reasons for use of facility delivery**

**Moderator:** Explain factors that would motivate women to utilize delivery services during their pregnancy.

**D8-** since the ambulance is coming fast for the service; this is making the mother use the delivery service. The mother likely feels that it is safer to have the baby delivered through the service rather than risk waiting for the ambulance. Additionally, having a trained professional assist with the birth can provide added reassurance and support.

**D5-** currently the HDAS is encouraging and following all pregnant mothers in our village to plan their delivery. Nowadays all pregnant mothers are delivering at the health facility.

**D1-** previously several mothers died when giving birth at home this experience made our community aware of facility delivery.

**D9-** the quality of the service during ANC follow-up provided by the health extension workers is also making the mother give birth at the health center.

**D8-** at the moment, pregnant women are pressuring each other to use the delivery service, as all mothers are aware. They advise me to arrive at the facility early.

**Barriers to attending facility delivery**

**Moderator:** If women deliver at home, what are their reasons? Explain the constraints that influenced women to utilize facility delivery services.

**D9-** mothers give birth at home because going to the hospital is expensive and there isn't enough food there. This emphasizes the demand for more readily available, reasonably priced transportation and better food options in healthcare facilities. Typically, it has to do with money issues.

**D1-** When the labor begins at midnight, the cost of transportation will increase three times, costing each person up to one hundred birds, and the majority of expectant mothers even lack money. Given that our kebele is the farthest from the facility and that the cost of food while waiting in the waiting room is also very high for most of us, women give birth at home. Along with the distance, the HP's lack of water and electricity forces the mother to deliver at home.

**D4-** Previously mothers who gave birth will not take showers causing poor personal hygiene but now all mothers will take baths after they gave birth which improves their hygiene. The mothers who gave birth at the health facility have good hygiene "su’nitino, biffino”.

**D7-** Mothers who have many children usually deliver because their labor is quick and we may not be able to reach the facility. Moreover, elder mothers will become shy and reluctant to give birth at the same place their daughter was delivered.

**D6-** The absence of an ambulance driver's phone is another factor, in addition to the distance. Due to this, the mother must deliver her child at home with a traditional birth attendant.

**Reasons for discontinuation across the continuum**

**Moderator:** Why do women go to the facility for ANC, yet mostly deliver at home?

**D6-** most of the mothers who follow ANC will deliver an HC, if the mother discontinues the HDAs will make them return and deliver at HC.

**D7-** the mother chooses to give birth at home due to the HF's lengthy travel distance and lack of transportation. Sometimes the mother has to give birth at home due to the inadequate care and uncleanliness of the HF.

**D3-** sometimes the attitude of the service providers could make the mothers deliver at home, they will insult the mother who previously gave birth at the health center.

**Traditional practices during the intrapartum period**

**Moderator:** Can you tell us about the traditional practices and beliefs during childbirth?

**D2-** the mother believes a new air/wind is bad for the laboring mother. They also say that male workers deliver the baby which makes them fearful. Delivering at home will expose the mother to excessive bleeding after delivery.

**D5-**Traditionally, the laboring mother has to hide from others, and they also cover her with four or five blankets, distressing her. When she gives birth at home, she might die, and the baby could also die.

**D4-** usually when the mother gives birth at home they could die since it is done by the traditional attendants. When the mother delivers at home, the hygiene is not good, putting the mother and the newborn at risk.

**Moderator:** How do you see community volunteers/TBAs and health professionals and maternal health services provided to the community?

**D2-** HEWs and HDAs collaborate to make the mothers give birth at the HC. While the healthcare professionals are providing good service, the HDAs support mothers starting from conception.

**D9-** the HDAs will encourage mothers to deliver at the HC and will accompany and counsel them. The rates of maternal and neonatal mortality will eventually go down as a result of this. Healthcare professionals will also be able to give new mothers essential care and education.

**For recently delivered mothers only**

**Moderator:** How do you rate the quality of care you received from the facility during childbirth?

**D3-** when we go to the health center for delivery most of the health care providers are compassionate. The service is satisfactory.

**D8-** besides a few health professionals with poor attitudes the service is somewhat moderate.

**D1-** the service is medium satisfactory.

**D4-** they are giving well services especially the HDAs and HEWs are very hard in our village.

**D5-** the service is good. We thank our government for this.

**Moderator:** In your opinion, what should be improved regarding facility delivery services? continuity of care?

**D1-** there needs to be an improvement in the availability of medications and the cleanliness of the delivery room. To ensure the safety and well-being of the mothers and the newborn, it is crucial to give these issues a top priority and to take action on them.

**D6:** The ambulance service has a poor reputation because it does not offer a return service for newly delivered mothers. This lack of service has been known to cause significant stress and inconvenience for new mothers who need to seek emergency medical attention.

**D2-** the head of the health center has to follow the health workers' timing and the availability of water during delivery.

**D9-** when the HDAs call the ambulance driver they do not answer the phone. Therefore, we must use another mode of transportation like a motorbike or cart to bring the mother.

**Postpartum for all participants**

**The practice of PNC**

**Moderator:** How early do women go for PNC? Why do they go at that time? Why earlier or later?

**D3-** the mother and the baby will come back on the 45th day for immunization and family planning service, and they will come back every month until the vaccination is completed.

**D5-** on the 45th day, she will return. The mother and child will be scheduled again for the following month after receiving their vaccinations.

**D7-** we usually go to the HF after 45 days for child immunization and use family planning services. Afterward, I will return based on the appointment given for my baby's immunization program.

**Moderator:** How often do they go to PNC? Why do they go at that time?

**D2-** the mother would be considered late if she arrived after the 45th day; however, if she arrived on the 45th day, she was on time. The immunization and other services may be ineffective if she arrives after 45 days.

**D9:** the postnatal follow-up will begin on the 45th day, and they will be given another appointment to return in 28 days. Depo injection may occasionally not be available, forcing us to seek treatment at private clinics. When this occurs, the mothers begin to doubt the availability of family planning drugs and eventually discontinue the postpartum checkups. The main issue following delivery is a lack of family-planning medications.

**Reasons for use of PNC**

**Moderator:** Explain factors that would motivate women to utilize PNC services in their childbirth.

**D4-** getting the vaccination for our children is the main reason why I return. Moreover, I will get a family planning injection.

**D8-** The reason why we come for the PNC is not due to the encouragement of the health workers rather we return for the sake of our and the baby's health. Their service did not encourage service utilization at all.

**D9-** the HDAs in our one-to-five team encourage the whole mothers, not only encourage they sometime will take the mother with them.

**D6-** HEWs workers encourage us to deliver at the HF, and not to feed other foods before the child reaches six months. They also taught us how to properly breastfeed our children. Generally, only the HEWs encourage us to utilize health services.

**Barriers to attending PNC use**

**Moderator:** What are the barriers to accessing PNC?

**D2-** there are areas in our kebele that are inaccessible for transportation and the ambulance does not come to the HP but usually they refuse to come unless the HEW calls them. There is a huge transportation problem for pregnant mothers.

**D5-** if the mothers have no husband she might fear using the service due to the social stigma. In addition, she might have financial problems.

**D7-** in addition to the above reasons lack of knowledge can also be a barrier.

**D3-** most of the time lack of awareness about the service is the main reason why the mothers did not use the service.

**Traditional practices during pregnancy**

**Moderator:** Can you tell us about the traditional practices and beliefs during the postpartum period?

**D6-** Traditionally the traditional healers feed the baby herbs “Amessa”, but the practice significantly decreased, few of the mothers are giving it after the health education provided by the health extension workers.

**D2-** the community thought when the baby was exposed to another person's eye in its early days; it might be exposed to an evil eye.

**D1-** few mothers fear that exposing the newborn to light and wind during transport to the health facility is bad for the newborn's health.

**D2-** Before stopping the practice, we used to cut our newborns' tonsils. When the baby has tonsillitis, we currently take him to the HC for medication.

**D9-** Previously, we gave our babies herbal drinking, which is prepared by traditional healers. However, society later learned that it was not advisable to give infants.

**D1-** Previously, there was a high rate of child illness and death, but this is no longer an issue in our area after education.

**9I-** How do you see community volunteers/TBAs and health professionals and maternal health services provided to the community?

**D7-** the HDAs are encouraging the mother and the families to start follow-up after delivery. In addition, they are giving health education concerning child feeding, and personal hygiene.

**D9-** HEWs, HDAs, and other health care professionals will instruct mothers on child feeding and hygiene.

**D6-** the providers of health services will conduct health checks, administer vaccinations, and offer family planning services. Furthermore, they may provide counseling for severe symptoms of childhood illness. Health professionals may also advise on healthy ways of preparing porridge and baby washing techniques.

**Reasons for discontinuation across the continuum**

**Moderator:** Why do women go to the delivery at the facility, yet most don't receive PNC?

**D2-** the mothers will not come for post-natal care due to a lack of knowledge by the mother.

**D5-** since the baby needs vaccination all mothers come for the service.

**D6-** HDAs will not let the mother discontinue, the mother will be asked by the kebele management if she failed to vaccinate her child.

**D8-** all mothers are coming for the service.

**Traditional practices during pregnancy**

**Moderator:** Can you tell us about the traditional practices and beliefs during the postpartum period?

**D9-** Previously, we gave "Amesa" to the baby, which is a harmful practice; now no mother is giving it except a few mothers who are giving it secretly.

**D2-**related cutting of the uvula was also practiced previously.

**D5-** In addition, the traditional birth attendant discards the clostridium; however, after the health education provided by the health workers, it is now given to the baby.

**Moderator:** In your opinion, what should be improved regarding PNC services? Continuum of care?

**D2-**the service is very good and we always thank our government for the service.

**D9-** the government must improve the availability of drugs in the facilities.

**D6-** No medication is present in the health facility; all required medications must be available. We are being compelled to purchase from independent pharmacies. Additionally, it is very challenging for laboring mothers when there is only one ambulance available, when the ambulance called two or three kebeles simultaneously it will be difficult.

**D3-** I want to thank all health professionals; they are doing a great job. I will pray for them.

**D1-** availability of Depo injections must be improved, and the health workers have to give the family planning drug we wanted. They are forcing us to use 5-year or 12-year medicine.

**FGD: 06**

**Antepartum for all participants**

1. **The practice of ANC**

**Moderator:** How early do women go for ANC? Why do they go at that time? Why earlier or later?

**D9.** The pregnant mother will begin visiting the health facility at 2 months of pregnancy to get ANC follow up including TAT vaccination.

**D2.** The pregnant mother will visit the first ANC follow at health at 2 months.

**D8**.pregant mother will visit a health facility after 2 months when she felt symptoms of pregnancy.

**D5.** Pregnant mothers should get the vaccination at 2 months and 8 months respectively.

**D1.**In general, she will visit a health facility to follow up on her health and fetus.

**D6.**After the first visit, she gets an appointment in order checkup her till the delivery time at the health center.

**D8.** During visiting time we get pills for increments of blood.

**D4.** They give written appointment for us.

**D1**.they provides health education on personal hygiene for both her and the child after born. In addition, they provide us with health education on complementary food for newborn children.

**Moderator:** Why earlier or later?

D6. Every mother regularly follows up on Tuesday of the week. When we faced any challenge we should visit and get medicines to tackle challenges.

**D1.** Pregnants should get help from health care workers when their fetus is not moving in the uterus.

**Moderator:** How often do they go to ANC? **Probe** why do they go at that time?

**D8.** The previous 3 times those pregnant mothers visit a health facility. Currently, at least 6 times should visit a health facility to get ANC services.

**Moderator:** Do women think skilled attendance during pregnancy helps their pregnancy?

**D1.** Yes, they get help like food FAFA for pregnant mothers and Plumpy Nut for children after six months from the facility.

**D5.** We get food like FAFA for pregnant mothers from the health facility. Also, we get Plumpy nuts for our kids after anthropometric measurements.

**D3.** They give FAFA once in two weeks for pregnant mothers and Plumpy Nut for our kids.

1. **Reasons for the use of ANC**

**Moderator:** Explain factors that would motivate women to utilize ANC services during their pregnancy

.

D9. The motivation factor is how we get services from the facility.

**D3.** HDA and health extension workers are motivating us to get services from a health facility.

**D6.** HDAs are motivating us to get services and health extensions and also give FAFA and Plumpy Nut.

1. **Barriers to attending ANC use**

**Moderator:** If women do not go for ANC, what are their reasons?

**D6.** Because of low socio-economic status, fear of their hygiene, and lack of wearing clothes.

**D7.** Because of the long distance, they prefer home delivery.

**D8.** To go health facility we might fear abdominal surgery or C/S due to this we prefer to give birth at home rather than at a health facility.

**Moderator:** Socio-cultural

**D6.** Previously most pregnant mothers shouldn't go to health facilities because their mother-in-law does not allow them to go to health facilities.

**Moderator:** Quality of care]

**D8.** There is no problem regarding the quality of care.

**D1**. Those who do not get FAFA because of their measurement are not interested to go health facilities.

1. **Reasons for discontinuation across the continuum**

**Moderator:** Why do women go to the facility for first ANC, but discontinue for subsequent ANC visits?

**D1.** Because of health care providers and inadequacy of measurement to get FAFA

**D7.** Because of the long waiting times at health facility

**Moderator:** Socio-cultural

**D6.** Multiparas are not wanted to visit health facilities due to shame or fear of their daughter-in-law when they get pregnant. But those young pregnant do not fear visiting a health facility.

1. **Traditional practices during pregnancy**

**Moderator:** Can you tell us about the traditional practices and beliefs during pregnancy?

**D6.** Not visiting health facilities.

**D3.** In our villages, there are no traditional practices currently.

**D6.** No skilled birth attendant traditionally around our kebele

**D1.** Currently, traditional birth attendants (TBAs) are advising pregnant mothers to visit a health facility.

1. **For recently delivered mothers only**

**Moderator:** How do you rate the quality of care you received during ANC follow-up? What kinds of services do you receive in ANC? Are you satisfied?

**D1.** We get very good services including vaccination and food FAFA

**D2.** Very good quality what we get from health facility

**D3.** We are happy to services

**Moderator:** Explain factors that motivate you to utilize ANC service in their pregnancy

**Moderator:** Food is a motivating factor, to cheek up our health syrup for kids and services.

**Moderator:** Explain to us your experiences relating to the utilization of ANC care provided by skilled birth attendants.

D1. I am not satisfied with their services because they are not respecting, care, and compassion for pregnant mothers. Also, they haven’t medical equipment even for checkups.

D3. We save our life by the health care provider

1. Community Perceptions about health providers and maternal health programs

**Moderator:** How do the communities see the maternal health programs and health professionals?

**D1.** Yes, very good services we get from the facility

**D6.** Very good places for our services including vaccination and treatments for kids

**D6.** They teach us about how we keep personal hygiene.

**Moderator:** What efforts has your community made to increase maternal health service in your community?

**D9**. Health care providers remind us to visit the facility at any time for our health.

**D3.** HDA and hews are corners for our follow-up.

**D6.** When the mother gets birth at home she gets “Miqa" which is blood in her uterus because of this, the pregnant mother not prefers home.

**D9.** Motivating factor that pregnant mother is health care providers

**D6.** We gave birth at home because healthcare providers are male and fearful but we prefer female healthcare providers to assist us.

**D7.** Healthcare providers do not keep our privacy, so we prefer home. And we believe that clothing with a blanket is prolonged labor at home.

**D2.** Healthcare providers simply cut by scissors our reproductive organs when they assist us.

**Intrapartum for all participants**

1. **The practice of facility delivery**

**Moderator:** Do women think skilled attendance during childbirth helps themselves and their babies?

**D2.** Our health needs to attend health facilities.

**D1.** Healthcare providers assist us during delivery time. Kids and mothers need to care.

**D5.** I am happy due to the services for me and my kid.

**Moderator:** Explain factors that would motivate women to utilize delivery services during their pregnancy.

**D1.**services we get from health facilities are motivating us including food, plumpy nuts even health care providers' advice

1. **Barriers to attending facility delivery**

**Moderator:** If women deliver at home, what are their reasons? Explain the constraints that influenced women to utilize facility delivery services.

**D3.** Suddenly, I got a blood show ''shafo dartu" at my home without any problem. I get delivered to home.

**Moderator:** Explain to us your experiences relating to the utilization of PNC care provided by skilled birth attendants. Prove for;

**D6.** After 45 days we get PNC services from a health facility

**D3.** Yes, it is important for mothers and children

**D6.** HEWs give education and community leaders, but those mothers who need PNC do not prefer the facility because there is an incentive at a health facility.

1. **Community Perceptions about health providers and maternal health programs**

**Moderator:** How do the communities see the maternal health programs and health professionals?

**D1**. They are providing food for all pregnant mothers, respecting pregnant, education for pregnant mothers, and quality services in the facility.

**Moderator:** What efforts has your community made to increase maternal health service in your community?

**D6.** Providing health education for those pregnant mothers by integrated health development army and health extension workers are the basis for increasing maternal health in the community.

**FGD: 07**

**Antepartum for all participants**

**The practice of ANC**

**Moderator:** How early do women go for ANC? Why do they go at that time? Why earlier or later?

**D4-** they start in the fifth month.

**D9-** they start in the third month. She will take pills for anemia and then she will continue till she gave birth.

**D6-** there is a problem in our ANC follow-up; they have an instrument for examination. They are referring to us simply so mothers are stopping the follow-up.

**Moderator:** How often do they go to ANC? Why do they go at that time?

**D1-** she will go three times. To prevent

**D7-** she will start her vaccination in the sixth month and she will return in the seventh and eighth month. In the ninth month, she will complete her vaccination and she will be back when the labor starts.

**Reasons for the use of ANC**

**Moderator:** Explain factors that would motivate women to utilize ANC services during their pregnancy.

**D9-** the neighbors will encourage her to start the follow-up. During labor, if she has the capability she will go to the HF by herself otherwise the ambulance will be called for her.

**D8-** the health education she took previously will encourage her to start the follow-up.

**D6-** previously many mothers suffer during labor and delivery. So this experience is making our mothers start the follow-up. Moreover, the quality service provided for other pregnant mothers attracts others to initiate the follow-up.

**D5-** previously several mothers experience excessive bleeding during delivery so to prevent this she will start the follow-up. The main motivating factor is to prevent excessive bleeding during delivery.

**Barriers to attending ANC use**

**Moderator:** If women do not go for ANC, what are their reasons?

**D3-** lack of awareness is the main reason plus few mothers are reluctant or negligent to start ANC follow-up.

**D7-** previously the HDAs are continues encouraging the pregnant mother to ANC follow up but now they decreased their effort and the mother is discontinuing/decreasing the follow-up. If the HDAs work as previously, no mother will be left behind.

**D8-** currently no one is encouraging our mothers to start to follow up and this is making the pregnant no to start the follow-up.

**Moderator:** What are the barriers to accessing ANC?

**D4-** we do not have HP in our kebele and this is the main barrier to accessing the follow-up.

**D1-** since our kebele is very wide and only one HEW is working. In addition, the supervision conducted by the health office also decreased which is making the service poor. The long-distance travel to the HP is also a barrier.

**D7-** the main obstacle for not starting the follow-up is the long distance of the HP, no other reasons.

**Reasons for discontinuation across the continuum**

**Moderator:** Why do women go to the facility for first ANC, but discontinue for subsequent ANC visits?

**D6-** they fear the surgery during the delivery this is also the reason.

**D8-** sometimes the service provided is also the reason for the discontinuation; they did not include my complaint anyway. It is not patient-centered.

**D5-** most of the mothers prefer going to Tula Hospital, the mother perceived the provider in the health center are not competent (inexperienced). The service provided in this health center is not satisfactory and the community is not using the service.

**Traditional practices during pregnancy**

**Moderator:** Can you tell us about the traditional practices and beliefs during pregnancy?

**D9-** since we all take health education harmful traditional practices stopped.

**D3-** the traditional birth attendants will massage the abdomen of the pregnant mother. This is harmful to the mother and the baby.

**D6-** delaying the mother for health service utilization during labor and delivery is one of the traditional practices.

**D8-** pregnant mother delivering by traditional birth attendants is a harmful traditional practice.

**D6-** previously the pregnant mothers were provided with "soicho" to drink for the belief of cleaning the body. No one is practicing it.

**Moderator:** How do you see community volunteers/TBAs and health professionals and maternal health services provided to the community?

**D9-** HDAs will follow the pregnant mother at home they will encourage her to start the follow-up early. They will follow the mother until the mother gives birth, however, the HDAs stopped their work and nobody is following the mothers in their village.

**D7-** the HDAs stopped their work currently,

**Moderator:** Explain to us your experiences relating to the utilization of ANC care provided by skilled birth attendants.

**D7-** here the health center is not clean, and the bed and the blanket are not clean. The cleaning workers are not working since they are not getting a salary.

**D6-** few healthcare providers at the health center are giving unnecessary drugs to pregnant mothers.

**Moderator:** In your opinion, what should be improved regarding ANC services?

**D3**- Since we have only one HEW in the HP. If two or three mother comes simultaneously she cannot give quality service. In addition, there is a shortage of health workers in the health center, pregnant mothers must not wait for service.

**D1-** when we take laboring mothers to the HC at night they will refuse to wake up from their sleep. The government has to hire highly skilled professionals the mother must get quality service, and the mothers must not suffer. Sometimes the service providers will be late even if they will be absent from their work which is affecting the service we are getting.

**The practice of facility delivery**

**Moderator:** Do women think skilled attendance during childbirth helps themselves and their babies?

**D2-** they will give her a waiting area, they will follow her, and they give medication for fasting labor. Sometimes they left the mother alone when an emergency case comes.

**D8-**they will give her glucose if she has anemia, they also give her beverages to drink. When she reaches the time they will assist her to give birth.

**Reasons for use of facility delivery**

**Moderator:** Explain factors that would motivate women to utilize delivery services during their pregnancy.

**D6-** since the ambulance is coming fast for the service this is making the mother use the service.

**D3-** they even help her when she has retained placenta and this is making our mothers use delivery services.

**Barriers to attending facility delivery**

**Moderator:** If women deliver at home, what are their reasons? Explain the constraints that influenced women to utilize facility delivery services.

**D**7-when the mother did not start the ANC follow up she will give birth at home.

**D**9-she has a lack of awareness she might give birth at home. If she gets good health education she will give birth at HC.

**Moderator:** Distance and access

**D7-** the long distance of the HF and lack of transportation are also making the mother give birth at home. Sometimes the poor provided and the lack of cleanness of the HF also makes the mother give birth at home.

**D6-** in addition to the distance lack of an ambulance driver's phone is also the reason. This makes the mother give birth at home with traditional birth attendants.

**D5-** since the HDAs stopped working the mother is giving birth at home.

**Traditional practices during the intrapartum period**

**Moderator:** Can you tell us about the traditional practices and beliefs during childbirth?

**D4-**when the mother delivers at home the hygiene is not good making the mother and the newborn at risk.

**D3-** they use an unsafe blade to cut the umbilical cord which is very harmful to the baby.

**D6-** to prevent the mother and the baby from getting "dilao" they will cover her with several blankets.

**Moderator:** Do you think these traditional beliefs, religious practices, and cultural norms affect mothers to use care during delivery? Explain how and why?

**D3-**delaying the mother from seeking health services by praying at home is also a traditional practice in our vicinity.

**D6-**the prophetic pray which tells the mother might die is causing anxiety to the family which has to be stopped.

**D1-** few religious individuals are telling the mothers not to go to the HF as prophetic service. The mothers are suffering due to this practice. Plus traditionally the elders will tell the mother that this day is a bad day for giving birth which makes the mother hide her labor and delay service utilization.

**Moderator:** How do you see community volunteers/TBAs and health professionals and maternal health services provided to the community?

**D3-** the HDAs knew the phone number of the ambulance driver so they call it when the mother have labor.

**D4-** they will give her health education, and they health workers also go with the laboring mother during the referral.

**D5-** they prevent excessive bleeding after delivery. The instrument they are using for umbilical cord cutting is also clean, they use a new one for each child.

**For recently delivered mothers only**

**Moderator:** How do you rate the quality of care you received from the facility during childbirth?

**D5-** they are giving medium service.

**D4-**They are helping us a lot. It is of moderate quality.

**D3-**the service is not satisfactory.

**D8-**there is a shortage of service providers in the health center.

**Moderator:** In your opinion, what should be improved regarding facility delivery services? Continuity of care?

**D6-** availability of electricity and the cleanness of the bed and the room have to be improved. Previously the waiting room is available but not now. There is also a shortage of service providers.

**D7-** the health center has no supervisor. The supervisor is not following the health workers; even he is not checking the cleanliness of the facility.

**D8-** the electricity problem has to improve immediately.

**D6-** the ambulance is not providing the return service for the delivered mothers which is creating a bad reputation for the service. In addition, the supervisor of the health center has to follow the health service providers. Additionally, the regional officers have to come and see the service.

**D9-** sometimes the ambulance driver will not pick up his phone when we call at night. So we have to take the mother using other transport. No mother is happy with the delivery service provided by the HC.

**Postpartum for all participants**

**The practice of PNC**

**Moderator:** How early do women go for PNC? Why do they go at that time? Why earlier or later?

**D9.** After the 45^th^ day, the mother and baby will take a vaccination.

**D3-** she will return on the 45th day and will return every month till the third and she will complete the immunization in the ninth month.

**D3-** she will have care on the 3^rd^, 4^th^, and 7^th^ day after delivery. They will check her health, and her uterus and they will provide health education child feeding, and personal hygiene. Previously traditionally the baby's umbilical cut will be covered by animal dung now the HWs will give her medication to apply to the wound. Currently, we apply the "yemisrach” drug which is given after delivery. She also gets advice and service in family planning.

**Barriers to attending PNC use**

**Moderator:** What are the barriers to accessing PNC?

**D9-** they usually did not open one vaccine till many babies gather which is a problem for us.

**D3-** the HP is located far from our area too.

**D6-**transportation problem is also making the mother stop.

**Moderator:** Socio-cultural

**D2-** the mother believes the baby will be eaten by "Buda” and also get “biche" and this makes the mother not return after delivery.

**Traditional practices during pregnancy**

**Moderator:** Can you tell us about the traditional practices and beliefs during the postpartum period?

**D9-** they will give “Amesa" to the baby which harmful practice.

**D6-**traditional healers will look for “fiancho" and they will make the baby drink herbal medication.

**D3-** sometimes when the baby’s tooth starts to erupt they will use unhygienic metal instruments to extract the tooth.

**D4-** cutting the uvula is also practiced previously.

**D9-** they will also discard the clostridium.

**Moderator:** How do you see community volunteers/TBAs and health professionals and maternal health services provided to the community?

**D7-** the HDAs will give education about child feeding, and personal hygiene and also follow the growth of the baby.

**D9-** the HEWs and other health service providers will teach the mothers on breastfeeding, and how to keep hygiene. They teach her to exclusively breastfeed and add complementary food after the sixth month.

**D6-**the health service providers will check the health, and provide vaccination and family planning service. They also give her health education.

**Moderator:** How do you rate the quality of care you received during PNC follow-up? What kinds of services do you receive at PNC? Are you satisfied?

**D5-** they educate us today. They are giving us good service.

**D2-**They are providing a very good service.

**Moderator:** In your opinion, what should be improved regarding PNC services? Continuum of care?

**D7-** the ambulance must return the delivered mother to her home. The availability of the drug also needs improvement.

**D9-** meeting with the health service providers is necessary. They have to provide us with continuous health education.

**D8-** the cleanliness of the health center needs improvement.

**D4-** the availability of water and electricity has to be improved.

**D3-**they are forcing us to use the fifth-year family planning drug; they have to provide the service we select.

**D6-** there is no drug in the health facility; all necessary drugs must be available. They are forcing us to buy from private pharmacies. Additionally, only one ambulance is available for 12 Kebeles, which is very difficult for laboring mothers.

**FGD: 08**

**Moderator**: When do pregnant women start ANC service in your area?

**D1**: at the third month.

**Moderator**: what type of service is provided to them at the health facility?

**D1**: they will provide the service and next appointment.

**Moderator**: When do pregnant women start ANC service in your area?

**D3**: first they go to a health facility and check their pregnancy status in the health center. After they confirmed their pregnancy the service will be provided for them. As per their appointment, they will obtain service monthly.

**Moderator**: what type of service is provided to them at a health facility?

**D3**: there are drugs they will obtain there.

**D4**: they first get a laboratory investigation for HIV. Then, they will start the vaccination.

**Moderator**: in how many months they will go to a health facility?

**D1**: since the third month of pregnancy.

**Moderator**: other?

**D5**: after they tested for HIV and syphilis at the health center. Then, they go to health posts and obtain iron to prevent anemia and TT vaccine monthly. After they give birth they will give vaccines for children up to six months.

**Moderator**: how many times do they go to a health facility for the ANC service?

**D5**: four times.

**Moderator**: other?

**D2**: eight times. They will attend pregnant women's forums and monthly health posts.

**Moderator**: Explain factors that would motivate women to utilize ANC services during their pregnancy.

**D4**: to check her health status.

**Moderator**: other?

**D6**: they will check their health status such as blood pressure and body weight. Another factor that motivates them is to obtain the counsel of HEWs.

**Moderator**: other?

**D9**: the mothers will go to a health facility if the fetus in the abdomen will not move properly.

**Moderator**: If women do not go for ANC, what are their reasons?

**D1**: some women will follow the traditional practice and don't go to health facilities unless faced health problems. Also, those young married women follow the principle of some traditional women's practices.

**Moderator**: other?

**D4**: previously women will give birth at home without encountering any health problems. However, currently, HEWs counsel them and through the HEWs ambulance will take them to health facilities. Now they use services at a health facility.

**Moderator**: is the a distance barrier not to go health facility?

**D6**: we have an ambulance driver's phone number so we will call and bring the women to a health facility.

**Moderator**: is the a distance barrier not to go health facility?

**D2**: yes, the distance is a barrier for pregnant women not using ANC and health facility delivery services.

**Moderator**: is the finance barrier not to go health facility?

**D1**: previously the lack of money is a barrier to the use of maternal health services. However, now the government has availed the ambulance service. Due to this service, the lack of money is not a big problem in our community.

**Moderator**: Why do women go to the facility for first ANC, but discontinue for subsequent ANC visits?

**D5**: after they use the complete dose of the TT vaccine will not go to a health facility unless faced dangerous signs of pregnancy.

**Moderator**: Can you tell us about the traditional practices and beliefs during pregnancy?

**D1**: nothing.

**D4**: The heavy workload is harmful to traditional practice for pregnant women.

**Moderator**: other?

**D9**: they carry heavy objects on their back. This is a harmful traditional practice that affects women's and child health.

**Moderator**: Can you tell us about the religious practices and beliefs during pregnancy?

**D1**: there are no religious practices that influence mothers not to use maternal health services. Some religious leaders will counsel the mothers to use maternal health services.

**Moderator**: is there any traditional medicine provision practice for mothers during pregnancy?

**D**: there is no such practice in our area.

**Moderator**: How do you see community volunteers regarding maternal health services provided to the community?

**D6**: they counsel mothers to use maternal health services from the health post and health center. They tell mothers not to give birth at home and teach them to receive counseling from health professionals.

**D4**: first they motivate pregnant women to use four TT vaccines. Second, they counsel mothers only to provide breast milk for the babies until six months and start complementary food immediately at six months.

**Moderator**: How do you see TBAs regarding maternal health services provided to the community?

**D1**: they counsel the women not to give birth at home.

**D4**: previously they attend delivery at home but now they will penalize on the legal ground if they will attend the delivery. We will take the laboring women to the health facility by ambulance and they never touch the laboring women at that time.

**Moderator**: other?

**D5**: previously they attend home delivery and women were seriously complicated. Currently, they are not visible in the area of laboring women due to legal punishment.

**Moderator**: other?

**D6**: currently the laboring women will not want the TBAs in their area. If the labor becomes very worsening the woman will tell to people to take her to a health facility.

**Moderator**: How do you see health professionals regarding maternal health services provided to the community?

**D6**: the community is satisfied by the service of health professionals in our area particularly at health posts.

**D2**: despite the far distance our community sees health professionals’ service as very good.

**D4**: the community is not satisfied due to the shortage of drugs in health posts.

**Moderator**: do the health professionals care for the women during the service provision?

**D6**: yes, they are women highly. However, there is a shortage of drugs since the start of 2015.

**Moderator**: do the health professionals protect the privacy of the women during the service provision?

**D2**: they very well protect their privacy of them. They investigate after they closed the door.

**D5**: the health professionals are very much satisfied to attend delivery as compared to providing the other services. They will give high protection for the laboring women and are highly satisfied to provide the service for women. The family will arrive after they have completed all procedures for the mothers otherwise no one can access them.

**Moderator**: Are there any health facility-related weak sides that need improvement during the maternal health service provision?

**D3**: there are shortages of drugs in the health center.

**Moderator**: other?

**D4**: the shortage of equipment and supplies.

**Moderator**: what services do women obtain during childbirth at a health facility?

**D8**: they will give injections and drugs after women give birth. Also, they provide injections and drugs for the neonates. The women and children become healthy if the women give birth at a health facility.

**Moderator**: other?

**D8**: they do the laboratory investigation for the laboring women. Also, they physically examine the mother to know the time the woman will give birth. They wait until the time of delivery and prepare women for childbirth. They will take laboring women to the delivery room and bed if the time of delivery is reached. Then, they will give the delivery of care or service to the mothers and neonates. Finally, they prepare the postpartum waiting area.

**Moderator**: other?

**D8**: they will test HIV and prevent not to transmission from mother to child.

**Moderator**: other?

**D5**: they will test HIV and check the time of delivery. If the time of delivery approaches they will attend delivery and remove the placenta. They will provide the needed things for neonates such as BCG and polio vaccine. They will keep in the postnatal ward for six hours and discharge accordingly. They will follow the danger signs of mothers and neonates.

**Moderator**: Explain factors that would motivate women to utilize delivery services during their pregnancy.

**D4**: the proper care for the mothers and neonates at health facilities motivates them to go to health facilities for the delivery service.

**Moderator**: other?

**D6**: the benefits they obtained from the health facility will motivate them.

**Moderator**: If women deliver at home, what are their reasons?

**D6**: they give birth at home due to traditional practice and are unwilling to receive the message of health professionals. They neglect the education provided by health professionals by saying previously our mothers gave birth at home without any problems. So, the women say why do we go to health facilities to give birth there without facing health problems? Sometimes women are facing serious health problems by giving birth at home due to their negligence.

**Moderator**: other?

**D6**: previously this is happing in our community but now there is some improvement. Most of them give birth at a health facility due to fear of some problems happening at home delivery such as morbidity and mortality of mothers and neonates. The attendant at home delivery will use the old blade to cut the umbilical cord which creates serious problems.

**Moderator**: Is distance a barrier to accessing health facility delivery?

**D1**: yes previously but not now.

**D2**: currently there is a network problem. The main problems are awareness and attitude. Some mothers give birth at home due to attitude problems.

**Moderator**: Is the quality of care a barrier to accessing health facility delivery?

**D5**: some delivery at home due to far distance and attitude problems. By chance, they will give birth at home during the delay of an ambulance. The quality of care isn't a problem that makes women give birth at home.

**Moderator**: Can you tell us about the traditional practices and beliefs during childbirth?

**D3**: home delivery by itself is a harmful practice that harms women.

**Moderator**: other?

**D4**: sometimes the abdominal massage and traditional way of balancing the fetal position.

**D5**: the traditional practice that harms women is an inappropriate way of attending home delivery. For example, women who are giving home birth will not open their legs adequately which may be suffocate the fetus. They will suffocate mothers with blankets and other things due to the misperception of this procedure will speed up labor. Other to deliver the retained placenta they will shake mothers which lead to serious bleeding and further complication of the retained placenta in the form of tearing. They also insert their unclean hand to deliver the retained placenta. These lead to serious maternal morbidity and mortality.

**Moderator**: How do you see community volunteers about health facility delivery services provision in your community?

**D6**: they provide counseling for the women to give birth at a health facility.

**Moderator**: other?

**D**: ……….

**Moderator**: How do you see TBAs about health facility delivery services provision in your community?

**D1**: they have harm rather than benefits.

**Moderator**: How do you see healthcare professionals about health facility delivery services provision in your community?

**D4**: they will provide quality service for all mothers without discrimination.

**Moderator**: other?

**D5**: they will provide education about the benefits of maternal health services. If the women become pregnant they will provide the ANC service. The mothers will be informed what to prepare and make ready during the pregnancy and prepare the transportation service after giving birth. Because the ambulance will not provide the service after women give birth.

**Moderator:** How do you rate the quality of care the mothers received from the facility during childbirth?

**D1**: it is quality service and women satisfied. The health facilities are providing the 24 hours service 7 days per week without interruption.

**Moderator:** Does the health professional provides quality care and non-dignified care for mothers in your area?

**D6**: yes, they provide non-dignified care for mothers.

**Moderator:** other?

**D3**: yes, they provide quality service.

**D6**: they provide a private room for the laboring mothers.

**D8**: most of the time older women in our area want to enter a delivery room and support healthcare providers but they do not allow them to enter the delivery room. They provide quality service for the mothers and maintain their privacy.

**Moderator:** is there any possibility that the health professional providers are unavailable in the health facility in your area?

**D3**: there is no such situation in our area.

**Moderator:** Does the health professional provides respect for the mothers in your area?

**D4**: sometimes there are health professionals who will not respect the mothers. The government seriously evaluated the professionals taking legal action for that matter.

**Moderator:** In your opinion, what should be improved regarding facility delivery services?

**D4**: the healthcare professionals should be available all the time in the delivery room.

**D2**: sometimes there is a shortage of materials like gauze and gloves in the delivery room. The mother was subjected to unnecessary referral due to a shortage of materials and equipment this should be corrected.

**Moderator:** other?

**D5**: For one woman the labor suddenly started last time and we called for the ambulance driver. He said it is not my round or duty to switch off his phone number and the second driver also switched off his phone number. The women faced serious problems at the time. Finally, we called for the administrator's car driver he took the mother to a health facility. Due to this delay, women developed serious complications. The ambulance service should need improvement.

**Moderator:** other?

**D4**: the shortage of fuel for the ambulance should be improved.

**Moderator:** other?

**D8**: the ambulance service should need improvement over the weekend.

**Moderator:** anything that requires improvement in the delivery room of the health facility?

**D**: there is no problem in a health facility. There is no sanitation problem in health facilities due to the adequate numbers of sanitary experts. They will immediately clean the room and bed after mothers give birth.

**Moderator:** anything that requires improvement on the side of a husband regarding health facility delivery use?

**D**: I think the husband is highly cooperative in our area to support women to attend health facility delivery. Nothing should be improved on the side of the husband.

**D4**: who cares if I do not support my wife at the time of childbirth?

**D1**: this issue doesn’t reflect our kebele and woreda in short.

**D8**: the ambulance should need to provide both rounds of service. Most of our people are poor and haven't financial access to pay for a car or motorcycle after their mothers give birth.

**Moderator:** How early do women go for PNC?

**D5**: they will go health facility on the 45^th^ day often with child vaccination service as per the national schedule until 1 year and 3 months.

**Moderator:** what types of service do women get after they give birth at a health facility?

**D4**: they will obtain family planning and counseling.

**Moderator:** what other service do women get after they give birth at a health facility?

**D3**: they will obtain child spacing service.

**Moderator:** If women don’t go for PNC, what are their reasons?

**D9**: the old age mothers and fathers will prevent mothers not to go outside the home with neonates due to traditional beliefs. However, now they are receiving education and there is improvement.

**Moderator:** Does the distance influence the mothers who don't go for PNC service?

**D3**: I think it may influence the service uptake. Distance is not a big problem in our kebele but the district administrator should need to identify the far kebles and search the means to avail the service for the mothers.

**D4**: I disagree that distance doesn't influence service use. I think it is all about neglecting the education provided.

**Moderator:** Can you tell us about the traditional practices and beliefs during the postpartum period?

**D5**: there is the fluid called ‘Amesa' that provides for the child.

**Moderator:** other?

**D6**: she said ‘Amesa' earlier. Some mothers will provide milk and boiled water for the child by saying my child's abdomen is empty before six months.

**D1**: the way they will handle the retained placenta is harmful traditional practice for mothers and neonates.

**Moderator:** other?

**D5**: they will milk through the first milk of mothers (colostrum). This is harmful to traditional practice during the postpartum period in our area. However, the science says this is a vaccine for the neonate. They also start complementary meals for six months.

**Moderator**: How do you see community volunteers with regards to postpartum services provision in your community?

**D**: they counsel the postpartum women about breastfeeding, child care, and personal hygiene.

**Moderator**: How do you see health professionals with regards to postpartum services provision in your community?

**D2**: they will provide the postpartum service as per the national schedule for example in the first 24 hours, 3^rd^ day, 7^th^ day, and 54^th^ day. They also counsel the postpartum danger signs and to come health facility if they experienced it. Further, they will inform women to use child vaccination and family planning service on the 45^th^ day.

**Moderator**: In your opinion, what should be improved regarding PNC services?

**D4**: the community-level performance review meeting should be strengthened to improve the maternal health service.

**Moderator**: other?

**D9**: the training should be provided for the community volunteers. Previously there was much training for the community volunteers but now the Woreda government is not giving for them.

**Moderator:** anything that requires improvement on the sides of the health facility?

**D2**: there is a lack of short-duration contraceptive drugs. The women in our area prefer a 3-month duration of family planning as compared to 3, 5, and 10 years. The health professionals counsel them but the women refuse to use long-duration contraceptive drugs. Due to this, there are a lot of unwanted pregnancies occurring in our locality. The government should avail the short-duration contraceptive drugs, particularly the three months.

**Moderator**: other?

**D9**: the electricity should be accessible in the health post.

**Moderator**: other?

**D1**: the shortage of HEWs should be addressed. They are two in number so they cannot able to provide health services at the health post and community level. The health post is closed if both of them go to the training or meeting in another place.

**Moderator**: Lastly if you want to add any things?

**D8**: the distance is a big problem in some areas of this woreda and even the health post is far to access. This thing should be corrected for the mothers to obtain proper service.

**FGD: 09**

**Moderator:** How early do women go for ANC service in your area?

**D3:** first, in our area, women go to the health post for ANC follow-up. When the date for delivery is approached and labor starts we call an ambulance to receive the woman from the health post to a health center. Women gave birth at health facilities by skilled healthcare providers and obtain the needed care. However, after a few time stay in a health facility the delivered women will come back to their home. Despite these services, there are women from poor families that lack food after they give birth. Our community hasn't any favorable conditions around 10 or 12 years back due to a shortage of food. There is a shortage in this regard and we are asking the aid from the government. Sometimes we obtain aid but most of the time we haven't from the government. There is service in health facilities but poor family women haven't access to the food and towel that covers the baby after childbirth. Thus, their life is miserable.

**Moderator:** others?

**D1:** there is a service in the health post and center in our area. However, there are great problems in the provision of maternal health services in our health center due to the lack of electricity. They have a generator but not work due to a shortage of petroleum. At night time healthcare providers attend delivery by a battery that hinders them not to provide quality service. Another thing is the lack of water supply in the health center which influences the cleanliness of the activities. Women obtain maternal health services from a health facility. However, the service provided doesn't benefit them because they are already under-nutrition as a result of the food shortage in our area. In this locality, due to poverty, the women are unable to care for and manage their children. They give their children to the government and other individuals who have better status. The food shortage is a serious problem in our area.

**Moderator:** others?

**D4:** I want to talk about family planning. Previously there is a short period of family planning drugs in our health center. Currently, the healthcare providers informed us this drug is disappeared from the health center. They force mothers to use long-period family planning drugs like five years. They insert the drug into the arm and counsel mothers not to do heavy work. During this time who is a responsible person to substitute her work? As you know our mother is under-nutrition and the complication of drugs add further pressure on their health why is this thing happing in this area? Regarding the ambulance service they only receive the laboring women from the health post to the health center but do not provide the service from the health center to home. During this neglected period, serious problems are happing in our mothers such as bleeding, infection, exposing the mothers and babies to pneumonia, and big confusion for the family. The referral system is also not well organized between the health center and the hospital. The healthcare providers from the health center give the referral paper to the mother but the hospital will not accept it.

**Moderator:** others?

**D2**: first, they use TT injection service as per what they learned during the pregnant women forum. After they have completed their injection during the pregnancy period and go to a health center for delivery service. However, if labor suddenly started and is very fast calling for an ambulance is mandatory. The problems in the health center are lack of electricity and clean water. After they gave birth in a health facility there is no ambulance service which is a serious problem. The other thing is a shortage of food in our Kebeles due to drought. Previously our mothers will prepare the 'kocho', butter, flour for porridge, and money for transportation before the date of delivery. Now a day due to poverty and economic crisis they will prepare nothing. They haven't anything that they eat after returning from health facilities to the home. Due to this reason, the mother and child become under-nutrition. They also haven't materials like blankets and towels to wear after delivery. Breast milk is deficient for the baby due to the mothers haven't adequate food intake.

**Moderator:** others?

**D3**: I want to add one thing. Due to the lack of a 3-month family planning injection mothers who have financial access are using this short-duration injection from a private clinic. However, poor mothers are forced to use 5 years of drugs which is creating a serious problem for mothers. This is a big concern and should need correction as soon as possible.

**Moderator:** in your locality how many times do women go to health facilities for ANC service?

**D6**: they go frequently for injection till the date of delivery. They will give injections once and pills. We will use this pill till the date of delivery. Previously I used 3 month contraceptive injection but now they provided me the 5 years. This five years drug is creating serious problems in our health. I have removed it because of this problem. I developed the wound during the removal procedure by 3 healthcare professionals. At the time I haven't used any contraceptives and faced unwanted pregnancy. For this pregnancy, I have used ANC service frequently both in health posts and health center. The labor suddenly started they called an ambulance and we arrived at the health facility. The health center referred me to Leku Hospital and further Leku Hospital referred me to Adare Hospital. I gave twin birth in Adare Hospital. The healthcare providers haven't provided me with information and much care but much care is needed for women who gave twin birth. Even they didn't demonstrate to me how to feed breasts. They also not provided me with ambulance service from the hospital to my home. I used bajaj to go back to my home. The kebele provided me aid but now stopped. We have a shortage of food due to this problem we are not properly caring for our children.

**Moderator:** in how many days you have been discharged from Adare Hospital after giving birth?

**D6**: in the same day I gave birth. I gave birth at night around 4:00 but they discharged me early morning at 2:00 local time. However, they haven't provided me with any support. One person gives me support such as a towel and other materials.

**Moderator:** How early do women go for ANC service in your area?

**D6**: they frequently go to health facilities till the 9^th^ month but stopped after the 10^th^ month. **Moderator:** How early do pregnant women go for ANC service for the first time?

**D1**: they will start ANC service in the fourth month and continue till 9^th^ month. However, in recent days healthcare providers gives pills to pregnant women. We don't know the benefit of that pill. Currently, women are using the five years contraceptive drug. The majority of mothers that used this drug will give twin birth but the government is not providing support for those mothers.

**Moderator:** do you mean women give twin birth after the removal of five years of contraceptives?

**D1**: yes, they give twin birth. In our area, this is a common phenomenon.

**Moderator:** do you know the color of the pill that was provided during the pregnancy?

**D1**: yes, it is red. They provide around 60 pills.

**Moderator:** Do women think skilled attendance during pregnancy helps their pregnancy?

**D6**: no one supports us rather than health posts.

**Moderator:** Do you think the service is helpful for mothers?

**D6**: I think it is important. However, no one supports us rather than health posts.

**Moderator:** Why do women go to the facility for first ANC, but discontinue for 2^nd^, 3^rd^, and 4^th^ ANC visits?

**D1**: The health center has scheduled the ANC service on Monday and Thursday. However, the health care providers will not be available at that time in the health facility. Besides, there is a shortage of drugs. These conditions will create a double burden for pregnant women. Due to these reasons, women will discontinue the subsequent visit.

**Moderator:** other?

**D4**: we reported the problems of our health center repeatedly to higher officials but till now the problems have not been solved. The lack of electricity and clean water supply in health centers seriously affects the quality of service provided. Also, the lack of adequate food for the delivering mothers.

**Moderator:** other?

**D3**: I want to add one thing; there is a serious shortage of drugs in health centers. We go to health facilities but they prescribe drugs to private health facilities. We have a limited amount of money in our pocket but they ask too cost amount that is the reason hindering the service use in health facilities.

**Moderator:** other?

**D9**: previously in our health centers there are qualified health professionals. Now the government transferred them to another place. Most of the time health facility has empty (without any health professionals) when we go to the health facility for the service. The security person will call them by phone but they come later. Besides, they refer all cases to Leku Hospital and prescribe to private health facilities. There is no water in our Kebeles and mothers are in serious problems. Our health center is built for the name and empty.

**Moderator:** water supply isn't available in the delivery room of the health center?

**D9**: nothing at all.

**Moderator**: do you think distance is a barrier to accessing the ANC service for mothers?

**D9**: yes. Far distance is a barrier for mothers to influence the utilization of service. Previously healthcare providers come up to our home to provide the health service but this trend isn't seeing in our area recently. We have 'Honso got’ or ‘Botano' village which is found beyond the river and hard to reach area. The pregnant woman who carries a fetus in her abdomen from that place cannot able to access ANC service due to it requires energy to cross the river and walk a long distance. However, pregnant women haven't much energy and it is big programs. It is also challenging for the women to bring the child to a health facility even after the 40^th^ day for child vaccination and family planning.

**Moderator**: other?

**D1**: in our village (Hireye) more than 30 to 40 mothers gave unwanted births due to a lack of the short-acting contraceptive drug. This drug is available in private pharmacies but not in our health center pharmacy. Its price is 100 birr in the private pharmacy which is too cost for most of our mothers. Why this drug is available there but not here? The electricity and clean water availability is a serious problem in our area. Due to the electricity problem, the health professionals use a battery at night which leads to improper suturing of the mothers and re-performing the procedure. This increases the rate of infection and doubles the burden for the mothers.

**Moderator**: the problem of light is at night only or at all times?

**D1**: at all times. They suture the mothers two times due to procedural errors. The health professionals correct each other and perform the same procedure many times. In our health center, there is a big program regarding the quality of service.

**D2**: in our health center absence of an adequate number of health care professionals. They transferred those qualified and experienced health professionals to other places. The currently available health professionals cannot able to diagnose the disease properly. They can't able to identify even malaria and typhoid fever infections. They have only drugs for the head but lack even for malaria and will not give us the malaria drugs. Last year we were ranked at the third level regards community-based health insurance but this year the number of members is significantly declining. This is due to the poor quality service they provided. There are problems in both health posts and health centers. We are not obtaining adequate service.

**D7**: we confirm our pregnancy status in the fourth month at the health facility but we will start ANC visit at 6 months as per their recommendation. We will start the ANC visit as per their suggestion and they provide us with the pill and we will use the pill till the date of childbirth. We will visit the health facility on the 45^th^ days after childbirth. During this time they will provide the vaccination service for the child. However, unfortunately, this tells us about the absence of family-planning drugs. The vaccine should be provided for children up to nine months. We appropriately obtain the vaccination service on the first visit (45^th^ day) but they tell us the absence of vaccine or drugs was not open for the rest of the appointment period. We will visit health facilities several times to obtain a single service. This is a big problem we encountered in our area.

**Moderator**: could they say we haven’t drugs for mothers in first contact at 6^th^ month of pregnancy?

**D7**: we obtain the vaccine for the first time and pill but they tell us the absence of the vaccine in the next appointment. Previously mothers will receive the vaccine for up to nine months but now they provide us only a pill. We will give birth by using this pill. If we encounter health problem the health professional examine our pregnancy status before prescribing the drugs. However, currently, they will provide ampicillin 500 mg without checking the pregnancy status. We are facing serious health problems or harm due to these reasons.

**Moderator**: Are there any socio-cultural barriers to accessing ANC service in this area?

**D2**: no. there are no sociocultural practices that prevent women not to using ANC services. Nothing will prevent women not to go health posts or health centers. Previously the health care providers provided education for the women to go to health facilities and use the services. As per their education, women are going to health facilities and using the service and thus nothing will prevent them from using the service.

**D1**: no socio-cultural barriers. They are using the service. However, as earlier mentioned by our brother previously the health care providers will visit home to home. However, currently, they provide the service at the health post and health center levels. They don't care what is happing at the community level. There is a big problem in health centers and the community is seriously disadvantaged currently. In one village 45 children delivered in one year means a big problem. We are under serious poverty and stress due to the lack of awareness.

**Moderator**: do you say home-to-home visits stopped recently in your area?

**D2**: yes, they stopped home for a home visit. Who cares if it is the choice of pregnant women to deliver in a home or health facility? They neglected the care provided to mothers and many things are expected and are not given to mothers.

**Moderator**: are there any barriers that influence women to deliver at home in this locality?

**D2**: they will come and receive the pregnant women by ambulance after several calls but most time our community uses a motorcycle and gave birth in a health facility. No woman gave birth at home. However, previously there is a promotion campaign from the health facility that motivates women to give birth in a health facility. Now the movement is not visible in our locality and the health center has become autonomous which means beyond our capacity to utilize it.

**Moderator**: other?

**D4**: previously there is a socio-cultural barrier the laboring women will delay at home for a long time without any care. Due to this women will develop serious problems. However, currently, in our area, there is no sociocultural barrier that influences women from the use of health facility delivery. Our community obtained education from health professionals about the benefits of health facility delivery. The women are giving birth at health facilities as per their education message. We will receive the laboring women at the health facility immediately after the start of labor by motorcycle or ambulance. Our catchment consists of four Kebeles, four health posts, and one health center. Sometimes women give birth at home because of a lack of strict follow-up from health centers and health posts. However, there is no sociocultural barrier that influences women from utilizing the service.

**Moderator**: other?

**D6**: the one reason that facilitates the women to give birth at home is the unknown expected delivery date. Previously health professionals will inform the mothers about the expected date of delivery during the ANC service. However, currently, they haven't informed them of the expected date of delivery. The labor will start for the mothers suddenly while they are performing their daily activities and giving home birth.

**D9**: there are many problems in our health center. First, the health professionals are not skilled to provide quality service. They cannot able to properly diagnoses the diseases and manage the laboring women. They cannot properly identify malaria and typhoid fever infections. Even not properly investigate the pregnant women and estimate the delivery date. They provide the injection in the injection room without the laboratory investigation. Also, they provide the injection for the person that requires the pill and the reverse is true. Further, they provide drugs for headaches for abdominal pain, and vice versa. I think the security person is better than them. The solar in our health center was damaged and they complain about the fuel shortage for the generator. Pregnant women will not obtain laboratory investigation services due to these problems. They also do not properly inform the pregnant women of the expected date of delivery. There are so many mothers has faced stillbirths in this health center. The health professional will go to the lounge while the laboring woman is at the delivery bed. They prescribe drugs to mothers without checking the pregnancy status. They refer us to Leku Hospital but we haven't money to go there. They discharge the delivered mothers after the second or third days. Sometimes even they discharge afternoon if women give birth in the morning. Health problems are so serious during this critical period for mothers and babies. Moreover, the problems are worsening in the case of twin pregnancy. There is no proper care for the mother after giving birth in a health center such as soap to wash her body and cloth to cover the baby. Maternal death is much higher in the health facility. Home death is safer. We haven't a health center or water supply in our locality. The pregnant mothers are drinking water that contains small parasites. The kebele leader cannot do any things without the support of the health center and woreda. Many laboring mothers are dying at home. The health professionals are too many in numbers but do not properly work the government working hours. They will receive a salary monthly from the government. Previously there is qualified and experienced health professionals who now transferred to other workplaces. There is a serious shortage of drugs in health centers and you can say the health center is empty. The laboring women are facing many problems due to the delay of ambulance service during the emergency.

**Moderator**: why the ambulance driver delays after your phone call?

**D9**: due to lack of health professional attention. There is no proper health care professional that calls ambulance drivers by emphasizing women's problems. However, they emphasize their problems. They say we haven't drugs and write the prescription for us to buy them from the Leku hospital. We can't able to buy drugs from the Tula Hospital with our community-based health insurance cards. However, this is not fair for the poor family or widowed women.

**Moderator**: do you think that all the important materials provide for mothers after giving birth such as cloth to cover the child and child caps not provided for mothers?

**D9**: yes, there is nothing provided currently. Even there is no soap for mothers to wash their bodies after they give birth. They will come home by their blood after giving birth at a health facility. Previously mothers will obtain many materials like towels and child caps after giving birth but now they stopped to give. They discharge mothers immediately after they give birth. They haven't cared for mothers in this critical period. The security person Mr. Tadele is better than them to treat mothers. They haven't understood even personal hygiene and treat the patient without washing their hands. The experienced and qualified health professionals were transferred to Hawassa City. Currently, we are not obtaining any services from the health center.

**Moderator**: other?

**D5**: I want to add something. Currently, there is no difference between giving birth at home and health facilities because of the lack of proper care there and this is one reason that makes women prefer to give birth at home. For example, there is no fluid to provide for the mothers during labor in a health facility. We go to the market to buy soft drinks to provide after they give birth. The health professionals even can't provide them with proper soft drinks for them. There is no soap to wash her body. Due to these reasons, women in our area prefer to give birth at home.

**Moderator**: do you mean they prefer the home birth?

**D5**: they prefer to give birth at home. They concluded that what makes the health facility differ from our home. They assume that even home delivery is safer than health facility deliveries due to the lack of care in health facilities.

**Moderator**: other?

**D5**: we buy the IV fluid after mothers give birth. Sometimes there is a time that IV fluid disappears from this area and we will go to Hawela town to buy it. At that time the mother will become unconscious the neighbor's people bring water and apply it to her head. We haven't obtained the service from the health center.

**Moderator**: other?

**D**…

**Moderator**: Are there TBAs in your locality?

**D1**: there are no TBAs in our locality. Most of the time the women are giving birth at health facilities and some give birth at home by themselves. But there are no TBAs in our area.

**Moderator**: other?

**D6**: they were around 2005/6 E.C. However, they do not exist now in our community due to the government taking serious legal action against them at that time.

**Moderator**: Are there community volunteers in your locality?

**D1**: yes, they are working together with health extension workers (HEWs).

**Moderator**: other?

**D8**: yes, they are working with HEWs.

**Moderator**: How do you see community volunteers about maternal health services provision in your community?

**D6**: they exist in our area at the name level. However, they are not providing the maternal health service properly. They are not counseling and teaching pregnant women.

**Moderator**: other?

**D5**: they are only visible at the time of the campaign. They provide injections and pills for vaccination and another campaign as per the schedule of the regional government. The HEWs will call them at the time of the campaign and together with the HEWs will work only at that time. However, they will not counsel, teach and provide maternal health services at health centers, health posts, and community levels.

**Moderator**: are you saying they only work during the campaign time?

**D5**: they are even visible at the campaign time to collect the perdiam. They are not providing any maternal health service at the community level, health post, and health center.

**Moderator**: How do you see HEWs about maternal health services provision in your community?

**D1**: previously after two years they properly provided the maternal health service at home-to-home visits. However, now they provide the service at the health post level only. The health center head doesn't supervise them and the women provide the service for those women visiting the health post.

**Moderator**: other?

**D6**: they are not properly providing the maternal health service. They only provide the service for mothers after women complain at health posts but they are not counsel and teach them.

**Moderator**: other?

**D2**: the health post is closed most of the time. They will not notify their program and the health post is closed most of the time. Many pregnant women and women who have little children for the ANC follow-up and vaccination service will wait for them for a long time. As you know those mothers will come by crossing the river and travel the long distance. They finally will return to their home without obtaining the service. There are many problems in HEWs and not providing maternal service properly.

**Moderator**: do you mean that they close health posts most of the time?

**D2**: yes, most of the time the women will not access them here (health post). They are not properly providing the service for mothers as per their monthly appointment even for the vaccine service. However, they completely stopped home-to-home visits despite the campaign days. I think the service is inadequate.

**Moderator**: If women deliver at home, what are their reasons?

**D2**: they prefer delivery at home because of the lack of proper care at the health facilities. Previously there are many proper cares for mothers after they give birth at a health facility such as soap to wash their body, towel, and cup to cover their child, and IV fluid if they bleed. Also, previously discharge mothers from health facilities after a reasonable time of stay. Now they are discharging the bleeding women. These reasons make women prefer home. The mothers also prefer to give birth at home because they want to maintain their privacy.

**D6**: in our area the wives’ of Ebiru Kasim gave birth at Leku Hospital after several processes. He has poor and we received her by motorcycle. They discharged her immediately after giving birth. They haven't obtained any transportation even a donkey-drawn cart. She returned to her house by walking foot slowly. Mothers state that they will face a similar situation with this woman if they go to a health facility to give birth. They say that an ambulance will take us to the hospital but no transportation service to return our home. They fear this kind of unnecessary exposure to transportation problems. They state that even if we die our home is safe as compared to the unnecessary problems we face during the referral system.

**D7**: I gave birth at the health center for my first child. During that time I faced several problems. First, during labor time, I thirsted for water but there is no available water source. My family provides me the soft drinks that highly disturbed my stomach. Second, due to a lack of water, I was not washed my body till the second day. I asked one health professional to give me water to wash my body and he searched for water from the water tank but lost water from that source. He automatically told me to call the people that receive me at my home. The day was Sunday and the midwife who attended my delivery went to church and no one care for me during that time. I asked him what would be better for me at that time. He said by whatever means you go to your home and wash your body at home. Then, I asked him to discharge me when I experienced pain but he discharged me using the informal way. At the time of returning to my home, I was ashamed of my body because it contain blood. Due to this I not followed the main road to my home and crossed through the farm plots. I washed my body after I had returned to my home. Due to all the improper care, I seriously refused my family not to take me there. I gave birth to my second child at home. I preferred to give birth at home not due to traditional practice but due to a lack of proper care at a health facility. In our home, we can drink water and wash our bodies as we like. We prefer to give birth at home because the health problems were increased in the health facility. However, there are no socio-cultural barriers that hinder us not to attend health facilities.

**Moderator**: are you saying they discharge the women using the informal procedure?

**D7**: yes, he told me he has planned to go to church. He has also told the security person that he has planned to discharge me due to the church program. Most times we gain many benefits if we give birth at home such as water to drink and wash our body. We will not obtain water access there.

**Moderator**: Are there other barriers that make women give birth at home?

**D8**: due to lack of proper care at the health facility. They order us to feed our breast to the neonate immediately after birth but our breast is contaminated with blood. They also forced us to wear blankets contaminated by blood and to take home our towels contaminated by blood. Previously they provide drugs and injections via the mouth and lower arm to neonates. Moreover, they provide injections for women that strengthen women after childbirth. Now these services are stopped and forced to use five years of contraceptive drugs immediately after we give birth. They will not give proper postpartum care to the mothers and neonates.

**Moderator**: Can you tell us about the traditional practices and beliefs during childbirth?

**D1**: nothing. Due to nutrition shortage lactating mothers and neonates may be harmed but there are no traditional practices that harm mothers and neonates.

**D2**: there are no traditional practices that harm mothers and neonates. The government and health care professional recommends exclusive breastfeeding for up to six months and nothing will provide to babies other than breast milk. However, the mothers haven't adequate diet due to this the breast milk will not satisfy the demand of babies. The mothers and neonates will be undernutrition.

**D4**: the main traditional practices in our area are those pregnant and lactating women who will do heavy manual work due to poverty. Due to this reason maternal and neonatal morbidity and mortality are high in our locality.

**D6**: most of the time women develop the infection during pregnancy time in our locality. Syphilis and urinary tract infections are common. These infections will lead to back pain and loss of appetite. However, there are no adequate medicines and vaccines in our health facility to prevent it immediate as infection happens.

**Moderator**: How early do women go for PNC service in your area?

**D8**: after one month and fifteen days will go to a health facility. We will go whether the health center or health post. They will provide the vaccination service to our children but not give us the family planning drugs. Besides, they will not counsel us about child and maternal health care during the postpartum period. They will inform the appointment dates of the child's vaccination.

**Moderator**: other?

**D7**: we go to the health facility on the 45^th^ day. They will give the vaccine service to children but we do not properly obtain contraceptive drugs.

**Moderator**: are there any other PNC services other than child vaccination and family planning in your area?

**D7**: nothing other than these services.

**D2**: the 5 years contraceptive drugs have serious consequences for rural women due to the workloads they have.

**Moderator**: other?

**D1**: we have finished and it is better if we stop here.

**Moderator**: thank you a lot for your time and information.

**IDI-01**

**Interviewer:** How early do women go for ANC?

**Interviewee:** we will go after 4 or 5^th^ of pregnancy to obtain laboratory investigation service. Also, the healthcare providers will give us the TT vaccine. Then, will visit the health facility till the date of delivery. We are giving birth at a health facility.

**Interviewer:** why do they go at that time (4 or 5^th^ months of pregnancy)?

**Interviewee:** we will go at that time to know the health status of the mothers and fetus. The TT vaccine is also mandatory to receive at that time.

**Interviewer:** other?

**Interviewee:** another reason is those mothers who have problems with the uterus carrying the fetus need to investigate at that time. If she has a serious problem they will send her to hospital. They also refer to those mothers who have fetal malposition and mal-presentation.

**Interviewer:** other?

**Interviewee:** because it is important for the mothers

**Interviewer:** Why do women go earlier or later than above stated time?

**Interviewee:** Mothers will not go there (to health facilities) before 4 months because their pregnancy isn’t visible before that time. They will come if they want to check their pregnancy status.

**Interviewer:** How often do they go to ANC?

**Interviewee:** they may go to a health facility based on their appointment provided by health professionals. They may probably go there 7 times till the date of delivery to check their health status.

**Interviewer:** Do women think skilled attendance during pregnancy helps their pregnancy?

**Interviewee:** all of them think it is important for mothers.

**Interviewer:** Explain factors that would motivate women to utilize ANC services during their pregnancy.

**Interviewee:** health professionals are motivating the mothers to use the subsequent ANC visit after their first contact.

**Interviewer:** other?

**Interviewee:** the WDT will motivate those mothers who are at home without ANC follow-up. They will tell the ANC visit is very important for the mothers and their fetuses. They also provide counseling for them.

**Interviewer:** other?

**Interviewee:** I don't know any more things that motivate the mothers to follow up on their ANC. The health problems during their pregnancy will motivate them to go to the health facility.

**Interviewer:** If women do not go for ANC, what are their reasons?

**Interviewee:** I think they are not wife and ashamed to go to the health facility. The old mothers will also be ashamed to show their bodies to the young health professionals.

**Interviewer:** other?

**Interviewee:** nothing

**Interviewer:** What are the barriers to accessing ANC?

**Interviewee:** the shortage of drugs is a barrier to accessing quality ANC from the health center.

**Interviewer:** are finance and opportunity costs related to barriers in your locality?

**Interviewee:** yes, for the laboratory service and some drugs the payment is mandatory. So if I haven't money how could I access the service? The lack of money is a barrier to accessing the ANC service.

**Interviewer:** Are distance-related barriers in your locality?

**Interviewee:** no, they will go to the health facility by using their umbrella even during the sun time but the serious problem is financial barriers.

**Interviewer:** are socio-cultural related barriers to your locality?

**Interviewee:** no, nothing socio-cultural related barriers.

**Interviewer:** are mothers and fathers-in-law related barriers in your locality?

**Interviewee:** no, nothing. The husband will take the pregnant woman to use the health service at a health facility.

**Interviewer:** are qualities of care related to barriers in your locality?

**Interviewee:** no. They will counsel the mothers and provide the appropriate care.

**Interviewer:** Why do women go to the facility for the first ANC, but discontinue for subsequent ANC visits?

**Interviewee:** aren't any problems. The service is free of charge. The health professionals will check the fetal movement in the abdomen. They will provide the appointment and mothers are using the ANC as per their appointment. We haven't problems they are providing the appropriate care for the mothers. Some mothers discontinue due to their attitudinal problems.

**Interviewer:** are finance and opportunity costs related to barriers in your locality?

**Interviewee:** yes,

**Interviewer:** Are distance-related barriers in your locality?

**Interviewee:** yes, some mothers will come from the far place like ‘Shamana Godo’ and ‘Gonowo Bulano’ Kebeles will pay 30 birr for the transport to go the health center. Due to the transportation fee and some drug costs, they will not go to the subsequent visit.

**Interviewer:** Are any religious practice-related barriers in your locality?

**Interviewee:** no

**Interviewer:** Are any traditional practice and belief-related barriers in your locality?

**Interviewee:** no

**Interviewer:** How do you see community volunteers regarding ANC services provided to the community?

**Interviewee:** They are motivating the mothers to use the ANC service.

**Interviewer:** How do you see ANC regarding ANC services provided to the community?

**Interviewee:** Since the establishment of this health center I can't see their role. Previously they provide a home delivery service. The problem the mothers faced during their traditional way of attending the delivery is they will tighten the placenta on the lower arm of mothers. Thanks to God since the establishment of this health center the health care providers are providing the proper service for us. The healthcare providers will follow us till the date of delivery.

**Interviewer:** In your opinion, what should be improved regarding ANC services?

**Interviewee:** we will receive the service based on government direction and improvement strategies.

**Interviewer:** what should be improved in the sides of your community?

**Interviewee:** the community should encourage the women to use the service as per government direction.

**Interviewer:** what should be improved on the side of mothers?

**Interviewee:** the women in the WDT and 1 to 5 network should encourage themselves to use the free ANC service in health facilities.

**Interviewer:** Do women think skilled attendance during childbirth helps them and their babies?

**Interviewee:** they think highly as it is important. The health care providers there will provide eye care and cord care to prevent bleeding. They also provide the proper care for placenta delivery. We think it is very important for the mothers and their neonates. However, the old mothers don't think it is important. They will ashamed to show their body to the young health professionals.

**Interviewer:** Explain factors that would motivate women to utilize delivery services in their childbirth.

**Interviewee:** the health professionals are motivating the women. They record the name of the mother during the first ANC contact and follow her till the date of delivery. They are giving us serious warming to prevent home delivery.

**Interviewer:** If women deliver at home, what are their reasons?

**Interviewee:** nothing. As you know the service is free of charge except for the shame of old mothers.

**Interviewer:** other?

**Interviewee:** currently there is no home delivery except for a few mothers.

**Interviewer:** are finance and opportunity costs related to barriers in your locality?

**Interviewee:** yes, most mothers will give birth at home due to financial problems. They haven't money to buy food, drinks, towel, and cup during childbirth. They are ashamed of this and give birth at home. Another reason is unwanted pregnancy.

**Interviewer:** do you say that unwanted pregnancy is a barrier to home delivery in your area?

**Interviewee:** yes, they are ashamed to use health facility delivery due to unwanted pregnancy. Unwanted pregnancies happen most of the time due to a shortage of short-duration contraceptives and methods failure. Due to financial problems, most mothers give birth at home.

**Interviewer:** Are distance-related barriers in your community?

**Interviewee:** no, the far distance is not a big problem alone but will have affected it combine with the financial problem. If the mothers have money they will take them by motorcycle to the health facility even if the distance is far. There are a lot of mothers who give birth at home due to financial problems. Even after they will give birth at a health facility due to financial problems they are walking by foot to their home. Their lip is very dry after they give birth due to a lack of fluid intake like soft drinks. They also are walking on foot to their home while bleeding after they give birth. These problems are due to the financial constraint. The majority of mothers are under serious problems in Sidama region.

**Interviewer:** are socio-cultural related barriers in your community?

**Interviewee:** no, nothing related to socio-cultural barriers in Sidama land. They all are giving birth at a health facility except for the challenges I mentioned to you earlier.

**Interviewer:** Why do women go to the facility for ANC, yet mostly deliver at home?

**Interviewee:** that is the problem of mothers. I don't the reasons why they give birth at home.

**Interviewer:** are qualities of care related to barriers in your community?

**Interviewee:** most of the time the health care providers give the available drugs. However, sometimes due to the drug shortage, they appoint the mothers for the next time. Mothers will not go back to the health center as per their appointment due to negligence.

**Interviewer:** Can you tell us about the traditional practices and beliefs during childbirth?

**Interviewee:** no, nothing in our community. They will go to a health center during their childbirth, deliver at a health facility without any problems, and provide proper care for the placenta delivery.

**Interviewer:** Can you tell us about the traditional practices and beliefs during childbirth at home?

**Interviewee:** I don’t have any information about this issue.

**Interviewer:** How do you see community volunteers regarding the health facility delivery services provided to the community?

**Interviewee:** they are encouraging the mothers to give birth at a health facility.

**Interviewer:** How do you see TBAs regarding the health facility delivery services provided to the community?

**Interviewee:** they haven't any contribution to the laboring mothers rather than harming. Previously they insert their unhygienic hands into mothers' bodies to deliver the retained placenta. Those mothers will develop serious complications and this is harmful to traditional practice. But now thanks to God they disappeared from our community since the establishment of this health center.

**Interviewer:** In your opinion, what should be improved regarding facility delivery services?

**Interviewee:** the poor women in our community will hungry in the delivery room after they give birth until the family brings food from home due to far distance. Previously some organizations provide the FAFA and oil for poor delivering mothers at health facilities. The family will prepare and give this food to delivered mothers at the health facility kitchen. During my second child's delivery in 2004, E.C. the health professional called Tarikuwa send me these materials after even I was discharged to my home. They prepare food in the kitchen of the health center but now stopped. The government should improve this and the drug shortage at health centers. I think the financial problem will influence the women to prefer to deliver at home and this should be improved.

**Interviewer:** what should be improved on the sides of health care providers?

**Interviewee:** nothing on the side of health professionals. They are providing the proper care for the delivering mothers like keeping the cleanliness of the delivery room and hygiene of the blanket that women wear after delivery.

**Interviewer:** what should be improved in the sides of your community?

**Interviewee:** I think the community should act in line with government policy to encourage women through their idea, finance, and labor force.

**Interviewer:** How early do women go for PNC?

**Interviewee:** between 2 and 3 months. They will provide child vaccine and family planning services on the 45^th^ day of delivery. They also provide the FAFA for some poor mothers.

**Interviewer:** why do they go on the 45^th^ day?

**Interviewee:** this is due to agreement and counseling with them. If we go to a health facility after the 45^th^ day the unwanted pregnancy will happen. We will go at that time to prevent unwanted pregnancy.

**Interviewer:** Why earlier or later than the 45^th^ day?

**Interviewee:** it is impossible before and after the 45^th^ day unless we will face health problems.

**Interviewer:** How often do they go to PNC?

**Interviewee:** we will go health facility on the 45^th^ day in first contact then subsequently based on their appointment till the date of the child's vaccine completed.

**Interviewer:** Do women think skilled attendance during postpartum help their babies and themselves?

**Interviewee:** all women think it is important.

**Interviewer:** Explain factors that would motivate women to utilize PNC services in their childbirth.

**Interviewee:** the health post, WDT, and 1 to 5 networks will motivate the women to use the PNC service.

**Interviewer:** other?

**Interviewee:** no

**Interviewer:** If women don’t go for PNC, what are their reasons?

**Interviewee:** home delivery is a reason that influences them not to go to a health facility.

**Interviewer:** Are there any financial and opportunity costs related barriers in your community?

**Interviewee:** many mothers will not go to health facilities due to financial problems. For example, they delay at home if they don't have money to pay for the motorcycle.

**Interviewer:** Are there any distance-related barriers in your community?

**Interviewee:** yes but the main one is finance. If you have money you will go even Addis Ababa. They remain at home without obtaining health care during the postpartum even for the child measles and skin diseases due to financial problems.

**Interviewer:** Are there any socio-cultural related barriers in your community?

**Interviewee:** no, nothing related to socio-cultural issues.

**Interviewer:** Are there any qualities of care-related barriers in your community?

**Interviewee:** no. They are providing the existing service.

**Interviewer:** Can you tell us about the traditional practices and beliefs during the postpartum period?

**Interviewee:** I don’t know any traditional practices. However, previously the mothers-in-law will prevent people not to visit the delivered women but now this tradition is stopped.

**Interviewer:** is there anything that provides for the infant before six months of age?

**Interviewee:** yes, some mothers will give the ‘Amesa' to their child before six months.

**Interviewer:** How do you see TBAs regarding PNC services provided to the community?

**Interviewee:** they are useless. We are using the advice and care of health professionals rather than TBAs.

**Interviewer:** In your opinion, what should be improved regarding PNC services?

**Interviewee:** as I have mentioned earlier. The government should avail the drug shortage and support those poor women who are unable to pay for drugs and services.

**Interviewer:** other?

**Interviewee:** healthcare providers should improve the quality of service.

**Interviewer:** other?

**Interviewee:** thank you for your information.

**IDI-02**

**Interviewer:** How early do women go for ANC?

**Interviewee**: thank you very much for your questions. Mothers will check their pregnancy after they become pregnant. They will use the TT vaccine and follow up on their ANC till the date of delivery. If there are any serious health problems meanwhile they will use a referral service. Finally, they will use a delivery service at the time of birth. Some mothers due to their attitudinal problems will not use ANC service.

**Interviewer:** after how many months of pregnancy they will start their first ANC visit?

**Interviewee**: between the 8^th^ and 9^th^ months

**Interviewer:** why do they go at that time (8^th^ month of pregnancy)?

**Interviewee:** they will go to a health facility at that time to check their health status.

**Interviewer:** Why do women go earlier or later than above stated time?

**Interviewee:** they will go to use the TT vaccine.

**Interviewer:** How often do they go to ANC?

**Interviewee:** they go to health facilities three times to use the TT vaccine.

**Interviewer:** Do women think skilled attendance during pregnancy helps their pregnancy?

**Interviewee:** they think it is important highly for the mothers.

**Interviewer:** for example, what do women think skilled attendance during pregnancy?

**Interviewee:** they think important to check their health status and their fetus.

**Interviewer:** Explain factors that would motivate women to utilize ANC services during their pregnancy.

**Interviewee:** the 1 to 5 networks and WDT leaders will motivate the women to go to health facilities to use ANC service. They counsel and teach the mothers about the benefits of ANC service for mothers and fetuses. The mothers will go to a health facility for ANC service.

**Interviewer:** other?

**Interviewee:** the HEWs also motivate mothers to use ANC service via home-to-home visits.

**Interviewer:** other?

**Interviewee:** sometimes the health professionals from the health center will motivate women.

**Interviewer:** If women do not go for ANC, what are their reasons?

**Interviewee:** they will go to the health center as per the counsel of the health professional.

**Interviewer:** are finance and opportunity costs related barriers that influence women not to use ANC in your locality?

**Interviewee:** no, because we have an ambulance service during the delivery time. An ambulance will take the referred mothers to the hospital and we haven't transportation problems currently.

**Interviewer:** Are distance-related barriers in your locality?

**Interviewee:** no, the distance isn't a big problem in our Balela 01 kebele.

**Interviewer:** are socio-cultural related barriers to your locality?

**Interviewee:** no, nothing socio-cultural related barriers. This disappeared after the community accepted the education provided through the different channels. However, previously there was a harmful traditional practice during childbirth. They suffocate the laboring women with wrong perceptions like the labor will become fast if the mother is deprived of air. This will create serious problems for the mothers and fetal health. But now mothers are giving birth at a health facility.

**Interviewer:** Are there any other harmful traditional practices in your area?

**Interviewee:** women circumcision. This will create serious problems during childbirth such as women's reproductive organ will not relax at the time of labor.

**Interviewer:** Are there any other harmful traditional practices in your area?

**Interviewee:** polygamous marriage

**Interviewer:** are qualities of care related to barriers in your locality?

**Interviewee:** no. The 1 to 5 networks will motivate and counsel the mothers to use the appropriate care.

**Interviewer:** Why do women go to the facility for the first ANC, but discontinue for subsequent ANC visits?

**Interviewee:** aren't any problems. They will obtain the service as per their appointment.

**Interviewer:** are finance and opportunity costs related to barriers in your locality?

**Interviewee:** no, because we are in 01 kebele the transport is not a big problem.

**Interviewer:** Are any traditional practice and belief-related barriers in your locality?

**Interviewee:** no, because the people in our community have obtained good education this doesn’t matter.

**Interviewer:** How do you see community volunteers regarding ANC services provided to the community?

**Interviewee:** They are motivating pregnant mothers to use the ANC service.

**Interviewer:** How do you see TBAs regarding ANC services provided to the community?

**Interviewee:** they don’t exist currently.

**Interviewer:** In your opinion, what should be improved regarding ANC services?

**Interviewee:** the government should avail the necessary thing to mothers based on the population size to avoid shortages. They should avail the FAFA and plumy nut for those under-nutrition mothers based on the population size.

**Interviewer:** what should be improved in the sides of your community?

**Interviewee:** the community should raise the idea and create awareness in government regarding the maternal health service.

**Interviewer:** what should be improved on the side of mothers?

**Interviewee:** the women should raise the idea in the area of service gap to the government about the maternal health service.

**Interviewer:** Do women think skilled attendance during childbirth helps them and their babies?

**Interviewee:** they think highly important because there is a difference between a child born at home and a health facility. They also provide the proper care of placenta delivery that will prevent abdominal cramps during the postpartum period. They are drugs, injections, and pills provided at health centers. They think it is very important for the mothers and their neonates.

**Interviewer:** Explain factors that would motivate women to utilize delivery services in their childbirth.

**Interviewee:** the education provided will motivate the women to go to the health facility.

**Interviewer:** other?

**Interviewee:** they will go to a health facility to attain their health status.

**Interviewer:** If women deliver at home, what are their reasons?

**Interviewee:** some mothers ashamed to give birth at a health facility

**Interviewer:** why are they ashamed to give birth at a health facility?

**Interviewee:** they have customs of a suffocate way of giving birth at home without any seeing their privacy. They say why I go to a health facility to give birth less privacy maintained place. They also raise the trend of their mothers and say why I could go to a health facility previously my mother delivered all of us home without any health problems. Some mothers ignored the education provided and give birth at home.

**Interviewer:** are finance and opportunity costs related to barriers in your locality?

**Interviewee:** no, most of the mothers are giving birth at home due to it is free of charge. The ambulance service is also free of charge during the referral time. They will obtain proper health care for themselves and their fetus.

**Interviewer:** are qualities of care related to barriers in your community?

**Interviewer:** Why do women go to the facility for ANC, yet mostly deliver at home?

**Interviewee:** the women who have hypertension will not give birth here but those mothers will refer to the hospital. Most of the time our mothers will not adhere to the counsel of health care providers. They refuse the referral and will give delivery at home. They will develop serious health problems.

**Interviewer:** Are there any mothers who refused the referral service?

**Interviewee:** yes, in rural areas.

**Interviewer:** Can you tell us about the religious beliefs during childbirth?

**Interviewee:** no, nothing in our community.

**Interviewer:** Can you tell us about the traditional beliefs during childbirth at home?

**Interviewee:** I don’t have any evidence about it.

**Interviewer:** How do you see TBAs regarding the health facility delivery services provided to the community?

**Interviewee:** after the establishment of new structures such as 1 to 5 networks and WDTs those TBAs disappeared.

**Interviewer:** In your opinion, what should be improved regarding facility delivery services?

**Interviewee:** I think the health professionals via community volunteers should motivate the mothers to use the service.

**Interviewer:** what should be improved on the sides of your government?

**Interviewee:** I think the government should motivate and support the health care providers.

**Interviewer:** what should be improved on the side of mothers?

**Interviewee:** 1 to 5 network leaders should motivate, counsel, and teach mothers to go to a health facility during labor.

**Interviewer:** How early do women go for PNC?

**Interviewee:** at the 45^th^ day of delivery.

**Interviewer:** why do they go on the 45^th^ day?

**Interviewee:** they will go to obtain child vaccination and family planning service.

**Interviewer:** Why earlier or later than the 45^th^ day?

**Interviewee:** the appropriate time is the 45^th^ day unless health problems are encountered. If it is after the 45^th^ day the vaccine service will delay for the child and there is a high probability of pregnancy.

**Interviewer:** How often do they go to PNC?

**Interviewee:** they will give the appointment for the next months for family planning and child vaccination service.

**Interviewer:** Do women think skilled attendance during postpartum help their babies and themselves?

**Interviewee:** yes, they think it is important.

**Interviewer:** Explain factors that would motivate women to utilize PNC services in their childbirth.

**Interviewee:** the community volunteers will motivate the women to use the PNC service.

**Interviewer:** other?

**Interviewee:** other like health professionals will motivate mothers.

**Interviewer:** If women don’t go for PNC, what are their reasons?

**Interviewee:** those mothers who have better nutritional status will give a chance for those who had more under-nutrition problems to obtain aid support in our community.

**Interviewer:** Are there any socio-cultural related barriers in your community?

**Interviewee:** no, nothing related to socio-cultural issues due to our community accepting the education provided at the earlier time.

**Interviewer:** Are there any mothers-in-law and fathers-in-law related barriers in your community?

**Interviewee:** no

**Interviewer:** Are there any financial-related barriers in your community?

**Interviewee:** no, because we are living here in town.

**Interviewer:** Are there any qualities of care-related barriers in your community?

**Interviewee:** no. They are providing quality service.

**Interviewer:** Can you tell us about the traditional practices and beliefs during the postpartum period?

**Interviewee:** I don’t know any traditional practices during the postpartum period.

**Interviewer:** In your opinion, what should be improved regarding PNC services?

**Interviewee:** I think the government and community should evaluate the service provided for mothers based on their proportion. If an additional service needs the government and community should avail it based on their needs.

**Interviewer:** what should be improved in sides of government?

**Interviewee:** the government should expand more services to women based on their needs.

**Interviewer:** what should be improved in the sides of a community?

**Interviewee:** the community should mobilize the women to obtain services.

**Interviewer:** what should be improved on the side of mothers?

**Interviewee:** the mothers should go to the health facility, follow and use services.

**Interviewer:** in your opinion, what should be improved for women to use the continuum of care?

**Interviewee:** the mothers should be mobilized and motivated to use services.

**Interviewer:** who is appropriate to mobilize the women?

**Interviewee:** the community volunteers

**Interviewee:** thank you for your information!

**Interviewee:** thank you!

**IDI-03**

**Interviewer:** How early do women go for ANC?

**Interviewee:** the women are using ANC service in our area. For example, in our kebele, we have 24 WDTs and leaders under HEWs. Due to this structure, we know all the pregnant women on our team. We counsel them to go to the health facility for an ANC visit and they will go. They use the TT vaccine. Some poor women may have anemia and we tell her to test at the health facility. She will receive a pill after health professionals tested her.

**Interviewer:** Why do women go later on for ANC visits?

**Interviewee:** in our kebele, we highly follow those mothers who become late due to we have responsibility. We counsel them about the bad health outcomes like bleeding and fetal distress that they may develop if they will not follow ANC.

**Interviewer:** Why do women go earlier on for ANC visits?

**Interviewee:** because they know the benefits of ANC service. If they remain at home, they will develop bad health outcomes like bleeding and fetal health problems.

**Interviewer:** If women do not go for ANC, what are their reasons?

**Interviewee:**  due to the cultural practice.

**Interviewer:** are finance and opportunity costs related to barriers in your locality?

**Interviewee:** yes, due to the financial problems women will not go to health facilities. Even if the service is free of charge at the health post the HEWs will send them to be investigated by the health center. During this time they will remain at home if they haven't transportation costs.

**Interviewer:** are qualities of care related to barriers in your locality?

**Interviewee:** no. we haven't experienced such a situation in the ANC service area.

**Interviewer:** Why do women go to the facility for the first ANC, but discontinue for subsequent ANC visits?

**Interviewee:** some due to lack of awareness and benefits of ANC service will discontinue the next appointment. Whereas those mothers who know the benefit of ANC will use the service properly until the date of delivery.

**Interviewer:** are finance and opportunity costs related to barriers in your locality?

**Interviewee:** yes previously but now the ANC service is free of charge and they will not consider it as a reason to discontinue the ANC service.

**Interviewer:** Are distance-related barriers in your locality?

**Interviewee:** yes the distance is a big problem previously but now due to the expansion of health facilities like health centers and hospitals in the Shebedino district it is not a big deal. Even though I have faced these problems previously but thanks to God these are not big problems. When I was in labor during the 2002 E.C. they referred me to Yirgalem Hospital. However, the health professionals at the hospital told me to stay there for nearly 13 days and due to the distance problem I decided to stay there with my family. We have also an ambulance service.

**Interviewer:** Are any traditional practice and belief-related barriers in your locality?

**Interviewee:** no, currently all mothers are wise due to the education given by health professionals.

**Interviewer:** How do you see community volunteers regarding ANC services provided to the community?

**Interviewee:**  we will counsel mothers after they give birth about maternal and child care. We will motivate the mothers to go to health facilities and receive child vaccination and family planning service.

**Interviewer:** How do you see community volunteers regarding ANC services provided to the community?

**Interviewee:**  we will meet with HEWs one time per month. I know all the pregnant women in my team members and report for the HEWs. The HEWs also will motivate pregnant mothers to go to health facilities for the ANC service.

**Interviewer:** In your opinion, what should be improved regarding ANC services?

**Interviewee:** thanks to God! The previous serious problems of poor pregnant mothers are now solved due to HEWs will screen and provide aid for under-nutrition pregnant women. I think this service should be much improved to support those mothers.

**Interviewer:** what should be improved on the sides of your health facility and health professionals?

**Interviewee:** previously there are poorly skilled health professionals and now this thing is corrected by different mechanisms. We have an ambulance driver's phone numbers we call immediately if women started signs of labor at night time. Currently, the service improved and mothers are receiving the proper care.

**Interviewer:** Explain factors that would motivate women to utilize delivery services in their childbirth.

**Interviewee:** Due to the proper care and drugs at health facilities they prefer to give birth there.

**Interviewer:** If women deliver at home, what are their reasons?

**Interviewee:** sometimes some mothers give home births due to the sudden onset of short labor after they followed the health facility for ANC service. However, the majority of them are following the ANC service and giving birth at health facilities.

**Interviewer:** other?

**Interviewee:** it is due to chance.

**Interviewer:** are finance and opportunity costs related to barriers in your locality?

**Interviewee:** yes previously but it is not a reason to give birth at home.

**Interviewer:** Are distance-related barriers in your community?

**Interviewee:** yes, during the summertime not all roads are accessible and suitable to carry women by motorcycle, animal-drawn cart, and ambulance.

**Interviewer:** Can you tell us about the traditional practices and beliefs during childbirth?

**Interviewee:** yes they will give ‘Amesa' for the infant previously but now stopped. The majority of traditional practices are now disappeared due to the health professionals' counsel during the health facility visit. The infant cord will bleed due to improper cord care during childbirths time. To prevent this bleeding they will apply animal dung and now this practice is also disappeared from our community. Currently, they are using health facility services and somewhat their health is improved.

**Interviewer:** How do you see community volunteers regarding the health facility delivery services provided to the community?

**Interviewee:** they are encouraging the mothers to give birth at a health facility.

**Interviewer:** How do you see TBAs regarding the health facility delivery services provided to the community?

**Interviewee:** we counsel and motivate mothers to go to health facilities during the delivery time by telling them about the harm of home delivery like excessive bleeding and fetal distress. We will teach them to give birth by skilled birth attendants.

**Interviewer:** other?

**Interviewee:** we inform those mothers who give birth at home for the HEWs. We and HEWs will follow those mothers as per our responsibility.

**Interviewer:** Do women think skilled attendance during childbirth helps them and their babies?

**Interviewee:** they will think and know birth pacing is very important for the mothers and child.

**Interviewer:** How do you see women's health care providers provided in this area?

**Interviewee:** HEWs will care, respect, and provide service for the mothers. They will counsel and refer to the health center if the case is beyond their capacity. They also assist them to complete their vaccination service.

**Interviewer:** How do you see husbands and community support for the delivering women in this area?

**Interviewee:** previously they will not support the women adequately and neglect them. However, now they perceive the women's problems as their own. They are cooperative with the mothers to use maternal health services at a health facility.

**Interviewer:** In your opinion, what should be improved regarding facility delivery services?

**Interviewee:** in my opinion, the services should improve much during the prenatal, childbirth, and postpartum period.

**Interviewer:** what types of service will provide for mothers during the postpartum period?

**Interviewee:** I counsel the mother will give breasts to a child at least 10 times per day, wash the child 3 times per day, and care for herself.

**Interviewee:** this is due to agreement and counseling with them. If we go to a health facility after the 45^th^ day the unwanted pregnancy will happen. We will go at that time to prevent unwanted pregnancy.

**Interviewer:** Do women know about the PNC service?

**Interviewee:** yes they know and they are happy to come here. Even the neighbors' women are happy to go to the health facility together with their mothers at 45^th^ and she will not go alone on that day.

**Interviewer:** If women don’t go for PNC, what are their reasons?

**Interviewee:** they will not go to health facilities due to a lack of awareness and knowledge of service benefits.

**Interviewer:** Are there any financial and opportunity costs related barriers in your community?

**Interviewee:** previously they complain the financial problems but now the services are provided free of charge.

**Interviewer:** Are there any distance-related barriers in your community?

**Interviewee:** yes they will not go health facility due to the distance.

**Interviewer:** Are there any socio-cultural related barriers in your community?

**Interviewee:** no,

**Interviewer:** Are there any qualities of care-related barriers in your community?

**Interviewee:** no.

**Interviewer:** How do you see families regarding PNC services provided to the community?

**Interviewee:** they are supportive. Some husbands will not support women.

**Interviewer:** In your opinion, what should be improved regarding PNC services?

**Interviewee:** for the future, the concerned body should improve the road and financial access to women to better improve the maternal health service.

**Interviewer:** other?

**Interviewee:** the government should support and strengthen the WDTs structure to improve the maternal health service.

**Interviewer:** other?

**Interviewee:** health professionals should improve the quality of care, respect, and appreciation for the mothers. Also, should be able to provide the appropriate services to all mothers without any discrimination.

**Interviewer:** anything that you want to add to our discussion?

**Interviewee:** I want to add in the drugs area. The drugs and supplies should be available all the time particularly for the women.

**Interviewer:** other?

**Interviewee:** the electricity and water supplies should be the biggest focus area to improve in our community.

**Interviewer:** thank you for your information!

**Interviewee:** thank you!

# IDI-04

**Interviewer: -** How early do women go for ANC? Why do you start ANC follow-up at six months of your pregnancy? Why do earlier or later?

**Interviewee: -** Women start ANC follow-up at six months of their pregnancy. I think that is the right time to start ANC follow-up. The health worker told us to start ANC follow-up at six months to vaccinate for tetanus.

**Interviewer: -** How often do you go to ANC? Why do you go at that time?

**Interviewee: -**In our area, we have visited four to five times for ANC services during pregnancy. We receive different services and information during ANC follows up from health workers; TT vaccine, and information about danger signs during pregnancy and delivery, they have to check the position of the pregnancy and whether it is at the right position or not.

**Interviewer: -** Do you think skilled attendance during pregnancy helps their pregnancy?

**Interviewee:** -Yes, skilled attendance during pregnancy is useful for pregnant mothers and for their pregnancy.

**Interviewer:** -What are the factors that would motivate women to utilize ANC service in their pregnancy in your area?

**Interviewee: -** Do you mean motivating factors in terms of people?

**Interviewer:** -Not only factors that are related to people but, you can mention any factors that motivate women to get ANC services in their pregnancy.

**Interviewee: -** During ANC follow up we have gate different services, like TT vaccination, MAMA/FAFA, and also health workers examining/checking our overall health status including our pregnancy status.

**Interviewer: -** What are the reasons that women do not go to health facilities for ANC services? What are the barriers to attending ANC services in health institutions?

**Interviewee:** - As I think there are no factors that make women not go to health facilities for ANC services to the health institutions but woman decides whether she goes to health facilities or not by themselves.

**Interviewer: -** Do you think that financial problems and opportunity of costs were barriers that women were not accessing ANC?

**Interviewee:** - As I think financial issues are associated with a lack of money to buy food for them when they feel hungry do women not utilize ANC service? On the other hand, some women think that they have paid for maternal health services and due to that reason, they are not going to health facilities for ANC services.

**Interviewer: -** What about distance and access to services?

**Interviewee:** - Yes, I think the distance from the health facilities affects ANC service delivery. It is difficult for pregnant mothers who live far from health facilities to go on foot and it requires more energy.

**Interviewer: -** How about socio-cultural-related factors?

**Interviewee: -**At earlier times there are different socio-culture-related factors that make women not use ANC services, but at this time there is no socio-culture-related factor in our area.

**Interviewer: -** Are general delivery of services and quality of care can affect the utilization of ANC services in your area?

**Interviewee: -**No, there is no problem related to the delivery of services and quality of care in health facilities.

**Interviewer: -** Why do women go to the facility for the first ANC, but discontinue for subsequent ANC visits?

**Interviewee: -**As I think women can decide by themselves whether they continue or discontinue the ANC services and there are no other external factors. But, sometimes due to financial and distance-related problems with transport and other access like food women discontinue ANC visits.

**Interviewer: -** Any factors related to socio-cultural and quality of care?

**Interviewee: -** No factors related to socio-cultural and quality of care for discontinuing ANC visits for pregnant mothers.

**Interviewer: -** Can you tell us about the traditional practices and beliefs during pregnancy?

**Interviewee: -** There are no known traditional practices and beliefs that were practiced during pregnancy in our area.

**Interviewer: -** How about religious practices?

**Interviewee: -** At the earlier time pregnant women believe that God can keep their health and no need for ANC services during their pregnancy but now no like these religious-related practices.

**Interviewer: -** How do you see community volunteers and health professionals and maternal health services provided to the community?

**Interviewee: -** As I think they are necessary for our community because they mobilize the community for maternal health services by giving all health education and information to our community.

**Interviewer: -** How do you see traditional birth attendants/TBAs/ in your area?

**Interviewee: -** At this time there are no Traditional birth attendants in our area because every woman goes to health facilities for delivery services.

**Interviewer: -** In your opinion, what should be improved regarding ANC services?

**Interviewee: -** After we come to health facilities we waited for a long period to get services and during that time we faced different problems; feeling tired, hungry, losing our time for our work,… So, waiting time for services should have to be improved.

**Interviewer: -** Do women think skilled attendance during childbirth helps themselves and their babies?

**Interviewee: -** Yes, because health professionals have assessed and given care to mothers and their newborn babies. For example, they have given necessary fluids to bleeding mothers, assessing and managing newborn babies with any difficulties or problems.

**Interviewer: -** What are the factors that would motivate women to utilize delivery services in their pregnancy in your area?

**Interviewee: -** In our area, there are community mobilizer women that motivate or mobilize women not to deliver at their homes.

**Interviewer: -** Ok, what are other additional factors that increase the interest of women to utilize delivery services?

**Interviewee: -** I don’t know other reasons.

**Interviewer: -** What are the reasons that women deliver at their homes?

**Interviewee: -** In our area, no women have delivered at home and I don't know other reasons why women deliver at home in other areas.

**Interviewer: -** Do you think that women deliver at home due to financial problems and opportunity costs?

**Interviewee: -** As I think due to the lack of towels for newborn babies and clothes that she wears after delivery some women decided to deliver at home.

**Interviewer: -** How about distance and access?

**Interviewee: -** As I think distance and access to transport were not a reason for home delivery because women can use different types of transport in addition to ambulances. Even if husbands have no money for transport fees they have borrowed from others.

**Interviewer: -** Socio-cultural related factors can make women deliver at home?

**Interviewee: -** No culture that makes home delivery at home in our area.

**Interviewer: -** Quality of care and non-dignified care can be a reason for home delivery.

**Interviewee: -** No, they have given good care for all mothers, especially for delivering mothers, delivery room was also well cleaned including the delivery coach, and also health professionals keep the dignity of the women.

**Interviewer: -** Why do women go to the facility for ANC, yet mostly deliver at home?

**Interviewee: -** Sometimes due to network failure and related factors to dial an ambulance driver or other concerned bodies at the time of labor women deliver at home.

**Interviewer: -** Financial barriers and opportunity costs, distance and access, socio-cultural, quality of care, and non-dignified care can be the factors that women deliver at home after she has gotten the services in a health facility.

**Interviewee: -** As I think these are not factors for women to deliver at home after she has gate ANC services in health facilities in our area.

**Interviewer: -** Can you tell us about the traditional practices and beliefs during childbirth?

**Interviewee: -** No traditional practices and beliefs during childbirth in our area.

**Interviewer: -** Do you think religious practices and cultural norms affect mothers to use care during delivery?

**Interviewee: -** As I think there are no religious practices and cultural norms that affect mothers' use of care during delivery.

**Interviewer: -** How do you see community volunteers/TBAs services provided to the community?

**Interviewee: -**They mobilize and give information for the community not to deliver at home.

**Interviewer: -** And also how do you see health professionals and maternal health services provided to the community?

**Interviewee: -** As I have mentioned before the services provided by the health professionals for women were good and they give all services which we want.

**Interviewer: -** In your opinion, what should be improved regarding facility delivery services?

**Interviewee:** - The service is good and I have no other points to say, but we accept that if good things are done.

**Interviewer:** After delivery, how early do women go to a health facility to check the health of themselves and their newborn babies (PNC)?

**Interviewee: -** Women go to a health facility 42nd days after delivery.

**Interviewer: -** Why do they go at that time?

**Interviewee: -** Women come to health facilities on the 42nd day of deliver to get family planning for themselves and vaccinate their child.

**Interviewer: -** Can she come earlier or later?

**Interviewee: -** No, she cannot come before or after the 42nd day of delivery.

**Interviewer: -** How often do they go to PNC services?

**Interviewee: -** Women go to health facilities for PNC services until their children were fully immunized and on the way that she gate family planning services.

**Interviewer: -** Do women think skilled attendance during postpartum help their babies and themselves?

**Interviewee: -** Yes, they think that skilled attendance during postpartum help their babies and themselves in our area.

**Interviewer: -** What are the factors that would motivate women to utilize PNC services in their childbirth?

**Interviewee: -** Vaccinating her child and getting FAFA until six months after delivery.

**Interviewer: -** What are the barriers to PNC service utilization in your area?

**Interviewee: -** If women deliver at home, she cannot get FAFA and she doesn’t come to health facilities.

**Interviewer: -** If women deliver at home, she doesn’t gate FAFA?

**Interviewee: -** Yes, she doesn’t gate.

**Interviewer: -** What are the other factors for not using PNC services?

**Interviewee: -** No other factors for not utilizing PNC services in our area.

**Interviewer: -** What traditional beliefs, religious practices, and cultural norms affect mothers to use care during the postpartum period?

**Interviewee: -** There are no religious and culture-related factors that affect the utilizing PNC services in our area.

**Interviewer: -** In your opinion, what should be improved regarding PNC services and the Continuum of care?

**Interviewee: - ………………….**

**Interviewer: -** I have finished my questions. Thank you.

**IDI-05**

**Interviewer: -** How early do women go to health facilities for ANC services when they recognize their pregnancy?

**Interviewee: -** Women go to health facilities after seven months of their pregnancy to gate ANC services. During ANC follow up women examined for the positions of their pregnancy, tests for syphilis (SHUFURO), and health professionals give counseling about the foods that they need to feed during pregnancy; carrots, potatoes, and…. body-building foods. Health professionals also give maternal health education so women to go to health facilities for skilled delivery services. After delivery, women go to health facilities for PNC services after 45^th^ days of delivery to receive immunization services for their child and family planning services for themselves. At the earlier time, many women were exposed to different health problems due to not taking immunization for their children and family planning services for themselves. But, at present time these kinds of the problem has improved and no women who deliver at home in our area.

**Interviewer: -** How often do pregnant women go to health facilities for ANC services in your area?

**Interviewee: -** Women go to health facilities for ANC services five times until they deliver and services are provided on Monday and Thursday at the health center level.

**Interviewer: -** Do women think skilled attendance during pregnancy helps their pregnancy in your area?

**Interviewee: -** Yes, women think that skilled attendance during pregnancy is important and they used this service. Women receive different services like FAFA in addition to medical services, skilled delivery services, and also plumpy nuts for their child if it is malnourished.

**Interviewer: -** What are the reasons that women do not go to health facilities for ANC services?

**Interviewee: -** Women who have no soap to wash their clothes and do not wear clothes due to lack of money fear going outside of their homes and they do not go to health facilities for ANC services.

**Interviewer: -** Do you think that due to distance and access women do not go to health facilities for ANC services?

**Interviewee: -** Yes, women who live far from health facilities need to pay money for transport services like motorcycles and those who have no money do not go to a health facility for ANC services. So, due to distance and lack of money women do not go to health facilities for ANC services.

**Interviewer: -** How about socio-cultural-related factors?

**Interviewee: -** No socio-cultural related factors but the main factors are financial-related problems.

**Interviewer: -** Are general delivery of services and quality of care can affect the utilization of ANC services in your area?

**Interviewee: -** No service quality-related factors in the health facilities that do women not to go ANC services.

**Interviewer: -** Why do women go to the facility for the first ANC, but discontinue for subsequent ANC visits?

**Interviewee: -** As I think some women go to health facilities by their foot for the first time and but when their gestational age increased they cannot go by foot and due to this reason they discontinue subsequent ANC visits.

**Interviewer: -** What kinds of traditional practices and beliefs during pregnancy are in your area?

**Interviewee: -** No traditional practices that affect the utilization of ANC services in the facility.

**Interviewer: -**Are there religious practices that make women not go to a health facility for ANC services?

**Interviewee: -** As I think some women think that God can keep their health and saves their life and there is no need to go health facility for ANC services.

**Interviewer: -**How do you see community volunteer services provided to the community?

**Interviewee: -** In our area community mobilizers do mobilize the community women to go for ANC services.

**Interviewer: -** Are there traditional birth attendants in your area?

**Interviewee: -** There are no traditional birth attendants in our area.

**Interviewer: -**How do you see services provided by the health professionals to the community?

**Interviewee: -** Health professionals give maternal health education to women to go to health facilities for ANC services and they give quality services to women.

**Interviewer: -** You told me that you have recently delivered at a health center and attended ANC services during your pregnancy. During your ANC follow up how many times you have visited for this service?

**Interviewee: -** I have attended ANC visits five times during my pregnancy. I have gate different services during my ANC follow-up; health professionals examine the position of my babies and the overall health of my pregnancy. I received delivery services in the health facility during my childbirth.

**Interviewer: -** How do you rate the quality of care you received during ANC follow-up? Are you satisfied?

**Interviewee:** - I received quality care during my ANC follow-up and I have satisfied with the services.

**Interviewer: -** What are the factors that motivate you to utilize ANC service in your pregnancy?

**Interviewee: - C**ommunity volunteers have told me to go health facility for ANC services. They also identify pregnant women in the community and do these women go to health facilities for ANC services. On the other hand, after I have received delivery services by skilled birth attendants I have also done women go to health facilities by telling them the importance of ANC follow-up.

**Interviewer: -** Is there support that you get from the community to receive maternal health services during pregnancy?

**Interviewee: -** I have no support from the community during my pregnancy that enforces me to use ANC services.

**Interviewer: -** What is your experience relating to the utilization of ANC care provided by skilled birth attendants?

**Interviewee: -** Health professionals respect me in a good way and the services provided to me by them are also good.

**Interviewer: -** Do you have confidence in skilled birth attendants’ abilities?

**Interviewee: -** Yes I have confidence in skilled birth attendants' because they have good skills and abilities regarding their work.

**Interviewer: -** Do health professionals respect and have compassion for attendants (respect for the traditional beliefs of the women, etc)

**Interviewee: -** As I have seen health professionals give respectful and compassionate care for women and also they respect the beliefs of the women.

**Interviewer: -** Do women think skilled attendance during childbirth helps themselves and their babies?

**Interviewee: -** Yes, women think skilled birth attendance during childbirth helps them and their babies.

**Interviewer: -** What kind of services do women receive during childbirth?

**Interviewee: -** Skilled health professionals attend childbirth and manage any difficulties related to the delivery processes also after childbirth, they clean and remove the remaining blood from the woman's uterus.

**Interviewer: -** Explain factors that would motivate women to utilize delivery services in their pregnancy and reasons for using a continuum of care.

**Interviewee: -** Pregnant women are meeting or conference in the kebele to increase the awareness of pregnant women. Community mobilizers also give education for women not to deliver at home by explaining what kinds of problems would happen for women when she deliver at home. In overall, awareness of the women in our community increased due to continuous maternal health educations.

**Interviewer: -** Some women deliver at home and what are their reasons to deliver at home?

**Interviewee: -** Some women deliver at home due to lack of water and soap to wash their clothes and their bodies. They do not want to go health facilities by wearing unclean and dirty clothes and they afraid to open unclean bodies in front of health professionals. Due to this reason some women need to deliver at home. This is related to the financial problems of the family.

**Interviewer: -** Do you think that due to distance and accesses women deliver at home?

**Interviewee: -** Yes, at sometimes women who lives far from health facility deliver at home until ambulance reaches to their home.

**Interviewer: -** Why do women go to the facility for ANC, yet mostly deliver at home?

**Interviewee: -** Most time women deliver at home after they have received ANC services due to lack of money to buy soap to wash their clothes and bodies, to pay for transport services if ambulance doesn’t arrive and lack of clothes to wear.

**Interviewer: -** Do quality of care and non-dignified care reasons for deliver at home after they have attended ANC services?

**Interviewee: -** Yes, because if there is problem in quality of the care women do not want to go health facility for deliver services.

**Interviewer: -** Can you tell us about the traditional practices and beliefs during childbirth?

**Interviewee: -** During home delivery they do wait for long period of time until she has deliver. Women wait long period of time for placenta removal by giving milk and water by thinking that she can gate power to push and it will be removed. On other hand they cut cord inappropriate way or size and leads to bleeding.

**Interviewer: -** What are the religious practices that affect mothers to use care during delivery?

**Interviewee: -** Some women do not go to health facilities during labor by believing that God speaks for me by prophet and nothing will be happen on me during delivery time. They said that the man of God tells me that I will deliver at home without any difficulties. So, they do not want to go health facility during labor for delivery services.

**Interviewer: -** How do you see maternal health services that provided by health professionals?

**Interviewee: -** The services that provided by the health professionals for women in the health facility during deliver is good.

**Interviewer: -**In your opinion, what should be improved regarding facility delivery services?

**Interviewee: -** In my opinion assigning one strong women who can lead and preparing continuous and weakly based pregnant women meeting is needed in the kebele. Health extension workers have to give additional maternal health educations for pregnant women and to create common consents not to deliver at home. It is important that making strong linkages with kebele administrative bodies to identify women those who deliver at home and doing to not deliver at home. On other hand, it is good if government provide other goods that needed for women that can increases the interest of women to go for services.

**Interviewer: -** How early do women go for PNC services after delivery?

**Interviewee: -** Women go to health facilities for PNC services at 45^th^ days after delivery.

**Interviewer: -**Why do they go to health facilities at 45^th^ days after delivery? Why earlier or later?

**Interviewee: -** Women will go to health facilities 45 days after delivery to get family planning for themselves and immunizations for their children. She doesn’t go before or after the 45^th^ day after delivery.

**Interviewer: -** How often do women go to PNC services?

**Interviewee: -** After women go to health facility for PNC services at 45^th^ days of deliver then they returned to back for services according to their appointments.

**Interviewer: -**Do women think skilled attendance during postpartum helps their babies and themselves?

**Interviewee: -** Yes, health professionals give counseling for women how to feed their child and what types of food they need have to eat and immunization services .

**Interviewer: -** What are the factors that would motivate women to utilize PNC service in their childbirth?

**Interviewee: -** Health extension workers motivate women to go for PNC services and also women health development army leaders also mobilize the women to go for PNC services.

**Interviewer: -** Some women do not go for PNC services and what are the reasons that do women don’t go for PNC services?

**Interviewee: -** Some women do not go for PNC services due to lack of money to buy the child’s clothes, soaps, and other financial related problems. Due to distances and access women do not go for PNC services.

**Interviewer: -** What types of traditional practices and beliefs that do during postpartum period?

**Interviewee: -** Husbands who have no formal educations and knowledge do not care for women do sex with her after three days of deliver without keeping maintain her body health. When the newborn child is female husband do sex with her at three days of delivery and if the newborn child is male he do sex with her at fourth days of delivery this practice is dangerous for health of women.

**Interviewer: -**In your opinion, what should be improved regarding PNC services to maintain continuum of care?

**Interviewee: -** As I have mentioned before many women faced different problems; lack necessary foods that maintain their body, lack of money to buy soaps, lotions and clothes for their child. So, governments should have to like these services for women after delivery.

**IDI-06**

**Interviewer:** how early do women go for ANC? Why do they go at that time? Why earlier or later? How often do they go to ANC? Why do they go at that time?

**Interviewee: y**es they come when pregnant mothers reach four months they come to the facility and receive ANC care and further receive their TT vaccines and follow up their care until birth.

**Interviewer:** among the women who attend the ANC care properly what do you think are the factors that motivate women to use ANC care?

**Interviewee:** the work that the community organizations and the health extension workers do to improve the awareness of the women in the community.

Interviewer: If women don’t go for ANC, what are their reasons?

**Interviewee:** some husbands have multiple wives and are much older than their wives and those husbands refuse to send them to the facility.

**Interviewer:** what about other barriers like financial and distance barriers?

**Interviewee: -** some say we don't have any money to pay. But we convince them all these services are free. We tell them of the benefits. We also see some who say the road is far but we tell them they will use ambulances during birth. These things aren't major barriers. Most of the attitudes are changing.

**Interviewer:** Why do women discontinue subsequent ANC visits?

**Interviewee: -** Sometimes they tell of their household and community life reasons. Others say nothing major service has been given during their care. When we see defaulters we convince them and motivate them again.

**Interview:** can you tell us about the traditional practice and beliefs during pregnancy?

**Interviewee: -** we recently found out in the remote part of our catchment some women have been performing FGM on girls. We went out there we identified the women and we called the police and the police arrested them.

**Interviewer:-**How do you see the role of community volunteers/TBAs, and health extension workers on ANC care service delivery?

**Interviewee: -** We have been working on improving awareness and motivating the mothers to attend ANC care. We work in collaboration with health extension workers

**Interviewer:** what should be done to further improve ANC care delivery?

**Interviewee: -** community organization ought to be strengthened. Although we try our best, our organizations are not going as sturdy as when we began. They are not giving us suitable support as before. They don't give us refresher training. If we receive proper support could be done there are things that we can ant to do. We want to do more.

Interviewer:-My next question is about facility delivery. Do mothers know the benefits of facility delivery in your area?

**Interviewee: -** Based on our motto that "no mother shall die while giving life" we motivate mothers to deliver at the facility. When I see the term women we warn them of delivering at home. We tell them about complications of home birth. The mothers currently understand the benefits and want to deliver at the facility.

**Interviewer:-**for mothers who deliver at home, what are their reasons?

**Interviewee: -** mostly it is due to some women who are pregnant out of wedlock. They refuse to present their child and themselves to the facility. Mostly these are the scenarios and lack of full awareness is the cause of home delivery.

**Interviewer: -** Do women present a reason for the lack of transport or lack of money as a reason for home delivery?

**Interviewee: -** No

**Interviewer:-**How do you see the job being done by the community health development army specifically about facility delivery?

**Interviewee: -** When I see the term women we warn them of delivering at home. We tell them about complications of home birth. The mothers currently understand the benefits and want to deliver at the facility. The others have also been the same as far as I know.

**Interview:** can you tell us about the traditional practice and beliefs during delivery?

**Interviewee: -** In the past older women will say that delivering mothers shouldn’t go out in the open. And used to de-motivate facility birth but that has been conquered recently.

**Interviewer:-**What do you think should be done to improve facility delivery as well as the continuum of care?

**Interviewee: -** enduring the health education and strengthening the service. Doing everything we can to improve the quality of the services.

**Interviewee: -** We go and visit the mothers when they are sent home after delivering at the facility. I give education on my part that the mother should breastfeed the baby up to ten times daily, that the mother should exclusively breastfeed the baby for six months

**Interviewer**: - Do mothers understand the benefit of PNC service to themselves and their newborns?

**Interviewee: -** most of them understand when the time for vaccination comes they come here happily and vaccinate the baby.

**Interviewer: -** if mothers don’t use PNC care what do you think their reasons are?

**Interviewee: -** not having adequate awareness. if mothers are aware they always come. I don't think money and access are issues for not using. Maybe the roads are not comfortable during winter. Some families may not support this that but these things are rarely seen.

**Interviewer:** - What do you think should be done to improve PNC care as well as the continuum of care?

**Interviewee: -** we have the basic working system in place. That we need to strengthen our activities and the government should support our efforts. They should support us. In terms of quality of care, there can be improvement in supplying all the necessary supplies. Sometimes essential medicines are missing. We also have problems with water and electricity I think these issues can be improved I think we will improve the health care given to mothers.

**Interviewer: -** I have finished my questions, thank you.

**IDI-07**

**Interviewer:** how early do women go for ANC? Why do they go at that time? Why earlier or later? How often do they go to ANC? Why do they go at that time?

**Interviewee: -** when mothers arrive first in the facility they will be tested for HCG and when it is known that they are pregnant they will be instructed to begin ANC care. After that, she will be told all the free services given until delivery. They are urged to follow all the ANC services and finally, they are told to deliver in the facility.

**Interviewer:** Why are the pregnant mothers late for ANC care?

**Interviewee: -** lack of awareness is why the mothers are late. But now we can say their attendance is improving.

**Interviewer:** among the women who attend the ANC care properly what do you think are the factors that motivate women to use ANC care?

**Interviewee: -** where the community orientations are strong the maternal motivations are strong for attending ANC care. That is because they have received continuous health education to change their awareness of ANC care.

**Interviewer:** If women don’t go for ANC, what are their reasons? what about other barriers like financial and distance barriers?

**Interviewee:** - lack of knowledge and awareness. Because the services are free and they can't present a lack of money as a reason. The road infrastructures are better in our area Since we are near Morocho town. Access and monetary problems can't be presented as a barrier in our catchment.

**Interviewer:** why do women discontinue subsequent ANC visits?

**Interviewee:** - **as** I have said before it is a lack of knowledge.

Interview: can you tell us about the traditional practice and beliefs during pregnancy?

**Interviewee:** -In the past, the mothers-in-law who are of the older generation refused that their daughter in laws shouldn't show their private parts to male professionals and used to prefer home delivery at the hands of traditional birth attendants. We worked on that and we were able to reverse that. In the past, these older mothers used to apply butter on the navel and feed the newborn butter and they feed additional food to babies below 6 months. They are also used to remove the maternal colostrum breast milk. Female genital mutilation was present in our area and when the children were ill with tonsillitis traditional healers are used to remove the tonsils with non-sterile metal. These were the traditional practices in our area but thanks to the effort of community organizations we were also to reverse these habits and they have been largely eliminated.

**Interviewer:-**How do you see the role of community volunteers/TBAs, and health extension workers on ANC care service delivery?

**Interviewee:** - **i**t has been essential in improving the awareness of the mothers to utilize modern services. Especially in person face-to-face health education works according to my experience. When you teach them through the radio some people don't have it and may miss the program. But in person, continuous health education has been cutting the deal for us in our area.

**Interviewer:** what should be done to further improve ANC care delivery?

**Interviewee:** - renewing and refreshing community organizations are very important. They have been the agent of change in our area.

Interviewer:-My next question is about facility delivery. Do mothers know the benefits of facility delivery in your area?

**Interviewee:** - yes they are delivering in the facility. Sometimes some mothers when they have fast labor deliver in no time and may fail to reach the facility. For example, Tadu who is my coir mate was heavily pregnant and continued to sing in Sunday service church. But when the service was over she went into labour and delivered at her father's home then I called an ambulance and the placenta was delivered at a health facility. What I mean to say is most mothers do deliver in the facility the exception being some mothers not being prepared for birth are the ones delivering at home.

**Interviewer:-**for mothers who deliver at home, what are their reasons?

**Interviewee:** - in the past, the mother in laws refuses to bring the mother to the facility. But that has now changed greatly. As I have said before home deliveries are linked with a lack of birth preparedness and accidental labor.

**Interviewer: -** Do women present a reason for the lack of transport or lack of money as a reason for home delivery?

**Interviewee: -** no in the past they may but currently all the MCH services are for free.

**Interviewer:-**How do you see the job being done by the community health development army specifically about facility delivery?

**Interviewee:** - we have been doing a great deal of a job in our capacity. But we need support from the authorities.

**Interviewer:-**What do you think should be done to improve facility delivery as well as the continuum of care?

**Interviewee:** - we have to strengthen our effort and improve our community organizations. And as I have said before e need to improve the infrastructure of our facility interims of water and power supply. We have a power problem in the facility and the mothers complain of having to deliver in the dark. The professionals work around the clock to deliver the mothers if these could be improved with automatic generators and other power options we can deliver quality service.

**Interviewer:-**What does PNC service look like in your facility?

**Interviewee:** -**-**the mothers after they have deliveries ask to go home**.** But advised to stay In the facility and receive their care. after the mothers are sent home we go to their homes. We see if there are any complications we teach them about hygiene and sanitation. We also teach them o breastfeeding as well as maternal nutrition.

**Interviewer: -** Do mothers understand the benefit of PNC service to themselves and their newborns?

**Interviewer: -** If mothers don’t use PNC care what do you think their reasons are?

**Interviewee: -** The mothers who deliver at home are the ones who miss out on PNC.

**Interviewer: -**How do you see the job being done by the community health development army and TBA specifically about PNC? What do you think should be done to improve PNC care as well as the continuum of care?

**Interviewee: -** as I said before after the mothers are sent home we go to their homes. We also teach them o breastfeeding as well as maternal nutrition. But I understand we need re-strengthening.

**Interviewee:** -most mothers understand the benefits but some don't

**Interviewer:** - I have finished my questions, thank you.

# IDI-08

**Interviewer: -** How early do women go to health facilities for ANC services when they recognize they are pregnant in your area?

**Interviewee: -** Women go to a health facility for ANC services after five months of their pregnancy.

**Interviewer: -**When can we say women go to a health facility earlier or later for ANC services?

**Interviewee: -** We say later if women go to health facilities after five months of pregnancy and earlier if pregnant women go to health facilities before five months of pregnancy.

**Interviewer: -** How often do pregnant women go to health facilities for ANC services in your area?

**Interviewee: -** Women go to health facilities three times for ANC services during pregnancy.

**Interviewer: -** What types of services do health professionals give to women during ANC follow-up?

**Interviewee: -** During ANC follow up skilled health professionals do an examination of pregnant women about the overall health of pregnancy and mothers, TT vaccine three times until delivery, and drugs in tablet forms that prevent a deficiency of red blood cells.

**Interviewer: -**Do women think skilled attendance during pregnancy helps their pregnancy?

**Interviewee: -**Yes, because ANC follow-up skilled health professionals do different examinations and tests to assess the health of women and their pregnancy; measuring blood pressures of women, asses overall conditions of pregnancy by using ultrasound in hospitals, …………

**Interviewer: -** What are the factors that would motivate women to utilize ANC services during their pregnancy?

**Interviewee: -** As I think the health-seeking behavior of women increased and they need to take the TT vaccine and check their health and pregnancy.

**Interviewer: -** What are the reasons that women do not go to health facilities for ANC services?

**Interviewee: -** As I think the reason that women do not go to health facilities for ANC services is a lack of knowledge of women about the importance of services.

**Interviewer: -** Do you think that due to financial problems and distance and access women do not go to health facilities for ANC services?

**Interviewee: -** Some women do not go to a health facility for ANC services due to financial problems but distance is not a factor because health facility is found in short distance.

**Interviewer: -** How about socio-cultural-related factors?

**Interviewee:** - As I think there are no socio-cultural related factors that do women not go to health facilities for ANC services in our area.

**Interviewer: -** Why do women go to the facility for the first ANC, but discontinue for subsequent ANC visits?

**Interviewee: -**Women discontinue subsequent ANC visits when they think their pregnancy is in good health condition with no need for ANC follow-up.

**Interviewer: -**Are there financial problems and distance and access-related factors that do women discontinue without completing ANC visits after they have received some ANC services?

**Interviewee: -** As I think there are no financial and distance-related factors that do women discontinue ANC visits in our area.

**Interviewer: -**How about socio-cultural and quality of care-related factors?

**Interviewee: -** No socio-cultural-related factors in our area.

**Interviewer: -** Are there quality of care-related factors that women do not go to health facilities for ANC services?

**Interviewee: -** No, because the services for pregnant mothers in the health facility are good and no women complain about service quality-related issues.

**Interviewer: -** Can you tell us about the traditional practices and beliefs during pregnancy in your area?

**Interviewee: -** No traditional practices and beliefs that applied during pregnancy in our area.

**Interviewer: -** Are there religious practices that affect mothers' use of care during pregnancy?

**Interviewee: -** No religious practices that affect mothers to use ANC services during pregnancy.

**Interviewer: -** What types of services community volunteers provided to pregnant women in your area?

**Interviewee: -** No community volunteers in our area.

**Interviewer: -** How do you see health professionals and maternal health services provided to the community?

**Interviewee: -** Skilled health professionals provide three doses of TT vaccine during ANC follow up and there are no other services that they provide for pregnant mothers. As I think, our community is satisfied with the services provided by health professionals in the health facilities.

**Interviewer: -** Do health professionals give respectful and compassionate care to pregnant mothers in the health facility?

**Interviewee: -** As have seen health professionals give respectful care by showing a good approach to the service-taker mothers without showing boredom until they finish their work and also they keep the dignity of the women during the service-giving time.

**Interviewer: -**How do the communities see the maternal health services provided to the community?

**Interviewee: -** As I think our community is satisfied by the services provided by the health professionals in the health facility.

**Interviewer: -** In your opinion, what should be improved regarding ANC services?

**Interviewee: -** I have no points to raise that need to be improved regarding ANC services in our area. Because the services provided for women are holistic and attractive and necessary services in the health facility including electricity and water available in the facility. Health professionals are also available at all times including lunch and nighttime, so women can gate services at all times.

**Interviewer: -** What are the services that skilled health attendance gives to women during childbirth for themselves and their babies?

**Interviewee: -** As I think during childbirth women receive different services for themselves and their babies. After women reach health facilities by seeking delivery services, skilled health professionals do examinations and attend the delivery, and give good care to mothers and newborn babies. After delivery health professionals give TTC ointment, drugs in the form of drops that give through the mouth for the newborn babies, and give drugs for the mother as it is needed (drugs for abdominal cramps…).

**Interviewer: -**What are the factors that would motivate women to utilize delivery services during their pregnancy?

**Interviewee: -** Health extension workers mobilize the women not to deliver at home by preparing pregnant women conferences in the kebele and giving continuous maternal health education for women. Women are exposed to different problems when they deliver at home, i.e. prolonged labor, and high bleeding …) so, women choose to deliver at health facilities.

**Interviewer: -**If women deliver at home, what are the reasons and constraints that influenced women to utilize facility delivery services?

**Interviewee: -** As I think some women deliver at home when their home far from the health facility and if they don't know the phone number of the ambulance driver to dial during labor. In addition to this, some women deliver at home due to lack of enough time when their labor is acute and fast and if they deliver immediately after the onset of labor.

**Interviewer: -** Are there socio-cultural related factors that women deliver at home?

**Interviewee: -** No socio-cultural related factors that women deliver at home in our area.

**Interviewer: -** Why do women go to the facility for ANC, yet mostly deliver at home?

**Interviewee: -** As I think some women deliver at home after they have received ANC follow-up at the health facility if labor is acute and deliver in a short period.

**Interviewer: -** Are there socio-cultural related factors?

**Interviewee: -** No socio-cultural related factors in our area that do women deliver at home.

**Interviewer: -** How about quality of care and non-dignified care?

**Interviewee: -** Skilled health professionals give quality and dignified care to women during delivery and have no quality-related complaints.

**Interviewer: -** Can you tell us about the traditional practices and beliefs during childbirth?

**Interviewee: -** No traditional practices and beliefs during childbirth time in our area. But, I have heard traditional practice during childbirth was women doing to wear many blankets and closing doors and windows of the house during labor until they give birth.

**Interviewer: -**Are there religious practices that are related to the home delivery of women?

**Interviewee: -** As I think some women think that God can help them to deliver at home without going to a health facility and decided to deliver at home.

**Interviewer: -** What kinds of services do community volunteers provide to the women during delivery?

**Interviewee: -** At sometimes community volunteers inform health extension workers about ambulance services if there is a laboring mother in our area and no other services that community volunteers provide for women during delivery.

**Interviewer: -** How do you see services that skilled health professionals provide to women during delivery?

**Interviewee: -** As I think health professionals provide different services for women and newborn babies during childbirth and our community looks at them in a good way. They provide respectful and compassionate care for delivering mothers and our community is satisfied by the services that are provided for them.

**Interviewer: -** How do the communities see the maternal health programs and health professionals? Tell me your perception of maternal health care services and your perception of different care providers.

**Interviewee: -** At present our community's knowledge, attitude, and practice to use maternal health services are changed they started to use all maternal health services and they are satisfied with the services provided by the health professionals.

**Interviewer:** -What efforts has your community made to increase maternal health service in your community?

**Interviewee: -** Mobilizing the women not to deliver at home by giving necessary support.

**Interviewer: -**In your opinion, what should be improved regarding facility delivery services?

**Interviewee: -** As I think the services provided by health professionals are good, they actively follow women during labor until they give birth, and the service-giving room including the bed of the waiting room also clean. But, in the Leku general hospital sometimes health professionals are ordered to buy drugs from outside of the hospital and this should be improved. After women go to the hospital with referral sheets from other health centers they do not give services immediately for women who have no ANC follow-up in the hospital previously.

**Interviewer: -** How early do women go for PNC services after delivery?

**Interviewee: -** Women go to health facilities for PNC services at 40^th^ days after delivery.

**Interviewer: -** When we say women go to health facilities earlier or later after delivery?

**Interviewee: -** We say earlier when women go to health facilities for PNC services before 40^th^ days of delivery and later if it is after 40^th^ days of delivery.

**Interviewer: -** What kinds of services do health professionals provide for PNC services in your area?

**Interviewee: -** Health professionals provide family planning services for women and immunization services for their babies during PNC visits.

**Interviewer: -** What are the factors that would motivate women to utilize PNC services in their childbirth in your area?

**Interviewee: -** Women do not want to become pregnant during the postpartum period and they need to take family planning services to control unwanted pregnancy and also they need to do their child take immunization services.

**Interviewer: --** If women don’t go for PNC, what are their reasons?

**Interviewee: -** As I think some women do not go for PNC services due to the distance from the health facility.

**Interviewer: -** Are there any other factors related to socio-culture that do women do not go to health facilities for PNC services?

**Interviewee: -** There are no socio-cultural related factors related to the utilization of PNC services.

**Interviewer: -** Are there any other factors related to financial problems do women do not go to health facilities for PNC services?

**Interviewee: -** Yes, some women do not go to health facilities for PNC services due to financial problems.

**Interviewer: -** Can you tell us about the traditional practices and beliefs during the postpartum period?

**Interviewee: -** At the earlier time women didn't give first milk (colostrum) they discard it and also they give AMESA to their newborn babies. But now there are no traditional practices and beliefs during the postpartum period in our area.

**Interviewer: -**What are the religious-related practices that affect mothers to use care during the postpartum period?

**Interviewee: -** There are no religious practices during the postpartum period except putting Bibles at the head side of babies.

**Interviewer: -** What kind of maternal health services that community volunteers and health professionals provide to the women after delivery?

**Interviewee: -** Some services are provided by the community volunteers for the women after delivery. On the other hand health extension workers provide immunization for children, identify defaulter and provide immunization.

**Interviewer: -**How do the communities see the maternal health programs and health professionals? Tell me your perception of maternal health care services and your perception of different care providers.

**Interviewee: -** Health professionals provide respectful and good care for women and I think our communities are satisfied by the services that are provided for women and their children.

**Interviewer: -** In your opinion, what should be improved regarding PNC services?

**Interviewee: -** Services that health professionals provide for women and children in hospitals, health centers, and health post level are good and I have no points to recommend except ordering drugs to buy outside of the hospital in case of hospital.

**Interviewer: --** I have finished my questions. Thank you.

**IDI-09**

**Interviewer: -** How early do women go to health facilities for ANC services when they recognize they are pregnant in your area?

**Interviewee: -** Women go to a health facility for ANC services at three months of their pregnancy.

**Interviewer: -**What types of services women do receive during ANC visits to the health facility?

**Interviewee: -**During the first time pregnancy testing was done to confirm the pregnancy. Then health professionals give counseling on what types of food need to eat during pregnancy time, not to do hard work, appointments for TT vaccine, and the next ANC visits will gate during her pregnancy.

**Interviewer: -** How often do pregnant women go to health facilities for ANC services in your area?

**Interviewee: -** Women go to health facilities three times for ANC services during pregnancy.

**Interviewer: -**What types of services do health professionals give to women during ANC follow-up?

**Interviewee: -**During ANC follow up skilled health professionals examine pregnant women and baby positions in the womb, do different laboratory tests, and give counseling to women on what needs to do during pregnancy.

**Interviewer: -** What are the factors that would motivate women to utilize ANC services during their pregnancy?

**Interviewee: -**As I think at the time of ANC visits skilled health professionals give appointments for women when to come back for the next visits and due to this reason women go to health facilities.

**Interviewer: -** What are the reasons that women do not go to health facilities for ANC services?

**Interviewee: -**As I think there are no other factors that do women not go to health facilities for ANC visits except women's weakness in decision-making due to lack of awareness about the importance of ANC follow-up and what kinds of problems may they face when she does not receive ANC services.

**Interviewer: -** Do you think that due to distance and access to services, women do not go to health facilities for ANC services?

**Interviewee: -** Distance is not factoring that women do not go to a health facility for ANC services, except for some women who live far from health facilities. Women who live in our area even can go by their foot due to the short distance.

**Interviewer: -** How about socio-cultural-related factors?

**Interviewee: -** As I think there are no socio-cultural related factors that do women not go to health facilities for ANC services in our area.

**Interviewer: -** Why do women go to the facility for the first ANC, but discontinue for subsequent ANC visits?

**Interviewee: -**There are no factors that do women discontinue ANC visits after they have received their first or second ANC services except a lack of willingness of women to go for ANC services.

**Interviewer: -** Can you tell us about the traditional practices and beliefs during pregnancy in your area?

**Interviewee: -**There are no traditional practices and beliefs during pregnancy in our area. It may be earlier but not now.

**Interviewer: -** What types of services community volunteers provided to pregnant women in your area?

**Interviewee: -**Community volunteers do mobilize pregnant women to go to health facilities for ANC services but no other things that they do for pregnant women and providing FAFA for malnourished women.

**Interviewer: -** Is there traditional birth attendant /TBAs/ in your area?

**Interviewee: -**There is no traditional birth attendant /TBAs/ in our area.

**Interviewer: -** How do you see health professionals and maternal health services provided to the community?

**Interviewee: -**As I think the services provided by skilled health professionals are good and attractive. They give quality care and keep the dignity of the women during the service-giving time.

**Interviewer: -**How do the communities see the maternal health programs and health professionals? Tell me your perception of maternal health care services and your perception of different care providers.

**Interviewee: -**As I think our community is satisfied by the services provided by health professionals in health facilities because there are a lot of improvements in care for pregnant women.

**Interviewer: -**In your opinion, what should be improved regarding ANC services?

**Interviewee: -** As I think the government needs to provide food for pregnant women because many are exposed to many problems due to not having enough food during their pregnancy. On the other hand health extension discharge pregnant women from the food-giving programs by saying women fulfill discharging criteria, but it is good if women stay in the program until delivery. After women are discharged from the program they will become malnourished because they do not have enough food from their home.

**Interviewer: -** What are the services that skilled health attendance gives to women during childbirth for themselves and their babies?

**Interviewee: -**We do not know what skilled health professionals do for women during the process of delivery because we keep them out of the delivery room until they finish delivering process. After delivery, they give medications for newborn babies, and women start immediately breasting feeding for their babies. On the other hand, skilled health attendants give glucose if it is needed.

**Interviewer: -**Where do you deliver your children last time?

**Interviewee: -**I have delivered all my children at home.

**Interviewer: -**What are the reasons that you deliver at home?

**Interviewee: -**Distance was the factor for me to deliver at home my last child because my labor was acute and fast and I have no enough time to go health facility. During that time I received ANC services at Yirgalem Hospital because there are no health centers in our area. But now, almost all women deliver at health facilities because there are health facilities in our area and health extension workers giving continuous maternal health education to women.

**Interviewer: -** If women deliver at home, what are the reasons and constraints that influenced women to utilize facility delivery services?

**Interviewee: -**As I think no reason that women deliver at home. All women need to deliver at a health facility because during home delivery women may face high bleeding, prolonged labor, retained placenta for a long period, and a piece of the placenta is left inside the mother. This can cause bleeding and other problems in women. Due to prolonged labor newborn babies may develop asphyxia and it also may die in the uterus.

**Interviewer: -** Can you tell us about the traditional practices and beliefs during childbirth?

**Interviewee: -**Covering or doing to wear many blankets during labor by believing that when a laboring mother is exposed to cold or air outside she cannot deliver quickly and this is one of the traditional practices during childbirth at home.

**Interviewer: -**What kinds of services do community volunteers provide to the women during delivery?

**Interviewee: -**Community volunteers do mobilize the community to go to health facilities for delivery services and they inform the health extension workers to dial for ambulance services.

**Interviewer:** - Are there traditional birth attendants/TBAs in your area?

**Interviewee:** - At the earlier time TBAs attend delivery at the time of home delivery, but now there are no traditional birth attendants/TBAs/ in our area.

**Interviewer: -**How do the communities see the maternal health programs and health professionals? Tell me your perception of maternal health care services and your perception of different care providers.

**Interviewee: -** **I think our community is satisfied with the services provided by health professionals in health facilities. Skilled health professionals give good care to women during delivery by showing a good face and giving compassionate services, and they assess the health of women and newborn children until discharge of women.**

**Interviewer: -**In your opinion, what should be improved regarding facility delivery services?

**Interviewee: -**At last time many problems should be improved regarding facility delivery services. There were problems related to the cleanness of the service-giving room and outside part of the room, at many times health professionals were not found in a health facility**.** At this time all these things are improved, water is also available to wash in the facility, and due to this, all women are satisfied with the services.

**Interviewer: -** How early do women go for PNC services after delivery?

**Interviewee: -** Women go to health facilities for PNC services at 45^th^ days after delivery.

**Interviewer: -**What types of services women do receive for themselves and their babies?

**Interviewee: -** During PNC visits women receive family planning services for themselves and immunization services for babies.

**Interviewer: -** How often do women go to PNC services?

**Interviewee: -** Women go to health facilities for PNC services three times.

**Interviewer: -** What are the factors that would motivate women to utilize PNC services in their childbirth in your area?

**Interviewee: -**As I think the factor that motivates women to utilize PNC services in their childbirth is the health-seeking behavior of the women for themselves and their children.

**Interviewer: -** If women don’t go for PNC, what are their reasons?

**Interviewee: -** As I think there are no factors that can do women do not go for PNC services except the laziness of the women.

**Interviewer: -** Are there any other factors related to distance and access that women do not go to health facilities for delivery services?

**Interviewee: -**As think distance is not a factor that does woman doesn't go to a health facility for PNC services because in case if her baby is sick she goes to a health facility without complaining about the distance.

**Interviewer: -** Are there any other factors related to financial problems do women do not go to health facilities for PNC services?

**Interviewee: -**As I think financial problems do not do woman doesn't go to health facility for PNC services.

**Interviewer: -**How about sociocultural factors?

**Interviewee: -** No socio-cultural related factors that do women not utilize PNC services.

**Interviewer: -** Can you tell us about the traditional practices and beliefs during the postpartum period?

**Interviewee: -**Giving clean (high land) water for newborn babies immediately during delivery at present and unclean water during an earlier time, giving AMESA to prevent FANCHO (rash on the skin of babies) and also earlier time applying butter on the cord after delivery.

**Interviewer: -** What kinds of services do community volunteers provide to the women after delivery?

**Interviewee: -**Community volunteers provide FAFA for malnourished women.

**Interviewer: -** What kinds of services do traditional birth attendants/TBAs give to women after delivery in your area?

**Interviewee: -**No services that traditional birth attendants provide for women after delivery.

**Interviewer: -** What kinds of services do health professionals provide for PNC services in your area?

**Interviewee: -**Health professionals give some foods to women after delivery for a short period.

**Interviewer: -**Do health professionals including health extension workers give care by respecting and showing a good face during service-giving time?

**Interviewee: -** Many health professionals do not serve by respecting and giving compassionate care to women.

**Interviewer: -**How do the communities see the maternal health programs and health professionals? Tell me your perception of maternal health care services and your perception of different care providers.

**Interviewee: -** As I think our communities does not satisfy by some services. After one malnourished woman starts to take food she should have to stay in the program until her child grows up but health professionals discharge the woman from the program in a short period.

**Interviewer:** -In your opinion, what should be improved regarding PNC services?

**Interviewee: -**Health professionals enforce women to use implants without the interests of women. Most time women want to use Depo-Provera due to its fewer side effects when compare to implants, but health professionals do not want to provide Depo-Provera. So, health professionals should have to provide family planning services according to the interests of women.

**Interviewer: -**Do women gate under five children treatments on time with necessary medications?

**Interviewee: -**Yes, health professionals appropriately give treat under-five children, and health extension workers are available at health during working times.

**Interviewer: -** Are there electricity services in the health facility in your area?

**Interviewee: -**In some health facilities there is no electricity so the government needs to solve these problems.

**Interviewer: -**How do you see the ambulance services that you receive in your area?

**Interviewee: -**There are no problems related to the services of the ambulance in our area except in the case of difficult roads for the ambulance.

**Interviewer:** - Do health extension workers give PNC services by going home to home in your area?

**Interviewee: -** Health extension workers do not give PNC services by going home to home.

**Interviewer: -** Do women can gate all drugs in the health posts of health centers?

**Interviewee: -**There are drug shortages in health posts and health centers in our area.

**Interviewee: -**Do husbands support women during their pregnancy and after delivery?

**Interviewer: -**Many husbands do not give support to women during their pregnancy and after delivery especially in rural areas. Women who live in rural areas do many works even when they are pregnant; preparing food and coffee for the whole family, preparing wood for fire, fetching water after a long journey, and…….. So, husbands should have to support women.

**Interviewer: -** I have finished my questions. Thank you.

**IDI-10**

**Interviewer:** How early do women go for ANC? Why do they go at that time? Why earlier or later? How often do they go to ANC? Why do they go at that time?

**Interviewee: -** I am the 29^th^ health promoter in the catchment. I serve up to 50 homes. Whenever I recognize that there is a pregnant mother in my area. I take her name and give her health education. I tell her to go to a health facility for ANC care as soon as she is 12 weeks pregnant.

Whenever we tell them they come. We have been educating the community for a long time about maternal health. Because of that pregnant mothers come and use the services. Whenever we see mothers who don’t use the services we advise them to seek care. Mostly they are motivated by their level of awareness.

**Interviewer:** If women don’t go for ANC, what are their reasons?

**Interviewee: -**yes, some mothers hold to traditional values and they reject our approaches. They say "We never used these things before, why should we now?" "We will be just fine. " But some of those rejecters have begun to believe after observing complications from not using modern services.

**Interviewer:** what about other barriers like financial and distance barriers?

**Interviewee: -** some say we don't have any money to pay. But we convince them all these services are free. We tell them of the benefits. We also see some who say the road is far but we tell them they will use ambulances during birth. These things aren't major barriers. Most of the attitudes are changing.

**Interviewer:** Why do women discontinue subsequent ANC visits?

**Interviewee: -** Sometimes they speak of their household and social life reasons and duties. Others say no major has been done during their care. When we see defaulters we convince them and motivate them again.

**Interview:** can you tell us about the traditional practice and beliefs during pregnancy?

**Interviewee: -** Not during ANC but FGM causes problems during delivery.

**Interviewer:-**How do you see the role of community volunteers/TBAs, and health extension workers on ANC care service delivery?

**Interviewee: -** I am the 29^th^ health promoter in the catchment. I serve up to 50 homes/mothers. We give health education to the HEW. Whenever I recognize that there is a pregnant mother in my area. I record and report to HEW. I urge and motivate her to use maternal services up to birth. That is what I do and the other promoters also do the same.

**Interviewer:** What should be done to further improve ANC care delivery?

**Interviewee: -** We the community organization need to be strengthened. Although we try our best, HDA is not going as strong as when we began. They are not giving us the proper support as before. They don't give us refresher training. If we receive proper support could be done there are things that we can ant to do. We want to do more.

**Interviewer:-**My next question is about facility delivery. Do mothers know the benefits of facility delivery in your area?

**Interviewee: -** The mothers know that professional care during delivery is very helpful to them. They say that they used to suffer complications but now thanks to the professionals they suffer no more. They know that facility delivery has helped greatly for their safety and the safety of their newborn.

The mothers are motivated by the quality of care they are receiving. They talk of how they received adequate care without much payment. When these mothers are treated well they call other mothers to use these services.

**Interviewer:-**For mothers who deliver at home, what are their reasons? Do women present a reason for the lack of transport or lack of money as a reason for home delivery?

**Interviewee: -** yes, there can be few mothers who deliver at home. When we ask them why mostly they reply that they had rapid labor and they couldn't reach a facility. They say it didn't give them time to call us. Others tell of the distance of the road from their home to the facility. it being far. But mostly lack of being prepared and lack o awareness. We try to educate everyone who delivered at home. If we see complications we bring them into the facility.

**Interview:** can you tell us about the traditional practice and beliefs during delivery?

**Interviewee: -** in the past female genital mutilation is an issue. But that practice has been outlawed and is being eliminated. We were also able to stop the habit of feeding traditional medicines called ‘Amessa' to children. We teach them about exclusive breastfeeding for six months. We tell them to not feed anything other than babies with underdeveloped digestive systems.

**Interviewer:-**How do you see the job being done by the community health development army specifically about facility delivery? What do you think should be done to improve facility delivery as well as the continuum of care?

**Interviewee: -** as I said earlier we need to renew our HDA army. If the government supports us there are a lot of things that could be accomplished. But the HDA needs to be strengthened and renewed

**Interviewer:-**What does PNC service look like in your facility?

**Interviewee: -** after the mothers are sent home we go to their homes. We see if there are any complications we teach them about hygiene and sanitation. We also teach them o breastfeeding as well as maternal nutrition.

Others like Mulu and Mastewal the health extension workers follow them up and visit them.

**Interviewer**: - Do mothers understand the benefit of PNC service to themselves and their newborns?

**Interviewee: -** most of the mothers understand how essential modern professional care is to them and their children, they are thankful for the care they receive,

**Interviewer: -** If mothers don’t use PNC care what do you think their reasons are?

**Interviewee: -** Those few mothers who deliver at home are the ones who miss out on PNC.

**Interviewer: -**How do you see the job being done by the community health development army and TBA specifically about PNC? What do you think should be done to improve PNC care as well as the continuum of care?

**Interviewee: -** as I said before after the mothers are sent home we go to their homes. We also teach them o breastfeeding as well as maternal nutrition. But I understand we need re-strengthening.

**Interviewer: -** I have finished my questions, thank you!

**Interviewee: -** Thank you!

**IDI-11**

**Interviewer:** How early do women go for ANC? Why do they go at that time? Why earlier or later? How often do they go to ANC? Why do they go at that time?

**Interviewee: -** Yes they come. After they come here they are tested for different aliments by the lab technician. Then they are vaccinated. After that, the mothers are told to follow their ANC care until the end and deliver in a health facility.

**Interviewer:** Why are the pregnant mothers late for ANC care?

**Interviewee: -** some mothers lack awareness and education. So they come late or they don’t

Come at all. Some women don't even know that they are pregnant. But the mothers who have better awareness come and take the health care provided here.

**Interviewer:** Among the women who attend the ANC care properly what do you think are the factors that motivate women to use ANC care?

**Interviewee: -** I use the service provided here. And all of the services provided here are provided for free. They also prepare different incentives for active participants in community discussions. The quality of care has been also improving in this facility. Essential drugs are also available in the facility. I think these factors motivate mothers to utilize the ANC care provided here.

**Interviewer:** If women don’t go for ANC, what are their reasons?

**Interviewee: -** Some of the mothers don't understand the benefits of ANC care. They say that they don't need these services and say that God will help them in their pregnancy and delivery. Some mothers who are pregnant for the first time fear needles and meet health professionals. They are shy and may not come to use ANC care.

**Interviewer:** what about other barriers like financial and distance barriers?

**Interviewee: -** No the services are given for free. And the roads are free. And I have not met any mothers who have quarreled with professionals and quit utilizing ANC care. These are not major issues in our area.

**Interviewer:** Why do women discontinue subsequent ANC visits?

**Interviewee: -** Because of forgetfulness and laziness. The mothers who have not fully committed and who lack complete awareness discontinue ANC care. But the health professionals do their at most to retain mothers who are enrolled in ANC care.

**Interview:** Can you tell us about the traditional practice and beliefs during pregnancy?

**Interviewee: -** No, not major practices that I know of.

**Interviewer:-**How do you see the role of community volunteers/TBAs, and health extension workers on ANC care service delivery?

**Interviewee: -** We meet every month with HEWs. And every one of us knows which woman is pregnant and reports their name to the health extension worker. We also counsel them to present themselves at health facilities and take professional care. This is the job we have been doing.

**Interviewer:** What should be done to further improve ANC care delivery?

**Interviewee:** We need to target the mothers who refuse to use the care. Strengthen the community mobilization effort at a community level. And further, improve the quality of ANC care at the facility.

**Interviewer:-**My next question is about facility delivery. Do mothers know the benefits of facility delivery in your area?

**Interviewee:** Most of them understand and some don't understand.

**Interviewer:-**For mothers who deliver at home, what are their reasons?

**Interviewee:** yes. There can be some mothers who deliver at home. Mostly it is due to a lack of being prepared and then their labor commences unexpectedly and they end up delivering at home. In other cases, some families are held into traditions and will be reluctant to facility delivery. Then they wait for the women to deliver at home. They only come to the facility if the mother develops complications.

**Interviewer: -** Do women present a reason for the lack of transport or lack of money as a reason for home delivery?

**Interviewee:** No there are not. When we ask the mothers why they delivered at home? They will say they already delivered peacefully and they dint need care.

**Interviewer:-**How do you see the job being done by the community health development army specifically about facility delivery?

**Interviewee:** We educate them about receiving care. How to take care of the babies and breastfeeding. Motivate the mothers and promote health services in the community.

**Interviewer:-**What do you think should be done to improve facility delivery as well as the continuum of care?

**Interviewee**: We need to target the mothers who refuse to use the care. Strengthen the community mobilization effort at a community level. And further, improve the quality of ANC care at the facility**.**

**Interviewer:-**What does PNC service look like in your facility?

**Interviewee**: yes**,** the take care of the PNC care within the twenty-four hours of delivery in the facility.

**Interviewer: -** Do mothers understand the benefit of PNC service to themselves and their newborns?

**Interviewee**: yes they know that vaccinations are important for babies. First-time mothers were also eager to receive health education about feeding and breastfeeding their children.

**Interviewer: -** If mothers don’t use PNC care what do you think their reasons are?

**Interviewee: -** Those few mothers who deliver at home are the ones who miss out on PNC.

**Interviewer: -**How do you see the job being done by the community health development army and TBA specifically about PNC? What do you think should be done to improve PNC care as well as the continuum of care?

**Interviewee: -** as I said before after the mothers are sent home we go to their homes. We also teach them o breastfeeding as well as maternal nutrition. But I understand we need re-strengthening.

**Interviewer: -** I have finished my questions, thank you!

**Interviewee: -** Thank you!

# IDI-12

**Interviewer: -** When did you give your last birth? When did you start ANC visits for your last pregnancy?

**Interviewee: -** I delivered my last child 15 months ago and I started ANC visits at three months of pregnancy during that time.

**Interviewer: -** How early do women go for ANC? Do mothers come early for ANC services when they recognize they are pregnant?

**Interviewee: -** Women should have to start ANC services at the time when she recognizes as she is pregnant and pregnancy becomes observable. I have followed ANC services twelve times at the health center and Yirba primary hospital. Health professionals told me to receive ANC services twelve times until childbirth. I have received ANC services starting from three months of my pregnancy, then at the fourth months, at sixth months, at seventh months,….in total, I have received ANC services three times at the health center and seven times at Yirba Primary Hospital.

**Interviewer:** - How do see the services that were provided by health professionals for you?

**Interviewee:** - Services that were provided by the health professionals during ANC follow-up and childbirth were good and more attractive. Health professionals gave respectful and compassionate care during my ANC follow-up; they do examinations of the positions of the babies in the uterus and different services until my childbirth. So, I was satisfied with the services that I have been got during my follow-up.

**Interviewer: -** What are the factors that would motivate women to utilize ANC services during their pregnancy?

**Interviewee: -** I delivered my child at home and during that time I faced the problem of not removing of placenta after childbirth due to reason I have decided to receive ANC services at a health facility and to deliver at a health facility. I have directly gone to a health facility for ANC services for my next child and delivered at Yirba health center and I have delivered my third child at Shololiyo health center. My third child was twins and it was identified by health professionals during my ANC follow-up.

**Interviewer: -**What are other services that you have got during that period?

**Interviewee: -** Health professionals also have given me counseling services for me how to take care of my pregnancy by not carrying heavy things and also not doing hard work.

**Interviewer: -** What are the reasons that women do not go to health facilities for ANC services?

**Interviewee: -** As I think the reason that women do not go to health facilities for ANC services is a lack of knowledge of women about the importance of services.

**Interviewer: -** Do you think that due to financial problems women do not go to health facilities for ANC services?

**Interviewee: -** As I think financial problems are not factors that do women not go to a health facility for ANC services because all maternal services that are provided in the health facility are free of cost (free services).

**Interviewer: -** How about distance and access to health facilities for ANC services?

**Interviewee: -** As I think distance is not a factor for not utilizing ANC services because people even go abroad for seeking better treatment after becoming ill. As I said before the main factor is woman’s lack of knowledge about the importance of services. Once up on time, I have been given counseling services for two women to go health facility for ANC services. After then health professionals provided ANC services and from them, one's pregnancy was outside of the uterus and she was referred to the hospital and she has got necessary services in the hospital.

**Interviewer: -**What are other factors that do women not go to health facilities for ANC services?

**Interviewee: -** There are no medical instruments that health extension workers used to give ANC services for pregnant women in our health post. So, health extension workers directly for pregnant women to go to health centers for ANC services, and in case of these some women do not go to health centers due to distance.

**Interviewer: -** How about socio-cultural-related factors?

**Interviewee: -** As I think there are no socio-cultural related factors that do women not go to health facilities for ANC services in our area.

**Interviewer: -** Why do women go to the facility for the first ANC, but discontinue for subsequent ANC visits?

**Interviewee:** - Some women discontinue subsequent ANC visits after they have received their first ANC services due to the distance of the health facility. When the gestational age increases it is difficult for pregnant women to go far distances on foot and other financial issues raised; for transport and might be for food.

**Interviewer: -** Can you tell us about the traditional practices and beliefs during pregnancy in your area?

**Interviewee: -** No traditional practices and beliefs that applied during pregnancy in our area. At an earlier time, some pregnant women do drink KOSO by thinking that it is useful for women and babies to clean the bodies of the babies in the uterus.

**Interviewer: -** Are there religious practices that affect mothers' use of care during pregnancy?

**Interviewee: -** No religious practices that affect mothers to use ANC services during pregnancy. Women do only praying to God to give health to them until they give childbirth and no other unnecessary religious-related practices that they do during pregnancy. But at the earlier time, many women do not want to go to a health facility for ANC services by believing that God can help them and do deliver at home without any problems and no need of going to a health facility except to pray to God. On the other hand, the mother-in-law does not permit pregnant women to go to a health facility for services by saying WOXA'YA (some sprits that helps women) do you give you birth at home without any difficulties and no need of going to a health facility. But now there are no like these kinds of problems in our area.

**Interviewer: -** What types of services community volunteers provided to pregnant women in your area?

**Interviewee: -** Community volunteers do mobilization of those pregnant women to go to health facilities for ANC service by continuously following those women whether they are going or not. There is one leader woman who led thirty women in the team or women development army and this leader woman does overall control whether pregnant women in the team utilize ANC services.

**Interviewer: -** How do you see health professionals and maternal health services provided to the community?

**Interviewee: -** Skilled health professionals give maternal health education to pregnant mothers about the problems or danger signs that might happen during pregnancy, at delivery time, and after delivery or post-natal period. On the other hand, they give different services to women during service-taking time. Skilled health professionals do overall examinations of pregnancy, give counseling services about the foods that she needs to eat during her pregnancy, not to do hard work, and also other related services. I am satisfied with the services provided by health professionals in the health facilities.

**Interviewer:** How do the communities see the maternal health programs and health professionals? Tell me the perception of maternal health care services and their perception of different care providers.

**Interviewee: -** Health professionals provide good ANC services in the health facility by respecting pregnant women and in a compassionate way. So our community is satisfied with the services provided by the health professionals in the health facility.

**Interviewer: -** In your opinion, what should be improved regarding ANC services?

**Interviewee: -** There are no medical instruments that health professionals can use during ANC service giving time in the health post. So, pregnant women are forced to go to a health center that is far from the health post, and due to distance and financial-related factors, many women do not go to health facilities for ANC services. So, the government should have to provide these necessary medical instruments for health posts. In the Shondoliwo health center sometimes health professionals do not available in the health center and they come after pregnant women wait for a long time and this should have to be improved.

**Interviewer: -** Where did you have been delivered your last child?

**Interviewee:** - I have been delivered my last child at Leku Hospital.

**Interviewer: -** Do you think that skilled health professionals had given good care for you and your newborns?

**Interviewee: -** During the time of my last childbirth skilled health professionals have given good care to me and my newborn babies; glucose during labor time, assisting me to push for second babies until I gave birth because it was twins, drugs by injection, and gave respect for me with compassionate care. They keep the dignity and confidentiality of the women during caregiving time.

**Interviewer: -** What are the factors that would motivate women to utilize delivery services during their pregnancy?

**Interviewee: -** I am motivated to utilize delivery services because I have got good maternal health education from health extension workers and skilled health professionals and I understand what kinds of problems women might be faced after they give birth at home. When women give a birth at health facility they gate good care for themselves and their newborn but when they deliver at home they use unclean material and put their newborn child in an unclean place which is exposed to bacteria and this can cause illness for a child.

**Interviewer: -** If women deliver at home, what are the reasons and constraints that influenced women to utilize facility delivery services?

**Interviewee:** - Some women deliver at home when their labor is acute and also if they were alone at home during labor and not having someone who takes them to a health facility.

**Interviewer:** -Do you think that women deliver at home due to financial barriers and opportunity costs and due to distance and access?

**Interviewee: -** Yes, some women deliver at home due to the distance of the health facility and lack of money; for example, one woman deliver on the road before reaching to health facility and we took this woman to the health facility by using local transport materials for placenta removal and other necessary services.

**Interviewer: -** Are there socio-cultural related factors that women deliver at home?

**Interviewee: -** As I think at the earlier time mother-in-law do women deliver at home by saying that God helps you during your delivery time and no need to go to a health facility for delivery services. But now our women have more awareness about maternal services and they utilize health facility delivery services and no socio-cultural related factors that women deliver at home at present time.

**Interviewer: -** Is there quality of care and non-dignified care-related factors that women not utilizing health facility deliver?

**Interviewee: -** As I think there is no quality of care-related factors because health professionals on giving well and quality care to women during delivery.

**Interviewer: -** Why do women go to the facility for ANC, yet mostly deliver at home?

**Interviewee: -** As I think some women deliver at home after they have received ANC follow-up at the health facility if labor is acute and deliver in a short period.

**Interviewer: -** Can you tell us about the traditional practices and beliefs during childbirth in your area?

**Interviewee: -** There are some community beliefs that children do not grow up and they will die if women deliver them out of the home and no other traditional practices and beliefs have been known.

**Interviewer: -** Do you think religious practices that affect mothers to use care during delivery?

**Interviewee: -** As I think there are no religious practices that affect mothers to use care during labor in our area.

**Interviewer: -** What kinds of services do community volunteers provide to the women during delivery?

**Interviewee: -** Community volunteers do mobilize pregnant women to utilize health facility delivery services and they do follow up on pregnant women during their pregnancy and take pregnant women to health facilities during labor. Some of them also even take women to health facilities by paying their money for transport services.

**Interviewer: -** What kinds of services that skilled health professionals provide to women during delivery?

**Interviewee: -**After women reach health facilities for delivery services skilled health professionals do overall examinations of laboring women and they do follow up actively. Firstly they checked labor whether it is true labor or not. After then, if it is true labor they do women wait in the health facility by taking bed until delivery and they do examinations in intervals.

**Interviewer: -** What kinds of services do you receive in childbirth? Are you satisfied?

**Interviewee: -** Skilled health professionals attend delivery services by giving good care to women and newborn babies. Health professionals do women take their newborn babies with a clean towel after giving cord care, administration of necessary medications, and giving vaccines, and our community is satisfied by the care that they received from skilled health professionals.

**Interviewer:** - As you have told me that you delivered your last child at the health facility 15 months ago what are the factors that motivate you to deliver in the health facility?

**Interviewee:** - I have delivered my second child at a health facility and I understand the advantages of delivering at a health facility. I am also decided to deliver at a health facility because I can gate good care for myself and my newborn babies. When I have to deliver at home may face different problems and even if the delivery place is not clean during home delivery and I decided to deliver at a health facility.

**Interviewer: -** Why do women deliver at home?

**Interviewee: -** Some women deliver at home due to the distance from health facilities and also some women fear opening their bodies during delivery in front of health professionals.

**Interviewer: -**Explain to us your experiences relating to the utilization of facility delivery care provided by skilled birth attendants; your interactions with skilled birth attendants during facility delivery and do they respect and compassion for attendants.

**Interviewee: -** Skilled health professionals do keep the confidentiality and the dignity of the delivering women and they give respectful and compassionate care during the entire process.

**Interviewer: -** How do the communities see the maternal health programs and health professionals? Tell me your perception of maternal health care services and your perception of different care providers.

**Interviewee: -** As I think except for the women who are lives far from the health facility and women who do not get support from their husbands and mother-in-law, our community awareness to use maternal health services are changed and they started to use all maternal health services and they are satisfied by the services that provided by the health professionals.

**Interviewer: -** In your opinion, what should be improved regarding facility delivery services?

**Interviewee: -** We have meetings at 15 days intervals and within these meetings health professionals give maternal health education and this should be continued until all women do deliver at a health facility. I have no points to raise regarding the quality of services because it is very good regarding health professionals and the services that they provide. In Shondoliwo health center sometimes health professionals do not available in the service-giving room at lunchtime and this should have to be improved.

**Interviewer: -** How early do women go for PNC services after delivery?

**Interviewee: -** I was gone to health facilities for PNC services at 45^th^ days after delivery.

**Interviewer: -**When did women need to go to a health facility for PNC services?

**Interviewee:** - For immunization services for their child women need to go at 45^th^ days of delivery and women can receive family planning services on that day or the day of delivery.

**Interviewer: -** When we say women go to health facilities earlier or later after delivery?

**Interviewee: -**We say later when women go to a health facility after two months of delivery.

**Interviewer: -** How often do women go to a health facility for PNC services?

**Interviewee: -** Women go to a health facility for PNC services on the 45^th^ day of delivery and after then they go according to appointments that health professionals give for them by writing on their appointment cards.

**Interviewer: -** What kinds of PNC services that health professionals provide for lactating women in your area?

**Interviewee: -** Health professionals provide family planning services for women according to their choices and immunization services for their babies until they receive their last vaccine doses at 15 months of a child during PNC visits.

**Interviewer: -**Do women think skilled attendance during postpartum help their babies and themselves?

**Interviewee: -** Yes, because they provide all family planning and immunization services without fees and within good approaches.

**Interviewer: -** What are the factors that would motivate women to utilize PNC services in their childbirth in your area?

**Interviewee: -** Women motivated to go to health facilities for PNC services; i.e. immunization services for their child that is necessary for the child to protect the child's illness and important for the child's growth, and also family planning services to control unwanted pregnancy.

**Interviewer: -** If women don’t go for PNC, what are their reasons?

**Interviewee: -** As I think almost all women go to health facilities for PNC services and if some women do not go to health facilities health extension workers inform community volunteers to mobilize them to go to health facilities for PNC services.

**Interviewer: -**Do you think that due to financial barriers and the distance of health facilities women do not go to health facilities for PNC services?

**Interviewee: -** No, because PNC services are provided in all health posts and health centers.

**Interviewer: -** Are there any other factors related to socio-culture that do women do not go to health facilities for PNC services?

**Interviewee: -** Some women think that their child will be ill and die when it is exposed to some evil eyes (BUDAKKO) and they decided to not take their child out to the house. On the other hand, some aged mothers-in-law do lactating women do not take their children to health facilities saying that children can grow up without immunization as earlier and no need for immunization.

**Interviewer: -** Are there any other factors in traditional practices that women do not go to health facilities for PNC services?

**Interviewee: -**Some women drink AMEESSA for their child by believing that it has protected FANCHO for the child.

**Interviewer:** -Are there religious practices that women do not go to a health facility for PNC services?

**Interviewee: -**In our area, there are no religious-related practices that women do not utilize PNC services in the health facility.

**Interviewer: -**How do you see community volunteers/TBA's services that they provide during the PNC period?

**Interviewee: -** Community volunteers do more work until one woman delivers her child at a health facility, and after delivery, the only women who go to a health facility for immunization services are those who don't go for immunization services.

**Interviewer: -** How do you see health professionals and maternal health services provided to the community during the PNC period?

**Interviewee: -** The services provided by the health professionals during the PNC period is very good and we are satisfied with their services.

**Interviewer: -** How do the communities see the maternal health programs and health professionals? Tell me your perception of maternal health care services and your perception of different care providers.

**Interviewee: -**Health professionals provide respectful and good care for women and I think our communities are satisfied by the services that are provided for women and their children. The awareness of our community regarding PNC services increased and almost all women utilized these services.

**Interviewer: -** In your opinion, what should be improved regarding PNC services?

**Interviewee: -** As I think health extension workers on providing immunization and family planning services in a very good way and they are available at working time. So we are satisfied with the PNC services and no other points that should be improved.

**Interviewer: -** I have finished my questions. Thank you.

**Interviewee: -** Thank you.

**IDI-13**

**Interviewer: -** How early do women go to health facilities for ANC services?

**Interviewee: -** As health development army leader (HDA) we identify pregnant women early in our area. Then we mobilize them to go to health facilities for ANC services and we linked them with health extension workers. Those pregnant women go to health facilities at four months of their pregnancy for ANC services. During their ANC follow up health professionals assess and examine the health status of pregnant women, giving education on what types of food are needed during the time of pregnancy.

**Interviewer: -** Are there women who come before or after 4 months of pregnancy for ANC services?

**Interviewee: -** No women come after 4 months of pregnancy. Even if she is not sure about the time of pregnancy we tell her to go to health facilities to get counseling and checking pregnancy.

**Interviewer: -** How often do you go to ANC services?

**Interviewee: -** Maybe I have missed frequencies and she goes to health facilities two to three times.

**Interviewer: -** Why does she go two or three times?

**Interviewee: -** To check the overall health status of her selves and pregnancy status and to get counseling services about any pregnancy-related problems and what to do at that time.

**Interviewer: -** Do women think skilled attendance during pregnancy is useful for their pregnancy?

**Interviewee: -** Yes, at the earlier time pregnant women do not accept ANC services and they think that God can keep our health and we do not need other services rather than praying to God for our health. But, after health extension workers gave continuous maternal health education the attitude and practice of our community changed. So, pregnant women think that skilled attendance during pregnancy is useful and all pregnant women in our area utilize ANC services.

**Interviewer: -** What are the factors that would motivate women to utilize ANC service in their pregnancy in your area?

**Interviewee: -** During earlier times women were delivering at home and they faced different problems; for example, due to excess bleeding and related factors they can lose their lives and their babies. But now these kinds of problems are well managed by health professionals and due to this reason they are motivated to go to health facilities for ANC services. In addition during ANC follow up they have gate good counseling services and quality care, getting bodybuilding foods FAFA, and also we mobilizing the community to go health facilities through health development army linkages.

**Interviewer: -** What are the reasons that women do not go to health facilities for ANC services?

**Interviewee: -** At this time, there are no factors that make women not go to health facilities for ANC services.

**Interviewer: -** Do you think that financial problems and opportunity of costs were barriers that women were not accessing ANC services?

**Interviewee: -** As I think all services for pregnant women are given for free and no need of paying for services. So, financial problem is not a factor in not utilizing the ANC services.

**Interviewer: -** What about distance and access to services?

**Interviewee: -** Due to distance and accesses no women were absent or dropped from ANC services utilization in our area. We have a functional and strong health development army and those who lead the network mobilizing women to go to health facilities for ANC services.

**Interviewer: -** How about socio-cultural-related factors?

**Interviewee: -** Women who become pregnant lately or somehow aged takes it as a shame and doesn't want to be exposed to others and go for ANC services. Women who become pregnant after their daughter has married are taken as unacceptable in society during an earlier time. But now the attitude of the community was changed and no like this kind of socio-cultural related factors.

**Interviewer: -** Are general delivery of services and quality of care can affect the utilization of ANC services in your area?

**Interviewee: -** Skilled health professionals are giving good and quality care for pregnant women and I don't hear service-related complaints.

**Interviewer: -** Why do women go to the facility for the first ANC, but discontinue for subsequent ANC visits?

**Interviewee: -** As I think those women who go to back without getting the services due to a lack of skilled health professionals may discontinue subsequent ANC visits.

**Interviewer: -** Can you tell us about the traditional practices and beliefs during pregnancy?

**Interviewee: -** During earlier times women were exposed to work-related problems; they prepare kocho from inset (Weese) by stretching their one leg up on MEETA, making flour by traditional ways, and carrying water after a long journey by foot. But now like these problems have improved and no traditional practices and beliefs during pregnancy.

**Interviewer: -** Are there any traditional birth attendants/TBA/ in your and how do you see them?

**Interviewee: -** During earlier times TBAs attends delivery and women faced different problems at that time. But now they are no TBAs in our area; for example, when I was delivering my second child my family bring TBAs to attend my delivery I reject her not to touch my body. After then, they have taken me to the health center and I delivered my second child this is the scenario for a long period.

**Interviewer: -** You are a community mobilizer and what kinds of service give you to those pregnant mothers?

**Interviewee: -** Our main work is community mobilization and we mobilize the women to go to health facilities for ANC services, not to deliver at home, and after deliver to PNC services.

**Interviewer: -**How do the communities see the maternal health programs and health professionals? Tell me your perception of maternal health care services and your perception of different care providers.

**Interviewee: -** Our communities take maternal health programs as good and it is necessary for women and children; they gate holistic and quality care, get FAFA and after delivery, they can gate plumpy nut if their child was malnourished. The services provided by health professionals are attractive and more acceptable and they have no skill gap.

**Interviewer: -** Your communities perception about different care providers; doctors, health officers, nurses, midwifes…

**Interviewee: -** As I think our communities cannot differentiate the types of professions of health professionals; doctors, midwives, … and only they need skilled health professionals.

**Interviewer: -** What efforts has your community made to increase maternal health service in your community?

**Interviewee: -** There is a collaboration of health extension workers with kebele administrative bodies to increase maternal health services, meeting is also held at a kebele level to create good awareness for women about maternal health and about the services given in health facilities.

**Interviewer: -** In your opinion, what should be improved regarding ANC services?

**Interviewee: -** As I think during ANC follow up women getting integrated services including FAFA programs and the services are good for women. If the government planned to give additional other services we agree on it, but we have no suggestion to give the improvement of maternal health.

**Interviewer: -** Do women think skilled attendance during childbirth helps themselves and their babies?

**Interviewee: -** Yes, when women deliver at home they faced different problems; i.e. excess bleeding problems, and not removing of placenta after childbirth. If the placenta does not remove after childbirth local birth attendants do women wait a long period by kneeling on their knees and pushing continuously until the removal of the placenta pumping QONCHO, higlands,… On the other hand, women discard first breast milk (colostrum). But now all these problems are prevented due to women deliver to health facilities by skilled health professionals.

**Interviewer: -**Why do women discard first breast produce milk (colostrum)?

**Interviewee: -** Women think that the first breast milk is not clean and it is not necessary for newborn babies. But now they understand that first breast produce milk is essential for a child.

**Interviewer: -**Why women were kneeling by their knees and they pushing something continually until their placenta was removed and what did they push?

**Interviewee:** - They pump bottle of soft drinks, QONCHO, and Highlands to get the power to remove the placenta and at that time no one give care for the newborns until the placenta is removed. But, at that time women losing her more energy and became tired.

**Interviewer: -**Is there a woman who died due to this traditional placenta removal process in your area?

**Interviewee: -** No, but many women were exposed to different problems like high bleeding, a child's loss of consciousness due to interruption of breathing, waiting a long period for removal of the placenta by kneeling at different styles and doing women to push until the placenta removing process finished.

**Interviewer: -**If women deliver at home, what are their reasons? Explain the constraints that influenced women to utilize facility delivery services.

**Interviewee: -** As I think some women deliver at home due to acute and fast labor and not having enough time to go to health facilities.

**Interviewer: -** Why do women go to the facility for ANC services, yet mostly deliver at home?

**Interviewee: -** No other known reasons to deliver at home after they have completed ANC services except those women who deliver at home due to acute and fast labor and not having enough time to go health facility.

**Interviewer: -** In your opinion, what should be improved regarding facility delivery services and to maintain the continuity of care?

**Interviewee: -** There is no electricity in health facilities and at night time health professionals use small hand batteries or lights. So, we need electricity in health facilities and also there is no water in the service giving a necessary room, especially in the delivery room. Lastly, it's good if more comfortable beds in the delivery room are accessed.

**Interviewer: -** How early do women go for PNC services? Probe why do they go at that time? Why earlier or later?

**Interviewee: -** Women go to health facilities for PNC service at 45^th^ days after delivery.

**Interviewer: -** Do women go earlier or later than 45^th^ days after delivery? Why earlier or later?

**Interviewee: -** Women do not go earlier or later than 45^th^ days after delivery.

**Interviewer: -** Do women think skilled attendance during postpartum help their babies and themselves?

**Interviewee: -** At present time awareness of women of PNC services is increased after they have gate more maternal health education and they know the advantages of PNC services for both mothers and their Children.

**Interviewer: -** What are the factors that would motivate women to utilize PNC services in their childbirth?

**Interviewee: -** During PNC services women can gate different services like FAFA, Vaccine for their child, and plumpy nut for malnourished children.

**Interviewer:** - Some women don’t go for PNC services and what are their reasons?

**Interviewee: -**In our area, no women who don't go for PNC and immunization programs for their child and they do not discontinue until their child completes the immunization services.

**Interviewer: -** Why do women go to the delivery at the facility, yet most don't receive PNC?

**Interviewee: -**No woman who doesn't go for PNC services after she has been delivered at a health facility in our area because they need to receive FP services and immunization for their child.

**Interviewer: -** In your opinion, what should be improved regarding PNC services and the continuum of care?

**Interviewee: -** In terms of health facilities PNC services are good and I have no points to give as a recommendation. But, if governments planned to add other maternal health services that are needed for women we accept it because no one knows what governments and God will do.

**Interviewer:** - In your opinion what should be done to women to go health facilities to get ANC to PNC without discontinuing until getting all services?

**Interviewee: -** At this time women getting all maternal services; ANC, Delivery, PNC, immunization services. During their follow up health professionals provide FAFA for mothers and plumpy nut for their child and regarding ambulance services, it is free of cost. So, we have no points to recommend but we accept that if government plans to provide other additional services.

**Interviewee: -** At last, if you have other additional points to say…

**Interviewee: -** There is a family planning commodities shortage in the health facilities especially short-acting family planning like Depo-Provera. Many women want to use Depo-Provera because its side effects for women are less when compare with other long-acting family planning like Implants. So, concerned bodies should have to provide Depo-Provera to fulfill women’s choice of FP services.

**Interviewer: -**I have finished my questions. Thank you.

**IDI-14**

**Interviewer:** how early do women go for ANC? Why do they go at that time? Why earlier or later? How often do they go to ANC? Why do they go at that time?

**Interviewee: -** yes they come and follow ANC care in our catchment we have 24 HDAs. Each of us HDA members knows every woman who is pregnant. We urge every pregnant woman to go to a health facility and take ANC care. When they come here the health care professionals give them necessary care starting from tabs for anemia and vaccinations for TT and other services.

**Interviewer**: what do you think are the factors that motivate women to use ANC care**?**

**Interviewee: -** because they know the harm that is caused by not taking care. the ones having awareness are motivated

**Interviewer:** Why are the pregnant mothers late for ANC care?

**Interviewee: -** sometimes the mothers say they wait because the baby inside them has not started moving. It’s cultural.

**Interviewer:** among the women who attend the ANC care properly what do you think are the factors that motivate women to use ANC care**?**

**Interviewee: -** it is a reception of the health education we and the HEWS have been giving, the

Mothers who have better awareness always go on to use the care.

**Interviewer:** If women don’t go for ANC, what are their reasons?

**Interviewee: -** they hold to traditional values. They say our god will take care of us and they don’t come.

**Interviewer:** what about other barriers like financial and distance barriers?

**Interviewee: -** yes some people in our catchment are very poor and may not come because of transportation and lack of roads. But I have not faced anyone the ones who didn't use it because of quality of care reasons.

**Interviewer:** why do women discontinue subsequent ANC visits?

**Interviewee: -** it’s because of a lack of awareness and holding into traditional values.

**Interview:** can you tell us about the traditional practice and beliefs during pregnancy?

**Interviewee: -** in the past there were but currently the community has been educated about them. The woman is wise and these habits have been eliminated greatly.

**Interviewer:-**How do you see the role of community volunteers/TBAs, and health extension workers on ANC care service delivery?

**Interviewee: -** yes as I have told you before we meet every month with HEWs. And every one of us knows which woman is pregnant and e reports their name to the health extension worker. We also counsel them to present themselves at health facilities and take professional care. This is the job we have been doing.

**Interviewer:-**My next question is about facility delivery. Do mothers know the benefits of facility delivery in your area?

**Interviewee: -** in the past traditional birth attendants used to deliver the mothers. But currently, those people have been retrained and are engaged as health promoters. So when mothers labor they are sent directly to health facilities. We have a phone number of the ambulances and we call the ambulances directly when it is night hours. In the day hours health extension workers call the ambulances. That is why facility delivery has improved in our area.

**Interviewer**: what do you think are the factors that motivate women to use ANC care**?**

**Interviewee: -** They get better care. They get enough medication. And they suffer fewer complications. That is why mothers prefer giving birth in the facility to giving birth at home.

**Interviewer:-**for mothers who deliver at home, what are their reasons?

**Interviewee: - Mostly, mothers will give birth at home due to fast labor**. Or some mothers who hold on to traditional values and who refuse modern care.

**Interviewer:-** Do women present a reason for the lack of transport or lack of money as a reason for home delivery?

**Interviewee: -** these days fewer and fewer mothers deliver at home. Sometimes roads could be bad during winter but we do have ambulances. In terms of money mostly the services are free and all these factors are not a significant hindrance.

**Interview:** can you tell us about the traditional practice and beliefs during pregnancy?

**Interviewee: -** In the past, the used to feed this traditional herb called "Hamessa’ to the newborn but that habit has been tackled and is being omitted. Among mothers who deliver at home the navel of the babies used to get wounded. As long as the home deliveries are getting fewer these habits are becoming absolute.

**Interviewer:-**How do you see the job being done by the community health development army specifically about facility delivery?

**Interviewee: -** we greatly discourage home births. When we find home deliveries we report to the facility. We give them health education about the benefits of facility delivery. We educate the mothers on how serious complications can be. We also run monthly health education campaigns with health extension workers.

**Interviewer:-**What do you think should be done to improve facility delivery as well as the continuum of care?

**Interviewee: -** continuing the health education and strengthening the service. We can improve the quality of the services.

**Interviewer:-**What does PNC service look like in your facility?

**Interviewee: -** We go and visit the mothers when they are sent home after delivering at the facility. I give education on my part that the mother should breastfeed the baby up to ten times daily, and that the mother should exclusively breastfeed the baby for six months. I also give her education about complications. That is the pattern we do our job.

**Interviewer**: - Do mothers understand the benefit of PNC service to themselves and their newborns?

**Interviewee: -** yes they understand when the time for vaccination arrives they come here happily and vaccinate the baby.

**Interviewer: -** if mothers don’t use PNC care what do you think their reasons are?

**Interviewee: -** not having adequate awareness. if mothers are aware they always come. I don't think money and access are issues for not using. Maybe the roads are not comfortable during winter. Some families may not support these that are rarely seen.

**Interviewer:** - What do you think should be done to improve PNC care as well as the continuum of care?

**Interviewee: -** we have the basic working system in place. That we need to strengthen our activities and the government should support our efforts. They should support us. In terms of quality of care, there can be improvement in supplying all the necessary supplies. Sometimes essential medicines are missing. We also have problems with water and electricity I think these issues can be improved I think we will improve the health care given to mothers.

**Interviewer: -** I have finished my questions, thank you.

**Interviewee: -** I thank you too!

**IDI-15**

**Interviewer: -** How early do women go to health facilities for ANC services when they recognize they are pregnant in your area?

**Interviewee: -**Women go to a health facility for ANC services at six months of pregnancy to take the TT vaccine. Women receive ANC services three times until delivery and at the ninth month of pregnancy, they take iron folate to prevent anemia.

**Interviewer: -** Why do earlier or later women go to health facilities for ANC services?

Interviewee: -Women are exposed to different problems like bleeding during pregnancy due to not taking the TT vaccine at six months of pregnancy. So, pregnant women should go to a health facility at six months of their pregnancy.

**Interviewer: -** How often do pregnant women go to health facilities for ANC services in your area?

**Interviewee: -**Women go to health facilities three times for ANC services during pregnancy and at nine months of Iron folate to prevent anemia.

**Interviewer: -** Do women think skilled attendance during pregnancy helps their pregnancy in your area?

**Interviewee: -**Yes, in our area women know about the importance of ANC services and they think that skilled attendance during pregnancy helps them.

**Interviewer: -** What are the factors that would motivate women to utilize ANC services during their pregnancy?

**Interviewee: -**Health extension workers give good health education on maternal services for women, and also women gate different services during ANC follow up including the tests and treatment for syphilis. So, due to these reasons, women are motivated to utilize ANC services.

**Interviewer: -** What are the reasons that women do not go to health facilities for ANC services?

**Interviewee: -**Women who do not have the experience to go to health facilities for ANC services last time do not want to go for ANC services. Women who deliver at home before think that God can help them as before and no need to go for ANC services.

**Interviewer: -** Do you think that due to financial problems, distance and access to services women do not go to health facilities for ANC services?

**Interviewee: -** No distance and access-related factors that women do not go to health facilities for ANC services because health facilities do not far from our homes. But some women do not go to health facilities those who have no money to pay for a card.

**Interviewer: -** How about socio-cultural-related factors?

**Interviewee: -**Yes, some women do not go to health facilities when it does not support by their mother-in-law. Some mothers-in-law do women do not go for ANC services by saying I have delivered all my children at home and nothing happened during that time for me and my children and no need to go for ANC services.

**Interviewer: -** Are general delivery of services and quality of care can affect the utilization of ANC services in your area?

**Interviewee: -**No, because they gate quality services in the health facility.

**Interviewer: -** Why do women go to the facility for the first ANC, but discontinue for subsequent ANC visits?

**Interviewee: -**As I think some women may discontinue subsequent ANC visits when they think it doesn't help them and only God can help them due to a lack of basic knowledge.

**Interviewer: -**Are there other factors related to financial problems, distance and access, and socio-cultural factors that do women discontinue subsequent ANC visits?

**Interviewee:** - As I think there are no other factors.

**Interviewer: -** Can you tell us about the traditional practices and beliefs during pregnancy?

**Interviewee: -**No traditional practices and beliefs during pregnancy in our area.

**Interviewer: -** Are there religious practices and cultural norms that affect mothers' use of care during pregnancy?

**Interviewee: -** Some women believe that God can help me and I don't have to go to health facilities for ANC services and they said that God speaks to me that no need to go to health facilities and everything related to my pregnancy becomes well.

**Interviewer:** - Is there traditional birth attendant /TBAs/ in your area?

**Interviewee: -** At this time there is no traditional birth attendant /TBAs/ in our area. At an earlier time, there were one TBAs in our area but she died, and no other TBAs.

**Interviewer: -** How do you see community volunteer services provided to the community?

**Interviewee: -** No I came to town at the recent time and no community volunteers I have seen in this area.

**Interviewer:** -How do you see health professionals and maternal health services provided to the community?

**Interviewee: -**As I have seen before health professionals give good maternal health services for pregnant women.

**Interviewer: -**What kinds of services do health professionals provide for pregnant women?

**Interviewee: -**During ANC follow up health professionals provide TT vaccines three times and those women do not see unnecessary bleeding during pregnancy at the ninth month they provide iron folate to prevent pregnancy-related anemia.

**Interviewer: -** In your opinion, what should be improved regarding ANC services?

**Interviewee: -**In my opinion, it is good if women go to health facilities for ANC visits because it is important for the women. On the other hand, the government needs to open health facilities at their near site for the women who live far from the health facilities, and also governments need to plant health information giving boards and pictures at near sites.

**Interviewer: -** What efforts has your community made to increase maternal health service in your community?

**Interviewee: -** Community needs to mobilize the women to go to health facilities for ANC services.

**Interviewer: -** Do women think skilled attendance during childbirth helps themselves and their babies?

**Interviewee: -**Women don't think that skilled attendance during childbirth helps themselves and their babies. There is nothing that motivates women to deliver at a health facility; like information-giving broachers and boards or billboards.

**Interviewer: -**What are the factors that would motivate women to utilize delivery services during their pregnancy?

**Interviewee: -**There are no motivating factors that would motivate women to utilize delivery services in the health facility regarding government except supports that women get from their families to go for delivery services. Women who have ANC visits are motivated to go for delivery services because, during their ANC follow-up, the gate counseling services do not deliver at home but rather go to a health facility immediately at the time of labor.

**Interviewer: -**If women deliver at home, what are the reasons and constraints that influenced women to utilize facility delivery services?

**Interviewee: -**There are no governing bodies that motivate women to go deliver services and due to reason they deliver at home. When someone asks women why they deliver at home they said that God helps them to deliver without any problems. So I don't want to go health facility.

**Interviewer: -**Are there any other factors related to financial problems and distance and access that women do not go to health facilities for delivery services?

**Interviewee: -**Yes, some women delivery at home due to lack of money to buy towels used for babies and some needed clothes. On the other hand, some women deliver at home if labor is acute and health facilities so far from their homes.

**Interviewer: -**How about socio-cultural-related factors?

**Interviewee: -** There are no socio-cultural related factors that women deliver at home in our area that I have heard.

**Interviewer:** - Is there any quality of care and non-dignified care-related factors that women deliver at home?

**Interviewee: -** No, health professionals give good care to women.

**Interviewer: -** Why do women go to the facility for ANC, yet mostly deliver at home?

**Interviewee: -** I don’t know why women deliver at home after they have utilized ANC services.

**Interviewer: -** What are the traditional practices and beliefs during childbirth in your area?

**Interviewee: -** Women wait long periods in labor until delivery, TBAs also put their hands on without wearing protective materials, and also do to wait long periods for placenta removal after delivery of their babies. On the other hand, TBAs use blades to cut a cord that may not be clean and cause other problems for newborn babies.

**Interviewer: -** How do you see health professionals and maternal health services provided to the community?

**Interviewee: -** Health professionals give good delivery services and care for mothers and if the case is out of their capacity they refer to other higher service-giving health institutions.

**Interviewer: -** In your opinion, what should be improved regarding facility delivery services?

**Interviewee: -** In my opinion, it is good if maternal health education posters were posted and delivered giving rooms decorated and attractive for women.

**Interviewer: -** How early do women go for PNC services after delivery?

**Interviewee: -** As I think women go to health facilities for PNC services at 45th days after delivery and during that women gate care and family planning services for themselves and immunization services for babies.

**Interviewer: -** Are there women who go to a health facility before the 45^th^ day of delivery?

**Interviewee: -** No, women will not go to health facilities before the 45^th^ day after childbirth unless illness happens for them and their children in our area.

**Interviewer: -** Are there healthcare workers who come to mothers' homes to give PNC services at their homes?

**Interviewee: -**No one comes to the mother's home to give PNC services at their home.

**Interviewer: -** How often do women go to PNC services?

**Interviewee: -** Women go to a health facility until their child completes immunization.

**Interviewer: -**Do women think skilled attendance during postpartum help their babies and themselves?

**Interviewee: -**Yes, because immunization helps a child to increase the immunity of the child to prevent diseases and control unwanted pregnancy for women during the postpartum period.

**Interviewer: -** What are the factors that would motivate women to utilize PNC services in their childbirth in your area?

**Interviewee: -**As I think there are no motivating factors, but women only decide to go to health facilities for PNC services. On the other hand, women go to health facilities if any illness in their child and they feel diseases themselves.

**Interviewer: -** If women don’t go for PNC, what are their reasons?

**Interviewee: -**Some women do not go to health facilities due to the lack of knowledge about the importance of PNC services.

**Interviewer: -** Are there socio-cultural cultural related factors?

**Interviewee: -** At most times in our community, it is not acceptable for women go to outside of their homes before the 45^th^ day of delivery.

**Interviewer: -**Are there any other factors that are related to financial problems do women not go to a health facility for delivery services?

**Interviewee: -**Yes, some women do not go to health facilities due to lack of money to buy towels which are used for babies, and some needed clothes for their children themselves.

**Interviewer: -**Why do women go to the delivery at the facility, yet most don't receive PNC?

**Interviewee: -**I don't know why they do not go to the health facility after they have delivered at a health facility.

**Interviewer: -** Explain the obstacles that influenced women to utilize skilled care during postpartum in your community. Do financial barriers and opportunity costs and distances?

**Interviewee: -** As I think some women do not go to health facilities when they it is difficult to without transport accesses and due to distances from the health facility.

**Interviewer: -**Are there women who do not go to the health facility by thinking of not gating quality care in the health facility?

**Interviewee: -**I do not know women who do not go to health facilities due to complaining about the quality of the care.

**Interviewer: -** Can you tell us about the traditional practices and beliefs during the postpartum period?

**Interviewee: -**No such many traditional practices and beliefs during the postpartum period, but some women give AMESA for their children by thinking that it prevents diseases called FANCHO (skin diseases with a rash on the skin) and helps for the growth of the child. Women give AMESA to a child until they start to stand up.

**Interviewer: -**Do you think that AMESA is useful for newborn babies?

**Interviewee: -**Yes, AMESA is useful for children. It cleans the internal bodies of a child, helps with growth, and increases the weight of the babies.

**Interviewer:** - Do you think that health professionals do women to go health facilities during the postpartum period?

**Interviewee: -**Yes, health professionals give health education for women to go to health facilities for the postpartum period.

**Interviewer: -**In your opinion, what should be improved regarding PNC services to all women who deliver either home or health facility?

**Interviewee: -** I have no other points to recommend, but the price of goods is increased and government needs to control and do to reduce the prices of it that are needed for women and their child.

**Interviewer: -** I have finished my questions. Thank you!

**IDI-16**

**Interviewer: -**  Firstly I would like to thank you for your voluntariness for interviews I directly go to interviews. How early do women go to health facilities for ANC services in your area?

**Interviewee: -** Women go to health facilities at three months of pregnancy when they feel the first sign and symptoms of pregnancy to confirm their pregnancy and to receive ANC services. After then they are registered and take appointment cards for the next follow-up until nine months of pregnancy.

**Interviewer: -**What kinds of services pregnant women would gate during ANC follow-up?

**Interviewee: -** During ANC follow up women gate different services; TT vaccine, Iron folate prevents pregnancy-related anemia and also it is useful for the mental growth of babies, examining the health status of both mother and pregnancy.

**Interviewer: -** Are there women who come before or after 3 months of pregnancy for ANC services?

**Interviewee: -** Women go to the health facility when they feel the first sign and symptoms of pregnancy they directly go to health facilities at three months of pregnancy and no women come after 3 months of pregnancy.

**Interviewer: -** Do women think skilled attendance during pregnancy helps their pregnancy?

**Interviewee: -** Yes, because during ANC follow up women gate different and useful services for themselves and their pregnancy. In health facilities health professionals give quality and attractive care including counseling services. So, pregnant women think that skilled attendance during pregnancy is useful and all pregnant women in our area utilize ANC services.

**Interviewer: -** What are the factors that would motivate women to utilize ANC service in their pregnancy in your area?

**Interviewee: -** Firstly community mobilizer identifies pregnant women in the kebele and gives more maternal education by using pregnant women forums to go to health facilities for ANC services. They also mobilize the women to use immunization services for their children by collaborating with health extension workers. Women also receive syphilis tests and how to manage any unwanted signs and symptoms related to pregnancy and also counseling services for false labor.

**Interviewer: -** What are the reasons that women do not go to health facilities for ANC services?

**Interviewee: -** AS I think some women do not go to health facilities due to the workload at their home. When their children go to school they are left to keep the cattle and work in the home, and shortage of time to go to health facilities.

**Interviewer: -** Do you think that financial problems and opportunity of costs were barriers that women were not accessing ANC services?

**Interviewee: -** AS I think due to the workload in their home some women go to health facilities at the time of lunch and at that time and they wait for the health professionals until they returned to work from lunch. During that time women who go hungry need to eat lunch and those who have money may think about this problem them missing the ANC services.

**Interviewer: -** What about distance and access to services?

**Interviewee: -**As I think those women who are far from health facilities need transport access; like motorcycles to go and it accessed by fees and those who have no money missed ANC services.

**Interviewer: -** How about socio-cultural-related factors?

**Interviewee: -**As I think some women don't go to health facilities for ANC services when it is doesn't support by their mother-in-law.

**Interviewer: -** Are general delivery of services and quality of care can affect the utilization of ANC services in your area?

**Interviewee: -** No service quality-related factors that do women do not use ANC services because the services in the health facilities are good, even if some drugs missed in the facility they gate appointment and come back but not missed it.

**Interviewer: -** Why do women go to the facility for the first ANC, but discontinue for subsequent ANC visits?

**Interviewee: -**As I think some women discontinue subsequent ANC visits due to the workload in their home, their children going to school, death in the family and neighbors, and financial and distance-related factors.

**Interviewer: -** What kinds of traditional practices and beliefs during pregnancy are in your area?

**Interviewee: -**As I think no traditional practices and beliefs during pregnancy except delivery time. During a time of home delivery traditional birth attendants women wait for longs periods of labor which may lead to the death of the woman and child, they insert their bare hands to remove the placenta, they cannot manage to bleed, and if there is a rupture in women's organs, and also short size cutting of the cord and may this practice leads to death of a child due to bleeding.

**Interviewer: -** Ok, is there traditional practice and beliefs after women deliver their child?

**Interviewee:** - No traditional practice on women and children after delivery. In the earlier time, women use AMESA prepared by home taking grasses that are called AMESA grass for their newborn children but not at present time.

**Interviewer: -**When should you first bathe your newborn during home delivery?

**Interviewee: -**When women deliver at home they take first bathe newborns immediately after delivery and this child is exposed to cold and also develops pneumonia. But, women who deliver at health facilities start to take a first bath after 24 hours of delivery according to health education from health professionals.

**Interviewer: -**Is there other traditional practices after delivery?

**Interviewee: -**Yes, women who deliver at home are recommended to take a shower after three or four days of delivery and this is not good for the health of women.

**Interviewer: -**Are there any other un-raised traditional practices after delivery?

**Interviewee: -**Yes, after delivery women used unclean towels or clothes for newborn babies and this may cause other infections for the child.

**Interviewer: -** In your opinion, what should be improved regarding ANC services?

**Interviewee: -**Building health centers and health posts near the place to women's gate all maternal service without going far distance, fulfilling necessary medical machines in health facilities that are used to examine the positions of the babies in the womb. Some women faced problems with the mal-presentation of their babies during delivery even though they have received ANC services why are they not examining by using machines due to lack of machines? So government should have to fulfill these essential machines in the health facility. I also wanted to recommend that governments work on the availability of insecticide-treated bed nets (ITN), soaps for personal hygiene, and clean towels at health facilities for women after delivery.

**Interviewer: -** What other things governments should be improved regarding ANC services?

**Interviewee: -**It is good if water and a shower-taking room are available for women after delivery in the health facility and heater machines water.

**Interviewer: -** Do women think skilled attendance during childbirth helps themselves and their babies?

**Interviewee: -**Yes, because women receive different services when they deliver at health facilities. But, women who become pregnant after woman's age increases do not think skilled attendance during childbirth is useful for them.

**Interviewer: -** What factors would motivate women to utilize delivery services during their pregnancy?

**Interviewee: -**In our area, pregnant women know the advantages of delivering at health facilities for their health and newborns. On the other hand, the services that they gate quality care in the health facilities.

**Interviewer: -** If women deliver at home, what are their reasons?

**Interviewee: -**Women deliver at home due to acute and fast labor because they do not have enough time to go to health facilities. Some women choose to deliver at home because they do not want to open their bodies to others like health professionals by thinking of it as shameful and it decreases the dignity of women.

**Interviewer: -** At which age group do women fear to deliver at a health facility?

**Interviewee: -**This is not common for younger women, but it is common in older women.

**Interviewer: -**Why do older women fear delivering at a health facility? Is the reason related to the non-dignified care of health professionals?

**Interviewee: -** Older women think that it is not good and unacceptable to open their bodies to health professionals who are younger than them and prefer home delivery. Some women deliver up to six to eight children and those women do not accept delivering at health facilities.

**Interviewer: -**Do you think that distance and access is the factor for women to deliver at home?

**Interviewee: -**AS I think some women deliver at home due to distance and access to transport and financial problems with buying necessary materials**.** Women who do not have money to buy towels, and other necessary clothes do not want to go to health facilities for deliver services.

**Interviewer: -**Are there socio-cultural related factors to delivering at home for younger women?

**Interviewee: -**No, when they experience signs of labor they directly go to a health facility.

**Interviewer: -**I have finished my questions. Thank you.

**KII-01**

**Interviewer:** how early do women go for ANC?

**Interviewee: -** yes, mothers come for ANC care before sixty weeks. Some mothers do come early but also some mothers come late. The mothers who come early are the ones who have previously used our services. New mothers and those lacking awareness come late for ANC care.

**Interviewer:** Why are the pregnant mothers late for ANC care?

**Interviewee: -** As most mothers who use our services are from rural areas awareness differs among users of our services. Lack of awareness is the major factor in being late for ANC care.

As you know ours is one of distant health facility and distance plays a role. We have two catchments these are Boro Shawala and Gasara Kuwe. There is also a problem with roads, especially in summer times pregnant mothers struggle to come to the facility. The road problem persists in the Gasara-Kuwe catchment.

**Interviewer:** Among the women who attend the ANC care properly what do you think are the factors that motivate women to use ANC care**?**

**Interviewee: -** We are teaching the community and the mothers through our monthly maternal forums on the benefits of ANC care. The mothers have also seen how traditional methods hurt women through complications. On the other hand, there is also work done to make MCH services accessible and affordable. These factors are helping to motivate mothers into receiving professional care.

**Interviewer:** If women don’t go for ANC, what are their reasons?

**Interviewee: -** Some mothers in our community lack awareness. They say our "ancestors never used modern services and they lived just fine." Other women are timid and decline to participate in our community forums. These people need a lot of pressing to engage. Mostly it is due to a lack of adequate awareness that stops them from using professional care**.**

**Interviewer:** what about other barriers like financial and distance barriers?

**Interviewee: -** As I explained earlier you know our facility is a distant health facility. Lack of money makes mothers struggle with transportation costs. So distance and access issues may affect our MCH service delivery.

**Interviewer:** why do women discontinue subsequent ANC visits?

**Interviewee: -** Mostly it is due to a lack of adequate awareness that stops them from using professional care. So, distance and access issues may affect our MCH service delivery.

**Interviewer:-**How do you see the role of community volunteers/TBAs, and health extension workers on ANC care service delivery?

**Interviewee: -** yes, they do work to educate the community about the benefits of health care and the risks of not using

**Interviewer:-**My next question is about facility delivery. Do mothers know the benefits of facility delivery in your area?

**Interviewee: -** In the past, they were embarrassed by giving birth at the hands of professionals but when they saw the value of our care and the complications from home delivery they know have changed their feelings about professional care. The inspiring factor is primarily the improved awareness of the mothers on the benefits of facility delivery. This was achieved by the uninterrupted health education activities we are carrying out in partnership with the community health organizations like HDAs, health extension workers, and professionals from our facility.

**Interviewer:-**For mothers who deliver at home, what are their reasons?

**Interviewee: -** as previously said for those mothers who hold to past views, mothers are more likely to deliver at home. They don’t know their expected delivery period and are less prepared and end up delivering at home. Some husbands and families may not be cooperative and may discourage facility delivery

**Interviewer**: - Do women present a reason for the lack of transport or lack of money as a reason for home delivery?

**Interviewee: -** ours is one of the remote health facilities and distance plays a role. The poverty level in parts of our catchment is high and mothers struggle with transportation costs. Distance and access issues defiantly play a role in home deliveries here.

**Interviewer:-**How do you see the job being done by the community health development army specifically about facility delivery?

**Interviewee:** The community volunteers and the traditional birth attendants have been organized into a community organization called the health development army/HDA. These organizations help us a lot in our service delivery. They bring a laboring mother to our facility. And also thy call ambulances when there is a need for the ambulances. They also report to us whenever home delivery occurs. I think they are helping the service delivery in our area.

**Interviewer:-**What does PNC service look like in your facility?

**Interviewee: -** We make the mothers stay for 24 hours in our facility. We follow them for complications. We also observe their breastfeeding and advise them. We also counsel the mothers on post-natal FP and provide the service as necessary**.** We also manage complications if and when they arise.

**Interviewer**: - Do mothers understand the benefit of PNC service to themselves and their newborns?

**Interviewee: -** Yes, they come and follow when we give them an appointment.

**Interviewer: -** if mothers don’t use PNC care what do you think their reasons are?

**Interviewee: -**Home deliveries and lack of awareness are the major hurdles for PNC care...

**Interviewer: -**How do you see the job being done by the community health development army and TBAs specifically about PNC?

**Interviewee: -** as I have told you before we educate the mothers on every maternal topic and they helped greatly in achieving this.

**Interviewer: -** I have finished my questions, thank you

**Interviewee: -** Thank you!

# KII 02

**Interviewer: -** What does the ANC service utilization look like in your area?

**Interviewee: -** In earlier times ANC services were given in 4 visits but now it has been increased to 8 visits. In our area, there is a culture where mothers aren’t considered properly pregnant before 4 months. They want to hide their pregnancy by considering it blood or water in their uterus and holding it in secret. But now they have begun to acknowledge their pregnancy early. ….this is partly attributable to our programs for pregnant mothers in our area and our mother's forums. These programs are called MAMA/FAFA programs and they enroll women and children for food aid. As we all know our area is affected by drought, conflicts, and security problems and a lot of people need food assistance. I believe these programs are helping mothers to receive ANC services as early as possible. When this program is undertaken all pregnant women are called to the facility by community mobilizes. During this time these women will begin their ANC care services

**Interviewer: -** Can you elaborate a little further on MAMA/FAFA programs you mentioned earlier?

**Interviewee: -**These MAMA/FAFA programs are nutritional support programs for pregnant mothers and children. Mothers and children are enrolled in these programs by screening their MUAC, and other nutritional deficiency indicators.

**Interviewer: -** What do you think are the barriers to attending ANC services in your area?

**Interviewee:** -Do you mean the challenges to women who come to use ANC services?

**Interviewer:** -No, I am talking about pregnant mothers who don't use ANC services. What do you think are the barriers that prevent women from Using ANC services?

**Interviewee: -** In my opinion awareness issues and issues related to cultural traditions are barriers that prevent mothers from using ANC services. Some women say our mothers never used any modern services but nothing happened to them. So that nothing will happen to us if we didn't use these services. As I have told you before there is a culture in our area that pregnant women want to hide their pregnancy until their abdomen becomes apparent fearing evil eye and other issues. These practices are hindering ANC services in our Area.

**Interviewer:-**Any barriers associated with the general delivery of services and their quality

Interviewee: - Yes, I think issues associated with the quality of services still hinder ANC service utilization. For example, there is a problem with electric power cuts in our institutions. Pregnant mothers who were sent for lab tests will have to wait for long hours and some of the mothers even may not return for the next visit because of these problems. ……… in other times for some visits done in health posts by health extension workers the health posts can be closed or the health extension worker may not be there….I think there needs to be better inspection and control of health extension workers regarding ANC service delivery……We also have a staff shortage for quality service delivery. …I raise all these as barriers to ANC service.

**Interviewer: -**What about financial barriers?

**Interviewee: -**No, I don’t think money problems affect ANC service provision. All of our ANC services are given free of charge.

**Interviewer: -** My next question is about mothers who discontinue ANC services. After having the first or/and second visit?

**Interviewee: -**Issues with ANC service discontinuation are in my opinion associated with lacking full awareness. For some mothers issues related to the distance of the facility from their home could also cause them to discontinue.. for example, mothers from 'Kitawa Danbe’ or ‘Konsore’ kebeles may not attend all the visits citing the road and the transportation fee demanded by the motorbike drivers. As you know motorbikes are the only means of transport for our mothers in some kebeles.

**Interviewer: -**Are there any traditional, cultural, or religious practices that affect ANC services that you observed?

**Interviewee: -…….**Other than the ones I mentioned above, I can’t think of any now,

**Interviewer: -**How do you see the role of community volunteers /TBAs and health professionals in ANC service provision?

**Interviewee: -**Yeah, I think they are putting an effort into ANC service delivery… but according to me there needs to be a significant improvement, especially on health extension workers on keeping the regular fixed schedule on ANC service provision and awareness raising programs. They must conduct a follow-up visit regularly. But on some days the health posts are closed and the mothers don't get the services. I think this should be improved upon.

**Interviewer: -** Do women think skilled attendance during childbirth helps themselves and their babies?

**Interviewee** lot of work has been done to improve the attitude of the community in our area. The attitude of the community has been changing on facility delivery. Also, the community has seen the suffering of the complications that mothers go through when delivering at home, they have also seen how the mothers are helped during delivery in our facility like giving them IV fluids for mothers who suffered bleeding and removal of retained placenta and other complications. The community is thankful for the services and the mothers have a positive attitude towards facility delivery.

**Interviewer: -** What do you think are the causes of home delivery in your area?

**Interviewee: -** When I ask mothers why they delivered at home most of them reply that they have a fast labor and it didn’t give them time to reach the health facility so they delivered at home. Sometimes they tell you that the distance that they have to travel and the lack of relatives to bring them to the facility and some mothers still feel being seen by health care professionals as shameful. I have observed these problems.

**Interviewer: -** What could be the reasons for mothers who have used the ANC service but still yet, and delivered at home?

**Interviewee:** - As I told you before they tell you that the distance that they have to travel and the lack of relatives to bring them to the facility. I haven't heard anything also.

**Interviewer:-**Can you tell us traditional practices and beliefs during childbirth?

**Interviewee: -** I think I have discussed some of the traditional issues associated with delivery previously. ….. Another thing that I can remember is that one time there was one mother who was told by a local pastor that she is delivering a male child but she delivered a girl in our facility. And she was not happy and she refused to breastfeed the child. But we together with her relatives convinced her to breastfeed her child.

**Interviewer: -** How do you see the role of community volunteers/TBAs and health professionals in improving facility delivery?

**Interviewee: -** The community volunteers and the traditional birth attendants have been organized into a community organization called the health development army/HDA. These organizations help us a lot in our service delivery. They bring a laboring mother to our facility. And also thy call ambulances when there is a need for the ambulances. They also report to us whenever there occurs a home delivery. I think they are helping the service delivery in our area.

**Interviewer: -** What does the practice of PNC delivery look like in your facility?

**Interviewee: -** The PNC service that is given in our facility following the birth of a child is given between 6 to 8 hours after the mother has delivered the baby. Under the PNC service, we follow the mother s vital signs and for complications like post-partum bleeding, we also educate the mother about breastfeeding and observe her breastfeed the baby. We also give the mother post-partum family planning according to her choice and her will. If other complications are above our capacity we refer them. That is the PNC services we deliver.

**Interviewer: -** Do women think skilled attendance during PNC services helps their babies and themselves?

**Interviewee: -** In the past mothers who delivered in our facility wanted to go home as soon as they delivered. They say now the placenta is out of the uterus (delivered) and what other reasons we should stay in here (health facility). We need to go to our home and give food and drinks to the newly delivered mother and care for her and baby they say. But now after we teach them about the complications that can happen in a post-partum period such as bleeding and we give them incentives like coffee and wheat flour the mother and her assistants stay from up to 8 to 24 hours in our facility. The community has also seen postpartum complications cause serious problems to mothers and they accept our instructions and now they are using PNC care.

**Interviewer: -** Explain factors that would motivate women to utilize PNC services and the continuum of care.

**Interviewee: -** I would say that factors that motivate mothers to utilize PNC and the continuum care are our mothers' forum. Our mother's forums are undertaken monthly in each kebele various maternal topics in the presence of one health professional and the health extension worker. Comprehensive and participatory health education will be given to mothers about the pros and cons of using maternal services like ANC, SKILLED birth attendance, PNC, and the continuum of care…..the second factors are the different incentives given to our mothers who use our services such as flour, soap, and MAMA/FAFA programs.

**Interviewer: -** What are the barriers to attending PNC care and the reasons for discontinuation of care across the continuum?

**Interviewee: -** Most of the causes are the ones I mentioned above these are sometimes associated with lack of awareness, distance from the facility, costs of transportation, and sometimes lack of quality in our service delivery like power outage and unavailability of some medications.

**Interviewer: -** Can you tell us the traditional practices and beliefs during the postpartum period?

**Interviewee: -** Yes I have seen some harmful traditional practices. One thing I observed is people feeding a traditional medication called "Hameessa'' to a newborn child. This is very harmful to a newborn child as his digestive system is not ready for any thing other than breast milk. I have also observed some women in our facility use their mouths to suck on a newborn mouth claiming he may drink some secretions during his delivery. The other tradition that e have observed is that during postpartum care women in our community are cared for mostly by the relatives of the husband and the mothers may not openly communicate their needs with the husband's family. I think women will receive better care if they are cared for by their own families, especially for the first deliveries. We are trying to solve these issues by telling these people these practices are wrong and negatively affect the health of the mother and the newborn.

**Interviewer: -** How do you see the role of community volunteers /TBA in PNC care?

**Interviewee: -**As have told you they are doing a good job. They do follow and visit mothers in the PNC period. There are some cases in with theses volunteers have observed and brought mothers who developed complications to our facility. …..I also say we need to motivate and train these community volunteers to improve their performances.

**KII-03**

**Interviewer:** How early do women go for ANC?

**Interviewee: -** Women come at twelve weeks in our area. Some women who reside in distant parts of our kebele like near the Bilate River may come late like twenty weeks. Some other women may come as late as thirty-two weeks. As you know ours is a remote health facility and distance plays a role in mothers coming late.

When women arrive in our facility we receive them. We measure their weight, height, and blood pressure. Then we send them for all lab tests needed for pregnant mothers. After that, we give them deworming pills and iron folic tabs. After counseling we reappoint them they go to their home.

**Interviewer:** Why are the pregnant mothers late for ANC care?

**Interviewee: -** As you know ours is a remote health facility and distance plays a role in mothers coming late.

**Interviewer:** among the women who attend the ANC care properly what do you think are the factors that motivate women to use ANC care**?**

**Interviewer:** If women don’t use ANC services, what are their reasons?

**Interviewee: --** Some women in our community lack awareness**.** They hold onto ancient beliefs and say our ancestors never used modern services and they lived just fine**.**

**Interviewer:** what about other barriers like financial and distance barriers?

**Interviewee: -**As you know ours is a remote health facility and distance plays a role. The poverty level in parts of our catchment is high and mothers struggle with transportation costs. The roads are not well maintained, especially in summer times pregnant mothers struggle to come here.

**Interviewer:** Why do women discontinue subsequent ANC visits?

**Interviewee: -** Some women may not complete their ANC care visits. Some may miss appointments when inquire why? Most of them tell us issues with household work and child care and social life reasons such as funerals and weddings which they have to attend.

**Interview:** Can you tell us about the traditional practice and beliefs during pregnancy?

**Interviewee: -** No, there are none to that I know of. People of different religions and ethnicities come here and deliver in our facility. Sometimes generations of older mothers may prefer to deliver at home. That is due to the awareness gap. Sometimes it is essential to include them in health education.

**Interviewer:-**How do you see the role of community volunteers/TBAs, and health extension workers on ANC care service delivery?

**Interviewee: -** When these community programs were began these organizations worked extensively to improve awareness among the community. They contributed greatly to the awareness changes. But in recent times we have seen a weakening in their activity.

**Interviewer:-**My next question is about facility delivery. Do mothers know the benefits of facility delivery in your area?

**Interviewee: -** In the past, they were embarrassed by giving birth at the hands of professionals but when they saw the value of our care and the complications from home delivery they know have changed their feelings about professional care.

The motivating factor is primarily the improved awareness of the mothers on the benefits of facility delivery. This was achieved by the uninterrupted health education activities we are carrying out in partnership with the community health organizations like HDAs, health extension workers, and professionals from our facility. These health education operations we are doing are done in regular maternal forums and are supported at a community level by health promoters.

**Interviewer:-**for mothers who deliver at home, what are their reasons?

**Interviewee: -** as a said before those mothers who hold to past views, mothers who don’t use ANC care are less likely to deliver at home. They are less likely to know their expected delivery period and are less prepared and end up delivering at home. Some husbands may not be cooperative and may discourage facility delivery

**Interviewer: -** Do women present a reason for the lack of transport or lack of money as a reason for home delivery?

**Interviewee: -** As you know ours is a remote health facility and distance plays a role. The poverty level in parts of our catchment is high and mothers struggle with transportation costs. Distance and access issues defiantly play a role in home deliveries here.

**Interviewer:-**How do you see the job being done by the community health development army specifically about facility delivery?

**Interviewee: -** The community volunteers and the traditional birth attendants have been organized into a community organization called the health development army/HDA. These organizations help us a lot in our service delivery. They bring a laboring mother to our facility. And also thy call ambulances when there is a need for the ambulances. They also report to us whenever home delivery occurs. I think they are helping the service delivery in our area. You know this can't be said to all of them. There are excellent persistent workers and some can be found lagging in efforts even at the level of health extension workers.

**Interviewer: -** What does the practice of PNC delivery look like in your facility?

**Interviewee: -** We make the mothers stay for 24 hours at a health facility. We follow them for complications. We also observe their breastfeeding and advise them. We also counsel the mothers on postnatal FP and provide the service as necessary.

**Interviewer**: - Do mothers understand the benefit of PNC service to themselves and their newborns?

**Interviewee: -** Most of them understand but some others lack the proper awareness and want to leave the facility as soon as the mother delivers. And argue with us for early departure.

**Interviewer: -** If mothers don’t use PNC care what do you think their reasons are?

**Interviewee: -** Mostly due to home delivery and lacking awareness.

**Interviewer: -**How do you see the job being done by the community health development army and TBA specifically about PNC?

**Interviewee: -** as I have told you before we educate the mothers on every maternal topic and they helped greatly in achieving this.

**Interviewer:** - What do you think should be done to improve PNC care as well as the continuum of care?

**Interviewee: -** We need to harmonize our efforts with the community and give continuous health education and awareness creation activities. The quality of care at the facility needs to be improved by supplying the necessary inputs and improving infrastructure.

**Interviewer: -** I have finished my questions, thank you!

**Interviewee: -**Thank you!

**KII-04**

**Interviewer:** How early do women go for ANC? Why do they go at that time? Why earlier or later? How often do they go to ANC? Why do they go at that time?

**Interviewee: -** In the past, we can say mothers don't use ANC care but now awareness has improved and they have begun utilizing our services.

Most of the mothers came for the first visit at 12 weeks. We appoint them every four weeks until delivery. When they come we vaccinate them with TT and iron folate then we send them for lab tests of urine, blood group, and RH. Then we counsel them for danger signs, reappoint and send them home.

**Interviewer:** Why are the pregnant mothers late for ANC care?

**Interviewee: -** In the past, the mothers came late when their near term or when they see complications. It is the culture in this area. But now we recognized this problem. And we are educating them in maternal forums end we are observing improvements.

**Interviewer:** Among the women who attend the ANC care properly what do you think are the factors that motivate women to use ANC care**?**

**Interviewee: -** We are training the community and the mothers through our monthly maternal forums on the benefits of ANC care. The mothers have also seen how home deliveries hurt women through complications. There is also a job done to make MCH services accessible and affordable. These factors are helping to motivate mothers into receiving professional care.

**Interviewer:** If women don’t come for ANC in your catchment, what are their reasons?

**Interviewee: -** some women in our community lack awareness. They say our “ancestors never used modern services and they lived just fine**.”** Other women are shy and refuse to participate in our community forums. These people need a lot of pushing and forcing. Mostly it is due to a lack of adequate awareness that stops them from using professional care.

**Interviewer:** what about other barriers like financial and distance barriers?

**Interviewee: -** Yes, mostly some mothers complain about the cost of transportation for not using ANC care. Sometimes we suffer power outages and shortages of inputs and medications. During these times the mothers are less motivated to come here,

**Interviewer:** Why do women discontinue subsequent ANC visits?

**Interviewee: -**according to our last report only a quarter of the mothers appeared for all the visits. Mostly it is due to the mothers not coming if there is an immediate necessity. We are working to improve on this.

**Interview:** Can you tell us about the traditional practice and beliefs during pregnancy?

**Interviewee: -** No, there are none to tell of.

**Interviewer:-**How do you see the role of community volunteers/TBAs, and health extension workers on ANC care service delivery?

**Interviewee: -** yes**,** they do work to educate the community about the benefits of health care and the risks of not using it.

**Interviewer:** What should be done to further improve ANC care delivery?

**Interviewee: -** We should strengthen community engagement and health education about ANC care,

**Interviewer:-**My next question is about facility delivery. Do mothers know the benefits of facility delivery in your area?

**Interviewee: -** In the past, they were ashamed of birthing at the hands of professionals but when they saw the quality of our care and complications from home delivery they now have changed their attitude about professional care.

**Interviewer:-**What are the factors motivating mothers to attend skilled delivery at the hands of professionals?

**Interviewee:** The factors motivating them are they have seen how easily they can deliver when compared to delivering at home plus the health education and awareness increasing jobs done by community organizations are bearing fruit.

**Interviewer:-**For mothers who deliver at home, what are their reasons?

**Interviewee: -** as a said before those mothers who hold to past views, mothers who don't use ANC care are less likely to deliver at home. They are less likely to know their expected delivery period and are less prepared and end up delivering at home. Certain women are reclusive and prefer to not engage with professional care.

**Interviewer: -** Do women present a reason for the lack of transport or lack of money as a reason for home delivery?

**Interviewee: -**mostly they say the ambulance was late and their labor was fast and didn't give them time to reach the facility and deliver at home.

**Interviewer:-**How do you see the job being done by the community health development army specifically about facility delivery?

**Interviewee: -** they are doing a good job. They identify women and bring them to forums. They also participate in health education programs. They also play a role in linking laboring mothers with ambulance services as they have established contact with them.

**Interviewer:-**What do you think should be done to improve facility delivery as well as the continuum of care?

**Interviewee: -** We need to coordinate our efforts with the community and give continuous health education and awareness creation activities. The quality of care at the facility needs to be improved by supplying the necessary inputs and improving infrastructure. The mothers should also have birth preparedness and a birth plan.

**Interviewer:-**What does PNC service look like in your facility?

**Interviewee: -** We make the mothers wait at our facility. We follow them for complications. We also counsel the mothers on post-natal FP and provide the service as necessary. We also observe their breastfeeding and advise them.

**Interviewer**: - Do mothers understand the benefit of PNC service to themselves and their newborns?

**Interviewee: -** Most of them understand but some others lack the proper awareness.

**Interviewer: -** If mothers don’t use PNC care what do you think their reasons are?

**Interviewee: -** Mostly due to home delivery and lacking awareness.

**Interviewer: -**How do you see the job being done by the community health development army and TBA specifically about PNC?

**Interviewee: -** as I have told you before we educate the mothers on every maternal topic and they helped greatly in achieving this.

**Interviewer:** - What do you think should be done to improve PNC care as well as the continuum of care?

**Interviewee: -** We need to coordinate our efforts with the community and give continuous health education and awareness creation activities. The quality of care at the facility needs to be improved by supplying the necessary inputs and improving infrastructure. The mothers should be tough and urged to have birth preparedness and birth plan.

**Interviewer: -** I have finished my questions, thank you!

**Interviewee: -**Thank you!

**KII-05**

**Interviewer:** How early do women go for ANC? Why do they go at that time? Why earlier or later? How often do they go to ANC? Why do they go at that time?

**Interviewee:** - Mothers do come for ANC care. When they present to our facility we record their history. After that, we send them to essential lab tests like PIHKT, RH, anemia, and other diagnostics. After that, we give them essential treatments like iron supplements and deworming them according to the results of their tests. Then we give them an appointment and send them home with advice about danger signs. In terms of being late, some mothers start their care late. As most mothers who use our services are from rural areas awareness differs among users of our services. The reason those late mothers tell us is that they were busy with other social life problems like funerals and weddings. Some also say they were sick and couldn't come. But most of the reasons for being late are not prioritizing the ANC care and being engaged in other stuff. But we could say attitudes, in general, have been improving.

**Interviewer:** Among the women who attend the ANC care properly what do you think are the factors that motivate women to use ANC care**?**

**Interviewee: -** Mothers are motivated by what they have seen during professional care. Mothers comparing the quality of care given by professionals with their past experiences at the hands of traditional care are motivated to receive ANC care. The other thing motivating the community is their improving awareness about the benefits of professional care. We work persistently to improve the awareness of the community for example I go out to the field every fifty days to give health education to mothers. These coupled with other mass media health education are helping to motivate the community for ANC care.

**Interviewer:** If women don't use ANC, what are their reasons?

**Interviewee:** - it is mostly due to a lack of adequate awareness. Some mothers in the countryside may say we will be fine delivering at home like our ancestors.

Other factors can be a lack of proper support and discouragement by family members of the woman to modern professional care.

**Interviewer:** What about other barriers like financial distance and barriers associated with quality of care?

**Interviewee:** - There can be some mothers who may not be aware that ANC services are being given for free and may be discouraged from coming but when we educate them we tell them all of ANC care is given for free. I have seen mothers preferring certain individuals over others but I have not seen it being a significant barrier.

**Interviewer:-**How do you see the role of community volunteers/TBAs, and health extension workers on ANC care service delivery?

**Interviewee:** - I can say they are doing a good job. Mostly they deal with health extension workers. They may know more.

**Interviewer:** What should be done to further improve ANC care delivery?

**Interviewee:** - I say all the basic health infrastructures are there. I say strengthening of activities.

**Interviewer:-**My next question is about facility delivery. Do mothers know the benefits of facility delivery in your area?

**Interviewee:** - The awareness of our mothers has improved. When their labor commences they don’t wait at home. They present themselves to our facility. That is because they have known the benefit of professional care. Our facility is a primary hospital. Mothers who come to us will receive the necessary delivery care according to their needs. Some women come by referrals when they came we run the necessary tests. For mothers who can deliver by SVA or those who deliver by CS all of them will be treated accordingly.

**Interviewer:-**What do you think are the factors that motivate women to use HFD service**?**

**Interviewee: -** Our mothers are motivated to deliver at the facility because of the standards of care that they are receiving. When they compare it with home delivery with many complications they prefer the contemporary care.

**Interviewer:-**For mothers who deliver at home, what are their reasons?

**Interviewee:** - Mothers who don’t use ANC care are more likely to deliver at home. Other mothers who lack birth preparedness and not having the necessary contacts for ambulances we have seen deliver at home. Even when the ambulances have been called they may not reach in time and deliver on the road or their way to the facility.

**Interviewer: -** Do women present a reason for the lack of transport or lack of money as a reason for home delivery?

**Interviewee: -** I have not met them personally but there can be some people with not many even for food and may choose for home delivery.

**Interviewer:-**How do you see the job being done by the community health development army specifically about facility delivery?

**Interviewee: -**It is good. They identify risky pregnancies and they send them to health facilities. They educate mothers

**Interviewer:-**What do you think should be done to improve facility delivery as well as the continuum of care?

**Interviewer:** Extensive health education delivered at all stages of services to mothers. In addition to improving the quality of care at all stages of service delivery.

**Interviewer: -** What does the practice of PNC delivery look like in your facility?

**Interviewee: -** The PNC service that is given in our facility following the birth of a child is given for 24 hours after the mother has delivered the baby. Under PNC care we follow the mother's vital signs and for complications like danger signs and post-partum bleeding, we also educate the mother about breastfeeding and observe her breastfeed the baby. Another treatment is given to the mother as necessary.

**Interviewer: -** Do mothers understand the benefit of PNC service to themselves and their newborns?

**Interviewee: -** Most mothers understand that PNC services are essential to them and their babies. But some mothers don't.

**Interviewer: -** if mothers don’t use PNC care what do you think their reasons are?

**Interviewee: -** Mothers miss PNC care if they don’t deliver in a facility. If mothers deliver at home it is impossible to give them PNC.

**Interviewer: -**How do you see the job being done by the community health development army and TBA specifically about PNC?

**Interviewee: -** we can say they are doing a decent Job. That is by observing some cases that they have sent to our facility. But I don't consider myself an expert on this subject as I don't deal with these people directly.

**Interviewer:** - What do you think should be done to improve PNC care as well as the continuum of care?

**Interviewee: -** About our facility, we can further improve the quality of our services if we can get the proper health equipment. For example, we need a Doppler fetoscope, ultrasound, and baby suction machine for distressed newborns. We also lack adequate running water as a primary hospital. We also sometimes lack medications and essential equipment like gloves.

Also, all stockholders from the community to professionals to upper managers should work with proper emphasis to fulfill services at all points of service delivery.

**Interviewer: -** I have finished my questions, thank you.

**Interviewee: -** Thank you!

**KII 06**

**Interviewer:** How early do mothers go for ANC in your locality?

**Interviewee:** They will come within the first trimester (before 16 weeks) for ANC service. However, the limited numbers of mothers attend their ANC visit within this period. The mothers from educated families, educated women like teachers, and those who have other professions will come early to HF to confirm their pregnancy by HCG test and start their first ANC service at least in the third month after being assured of their pregnancy. Most of the time majority of them will attend their first ANC visit around the 6^th^ or 7^th^ month.

**Interviewer:** why do they come at that time most of the time?

**Interviewee:** first, they have a shortage of awareness. Previously HEWs had been workhouse to house and identify at least five pregnant women per day. In the meantime, they will create awareness regarding the ANC visit in the community. Now they have based their work only on the health post level and that is why I said the shortage of awareness because most of the time they provide the services for those mothers who only visit health post. Second, the pregnant women's forum has been interrupted and weakened in our area. The majority of women believe in the benefits of pregnant women's forums by interpreting in terms of the benefits they provide for example FAFA, flour, and oil. Most of them are attending the ANC visits despite there haven't awareness.

**Interviewer:** Why do they go earlier or later than above stated time?

**Interviewee: They have family responsibilities. For example, those women who have three or more children are highly busy preparing food, caring for younger children, fetching water, keeping animals, etc. Most times, due to these reasons, they will not go to HF or become late for an ANC visit.** Also, those mothers whose husband hasn't supported them go to HF lately in a rural locality. The majority of husbands in the rural area prefer the women to become busy with their workload rather than to follow ANC visit. However, in the urban area, their husband is supportive and goes together with them to HF. Due to these reasons, they go to the health facility after their abdomen becomes very big or large. Even those mothers lately go to HF for the purpose to obtain TT vaccination but not for ANC service. The TT vaccination is a big deal for them and they believe ANC visits a TT vaccination.

**Interviewer:** How many times they will come for ANC follow-up?

**Interviewee:** as I have mentioned earlier the women from the better family will come four times. However, the majority of them will come two times after several efforts or push following the first contact. Even those women will come for ANC visits due to faced health problems like hypertension and syphilis etc.

**Interviewer:** Do women think skilled attendance during pregnancy helps their pregnancy?

**Interviewee:** yes, they think it is very important. However, several factors influence women not to come to the health facility. First, the long waiting time in ANC rooms due to the shortage of HCP providers. In our health facility, the ANC services are provided one day per week in a fixed scheduled manner. During that day they will come collectively together but one health professional is assigned to handle all activities in MCH. The standard says that women need to obtain all contents of ANC service including adequate counseling within a minimum of 30 minutes. However, in one room we are providing many services like ANC and family planning. Besides, due to high cases flow we rush to provide service to all women that are one reason they are unsatisfied. Further, most times due to electricity interruption they may not obtain the few existing laboratory services. Moreover, they will return to their home without obtaining any service if that health professional attended delivery. Look at he/she as one person who can handle the women in the ANC room as well as laboring women in the delivery bed. Please judge me in place of this health professional but I focus on laboring women and give the next appointment for ANC women. Those women will not come again to a health facility for ANC visits due to these reasons.

**Interviewer:** could you explain the factors that would motivate women to utilize ANC service?

**Interviewee:** the TT vaccination. They perceive the TT vaccine as very important to speed up labor that why they are motivated to visit ANC. I think no one correctly explain to them the benefits of the TT vaccine that they wrongly perceived. Also, I think they were misinformed about the benefit of the TT vaccine during the pregnant women forum that was conducted at health post levels. The TT vaccine is a big deal for them that motivates them to use ANC service despite the many services provided during ANC visits. For instance, laboratory investigations such as hemoglobin and hematocrit level determination, VDRL test, and urine analysis conducting during follow-up. Besides, the physical examination is very important to check their fetal presentation and position. Despite these all services during ANC visits they focus and complain about the number of TT vaccines. They mainly come back to the health facility after the first contact of the ANC visit because of to obtain the remaining TT injection. Sometimes we try to explain to them the difference between the TT vaccine and ANC visits. However, unable to explain all of them because we are too busy with our workload and large case flow. Loot at all these cards is ANC visit cards I examined today and very difficult to adequately counsel these all women about the benefits of ANC. Shortage of time to provide adequate advice due to I measure blood pressure, and weight and send for laboratory investigation. However, we exert our maximum efforts to counsel about ANC. Surely they come for ANC services because of to obtain reaming TT injection.

**Interviewer:** Did they perceive the TT vaccine as a big ANC service?

**Interviewee:** yes, perfectly

**Interviewer:** What are the barriers to ANC use?

**Interviewee:** the barriers related to health facilities?

**Interviewer:** Yes?

**Interviewee:** the long travel distance from home to the health facility. We have two catchments namely Hanja Cafa and Konsore Cafa. The Hanja Café catchment is very far from the health center that is bordered by the main Asphalt. If women did not obtain adequate ANC services in first contact after they have traveled a far distance that is one reason that influences the women not to come back again. In our district (Boricha) the topography isn't a big problem but it is not free from hills and valleys. However, the far distance is one reason that makes women negligent of ANC visits due to the tiredness they felt. Other reasons like an interruption of electricity and poor quality service. Sometimes women not only for the ANC service but also for child vaccination weren't coming to health facilities due to long distances and we are addressing this by using outreach programs.

**Interviewer:** Are there any financial and transport-related barriers that influence ANC use?

**Interviewee:** yes, if women are denied money and transport will not go to health facilities.

**Interviewer:** Are there any socio-cultural-related barriers?

**Interviewee:** yes. For example, in our community, most of the time husbands do not come together with their wives for ANC service (the husband's involvement is very minimal) even from educated families. The mothers are negligent for the ANC service use and their spouse's support is very important to motivate them. However, there is no pressure from the husband's side to influence the use of the service in our locality.

**Interviewer:** Are there any mother/father-in-law-related barriers?

**Interviewee:** no

**Interviewer:** Are there any ANC qualities of care-related barriers?

**Interviewee:** yes. As I have mentioned the earlier majority of women are unsatisfied with ANC service due to long waiting times, shortage of HCPs, inadequate counseling service, and electricity interruption. For example, one woman becomes angry and goes back to her home without obtaining ANC service due to the long waiting time. There is one fixed ANC schedule (always Thursday) per week and comes collectively from both catchments on that day. Due to this reason, it is beyond the capacity of one health professional to manage all mothers. It is not possible to provide quality ANC service as per standard due to time shortage.

**Interviewer:** Why do women come to the facility for first ANC, but discontinue for 2^nd^, 3^rd^, and 4^th^ ANC visits?

**Interviewee:** the first time visit the health facility around the 4^th^ or 5^th^ for TT injection and we will provide them the first TT vaccine. Then, we will provide them the next appointment after one month for the next ANC visit but they come after postponing their appointment. Even during this time, they are coming for TT injection purposes but not ANC service. After they obtained the second dose of TT injection they will perceive the ANC follow-up completed and not come again health facility. This is the main reason that influences them not to complete the ANC visit as per standard. However, we are advising them to change this behavior and proving the service for them.

**Interviewer:** are there any other barriers other than TT vaccine misperception that lead to discontinuous the subsequent ANC visit?

**Interviewee:** the lack of money and distance accounts for the big place. They (HEWs) at the health post level only provide the TT injection for ANC women but do not check fetal presentation and position, weight and blood pressure measurement, and laboratory service. They send mothers to come health centers for laboratory investigation but mothers will not come to health centers due to far distance and lack of money. Several women remain at home without using ANC services due to poverty. They are ashamed to come to health facilities because they are under-nutritional and aren't clothes to wear due to poverty. Thus, poverty is the main barrier to hindering ANC service use.

**Interviewer:** Can you tell us about the traditional practices and beliefs during pregnancy?

**Interviewee:** the abdominal massage and suffocating mouth during delivery. Even in health facilities in delivery beds after we leave the room to bring some equipment they will automatically start massaging and suffocating the laboring mother. If the labor started at home they seriously and several times try abdominal massage and suffocating mouth during delivery. They will bring the woman after she develops serious complications like bleeding, tearing, etc. They also delay bringing women as soon as the labor started due to the spiritual practice. Most of the time they bring the laboring women to health facilities after several time elapsed by pray and problems worsened.

**Interviewer:** in your opinion, what should be improved to improve the quality and use of ANC services?

**Interviewee:** I think all mothers need to obtain ANC service at all times as they come without providing the next appointment. As I have mentioned earlier in our health facility the ANC service is provided based on a fixed schedule (once per week). The electricity interruption should be minimized. Adequate numbers of qualified and experienced healthcare providers should be available at all times in health facilities. The women need to adequately counsel to avoid misunderstanding ANC service. For example, women can't receive many important drugs provided based on a diagnosis like Albendazole during pregnancy even if they have serious problems. The husband should come together with his wife for ANC service. The husband should remind her about the appointments and support her to go health facility by arranging the transport. They should to go health facilities even if no transport walking together. If these things improved they will come to health facilities for ANC service without any interruption.

**Interviewer:** Do women think skilled attendance during childbirth helps them and their babies?

**Interviewee:** yes, they think as helpful. They all also understand the benefits of health facility delivery by skilled health providers. However, some barriers hinder women not to utilize institutional delivery care. I as described above like long distances from health facilities, shortage of money and lack of transportation, etc. For instance, the ambulance only provides the referral service from here to the hospital but not receiving the laboring women from their homes. Some also haven't towels, hats or caps, and small materials which are important to their babies after delivered here due to poverty. Due to these reasons, they prefer to give birth at home even though they think very important.

**Interviewer:** are there any other barriers rather than these?

**Interviewee:** no other barriers.

**Interviewer:** Explain factors that would motivate women to utilize delivery services.

**Interviewee:** sometimes there is an event that motivates them to come here. For instance, I think in 2014 E.C., the women were delayed in a home for a long time after labor started due to pray and developed a serious complication. They brought the women here and we referred them to Leku Hospital. Then, the Leku hospital was also referred to a referral hospital but women died there. If they hear this kind of event all of them will go health facility for institutional delivery care. They will come by fear. Most times the pregnant women forum motivates them to attend health facility delivery. During the forum, the midwife will provide detailed information about the obstetric danger signs and counsel them. However, currently, there is a shortage of midwives here in our facility that why I am here (MCH room), and no forum currently. If we start the forum those women who didn't use ANC visit may come for institutional delivery care due to fear of complications that happen during childbirth. They also fear the baby's suffocation during childbirth.

**Interviewer:** are there any other motivating factors rather than these?

**Interviewee:** the educated family will bring them soon the start of labor. Another reason after they gave birth at the health facility we provide the health facility birth notification certificate. That certificate is a very important pre-requisite to obtaining many services like free child vaccination cards, kebele loan programs, and HEWs under-nutrition programs. Many poor women will come for institutional delivery by considering these benefits. The awareness raised during the pregnant women's forum about the danger signs will motivate them to obtain the service.

**Interviewer:** in your opinion, what should be improved to improve the health facility services from the government, community, and health facility sides?

**Interviewee:** first, for those mothers who have far apart from the health facility the inaccessible road is the main thing that needs to be improved to improve health facility delivery. Other awareness creation should be conducted not only by pregnant forum but also public meeting that includes different audience like male sex, religious leaders, community members, and leaders. Because women are highly dependent on their husbands in our culture and support from this side is very critical to the improvement of health facility delivery. The woreda-level governmental official should be decided to expand the ambulance service at a community level. Bringing the laboring women from home to the health center is a challenging issue by motorcycle or local stretcher. If these things improved I think nothing else will hinder women from using the health facility delivery. Male partner awareness creation has superior benefits over the merely pregnant women forum. The awareness creation of male partner involvement is a very critical issue to improve health facility delivery not only in our community but the whole of Ethiopia because the women are highly dependent on their husbands. If the male partner actively participated the women will use health facility delivery service by any means even those from inaccessible road areas (they will bring laboring women by using foot, local stretcher, motor, and car). The health center should strengthen the pregnant women forum by allocating the existing midwife. For instance, as you know the Malga district is one of the highest maternal mortality recorded districts in the Sidama region. Maternal mortality declined and maternal health service utilization increased immediately after the strong and comprehensive public forum. The forum comprised medical doctors from the hospital, midwives, woreda and Kebeles level administrators, women, and the larger society. Our community will accept the messages of educated people who have professional skills. If this experience is scaled up in our area the health facility delivery service will completely improve or the number of women that use the health facility delivery increase.

**Interviewer:** How early do women go for PNC?

**Interviewee:** if the mother gave birth in our health facility we discharge them between six and twenty-four hours due to a shortage of beds and room. However, they will come again on the 45^th^ day for child vaccination and family planning service unless they have experienced health problems. Sometimes they will come between when in case experienced complicated health problems.

**Interviewer:** How many times do they use the PNC service?

**Interviewee:** they only come on the 45^th^ day for the child vaccination purpose.

**Interviewer:** for what reasons do they come in 45^th^ days?

**Interviewee:** for the child vaccination and family planning service purpose.

**Interviewer:** Do women think skilled attendance during postpartum help their babies and themselves?

**Interviewee:** they think as important. However, in this area due to traditional beliefs, the delivered mothers are not allowed to go outside the house for two to three months. Most of them pass their time in the home garden area and are not allowed to walk the long distance.

**Interviewer:** are there any other barriers that influence the women attending the PNC service?

**Interviewee:** the long distance and lack of money for transport are the main other reasons that influence women will not use PNC as per schedules such as the 3^rd^ day, 7^th^ day, and 6 weeks after first contact.

**Interviewer:** Can you tell us about the traditional practices and beliefs during the postpartum period?

**Interviewee:** traditional practice in babies after delivery?

**Interviewer:** yes, both in mothers and babies?

**Interviewee:** previously they applied the animal dung on the babies' umbilical cords but now this practice has stopped. Now they are careful in this regard but they start to use 'Amessa' as babies feeding fluid. Due to this substance, they will bring the babies by complaining the abdominal pain before 2 months of delivery.

**Interviewer:** why do they provide the 'Amesa’ for the babies?

**Interviewee:** they will perceive the ‘Amesa' as helpful for the measles and prevent the babies from the 'Buda'. They also apply in the body of the baby because they perceive it will protect the 'Buda'.

**Interviewer:** could they believe the Amesa prevent measles?

**Interviewee:** yes. They apply to the body of the baby because of the suspected measles. Besides, they believe it protects babies from intestinal parasites and prevents diarrheal disease.

**Interviewer:** what other benefits do they perceive of 'Amessa’?

**Interviewee:** they also think the ‘Amesa' will make baby fat or big and provide energy for them.

**Interviewer:** In your opinion, what should be improved regarding PNC services?

**Interviewee:** if the mother gave birth at a health facility strong counseling that involves the mother, her husband, and family about the PNC service should be designed. Provision of detailed information on what women can potentially obtain if come back again for PNC service as per schedule. Also, detailed information should be provided about the problems that women possibly face if not used the PNC service. If they are aware of PNC at this time, they will come for the service.

**Interviewer:** In your opinion, what should be improved regarding PNC services from the government side?

**Interviewee:** as I have mentioned to you earlier the mothers relate everything with benefits. Previously women obtain TTC eye and chlorohexidine ointment, caps, and towels from a health facility which motivates women to go to PNC service. However, these all materials have been stopped since the start of the conflict and the government should avail it. Even those educated families did not come by themselves for PNC service. Quality service by experienced healthcare providers should be provided to improve the PNC service. Healthcare providers should be further trained in this service area. The Woreda office needs to strengthen the awareness creation programs for the general public. I have finished on my side.

**Interviewer:** anything that you want to add to our discussion so far?

**Interviewee:** no

**Interviewer:** Thank you a lot!!!

**KII-07**

**Interviewer:** How early do women go for ANC? Why do they go at that time? Why earlier or later? How often do they go to ANC? Why do they go at that time?

**Interviewee: -** We are serving 3 kebeles in our facility. Women used to come late like after six months of pregnancy. But awareness is changing and times are modernizing women are coming as early as three months of pregnancy. They will come and receive essential ANC services like iron supplementation and TT vaccines as soon as they come. In the past, we used to give our ANC care package by four visits but now we do it by eight visits. These are four essential visits to our facility and the other four visits to the nearby health post.

**Interviewer: A**mong the women who attend the ANC care properly what do you think are the factors that motivate women to use ANC care**?**

**Interviewee: -** The motivating factor is mainly the improved awareness of the mothers on the benefits of ANC care. This was achieved by the continuous health education activities we are carrying out in collaboration with the community health organizations like HDAs, health extension workers, and professionals from our facility. These health education campaigns we are doing is done regularly by using maternal forums and health education at a community level by health promoters.

**Interviewer:** As you have said before not all mothers receive ANC care. If women don’t come for ANC, what are their reasons?

**Interviewee:** Yes, there can be many reasons for mothers not using ANC care. According to my experience, mothers who are pregnant out of wedlock most of the time don't use ANC care. Another reason is mothers who don't use our other services like family planning are less likely to use ANC care. Mothers who don't use birth spacing are less likely to use ANC care. The other reason is the lack of support from the family of the mothers. Some people in the mother's family may discourage the mother from attending ANC care by talking negatively talk about modern care. But most of the reasons for not using ANC care are associated with a lack of complete awareness by the mother about the benefits and risks of not using medical interventions.

**Interviewer:** What about other barriers like financial and distance barriers?

**Interviewee:** As I have told you before we serve three kebeles one of the kebeles Allawo Arke is remote and lacks proper road. The topography is also rough. Some mothers from their face distance barriers. In terms of cost, almost all of the services are given for free in our facility. ANC is free, lab tests are free, and delivery services and ANC services are given for free. But some mothers may need a referral for certain complications and may be demanded payment there. They may not have the means so they discontinue the service altogether.

**Interviewer:** Why do women discontinue subsequent ANC visits?

**Interviewee:** Once initiated most mothers do go on and finish their care and deliver in our facility. But the issue of discontinuation is associated with a lack of awareness. Other times some mothers who are not educated may forget one appointment date and stop coming for subsequent visits altogether. So we need to work further in personalizing ANC care to the specific situation and the need of the mother.

**Interview:** Can you tell us about the traditional practice and beliefs during pregnancy?

**Interviewee:** In the past there were but currently most of the harmful traditional practices are being eliminated by our society.

**Interviewer:-**How do you see the role of community volunteers/TBAs, and health extension workers on ANC care service delivery?

**Interviewee:** When the community programs were initiated these organizations worked extensively to improve awareness among the community. They contributed greatly to the awareness changes. But in recent times we have seen a weakening in their activity. I don't know why that happened but it can be associated with a lack of attention and motivation by the responsible bodies. Other reasons can be because the community has begun to come to the facility on their own some members thought their work is over.

**Interviewer:** What should be done to further improve ANC care delivery?

**Interviewee:** Strengthening the awareness creation activities and health education to the community. The other thing is delivering quality services in our facility. This begins by creating a well coming environment in our facility. Making quality services available in our facility at all times. In this aspect, we have observed limitations in our facility, as there are frequent interruptions to our electricity and lab services. Other services like maternal waiting rooms are not built in our facility and also we have problems with running water and our delivery area is not as suitable as it should be. The quality of services is essential in the retention of mothers.

The other thing is readying all our staff by improving their skills knowledge and attitude and working and being in among the community. If we asses our performance by the above criteria I thing we can frankly say we are found wanting.

**Interviewer:-**My next question is about facility delivery. Do mothers know the benefits of facility delivery in your area?

**Interviewee:** yes, awareness has improved greatly in our area. The community has learned greatly from past experiences and doesn't hold laboring mothers at home and they present them to our facilities. If home deliveries occur they occur due to the mothers, especially multipara women having fast labor and delivering at home. They understand the benefits of delivering at the hands of professionals and

**Interviewer: -** Do women present a reason for the lack of transport or lack of money as a reason for home delivery?

**Interviewee:** Most of our services are given without any payment. We have seen mothers who prefer to deliver In other nearby facilities other than ours that is due to the lack of electricity in our facility. The solar panels that have been installed are also not functioning. If mothers go to other facilities this is the feedback that we get.

**Interviewer:-**How do you see the job being done by the community health development army specifically about facility delivery?

**Interviewee:** as I have said before although there are lagging of activities in recent times but I can say they have done a substantially good job.

**Interviewer:-**What do you think should be done to improve facility delivery as well as the continuum of care?

**Interviewee:** as I have told you before what we need is a coordination of our activities with the community-level actors and improve awareness. And also we need to improve the quality of care in our facility. But these all need the input of the stakeholders including the woreda health bureau. As I have said our facility lacks some basic infrastructures such as maternal waiting rooms to electric power supply. These problems need the participation of all the players including NGOs. In addition, if we increase and strengthen our awareness creation of jobs we can achieve better results.

**Interviewer:-**What does PNC service look like in your facility? Do mothers know about PNC care?

**Interviewee**: It can be said most mothers understand that PNC services are essential to them. But this can't be said for all mothers.

**Interviewer: -**How do you see the job being done by the community health development army and TBA specifically about PNC?

**Interviewee: -** I think they are doing a good job but they need to be strengthened and given renewal and refresher training.

**Interviewer: -** What do you think should be done to improve PNC care as well as the continuum of care?

**Interviewee: -** Yes, as I have said earlier before if we can harmonize our efforts, we can improve PNC care and continuum of care. As we as professionals show concern and adequately follow the mothers through the continuum of care better services can be delivered to mothers. The most important thing is giving the right focus and coordination efforts.

**Interviewer: -** I have finished my questions, thank you.

**Interviewee: -** Thank you.

**KII-08**

**Interviewer:** What does the practice of ANC care look like in your area? Do mothers come and receive ANC care in your facility?

**Interviewee:** Yes, that is a good question. Mothers do come for ANC but it is difficult to say all of them come. Even among those who come they need to be told by HDA campaigns to come and receive ANC care. In terms of the mothers coming early and late for ANC care. Some mothers come early. These mothers are the most aware they know the benefits of ANC care and understand the danger signs during pregnancy. While the others who come late and miss out on ANC care are the ones lacking knowledge and awareness.

**Interviewer:** Do women think skilled attendance during their pregnancy helps their pregnancy? Do the mothers come for all the visits in ANC care?

**Interviewee:** Yes, From time to time mothers are changing their attitude about ANC care. The mothers who are initiated in our ANC care routine do continue and finish. That is because we urge them to finish the routine. We have recently introduced 8 ANC contacts with 4 through visits. Some active mothers do come even when there before their appointment when they don't feel well. But I can say most mothers do come and finish when they are initiated into the care routine.

**Interviewer:** Explain factors that would motivate women to utilize ANC services and the continuum of care.

Interviewee: Yes the motivating factors are there was a strong awareness creation job we have done with the help of HEWs to improve the utilization of ANC care. I think we are seeing the fruits of that job. The other thing is we have made an effort to improve the quality of the service delivered to mothers. That helped us through the mothers who used themselves to become an advocate for our ANC services. In this aspect we have improved the lab services that we give such as PIHCT, blood type, blood pressure, and other important investigations are delivered for free. The other factor is the negative outcomes that the mothers who deliver at home face such as bleeding and placental retention which cause major complications. The mothers in our community have observed the things that occur to women who don't use professional care. The factors combined have helped to motivate mothers to use the ANC services we deliver.

**Interviewer:** If women don’t use ANC care, what do you think are their reasons?

**Interviewee**: **The major reasons are that some women do not know the benefits of ANC service and lack awareness of the negative impacts of not using ANC care.** The other reasons can be shyness and fear among some first-time pregnant mothers.

**Interviewer:-**What do you think are the barriers to attending ANC services in your area? Do women tell financial and distance issues as a barrier?

**Interviewee: -** Every service for pregnant women in our facility is a free service so financial costs cannot be a major reason for non-use. Interims of distance most of the services in eight visits

ANC care can be given in a health post. I have heard some mothers complain about some drugs not being available in our pharmacy. That may have impacted the quality of care given in the facility.

**Interview:** can you tell us about the traditional practice and beliefs during pregnancy? Which negatively impacts ANC care?

**Interviewee:** in the past there were but currently most of the harmful traditional practices are being eliminated by our society.

**Interviewer:-**How do you see the role of community volunteers/TBAs, and health extension workers on ANC care service delivery?

**Interviewee:** it's a good question. In the past, these HDA and associated community organizations used to identify pregnant women in every district and bring them to the facility. They were able to eliminate the traditional care by birth attendants in the community during home deliveries. They also used to give health education to the community with great motivation. But now these services by the HDA have been interrupted. Their members are not as motivated as when they began their job.

**Interviewer: -** What should be done to further improve ANC care delivery in your area?

**Interviewee: -** I think we can improve ANC care if we engage in and work in our community. If we strengthen and motivate the HDA and the community organizations, I think we could achieve better results. The monthly meetings and health education for the women and the community needs to be strengthened too.

In terms of quality of care if we can fully supply essential drugs and deliver lab tests without interruptions we can satisfy our mothers and reduce discontinuation of care.

**Interviewer:-**My next question is about facility delivery. Do mothers know the benefits of facility delivery in your area?

**Interviewee: -** Yes, the mothers come and deliver in our facility. When the mothers suspect their labor has commenced they present themselves to us without, anyone motivating them to do so. When they come we give them all the necessary care within our capacity both to the mother and the newborn. When the community observes that complications can be easily avoided by professional care during delivery, it is further improving skilled birth attendance.

**Interviewer: -** what do you think are the factors that are motivating mothers to deliver at health facilities? And utilize the continuum of care?

**Interviewee: -** the skilled birth attendance is being improved by the awareness-increasing jobs that have been done in the community. We have monthly forums for pregnant mothers. A pregnant mother attends at least six forums before she delivers. During these forums, we try our best to make mothers fully aware of all the important issues of MCH and skilled attendance. Mothers who have a better awareness and knowledge always deliver in a health facility.

**Interviewer: -** For mothers who deliver at home what are their reasons?

**Interviewee: -** Most of the mothers give having rapid labor as a reason. They say that their labor was fast and didn't give them time to reach a health facility would say this is due to the lack of birth preparedness and a lack of a birth plan. Some new mothers sometimes feel ashamed to deliver at the hands of professionals. Sometimes some families fail to encourage women to deliver in health facilities. These people do that by using the word salads to make the mothers overlook the risks and complications. We have observed these things to be the reasons for home delivery in our area.

**Interviewer: -**Did you meet mothers who told you that finance, access, and quality of care are a reason?

**Interviewee: -**No, I have not any mothers who have presented those issues for delivering at home in our area.

**Interviewer: -** How do you see the role of community volunteers/TBAs and health extension workers in facility delivery?

**Interviewee: -** I believe they are doing good. They tell us when the mothers deliver at home, they organize forums and motivate mothers to deliver at a facility. They call ambulances for laboring mothers. But as I have said before these organizations need to be further strengthened and additional renewing training should be given to their members to motivate them in their efforts.

**Interviewer:** What should be done to further improve facility delivery and the continuum of care in your area?

**Interviewee:** Yes, if the community organizations show concern and adequately follow the mothers through the continuum of care better services can be delivered to mothers. If the responsible bodies support the community organizations through training and renewal, if we as professionals show concern and adequately follow the mothers through the continuum of care better services can be delivered to mothers. Necessary inputs in terms of drugs, lab reagents, and improving the infrastructure in our facility like water and electricity, I think we can retain mothers in a continuum of care.

**Interviewer: -** What does the practice of PNC delivery look like in your facility?

**Interviewee: -** The PNC service that is given in our facility following the birth of a child is given for 24 hours after the mother has delivered the baby. Under the PNC service, we follow the mother's vital signs and for complications like danger signs and post-partum bleeding, we also educate the mother about breastfeeding and observe her breastfeed the baby.

**Interviewer: -** Do mothers understand the benefit of PNC service to themselves and their newborns?

**Interviewee: - M**ost mothers understand that PNC services are essential to them and their babies. But some mothers don't.

**Interviewer: -** if mothers don’t use PNC care what do you think their reasons are?

**Interviewee: -** Mothers miss PNC care if they don’t deliver in a facility. Otherwise, mothers who deliver in a facility always receive PNC care. If mothers deliver at home it is impossible to give them PNC care. So that the biggest reason for mothers not receiving PNC care is home delivery.

**Interviewer: -**How do you see the job being done by the community health development army and TBA specifically concerning PNC?

**Interviewee: -** we can say they are doing a decent Job. That is by observing some cases that they have sent to our facility. But most of the HDA service is supervised by HEWs and HEWs may know more about this question.

**Interviewer:** - What do you think should be done to improve PNC care as well as the continuum of care?

**Interviewee: -** About PNC care some mothers may be poor and may not have something to eat during their stay in our facility. I have seen mothers who have been abandoned by their husbands. We may need some flour and coffee to prepare porridge and to make them stay for them. We may need support in delivering these items. About the continuum of care, I think If all the stakeholders of MCH Care can coordinate their efforts with us and the community organizations, I think we can do a good job.

**Interviewer: -** I have finished my questions, thank you.

**KII-09**

**Interviewer:** what does the practice of ANC care look like in your area? Do mothers come and receive ANC care in your facility?

**Interviewee:** concerning ANC care mothers in our area do come to our facility and receive ANC care. But most mothers begin their ANC care late. Once they begin their ANC care we enroll and educate them through maternal conferences and forums so that they complete their visits and deliver in our facility.

**Interviewer:** Why do the mothers come late for ANC care?

**Interviewee:** The mothers claim that they have not known that they are pregnant. They say that they understood that they are pregnant by the movement of the baby in their abdomen. That is the reason mothers are late for ANC care given to us when we ask them.

**Interviewer:** Do women think skilled attendance during their pregnancy helps their pregnancy?

**Interviewee:** Yes. From time to time mothers in our area are changing their attitude about ANC care. And the number of mothers receiving ANC care is increasing in our area.

**Interviewer**: Do the mothers come for all the visits in ANC care?

**Interviewee:** most of the mothers who started their ANC care usually finish their visits and deliver in our facility. We follow them and advise them to not discontinue and to not deliver at home. Most of the time those mothers deliver in our facility.

**Interviewer**: what do you think are the factors that motivate women to use ANC care**?**

**Interviewee:** I would say we have been working for a long time to create a well-coming environment for mothers in our facility. As part of that, you know almost all of the ANC services are given for free. As professionals, we are also trying to give respectful and compassionate care to mothers. If we respect the mothers they will be motivated to receive our services. I would also say health extension workers and community health organizations like the HDA have also worked with the community in reaching a consensus on the benefits of receiving medical care before during and after delivery for pregnant mothers. I believe these are the factors that are motivating women in our area for MCH care.

**Interviewer:** If women don’t come for ANC, what do you think are their reasons?

**Interviewee:** Mostly it is due to the mothers not giving proper emphasis to their health and that's due to some women lacking awareness of the benefits of ANC care. I previously said as a cluster we have done a good job in educating the mothers on the benefits of MCH care but I say believe there is also room for improvement. You know, for some women there needs to be repeated health education to motivate them. Some women are laggards and need extra motivation to get into the system.

**Interviewer: -**What do you think are the barriers to attending ANC services in your area? Do women tell financial and distance issues as a barrier?

**Interviewee: -** As told you before most of the MCH services are given for free in our facility. I have never heard mothers mention a lack of money for not using ANC care. Regarding distance, our facility is located in an accessible area to almost all areas of our catchment population. Besides, we also have health posts in between the community. Some of the ANC visits are given in health posts. Because of that, I don't think distance is a major barrier to ANC care. Regarding the quality of care, we do what we can with the available inputs there can be some shortcomings in the area of laboratory services and sometimes some medications may not be available but I think these things could be barriers but they are not major barriers. I think issues associated with awareness and health education are the major barriers to ANC care.

**Interview:** can you tell us about the traditional practice and beliefs during pregnancy?

**Interviewee:** in the past there were but currently most of the harmful traditional practices have been eradicated in our area.

**Interviewer:-**How do you see the role of community volunteers/TBAs, and health extension workers on ANC care service delivery?

**Interviewee: -** TBA and health promoters work more closely with health extension workers than with us. In the past when the number of mothers who use our services became low we tell the TBAs used to bring mothers from the community to our facility. But currently, the HDA and the promoters have not been very active in their duty according to me. I think we can say there has been a limitation in that area.

**Interviewer:** What should be done to further improve ANC care delivery in your area?

**Interviewee: -** I think if there is willingness there are many things that can be done to improve ANC service delivery. We as professionals needn't wait for the facility to come to us instead we can go to the community and ask the mothers why they don't use the services. As we do that we can also give health education to our mothers about the benefits and the risks of not receiving professional care to them and their children.

The HDA organization needs to be strengthened and they need refresher training to motivate them. They are volunteers and are not being paid, their motivation will be affected if they are not given proper attention.

**Interviewer:-**My next question is about facility delivery. Do mothers know the benefits of facility delivery in your area?

**Interviewee: -** Yes the mothers are delivering in our facility. And they are receiving all the necessary care associated with facility delivery. That is because they have now known the benefit of facility delivery through the different awareness-raising programs that have been undertaken and the health education the mothers receive during ANC care.

**Interviewer: -** what do you think are the factors that are motivating mothers to deliver at health facilities? And utilize the continuum of care?

**Interviewee: -** As I told you before we have been working for a long time to create a well-coming environment for mothers in our facility. As part of that, you know almost all of the MCH services are given for free. As professionals, we are also trying to give respectful and compassionate care to mothers. If we respect the mothers they will be motivated to receive our services. I would also say health extension workers and community health organizations like the HDA have also worked with the community in reaching a consensus on the benefits of facility delivery.

**Interviewer: -** For mothers who deliver at home what are their reasons?

**Interviewee: -** Most of the mothers give having rapid labor as a reason. They say that their labor was fast and didn't give them time to reach a health facility. They say we called an ambulance but we already delivered before it reached us. I would say this is due to the lack of birth preparedness and lack a birth plan. Mothers' especially multi-Para women don't give the necessary attention to their delivery plan.

**Interviewer: -**Did you meet mothers who told you that finance, access, and quality of care are a reason?

**Interviewee: -**No, I have not any mothers who have presented those issues for delivering at home in our area.

**Interviewer: -** How do you see the role of community volunteers/TBAs and health extension workers in facility delivery?

**Interviewee: -** I believe they are doing fairly well. They call ambulances for laboring mothers. They tell us when the mothers deliver at home, they organize forums and motivate mothers to deliver at a facility. But as I have said before these organizations need to be further strengthened and additional renewing training should be given to their members to motivate them in their efforts.

**Interviewer: -** Can you tell us how the maternal forums proceed in your area?

**Interviewee: -** Yes our forums are undertaken monthly. Mothers are called to our facility by community volunteers. In these forums health education on different maternal health topics is given. These topics are maternal nutrition, hygiene, sanitation, the benefits and risks of receiving facility care, and others to improve their awareness. We also tell these mothers to bring other women who don't come to us and to pass on the information they have got from us to those mothers who don't engage with us.

**Interviewer:** What should be done to further improve facility delivery and the continuum of care in your area?

**Interviewee:** Yes, as we have talked before if we can coordinate our efforts, we can improve facility delivery and continuum of care. If for example, the woreda health bureau can support the community organizations through training and renewal, if we as professionals show concern and adequately follow the mothers through the continuum of care better services can be delivered to mothers. If managers at high and mid-level can provide the necessary inputs in terms of drugs, and lab reagents and improve the infrastructure in our facility like water and electricity, I don't see any reason that we can't retain mothers in a continuum of care.

**Interviewer:** - What does the practice of PNC delivery look like in your facility?

**Interviewee: -** The PNC service in our facility is given for 24 hours after the mother has delivered the baby. Under the PNC service, we follow the mother s vital signs and for complications like postpartum bleeding, we also educate the mother about breastfeeding and observe her breastfeed the baby. Health education about services in the continuum of care is given and the mother will be discharged if there are no complications.

**Interviewer: -** Do mothers understand the benefit of PNC service to themselves and their newborns?

**Interviewee: -** Mothers want to leave the facility and go home in shortly after delivering the baby. They don't want to wait the mandatory 24 hours. They think that if the baby has been delivered then that is enough. But we tell them there can be complications and issues. But now most mothers do understand the benefits of PNC care.

**Interviewer: -** Can you tell us the traditional practices and beliefs during the postpartum period?

**Interviewee: -** In the past, the mothers used to feed traditional drugs to newly born babies, they also used to apply to the navel of the baby. These are very harmful to the health of the baby these practices have been practiced in our community in the past. But these practices have been reduced greatly if not eliminated from the community thanks to the efforts of our health care delivery cluster.

**Interviewer: -**How do you see the job being done by the community health development army and TBA specifically about PNC?

**Interviewee: -** I think they are doing a good job but they need to be strengthened and given renewal and refresher training.

**Interviewer: -** What do you think should be done to improve PNC care as well as the continuum of care?

**Interviewee: -** Yes, as I have said earlier before if we can coordinate our efforts, we can improve facility delivery and continuum of care. As we as professionals show concern and adequately follow the mothers through the continuum of care better services can be delivered to mothers. The most important thing is giving the right focus and coordination efforts.

**Interviewer: -** I have finished my questions. Thank you.

**Interviewee: -** Thank you.

**KII-10**

**Interviewer:** how early do women go for ANC? Why do they go at that time? Why earlier or later? How often do they go to ANC? Why do they go at that time?

**Interviewee:** in our area, pregnant women don't come to ANC early. If I have to be frank only two mothers out of ten come to in their first four months. Most of the pregnant women come after six months. According to our new working guidelines, the first contact with pregnant mothers must be created before twelve weeks. But the only women who come to ANC before twelve weeks are the ones who we identify as pregnant through HCG testing after they come to our facility for other services and we link them to the ANC service and the other few women who are well aware and who follow the advice and recommendations of our professionals and health extension workers.

**Interviewer:** Why are the pregnant mothers late for ANC care?

**Interviewee:** Mostly, it is a lack of awareness. As you know, the women in our area are less educated, and they will only recognize that they are pregnant after the fetus starts to move in their abdomen. They wouldn’t even recognize that they were pregnant after the stoppage of their menstrual cycle. That is why they come late to the health facility. However, recently, together with HEWs, we have designed a program to improve their awareness of seeking MCH care early and have begun seeing results.

**Interviewer:** among the women who attend the ANC care properly what do you think are the factors that motivate women to use ANC care**?**

**Interviewee:** the women who are more aware come for ANC care. These women have understood the benefits of receiving ANC care about the health of themselves and their babies. For example, they will know that being vaccinated for TT during their pregnancy is essential for the baby that will be delivered and so this woman with awareness come and receive the ANC care. Our health extension workers are also working tirelessly to improve the awareness of women on MCH care.

**Interviewer:** If women don’t go for ANC, what are their reasons?

**Interviewee:** yes as I said earlier, some women don't come for ANC care that is mostly associated with awareness and knowledge. These women will only come for delivery after their labor has begun or some come after delivering at home and they developed complications. When these women come. When do we ask them where their ANC was followed up? They will tell us that they don't have any ANC follow-up. After that, we will advise them that if they have followed our ANC care they wouldn't have developed these complications. And we will also teach them to educate other persons in their area about this complication caused by not having ANC care.

**Interviewer:** what about other barriers like financial and distance barriers?

Interviewee: all MCH services in our facility are given for free so I can't say there are financial barriers to our service delivery. In terms of distance, there are some areas which are far from our institution but every area in our catchment has access to roads so I can't consider distance as a barrier. Interims of the quality of service in our facility there can be some issues here and there but I don't think it's a major barrier for ANC care delivery. According to me maternal awareness and motivation are the major factors for ANC service delivery.

**Interviewer:** why do women discontinue subsequent ANC visits?

**Interviewee:** as you said some women may not complete their ANC care visits. Some may miss appointments when inquire why most of them tell us issues with household work and child care and social life reasons such as funerals and weddings which they have to attend. But most women in our facility who began their ANC care do finish.

**Interview:** can you tell us about the traditional practice and beliefs during pregnancy?

**Interviewee:** in the past there were but currently most of the harmful traditional practices have been eradicated in our area.

**Interviewer:-**How do you see the role of community volunteers/TBAs, and health extension workers on ANC care service delivery?

**Interviewee:-**in the past TBAs were the ones who deliver the mothers, but now they have been trained as promoters of facility delivery in the community. Most of the contact is between the TBAs and the health extension workers. But during labor, I have seen TBAs who brought mothers to the facility. Other TBAs have been crucial in calling an ambulance when mothers go into labor in a facility

**Interviewer:** what should be done to further improve ANC care delivery?

**Interviewee:-**as I have said earlier most mothers are not coming for all the eight visits. They miss visits. But now we have taken a stand to strengthen our maternal monthly forums. We have also prepared coffee ceremonies and different incentives to improve the participation of women in these forums. As you know if can get many mothers we will also teach them to bring other mothers to our facility. We are also planning other months of home-to-home visits by health professionals. In addition to the extension visits facility, to identify mothers who don't come to the facility and ask 5hem and urge them to come and receive the care we deliver. We already began to take these steps to improve ANC care and we are already seeing results.

Concerning the TBAs and community volunteers in addition to the awareness-raising jobs they are doing we are planning that they increase their activity of appraisal mothers in social gatherings like church gatherings and markets.

**Interviewer: -** can you tell us how the maternal forums proceed?

**Interviewee: -** yes our forums are undertaken monthly. Where pregnant mothers, lactating mothers, and delivered mothers are called to our facility by community volunteers. In these forums health education is given on the danger signs of pregnancy, the benefits of ANC care follow-up, the benefits of facility delivery, breast feeding infants, and complimentary feeding. As an incentive, we always have a coffee ceremony.

**Interviewer:-**My next question is about facility delivery. Do mothers know the benefits of facility delivery in your area?

**Interviewee: -** in the past, the mothers used to feel ashamed of delivering in the facility. They used to say we don't want to deliver on the hands of male health professionals. But now their attitude has changed greatly. Most mothers know and understand the benefits of facility delivery. This is due to the awareness increasing job done through health education in the community and also the mothers have seen the complications developing from home delivery. The mothers in our area are thankful for the service we provide regarding birth. For example, mothers will come to our facility even in the case of false labor. When we check them and talk them it's false labor we give them an option for mothers to stay in our maternal waiting room until they deliver the baby. This is due to the improved awareness of the mothers towards facility birth.

**Interviewer:-**for mothers who deliver at home, what are their reasons?

**Interviewee: -** yes some mothers do deliver at home. When mothers who delivered at home come to us we ask them why they didn’t deliver according to our EDD appointment in the facility. Most of the mothers give fast labor as a reason. They say that their labor was fast and didn’t give them time to reach a health facility. The mothers who don't have a birth preparedness plan will give birth at home due to short-onset labor. We advise these mothers about the complications of having an outside facility delivery. For instance, recently there was a mother who delivered at home and come to us with retained placenta. We admitted her and gave her IV fluids. Then we manually removed the placenta. When she improved we told her all these complications arose due to her delivering at home and we warned her that the outcomes of labor are not always positive so women must deliver in a facility. But most women are thankful for the delivery service we give.

**Interviewer:-** Do women present the reason for the lack of transport or lack of money as a reason for home delivery?

**Interviewee: -** No most of the maternal services are free and we don't charge them anything. The roads to our facility are relatively good. These cannot be the reasons for home delivery in our area. The most important reason is the lack of readiness of the mother during birth.

**Interviewer:-**How do you see the job being done by the community health development army specifically about facility delivery?

**Interviewee: -** we can say they are doing a good job. Most of the behavioral changes are brought by these organizations together with health extension workers. These community health development armies are from the community and they have a chance to educate the community on health subjects. I can say they are doing a good job.

**Interviewer:-**What do you think should be done to improve facility delivery as well as the continuum of care?

**Interviewee: -** I say supporting the MCH service more. There was an NJO called FH International that used to support us. They give food and clothing accessories like caps and socks to newly delivered babies and mothers. Those incentives motivated mothers. As you know the people are motivated by small incentives in rural areas. That program ended recently. I think we can improve satisfaction if we improve services in our lab testing area. As you can see there is no electricity in our facility. I think if we can improve our electricity power we can improve our lab services. We also don't think have a water supply that also needs to be addressed. we can do is strengthen our health education in our community. If we can collaborate And work with different stakeholders together I think we can improve the quality of services given across the continuum of MCH care.

**Interviewer:-**What does PNC service look like in your facility?

**Interviewee: -** we give PNC service in our facility by extending the stay of delivered mothers for 24 hours in the facility. During this time we follow the mother for immediate complications like PPH. We also follow the mothers' vital signs. After that, if there are no complications we send the mother home. She will be given an appointment to return for the 45^th^ day to start the EPI program for the child.

**Interviewer**: - Do mothers understand the benefit of PNC service to themselves and their newborns?

**Interviewee**:-it is difficult to say all of them understood the benefits of PNC care. Some mothers and their families want to leave the facility and go home in shortly after delivering the baby. They don't want to wait the mandatory 24 hours. They will say "Now that she has delivered we want to go home, what more do we want to wait for". They think that if the baby has been delivered then that is enough. But we tell them there can be complications like bleeding and abdominal pain in the mother. She needs to wait here and we have to observe the continued wellness of the baby and the mother. But some of the mothers and their families will not be willing to stay in the facility after delivery. we are teaching them about the benefits of PNC care and we are observing the changes in awareness and attitude about PNC care.

**Interviewer: -** if mothers don’t use PNC care what do you think their reasons are?

**Interviewee: -** Mothers miss PNC care if they don’t deliver in a facility. Otherwise, mothers who deliver in a facility always receive PNC care. If mothers deliver at home it is impossible to give them PNC care. So that the biggest reason for mothers not receiving PNC care is home delivery.

**Interviewer: -**How do you see the job being done by the community health development army and TBA specifically about PNC?

**Interviewee: -** we can say they are doing a decent Job. That is by observing some cases that they have sent to our facility. But most of the HDA service is supervised by HEWs and HEWs may know more about this question.

**Interviewer:** - What do you think should be done to improve PNC care as well as the continuum of care?

**Interviewee: -** About PNC care some mothers may be poor and may not have something to eat during their stay in our facility. I have seen mothers who have been abandoned by their husbands. We may need some flour and coffee to prepare porridge and to make them stay for them. We may need support in delivering these items. About the continuum of care, I think I have said it before. If all the stakeholders of MCH Care can coordinate their efforts with us and the community organizations, I think we can do a good job.

**Interviewer: -** I have finished my questions, thank you.

**KII-11**

**Interviewer: -** How early do women go for ANC services?

**Interviewee: -** In earlier times ANC services were given for pregnant woman after 16 weeks of their pregnancy and she has visits at 4 times until delivery. But now it has been increased to 8 visits and she should have started at 12 weeks of her pregnancy. During ANC visits pregnant women will have receive different services, like doing EDD by asking their last menstrual period, laboratory tests of blood type, providing iron-folate, and etc.

**Interviewer: -** When we can say pregnant women go to health facility at early or late?

**Interviewee: -** As standard pregnant women should have to start ANC visits at 12 weeks of their pregnancy and we provide these services accordingly, but it does not mean not providing services for women coming lately. Some women who lives far from health facility sometimes comes after 16 weeks, some women also comes at 20 weeks of pregnancy and we provide all necessary services for those women even if they comes lately.

**Interviewer: -** How often do pregnant women come to your health facility for ANC services?

**Interviewee: -** Women are come to health facility for ANC visits at 8 times during their pregnancy and first visits at 12-16 weeks, 2^nd^ visits at 20 weeks, 3^rd^ visits at 24 weeks, 4^th^ at 28 weeks, 5^th^ at 32 weeks,... last visits at 40 weeks.

**Interviewer: -** What are the other services that health professionals provide during ANC visits?

**Interviewee:** - Pregnant women come to health facility in two cases; for ANC visits and at the time when they feel any sickness. So, health professionals provides health education and counseling services about nutrition, personal hygiene and,..., in addition to different laboratory investigations and examinations.

**Interviewer: -** What are the factors that would motivate women to utilize ANC service in their pregnancy?

**Interviewee:** - In our area there are strong and functional health development army/HDA/ linkages in the kebele; 1 to 5 networks and 1 to 30 development army. In 1 to 5 networks there are regular meetings and they discussed about all health extension packages and during that time they identify pregnant women and they report to 1 to 30 network leader then, leader will also report to health extension workers. Lastly, health extension workers recording those pregnant women in registration books and they will refer them to health facility.

**Interviewer:** - Do you think that all pregnant women would receive ANC services in your area?

**Interviewee:** - No

**Interviewer:** -So, what do you think are the barriers for attending ANC services in your area?

**Interviewee:** -As I think some women move to one place to other place due to work opportunity and this might be a factor for not utilizing ANC services.

**Interviewer:** - Do you think that due to distances and access, financial problems and opportunity costs women do not go to health facility for ANC services?

**Interviewee: -** Ye**s**, some women who lives far from health facility especially women from Horameda and Soyama kebele according to our setup do not utilize ANC services due do distances and lack of money for transport services.

**Interviewer:** - Is there any socio-cultural factors that do women didn’t utilize ANC services?

**Interviewee: -** No socio-cultural related factors in our area.

**Interviewer:** -Why do women go to the facility for first ANC, but discontinue for subsequent ANC visits?

**Interviewee: -** As I think some women discontinue ANC visits due to moving one place to other place by seeking work opportunity, due to sickness, and lack of time by doing different works due to financial shortages for home needs.

**Interviewer:** -Do you think that quality of care and lack of health professionals in the health facility during working time are reasons to discontinue for subsequent ANC visits?

**Interviewee: -** No, because there are five midwifery and enough other health professionals in our health facility including doctors and they provide health services at the all-time.

**Interviewer: -** Can you tell us about the traditional practices and beliefs during pregnancy?

**Interviewee: -**No such much traditional practices and beliefs during pregnancy, but some women take traditional medicines that prepared from leafs of the trees by believing it prevents BUDAKO (evil eyes) for their pregnancy.

**Interviewer: -**Do you think religious practices that affect mothers to use ANC care during pregnancy? Explain how and why?

**Interviewee: -**No religious related practices that affect mothers to use ANC services in our area because health professionals give them health educations and also they discuss by raising all issues in their 1 to 5 networks and 1 to 30 HDA.

**Interviewer: -** Is there traditional practices and cultural norms affect mothers to use ANC care during pregnancy?

**Interviewee: -**It might be in other area but it is not in our area.

**Interviewer: -**How do you see community volunteers services provided to the community?

**Interviewee: -** Community volunteers/health development army leader/ do identify, mobilize and give education for thus pregnant women and then directly link to health post for ANC services and give reports for health extension workers. They also identify mothers who have received ANC services and not.

**Interviewer: -** What kinds of services that traditional birth attendants/TBAs/ provide to the community?

**Interviewee: -**At the earlier time they have attend delivery but at this time we use them as community volunteers and they give educations for pregnant women not to deliver at home and do to utilize ANC services.

**Interviewer: -** How the communities see the maternal health programs and health professionals? Tell me the perception about maternal health care services and perception about different care providers.

**Interviewee: -**There are public forums or community score carding systems that are the systems that members give grade for health services and health facility by quarter, and our health center on green color that is grade one. During service giving time health professionals keep the confidentiality, privacy and the dignity of the women and their approach is friendly. So, our communities were satisfied by the services that provided for them. On other hand, there are some logistic related complaints that are shortage of drugs from public but it has improved at this time.

**Interviewer: -**In your opinion, what should be improved regarding ANC services?

**Interviewee: -**As I think it is better if responsible body proved chemistry machines in laboratory room to do complete blood count /CBC/, and reagents for syphilis tests for all time and also kits for hepatitis B tests. It is better if responsible bodies prepare and give additional training for those who are fresh health professionals to fill their skill gaps.

**Interviewer: -** What kinds of services that health professionals provides for women during delivery in your health facility?

**Interviewee: -**When women come to our health facility at the time of labor we have been provide all necessary services; doing examinations, blood tests and doing follow-up until delivery. Then necessary delivery services for women, newborn care and also repairing the tear if there is and treatment at last. Applying chlorhexidine on cord cutting site for newborn child, giving vaccines that has needed to give at birth like BCG, OPV0 for all newborn children.

**Interviewer: -** What are the factors that would motivate women to utilize delivery services in their pregnancy?

**Interviewee: -**At the earlier time pregnant women do not want to health facility for delivery services by thinking that opening their body in front of health professionals is shame and not accepted by the community. But after they have gated health educations by health workers their awareness changed and they started to utilize delivery services. Other motivating factors are pregnant women forum in the kebele, and also community volunteers also do promotion work on women to use delivery services.

**Interviewer: -**If women deliver at home, what are their reasons? Explain the constraints that influenced women to utilize facility delivery services?

**Interviewee: -**Some women deliver at home if there is spontaneous labor, but they come to our health center for placenta removal and post natal care. On other hand some women also deliver at home due to lack of transport access at the time when ambulances were out services.

**Interviewer: -** Is there socio-cultural related factors that do women deliver at home?

**Interviewee: -No**

**Interviewer: -** How about distance and access?

**Interviewee: -**At some times it might be the factors for home deliver at the time when ambulance is not giving the routine services due to technical problems for the women who lives far from the health center.

**Interviewer: -** Is the financial barriers and opportunity costs do women deliver at home?

**Interviewee: -**During their last ANC visits we have told them about birth preparedness including money that needed for them, but some women didn’t gate this money due to economic problem and this is also one of the factors.

**Interviewer: -** How about quality of care and non-dignified care?

**Interviewee: -** No, because the services that has given for women during deliver is very good and we have also cross checking strategies of health professionals by doing women give suggestions during they leave the health facility after she has taken deliver services.

**Interviewer: -** Why do women go to the facility for ANC, yet mostly deliver at home?

**Interviewee: -**As I think some women move to one place to other place due to working opportunities and other factors and from those women some women deliver at home.

**Interviewer: -** As we interviewed women in the community they said that some women deliver at home due lack of quality and compassionated care in the facility and do you think that this is factor that do women deliver at home after they have received ANC visits according to your facility?

**Interviewee: -**No, because health professionals in our health facility give health services as health ethics and quality care for them.

**Interviewer: -** Can you tell us about the traditional practices and beliefs during childbirth?

**Interviewee: -**Traditional practices during childbirth are applying butter and dung on cord cutting areas of newborn, and giving AMESA for newborn child. But these practices are not common at present time.

**Interviewer: -** What kinds of services Health development army/HDA/ women do for women during deliver?

**Interviewee: -**Health development army women do many mobilizations work in the community. 1 to 5 women network leader dial phone for health center head and health extension workers for ambulance services at the time labor starts for women in the their 1 to 5 network member and also they even take this women to health facility for delivery services.

**Interviewer: -** What kinds of services Traditional birth attendants/TBA/ women do for women during deliver?

**Interviewee: -**In our area traditional birth attendants do only mobilizing women to utilize ANC and delivery services in the health facility, but they not attend deliver as earlier.

**Interviewer: -** How the communities see the maternal health programs and health professionals and do they satisfied by the services that provided for them?

**Interviewee: -**According to our health facility communities see maternal health services as good and they have satisfied by the services that provided for them.

**Interviewer: -** Do health professionals give care for women’s dignity and keep their privacy?

**Interviewee: -**Health professionals do keeping the privacy of women during service giving time and their approach is as family for women.

**Interviewer: -** Do health professionals give motivated competent and compassionated care for women in your facility?

**Interviewee: -**Yes, all health professionals on providing according to the approaches of MCC care for women.

**Interviewer: -**In your opinion, what should be improved regarding facility delivery services and continuity of care?

**Interviewee: -**As I think it is better if all necessary logistic availed by the concerned bodies, and also continuous and enough kerosene availed for ambulance to give 24hr and 7 days services for women.

**Interviewer: -** How early do women go for PNC?

**Interviewee: -**We give first postnatal care for women within 24hrs after delivery. Then we link with health extension workers for the next PNC services and HEW give these services by going to home of this woman.

**Interviewer: -**How often do women go to PNC and what kinds of services that you provide for them and their newborn child?

**Interviewee: -**In our health center we give only the 1^st^ PNC services and other remaining PNC services provided by health extension workers in their home. During this 1^st^ PNC time we check vital signs; like blood pressures of women, body temperature, checking whether there is excess bleeding or not.

**Interviewer: -** Ok, in addition to services that you provide for women, what kinds of services you provide for newborn child?

**Interviewee: -**During 1^st^ PNC period we give newborn care, applying chlorhexidine on cutting site of cord, vaccines that given at birth, and giving health education about exclusive breastfeeding until six months and also counseling and providing IPPF, lastly next appointment and services.

**Interviewer: -** Where can women gate remaining PNC services?

**Interviewee: -** Women gate next PNC services at their home by health extension workers.

**Interviewer: -**If women don’t go for PNC, what are their reasons?

**Interviewee: -**Health extension workers provide PNC services by going home to home and sometimes due to topography it is difficult to HEW to provide PNC services. On other hand some women do not gate PNC services due to the shortages of medical logistics.

**Interviewer: -** How about socio-cultural factors?

**Interviewee: -** No, socio-cultural related factors that do women didn’t use PNC.

**Interviewer: -**Can you tell us about the traditional practices and beliefs during postpartum period?

**Interviewee: -**Some women give AMESA for newborn child by believing it protects from BUDAKO (evil eyes) and is also useful for child’s growth. On other hand women also take traditional medicines by thinking it prevents abdominal cramps.

**Interviewer: -** What kinds of services Health development army/HDA/ women do for women during post natal period?

**Interviewee: -**Those leaders of women development army do follow up by going to women’s home whether any problem was happened women and her newborn child or not, if any infection for newborn child at umbilical cord they directly report for HEWs. In addition those HDA women leaders practically show how to mother take their child during breastfeeding.

**Interviewer: -** What kinds of services Traditional birth attendants/TBA/ women do for women during post natal period?

**Interviewee: -**TBAs give educations for lactating women not to give AMESA, to give exclusive breastfeeding and in generally work of promotion and education.

**Interviewer: -**In your opinion, what should be improved regarding PNC services and continuum of care?

**Interviewee: -**As I think it is better if concerned bodies provide motor bicycle for care provider and also giving priorities for the constructions of roads at kebele level.

**Interviewer: -** Lastly, in your opinion, what should be improved regarding to ANC, Delivery and PNC services?

**Interviewee: -**There is water shortage and this should have to improved, there is intermittent of electricity and this should have to improved, and lastly it is good if concerned bodies avail wireless phone in health facilities.

**Interviewer: -** I have finished my questions. Thank you.

**Interviewee: -** Thank you.

**KII-12**

**Interviewer:** How early do women go for ANC?

**Interviewee:** after three months of their pregnancy.

**Interviewer:** what service the mothers will get during the ANC visit?

**Interviewee:** first the mothers should need to be investigated.

**Interviewer:** what is that investigation about?

**Interviewee:** it may be urine, blood pressure, and there is laboratory investigation that they should investigate. Kkk

**Interviewer:** How often do they go to ANC?

**Interviewee:** she should be come four times but I don’t know if there is any update.

**Interviewer:** what service will women get if they come at health facility for ANC?

**Interviewee:** They will know the fetal presentation, receive iron pill, and check for the chronic diseases. They will obtain the service based on their appointment and ask as per the checklist

**Interviewer:** Explain factors that would motivate women to utilize ANC service in their pregnancy?

**Interviewee:** sometimes the local happened condition will motivate them. For example, if there were women who experienced the death of neonates that gave birth at home without visit or better health status of those who delivered at health facility. The health service provided for them will motivate to come again the health facility. The health professionals’ welcoming (way of handling) the mother at health facility also may be motivated and may be unaware of the benefit of ANC service.

**Interviewer:** If women do not go for ANC, what are their reasons?

**Interviewee:** first, may be lack of awareness, poor quality service, lack of benefit in previous use, if they have many children and haven’t any health problems.

**Interviewer:** What are barriers to accessing ANC like transport and distance?

**Interviewee:** yes, these are reasons. I have been worked in the kebele that haven’t entry to ambulance and they may be gave birth at road before reaching the health facility. Due to the distance they will come here after we have departed to lunch. They will go back without obtaining the service.

**Interviewer:** are socio-cultural related barriers in your locality?

**Interviewee:** yes, they believe that the placenta should be buried in house and give birth by TBAs. They say if we go to health facility the health professionals undermine us and the socio-cultural barriers are serious one.

**Interviewer:** Why do women go to the facility for first ANC, but discontinue for subsequent ANC visits?

**Interviewee:** first, they will perceive they don’t receive the service. Lack of awareness, distance, lately coming and lack of transport are may be reasons.

**Interviewer:** Can you tell us about the traditional practices and beliefs during pregnancy?

**Interviewee:** abdominal massaging using butter and fetal position correction but now stopped.

**Interviewer:** other?

**Interviewee:** ……

**Interviewer:** Can you tell us about the religious practices and beliefs during pregnancy?

**Interviewee:** pray kkkk

**Interviewer:** prophet influence?

**Interviewee:** yes, they say you will give birth without any problems. They say lord told me you will give birth without any problems. Due to this they may be remain at home. Don’t care about anywhere because they received to give birth without any health problems. It is exists currently but not previously.

**Interviewer:** How do you see community volunteers regarding ANC services provided to the community?

**Interviewee:** They highly support. The 1 to 5 networks and WDTs leader is better than the other members in their groups. The provide counsel and teach them. They motivate and support mothers to go the health post and health center. They will share the good and bad things to each other and during this time mothers will obtain education.

**Interviewer:** How do you see TBAs regarding ANC services provided to the community?

**Interviewee:** they highly support. They will motivate and bring together mothers to the health facility. They teach the previous experience and bring immediately mothers to health facility. They will provide the counsel for mothers about what they will prepare. They also provide the ambulance driver and HEWs and all the important things.

**Interviewer:** How do you see health professionals regarding ANC services provided to the community?

**Interviewee:** they will provide all care and support for mothers as per their capacity and refer those cases beyond their capacity to hospital.

**Interviewer:** In your opinion, what should be improved regarding ANC services?

**Interviewee:** the government should avail all the important things at all levels. For example, mothers should be able to obtain all service at health post level that is designed at that level without any limitation to minimize unnecessary wastage in different perspectives. The government should add one to three ambulances on currently existing two ambulances at the woreda level. The government should arrange the training to health professionals. The health professionals should support at the community levels.

**Interviewer:** what types of service the women and newborn will obtain if they come for health facility delivery services?

**Interviewee:** after they give birth without any problems we will give counseling on breast feeding, child vaccination, child care, family planning, exclusive breast up to six months and complementary feeding and hygiene.

**Interviewer:** Explain factors that would motivate women to utilize delivery service in their childbirth?

**Interviewee:** the health professional cares and the service women received at here may be motivating them. If food and drink at here, they will motivate to use service but unavailable here kkkk.

**Interviewer:** is there women waiting room at here?

**Interviewee:** yes, there is waiting room

**Interviewer:** If women deliver at home, what are their reasons?

**Interviewee:** negligence, poor knowledge, distance, unknown date of delivery, lack of proper ANC follows up, and poor satisfactions by health facility service are some of reasons.

**Interviewer:** Why do women go to the facility for ANC, yet mostly deliver at home?

**Interviewee:** if they hear the bad situation that happened by chance at health facility such as suffocated and died neonate in their area. As have mentioned earlier there is cultural issues like if I give birth at home the placenta will be buried at here and I will not stressed. They have many anxieties like fear of referral. It is combined factors.

**Interviewer:** Can you tell us about the traditional practices and beliefs during childbirth?

**Interviewee:** during home delivery they will use blade and unclean materials to cut placenta. This will lead other complication but this procedure will perform by sterile materials at here.

**Interviewer:** How do you see community volunteers regarding the health facility delivery services provided to the community?

**Interviewee:** they are alarming the mothers to give birth at health facility when the date of delivery approached. They counsel, communicate, support and teach mothers to go the health facility during the delivery.

**Interviewer:** How do you see TBAs regarding the health facility delivery services provided to the community?

**Interviewee:** they initiate and bring the mothers to health facility. The mothers are hearing their counsel more than others because they are sharing their previous bad experience.

**Interviewer:** In your opinion, what should be improved regarding facility delivery services?

**Interviewee:** the supplies. For example, in here there is no water, will not accessible at the appropriate time in all rooms. Mothers need to wash their body after they give birth but no water. This should improve in order the mothers to obtain all service. The waiting room, food and drink service should be improved to improve the health facility delivery. The necessary materials supplies should be improved in delivery rooms like bed and infant radiator.

**Interviewer:** what should be improved in the delivery rooms?

**Interviewee:** as I told you earlier these will happen at anywhere. These are supplies problems but it is not personal problems. Sometimes we will face the shortage of glove and borrow from other place.

**Interviewer:** How early do women get PNC service at here?

**Interviewee:** within 24 hours.

**Interviewer:** what types of service will get at that time?

**Interviewee:** child care. We will counsel and teach about the proper breast feeding and attachment. We will provide the BCG vaccine for the child if the dose opened. We will proper check and manage postpartum hemorrhage by taking vital sign.

**Interviewer:** How often do they go to PNC?

**Interviewee:** they will go to health facility at the 3^rd^, 5^th^ and 7^th^ day. The HEWs will check their vital sign because they will develop the preclamption after child birth at health post.

**Interviewer:** Explain factors that would motivate women to utilize PNC service in their childbirth?

**Interviewee:** if they know about PNC service they will come.

**Interviewer:** what types of service they will get after they go to health facility for PNC service?

**Interviewee:** they will get PNC service

**Interviewer:** what?

**Interviewee:** family planning and child vaccination.

**Interviewer:** If women don’t go for PNC, what are their reasons?

**Interviewee:** poorly satisfied mother by service may don’t go for PNC service.

**Interviewer:** are there any distance related barriers in your community?

**Interviewee:** yes, the distance is one reason. There are also socio-cultural related barriers.

**Interviewer:** are there any mothers-in-law and fathers-in-law related barriers in your community?

**Interviewee:** they say ‘mich newu’ and influence them not go to health facility.

**Interviewer:** are there any financial and opportunity costs related barriers in your community?

**Interviewee:** most of times they walk by their foot. However, the distance to come with child may influence them. They will never come if there is anything that requires money. They are coming because they know the child vaccination is free of charge.

**Interviewer:** are there any qualities of care related barriers in your community?

**Interviewee:** yes, they will not go to health facility after several unnecessary appointments like the vaccine may run out, BCG dose not opened, poor satisfaction by service and some health facility related issue. The far distance is another reason.

**Interviewer:** Can you tell us about the traditional practices and beliefs during postpartum period?

**Interviewee:** they add something harmful in milk.

**Interviewer:** is there anything that provide for the infant?

**Interviewee:** yes, women will give the ‘Amesa’ for their child. kkk

**Interviewer:** other?

**Interviewee:** they apply something on skin by saying the skin rash. They also provide the fluid drink for the child.

**Interviewer:** How do you see TBAs regarding PNC services provided to the community?

**Interviewee:** yes, they have role. They will teach about the harmful traditional practice. The mothers hear them because they were more experience in that regard.

**Interviewer:** How do you see community volunteers regarding PNC services provided to the community?

**Interviewee:** yes, they all have role. They will counsel and teach about the child care, breast feeding, child vaccination, and family planning. They will also motivate mothers to go to the health facility.

**Interviewer:** In your opinion, what should be improved regarding PNC services?

**Interviewee:** The government should avail all the necessary supplies, materials and ambulance. The training should be provided to the HDTs. The government should more work at health post level.

**Interviewer:** In your opinion, what supplies should be improved regarding PNC services?

**Interviewee:** The Depo injection may not be available for the long time in our health center. The mothers prefer to use this contraceptive method they are correct and that is their right. Due to its shortage they may be expose themselves to unwanted pregnancy. We try to counsel them to receive other options but they refuse.

**Interviewer:** In your opinion, what should be improved regarding the continuum of care?

**Interviewee:** They will properly obtain all service at here but there may be unknown at kebele level due to heavy workload and high number of case.

**Interviewer:** thank for the information you provided!

**Interviewee:** Thank you!

**KII-13**

**Interviewer:** how early do women go for ANC? Why do they go at that time? Why earlier or later? How often do they go to ANC? Why do they go at that time?

**Interviewee: -**Most of the mothers come very late into their pregnancy. But health extension workers try to identify pregnant women and try to send them here.

**Interviewer:** Why are the pregnant mothers late for ANC care?

**Interviewee: -** Mostly the road is tough even for normal late alone pregnant women. Our area is of tough topography.

**Interviewer:** among the women who attend the ANC care properly what do you think are the factors that motivate women to use ANC care**?**

**Interviewee: -** Mostly the mothers who attend ANC care are the ones who participate in our maternal forums and the ones who have engagement with our HDA organization.

**Interviewer:** If women don’t go for ANC, what are their reasons?

**Interviewee: -** Mostly it is due to a lack of awareness.

**Interviewer:** what about other barriers like financial and distance barriers?

**Interviewee: -**Mostly it is road and topography. Most of our services are free**.** But about the quality of care in the beginning some mothers rejected male midwives but that was improved progressively with health education.

**Interviewer:** why do women discontinue subsequent ANC visits?

**Interviewee: -**most of the mothers who begin ANC care finish in our facility because we counsel them intensively.

**Interviewee:** - once they begin their ANC most of the mothers finish their visits.

**Interview:** can you tell us about the traditional practice and beliefs during pregnancy?

**Interviewee: -** no there are none that I know of that I related with ANC care.

**Interviewer:-**How do you see the role of community volunteers/TBAs, and health extension workers on ANC care service delivery?

**Interviewee: -** maybe they are working but I didn't have personal experience with them. I have not seen this organization working on bringing mothers to the facility...

**Interviewer:** what should be done to further improve ANC care delivery?

**Interviewee: -** Strengthening health education is essential. We need to continuously educate mothers about the benefits and risks of ANC care. But one major problem in our area is the road. The topography is tough and mothers struggle to come to the facility even for delivery. If this problem is solved we will do a major favor for this area.

**Interviewer:-**My next question is about facility delivery. Do mothers know the benefits of facility delivery in your area?

**Interviewee: -** There have been improvements but there is still also a major problem with home delivery

**Interviewer:-**Besides the road access problem for mothers who deliver at home what are their reasons?

**Interviewee: -** The reasons are not clear but mostly it is a lack of birth preparedness and not having adequate awareness about professional care. But we are doing the best we can to solve this...

**Interviewer: -** Do women present a reason for the lack of transport or lack of money as a reason for home delivery?

**Interviewee: -** Besides the road and access problems I have previously mentioned. I don’t think these are significant barriers as most of our service is delivered for free.

**Interview**: can you tell us about the traditional practice and beliefs during pregnancy?

**Interviewee: -** I have seen some mothers who deliver at home where they intensively rub the mother's abdomen with better Vaseline. I have seen this practice cause distress in the fetus. Immediately after the baby is delivered some women feed the baby certain herbs in our area. These are the traditional practices we have seen.

**Interviewer:-**How do you see the job being done by the community health development army specifically about facility delivery?

**Interviewee: -** maybe I don’t know but those organizations were functioning very well initially but currently it looks like they have failed.

**Interviewer:-**What do you think should be done to improve facility delivery as well as the continuum of care?

**Interviewee: -**we need to extensively educate the mothers on the health benefits. Plus these areas are remote and giving incentives works. There was a certain NGO that gave out children's clothing and food to the newly delivered mothers who delivered in the facility. But that NGO has phased out. And if all the stakeholders would work and coordinate I think we can improve the retention of clients on the continuum of care.

**Interviewer:-**What does PNC service look like in your facility?

**Interviewee: -** after the mothers deliver here. We follow them. when the mothers go home the health extensions follow them.

**Interviewer**: - Do mothers understand the benefit of PNC service to themselves and their newborns?

**Interviewee: -** some of the mothers understand but there is a sizable problem with home delivery.

**Interviewer: -** if mothers don’t use PNC care what do you think their reasons are?

**Interviewee: -** Yes, they come and follow when we give them an appointment.

**Interviewer: -** if mothers don’t use PNC care what do you think their reasons are?

**Interviewee: -**Home deliveries and lack of awareness are the major hurdles for PNC care...

**Interviewer: -**How do you see the job being done by the community health development army and TBAs specifically about PNC?

**Interviewee: -** as I have told you before if we can strongly educate the mothers on every maternal topic and they helped greatly in achieving this. Health education can improve a lot of services here.

**Interviewer: -** I have finished my questions, Thank you!

**KII-14**

**Interviewer:** How early do mothers go for ANC in your locality?

**Interviewee:** most of the time they will come after four months of their pregnancy. Some mothers will come after 2 months of their pregnancy.

**Interviewer:** why do they go at that time?

**Interviewee:** they expect the proper care from here. We will give them counseling and the TT vaccine.

**Interviewer:** what other services do you provide other than what you mentioned above?

**Interviewee:** we will give iron pills, TT vaccine, and deworming tablet after 7 months of their pregnancy.

**Interviewer:** Why earlier or later you mentioned the time above?

**Interviewee:** I think those mothers who come lately for ANC will not complete the recommended ANC follow-up till the date of delivery. Previously the recommended ANC follow-up is 4 but currently changed to 8 visits.

**Interviewer:** How often do they go to ANC?

**Interviewee:** most of them will come only two times and some will come four times till the date of delivery. They will not be able to complete the recommended ANC because they always start ANC visits late in pregnancy.

**Interviewer:** Do women think skilled attendance during pregnancy helps their pregnancy?

**Interviewee:** a majority of them think it is useful but some women think it is unnecessary. Currently, we are strengthening our pregnant women forum to counsel and create awareness for them. However, 60% of them think it is useful. The remaining thing is unnecessary by saying that our mothers were not visited health facilities so that is why we go there.

**Interviewer:** Explain factors that would motivate women to utilize ANC services during their pregnancy.

**Interviewee:** the health professionals and HEWs will motivate them to go here. Also, we will motivate them during the pregnant women forum.

**Interviewer:** Explain if women do not go for ANC, what are their reasons?

**Interviewee:** the lack of awareness and attitude problem but there is no on our side.

**Interviewer:** others?

**Interviewee:** no

**Interviewer:** Are there any financial and distance-related barriers in your area?

**Interviewee:** yes, there is Kebele so called Hayisa Baraha which is too far from the health center. Also, the topography is a serious problem in our catchment. They calculate the problems like distance, absence of health professionals in a health facility, and lack of electricity and vaccine. Some mothers will not come due to these reasons.

**Interviewer:** Are there any socio-cultural-related barriers in your locality?

**Interviewee:** no

**Interviewer:** Are there any mother and father-in-law-related barriers in your locality?

**Interviewee:** no. However, old mothers prefer to deliver at home.

**Interviewer:** is there any quality of care-related barriers in your locality?

**Interviewee:** no

**Interviewer:** Why do women go to the facility for the first ANC, but discontinue for subsequent ANC visits?

**Interviewee:** in the first place they will come here due to some health problem they faced like back pain. After they received the first ANC they will evaluate the effect of the vaccine in terms of curing their back pain. If the vaccine hasn't any effect on their back they will evaluate it as useless and then discontinue the subsequent ANC visits.

**Interviewer:** Are there any distance and topography-related barriers in your locality?

**Interviewee:** yes, the distance and topography are barriers for some mothers.

**Interviewer:** Can you tell us about the traditional practices and beliefs during pregnancy?

**Interviewee:** no, nothing in our community.

**Interviewer:** Can you tell us about the traditional and religious beliefs and cultural norms during pregnancy?

**Interviewee:** no, nothing in our community.

**Interviewer:** How do you see TBAs regarding ANC services provided to the community?

**Interviewee:** now they aren’t prevailing in our community.

**Interviewer:** How do you see community volunteers regarding ANC services provided to the community?

**Interviewee:** They are motivating the pregnant women in the community to use ANC service.

**Interviewer:** other?

**Interviewee:** They are motivating the laboring women, husbands, and family to come here. Sometimes they will come here and take stricter to bring the laboring women together with their families.

**Interviewer:** How do you see healthcare professionals regarding ANC services provided to the community?

**Interviewee:** They are providing care for pregnant women. They also go to Kebeles during the pregnant women's forum and provide counseling. Further, they provide an outreach vaccination service for those mothers who are far from home.

**Interviewer:** In your opinion, what should be improved regarding ANC services?

**Interviewee:** in our area, the pregnant women forum is undergoing one time per month. In my opinion, if the forum is held two times per month the ANC service will improve.

**Interviewer:** other?

**Interviewee:** during the forum time if the tea and coffee ceremony available will improve the ANC service.

**Interviewer:** other?

**Interviewee:** I think it is enough.

**Interviewer:** Do women think skilled attendance during childbirth helps them and their babies?

**Interviewee:** yes, they think. The majority of them think it is necessary but some don't think it is important.

**Interviewer:** why do those women think skilled attendance during childbirth helpless?

**Interviewee:** it is their attitudinal problem.

**Interviewer:** Explain factors that would motivate women to utilize delivery services during their pregnancy.

**Interviewee:** those mothers who gave home birth for their first pregnancy will fear the severe abdominal pain they faced. Whereas those who gave birth at a health facility in their first pregnancy acknowledge the care they obtained from health care providers and motivate themselves to use the service again in the health facility. Those who delivered at a health facility will tell us they had minimal abdominal pain due to we clean their uterus to become free of blood after delivery service.

**Interviewer:** Explain factors that would motivate women to utilize delivery service in their pregnancy after they follow ANC here.

**Interviewee:** the benefits they obtained during the ANC from here motivate them.

**Interviewer:** other?

**Interviewee:** ………

**Interviewer:** If women deliver at home, what are their reasons?

**Interviewee:** they say thanks to God! We have delivered the previous child at home without any problems. So why would go there if we haven't any problems during childbirth**?**

**Interviewer:** Are there any financial and opportunity costs related barriers in your locality?

**Interviewee:** yes, some mothers will not come here due to financial problems.

**Interviewer:** Are there any distance-related barriers in your locality?

**Interviewee:** yes, some women worry about the far distance after the labor started and how I could arrive at a health facility. They fear may the delivery will happen on the road without reaching the health facility.

**Interviewer:** Are there any qualities of care and non-dignified care-related barriers in your area?

**Interviewee:** yes, some women will prefer to deliver at home due to the non-dignified care at the health facility. However, the majority of mothers are using health facility delivery.

**Interviewer:** Why do women go to the facility for ANC, yet mostly deliver at home?

**Interviewee:** they say the labor was short. The child was delivered while I am preparing to go health facility suddenly.

**Interviewer:** Are there any qualities of care and non-dignified care-related barriers in your area?

**Interviewee:** more than distance and finance the non-dignified care will influence them to give birth at home.

**Interviewer:** Can you tell us about the traditional practices and beliefs during childbirth?

**Interviewee:** no, nothing in this community.

**Interviewer:** Can you tell us about the traditional beliefs and cultural norms during childbirth?

**Interviewee:** no, nothing in this community.

**Interviewer:** Can you tell us about the religious practice during childbirth?

**Interviewee:** no, nothing in this community.

**Interviewer:** How do you see TBAs regarding health facility delivery services provided to the community?

**Interviewee:** no, I can't see them in this community.

**Interviewer:** How do you see community volunteers regarding health facility delivery services provided to the community?

**Interviewee:** as I have mentioned to you earlier the community volunteers will motivate the pregnant **Interviewer:** women to use ANC service and laboring women to deliver service at health facilities.

How early do women go for PNC?

**Interviewee:** Most of the time, they will not come to the health facility for PNC services. However, they will come on the 45th day for the child's vaccination and family planning service.

**Interviewer:** why do they go on the 45^th^ day?

**Interviewee:** they will come on the 45^th^ day as per our counseling. They will use child vaccines and family planning services.

**Interviewer:** When do you check mothers at home after they give birth?

**Interviewee:** we will check on the 3^rd^ day and 7^th^ day.

**Interviewer:** did you visit earlier or later than that day?

**Interviewee:** we never go there but they will come if they face problems.

**Interviewer:** How often do they go to PNC?

**Interviewee:** four times

**Interviewer:** why did they go to PNC four times?

**Interviewee:** to know their health status

**Interviewer:** Do women think skilled attendance during postpartum help their babies and themselves?

**Interviewee:** they think it is useful. 90% of them think it is useful.

**Interviewer:** Explain factors that would motivate women to utilize PNC services in their childbirth.

**Interviewee:** the good service provided during the health facility delivery care will motivate them to come for PNC service.

**Interviewer:** other?

**Interviewee:** the health problems they faced and any confusion about their health will motivate them to come here. They will ask questions and get answers from the health professionals regarding their health problems.

**Interviewer:** What are the barriers to accessing PNC?

**Interviewee:** the far distance influences them not to come here.

**Interviewer:** Are there socio-cultural barriers to accessing PNC?

**Interviewee:** no

**Interviewer:** Is there quality of care barriers to accessing PNC?

**Interviewee:** yes, lack of quality of service will influence them to access PNC.

**Interviewer:** Why do women go to the delivery at the facility, yet most don't receive PNC?

**Interviewee:** one reason is far distance. Second, the old people will prevent mothers not to go outside the home due to socio-cultural and attitudinal problems. Most people think women will expose to 'mich’ or ‘Buda’ if they go outside their homes during the postpartum period.

**Interviewer:** Can you tell us about the traditional practices and beliefs during the postpartum period?

**Interviewee:** I don’t know anything so far.

**Interviewer:** How do you see TBAs regarding postnatal care services provided to the community?

**Interviewee:** I don’t know any TBAs

**Interviewer:** How do you see community volunteers regarding postnatal care services provided to the community?

**Interviewee:** they motivate those mothers who have road access to use the PNC service

**Interviewer:** How do you see health professionals regarding postnatal care services provided to the community?

**Interviewee:** we will give PNC services after they come like counseling and other things that need for them.

**Interviewer:** In your opinion, what should be improved regarding PNC services?

**Interviewee:** previously there are things that mothers will obtain after they give birth like cups and towels. These are motivating things mothers to use service and should be availed to motivate mothers.

**Interviewer:** other?

**Interviewee:** the improved service here motivates them.

**Interviewer:** what should be improved from the side of the government?

**Interviewee:** The materials like cups and towels for the children should be availed if possible because these materials are unavailable here.

**Interviewer:** what should be improved from the sides of a community?

**Interviewee:** community mobilization by using the Kebeles leader is very important to motivate women to use PNC.

**Interviewer:** what should be improved from the side of health professionals?

**Interviewee:** health professionals should be provided adequate counseling for mothers.

**Interviewer:** what should be improved from the sides of HEWs?

**Interviewee:** the HEWs should provide the home to home visits for mothers.

**Interviewer:** others?

**Interviewee:** ……………

**Interviewer:** what should be improved from the sides of HEWs?

**Interviewee:** the HEWs

**Interviewer:** In your opinion, what should be improved regarding the continuum of care?

**Interviewee:** if the quality of service provided for them improved and benefits they will use a continuum of care properly.

**Interviewer:** other?

**Interviewee:** the road should be properly built and accessible for them

**Interviewer:** any things that you would like to add to our discussion?

**Interviewee:** I have finished

**Interviewer:** thank you!

**KII-15**

**Interviewer:** how early do women go for ANC? Why do they go at that time? Why earlier or later? How often do they go to ANC? Why do they go at that time?

**Interviewee: -** Most of the mothers come late for ANC. Most of the ANC is late ANC in a facility.

**Interviewer:** Why are the pregnant mothers late for ANC care?

**Interviewee: -**Mostly it is associated with a lack of awareness and knowledge. Those who have awareness utilize care but those who don't utilize it.

**Interviewer:** among the women who attend the ANC care properly what do you think are the factors that motivate women to use ANC care**?**

**Interviewee: -**those who have awareness utilize care. It's having adequate awareness.

**Interviewer:** If women don’t go for ANC, what are their reasons?

**Interviewee: -**issues of lack of awareness. Most of our services are free. But about the quality of care, we sometimes lack a supply of vital inputs. Like medicine and lab regents. Once some women are dissatisfied they go and disseminate negative information. That holds mothers back.

**Interviewer:** what about other barriers like financial and distance barriers?

**Interviewee:** yes, these barriers are affecting mothers. Most mothers use motorbikes for transport and transportation costs have grown over time. Some areas are distant and motors may feel not motivated to make multiple trips to the facility. These barriers are significant but mostly it is associated with lack of knowledge and awareness.

**Interviewer:** why do women discontinue subsequent ANC visits?

**Interviewee:** Most of them tell us issues with household work and child care and social life reasons such as funerals and weddings which they have to attend.

**Interview:** can you tell us about the traditional practice and beliefs during pregnancy?

**Interviewee:** I didn’t come across any but there can be in the community

**Interviewer:-**How do you see the role of community volunteers/TBAs, and health extension workers on ANC care service delivery?

**Interviewee:** Yes, they are working. They say they educate women and bring them to forums. But it has not been as effective as it was in the beginning.

**Interviewer:** what should be done to further improve ANC care delivery?

**Interviewee:** we should give them health education. We need to go to the community. We should also improve the quality of care. we need to have all the necessary supplies when they are needed.

**Interviewer:-**My next question is about facility delivery. Do mothers know the benefits of facility delivery in your area?

**Interviewee:** when they come to deliver here they are happy. The ones who use are happy and understand it helps them and their babies. The factor that motivates them is the health education given by health extension workers. The mothers having awareness are motivated.

**Interviewer:-**for mothers who deliver at home, what are their reasons?

**Interviewee:** mostly it is a misconception and they claim 'our mothers give birth at home so we don't need it to'. Sometimes they call an ambulance and the ambulance gets late and they have delivered at home.

**Interviewer: -** Do women present a reason for the lack of transport or lack of money as a reason for home delivery?

**Interviewee:** yes I have already said the ambulance issues. Concerning the quality of care I have met some mothers who claimed that they didn't like delivering at the facility because of the lack light of in our facility.

**Interviewer:-**How do you see the job being done by the community health development army specifically about facility delivery?

**Interviewee:** I can say they didn’t do a good job. If they have worked the mothers would have come for all our services. I think they need to be reorganized and need to work again

**Interviewer:-**What do you think should be done to improve facility delivery as well as the continuum of care?

**Interviewee:** as I have previously said they didn’t do a good job. If they have worked the mothers would have come for all our services. I think they need to be reorganized and need to work again.

**Interviewer:-**What does PNC service look like in your facility?

**Interviewee: -** We make the mothers wait at our facility. We follow them for complications. We also counsel the mothers on post-natal FP and provide the service as necessary. We also observe their breastfeeding and advise them.

**Interviewer**: - Do mothers understand the benefit of PNC service to themselves and their newborns?

**Interviewee: -** Most of them understand but some others lack the proper awareness.

**Interviewer: -** If mothers don’t use PNC care what do you think their reasons are?

**Interviewee: -** Mostly due to home delivery and lacking awareness.

**Interviewer: -**How do you see the job being done by the community health development army and TBA specifically about PNC?

**Interviewee: -** as I have told you before we educate the mothers on every maternal topic and they helped greatly in achieving this.

**Interviewer:** - What do you think should be done to improve PNC care as well as the continuum of care?

**Interviewee: -** We need to coordinate our efforts with the community and give continuous health education and awareness creation activities. The quality of care at the facility needs to be improved by supplying the necessary inputs and improving infrastructure. The mothers should be tough and urged to have birth preparedness and birth plan.

**Interviewer: -** I have finished my questions, thank you!

**Interviewee: -**Thank you!
