# Supplementary material for: Perceptions, barriers, and facilitators of maternal health service utilization in southern Ethiopia: A qualitative exploration of community members’ and health care providers’ views
Source: PLoS One. 2024 Dec 19;19(12):e0312484. doi: 10.1371/journal.pone.0312484 (PMC11658624; doi:10.1371/journal.pone.0312484)
Supplement: Attachment — Submitted filename: Response to Reviewers.docx [file pone.0312484.s005.docx]

***Point-by-point responses to editor’s comments***

**Comment 1:** The abstract section line 22 on methods should avoid showing the dates and can be described more succinctly by saying the study was conducted in the month of November. Kindly change that

**Authors’ response:** Thank you a lot for this valuable comment. We have accepted the comment and made the required revision.

**Comment 2:** In Line 31 MAXQDA has been written as MAXQAD that too should be changed.

**Authors’ response**: Thank you for your vital comment and for fixing our mistake. Now we have revised it based on your edition in the abstract and data analysis section of methods.

**Comment 3:** In line 86 you use unconventional abbreviations such as MHSU for Maternal Health Service Utilization please avoid this and use the correct verbs to describe utilization. You can easily describe utilization without the initials.

**Authors’ response**: Dear Editor, thank you for your important comment. Now we have used the term “Maternal Health Service Use” consistently throughout the manuscript.

**Comment 4:** Lines 346-349 should be contained in a table of characteristics of the respondents. This will make it more readable.

**Authors’ response**: Dear Editor, thank you for your critical comment. Now we have provided the table that indicates characteristics of the study respondents, as per your suggestion to make the results more visible for readers.

**Journal requirements**

**Requirement:** Please review your reference list to ensure that it is complete and correct. If you have cited papers that have been retracted, please include the rationale for doing so in the manuscript text, or remove these references and replace them with relevant current references. Any changes to the reference list should be mentioned in the rebuttal letter that accompanies your revised manuscript. If you need to cite a retracted article, indicate the article’s retracted status in the References list and also include a citation and full reference for the retraction notice.

**Authors’ response:** All required revisions have been done to ensure that all references utilized in this manuscript are complete and correct, and the manuscript is updated accordingly. Now we are sure that all references are complete and correct and that no retracted articles were included.
